# Identification of Functional Candidates amongst Hypothetical Proteins of *Treponema pallidum ssp*. *pallidum*

**DOI:** 10.1371/journal.pone.0124177

**Published:** 2015-04-20

**Authors:** Ahmad Abu Turab Naqvi, Mohd Shahbaaz, Faizan Ahmad, Md. Imtaiyaz Hassan

**Affiliations:** 1 Department of Computer Science, Jamia Millia Islamia, Jamia Nagar, New Delhi—110025, India; 2 Center for Interdisciplinary Research in Basic Sciences, Jamia Millia Islamia, Jamia Nagar, New Delhi—110025, India; University of Copenhagen, DENMARK

## Abstract

Syphilis is a globally occurring venereal disease, and its infection is propagated through sexual contact. The causative agent of syphilis, *Treponema pallidum ssp*. *pallidum*, a Gram-negative sphirochaete, is an obligate human parasite. Genome of *T*. *pallidum ssp*. *pallidum* SS14 strain (RefSeq NC_010741.1) encodes 1,027 proteins, of which 444 proteins are known as hypothetical proteins (HPs), i.e., proteins of unknown functions. Here, we performed functional annotation of HPs of *T*. *pallidum ssp*. *pallidum* using various database, domain architecture predictors, protein function annotators and clustering tools. We have analyzed the sequences of 444 HPs of *T*. *pallidum ssp*. *pallidum* and subsequently predicted the function of 207 HPs with a high level of confidence. However, functions of 237 HPs are predicted with less accuracy. We found various enzymes, transporters, binding proteins in the annotated group of HPs that may be possible molecular targets, facilitating for the survival of pathogen. Our comprehensive analysis helps to understand the mechanism of pathogenesis to provide many novel potential therapeutic interventions.

## Introduction

*Treponema pallidum ssp*. *pallidum* is experimentally investigated to be the cause of venereal syphilis, a globally existing sexually transmitted disease (STD) [1–4]. *T*. *pallidum ssp*. *pallidum* is a Gram-negative bacterium, classified as a member of family Spirochaetaceae [5]. The syphilis infection is frequently transmitted through sexual contacts, which results in the pandemic of this particular disease [6]. The primary effects of infection can be seen as skin lesions on the site of infection [4]. The secondary and tertiary stages of syphilis are assumed to be lethal because of the prevalence of the organism in the body of host [7,8]. The infection of syphilis is severe in nature as 12 million new cases of venereal syphilis were reported by World Health Organization in the year 1999 with most of the cases were from the developing countries [4].

The SS14 strain of *T*. *pallidum ssp*. *pallidum* was first isolated from the skin lesion of a patient with secondary syphilis [2,9]. The genome sequence of *T*. *pallidum ssp*. *pallidum* is available in the NCBI database containing 1,087 genes encode 1,027 proteins. Among these, function of 444 proteins are not experimentally determined so far, and are termed as hypothetical proteins (HPs). A hypothetical protein is one predicted to be encoded by an identified open reading frame, but for which no protein product has been confirmed or characterized. [10]. However, HPs possibly play important roles in the survival of pathogen, and hence disease progression [10,11]. Since, it is very difficult to work on *T*. *pallidum ssp*. *pallidum* because of its complete obligate dependence on a mammalian host system to survive in the environment. Therefore, genomic sequence of *T*. *pallidum ssp*. *pallidum* offers a wealth of basic information which can be further analyzed to extract useful information [3]. A precise function of HPs from several pathogenic organism have been reported already using sequence and structure based methods [11–14].

The already sequenced genome of the *T*. *pallidum ssp*. *pallidum* was taken in our study to explore the function of these HPs with high precision using well optimized bioinformatics tools described elsewhere [15]. To predict function of HPs with high confidence, their sequences are retrieved from the NCBI and analyzed by using various bioinformatics tools for the prediction of physicochemical properties, sub-cellular localization, sequence similarity search, virulence factor prediction, etc. Moreover, HPs may act as potential virulent factors which may be predicted by bioinformatics tools and targeted further for the structure based rational drug design [16–20]. The predicted functions of HPs are further validated by using a statistical technique like ROC (Receiver operating characteristic) that is helpful to assess the performance of used bioinformatics tools. We believe that such analyses expand our knowledge regarding the functional roles of HPs of *T*. *pallidum ssp*. *pallidum* and provide an opportunity to discover novel potential drug targets [21].

## Materials and Methods

Here we used our well optimized series of tools for the functional annotation of HPs [11,15,22]. The sequences of all HPs were obtained from the NCBI (http://www.ncbi.nlm.nih.gov/genome/741). The sequences of all 444 HPs were retrieved using their primary accession numbers in FASTA format from Uniprot database (http://www.uniprot.org/).

### Analysis of physicochemical properties

Physicochemical parameters of all HPs were analyzed using Expasy’s ProtParam server (http://web.expasy.org/protparam/). This online server performs the theoretical measurement of various physicochemical parameters such as molecular mass, isoelectric point, extinction coefficient, instability index, aliphatic index and grand average of hydropathicity (GRAVY). The predicted properties of HPs are listed in the S1 Table.

### Sub-cellular localization

The precise estimation of sub-cellular localization (such as cytoplasm, periplasm, inner membrane, outer membrane and extracellular space) of a protein is helpful in predicting its function at the cellular level. Previous studies show that a protein present in the cytoplasm is a drug target. While membrane proteins found on the surface are considered to be a vaccine targets [23]. Array of online subcellular localization software is used to predict the location of HPs in the *T*. *pallidum ssp*. *pallidum*. PSORTb CELLO (v2.5) and PSLpred are effective tools to predict the subcellular localization of a particular protein. The SignalIP4.1 was used to predict signal peptide cleavage sites. SecretomeP2.0 was used to predict non-classical protein secretion, i.e., signal peptide independent secretion. TMHMM and HMMTOP were used to predict transmembrane helices in proteins as it is helpful in identification of the membrane proteins. Detailed information on subcellular localization is listed in S2 Table.

### Sequence comparisons

In order to search for known functional homologues of HPs, we performed sequence similarity searching using BLASTp against non-redundant (nr) database of proteins. We have performed HMM based similarity search using HMMSCAN, a module of HMMER server used to search for a similar domain and families. It works as an interface for searching the Pfam, TIGRFAMs, Gene3D and superfamily databases of protein families and domains. Results of sequence comparison are listed in the S3 Table.

### Domain and function assignment

Proteins are classified into families and superfamily on the basis of their sequence, structure and function by various protein classification tools like CATH, SCOP, etc. Here, we used varieties of tools to predict the function of HPs. We have also used PANTHER, a database distinguishing proteins in families and subfamilies, which provides GO based function assignment of the protein. Furthermore, Pfam database was used to predict the function of proteins based on sequence similarity. We have also performed protein classification using clustering techniques using SYSTERS and ProtoNet. SYSTERS is a database of protein family which uses BLASTp to search the database for similar sequences and provides the cluster of proteins formed on the basis of functional similarity. However, the ProtoNet provides hierarchical classification of proteins. CDART tool was used to search the conserved domains in HPs which searches the query sequence against Conserved Domain Database (CDD). We have also analyzed HPs using Simple Modular Architecture Research Tool (SMART) which predicts the function of a protein based on the domain architecture. The motif search in protein sequences was done by using InterProscan, which searches various available databases for function prediction. Results of function prediction based on these tolls are listed in the S4 Table.

### Virulence factor analysis

Identification of bacterial virulence factors can help to understand the mechanism of pathogenesis and search for potential therapeutic targets [23,24]. We used VICMpred [25] and VirulentPred [26] for identification of HPs which may be responsible for virulence in the *T*. *pallidum* *ssp*. *pallidum*. Virulent HPs from *T*. *pallidum ssp*. *pallidum* are listed in the S5 Table.

### Prediction of protein interaction network

Functional association among proteins is necessary to complete any biological process, therefore, the knowledge of protein-protein interaction is also helpful for prediction of function of a protein. Here we have used STRING (version-9.1) [27] to predict the proteins which show interaction with HPs and hence its involvement in a particular metabolic process.

### Performance assessment

The predicted functions of HPs from the genome of *T*. *pallidum ssp*. *pallidum* are validated using the receiver operating characteristic (ROC) analysis. This statistical analysis is performed using 100 sequences of proteins with known function (S6 Table). Functions of these proteins are predicted using the adopted pipeline for the annotation of the HPs. The diagnostics efficacy is evaluated at six levels. The true positive or true negative prediction is classified as ‘‘0” or ‘‘1” binary numerals. In addition, 1, 2, 3, 4 and 5 is the adopted confidence ratings. The average accuracy of the used pipeline is found to be 93.91% (S8 Table). ROC analysis indicates high reliability of bioinformatics tools used here (S7 and S8 Tables).

The level of confidence for each prediction is assumed on the basis of number of tools predicting similar function. For a particular HP, if its similar function was clearly given by four and more tools, then such prediction was considered as output with high level of confidence. Whereas if the function predicted by less than four tools, we have not included these HPs in the Table 1. Although, we separately provided a table for function prediction at low level of confidence in the S9 Table.

**Table 1:** Functionally annotated HPs from *T*. *pallidum ssp*. *pallidum*.

Protein name	GeneID	Uniprot ID	Function
HP TPASS_0017	6333127	B2S1W5	Tetratricopeptide repeat containing protein
HP TPASS_0022	6333189	B2S1X0	Helicase C terminal domain protein
HP TPASS_0024	6333763	B2S1X2	Potassium ion(K+) transporter
HP TPASS_0025	6332893	B2S1X3	Peptidase M16(Metalloenzyme)
HP TPASS_0042	6333174	B2S1Z0	Peptidoglycan binding(LysM domain- bacterial cell wall degradation)
HP TPASS_0046	6332886	B2S1Z4	PSP1 C-terminal(polymerase suppressor 1)
HP TPASS_0048	6333172	B2S1Z6	Polymer forming cytoskeletal
HP TPASS_0049	6332885	B2S1Z7	Peptidoglycan hydrolase(Peptidase M23)(LytM domain)
HP TPASS_0050	6333168	B2S1Z8	Phosphoribosyl transferase(PRTase)
HP TPASS_0054	6333745	B2S202	RNA 2’-O ribose methyltransferase(Substrate binding)
HP TPASS_0055	6332884	B2S203	Oxaloacetate decarboxylase (gamma subunit)
HP TPASS_0064	6332880	B2S212	Alpha-ketoacid dehydrogenase kinase(N terminal)
HP TPASS_0065	6333159	B2S213	S-adenosyl-L-methionine-dependent methyltransferases
HP TPASS_0066	6333156	B2S214	Tetratricopeptide repeat containing protein
HP TPASS_0067	6332879	B2S215	Tetratricopeptide repeat containing protein
HP TPASS_0068	6333820	B2S216	Ribosomal RNA large subunit methyltransferase N (Radical SAM enzyme)
HP TPASS_0072	6333819	B2S220	Glutaredoxin
HP TPASS_0073	6333821	B2S221	Metal dependent phosphohydrolases with conserved 'HD' motif
HP TPASS_0079	6333203	B2S227	Xanthine dehydrogenase(Molibdoprotein binding)
HP TPASS_0081	6332890	B2S229	Xanthine dehydrogenase(Molibdoprotein binding, FAD binding)
HP TPASS_0083	6333811	B2S231	glycosyl hydrolase
HP TPASS_0084	6333817	B2S232	Thioredoxin
HP TPASS_0086	6333164	B2S234	PilZ domain containing protein(c-di-GMP binding)
HP TPASS_0095	6332871	B2S243	Tetratricopeptide repeat containing protein
HP TPASS_0121	6333137	B2S269	Lysine-2,3-aminomutase
HP TPASS_0123	6332867	B2S271	Tetratricopeptide repeat containing protein
HP TPASS_0126	6333787	B2S274	Outer membrane protein (beta-barrel domain)
HP TPASS_0139	6333134	B2S287	Potassium ion(K+) transporter(NAD(P) binding)
HP TPASS_0151	6332899	B2S298	NADH-quinone reductase(NQR2/RnfD)
HP TPASS_0153	6333195	B2S2A0	Acid phosphatase/vanadium-dependent haloperoxidase
HP TPASS_0154	6333194	B2S2A1	RNA pseudouridylate synthase
HP TPASS_0156	6333198	B2S2A3	4-hydroxybenzoyl-CoA thioesterase
HP TPASS_0157	6333190	B2S2A4	Glycerol-3-phosphate O-acyltransferase
HP TPASS_0158	6333188	B2S2A5	Haloacid dehalogenase
HP TPASS_0181	6333597	B2S2C8	Septum formation initiator
HP TPASS_0182	6333036	B2S2C9	Telomere recombination(Sua5_yciO_yrdC family)
HP TPASS_0223	6333564	B2S2H0	Aspartate aminotransferase (EC 2.6.1.1))
HP TPASS_0226	6333555	B2S2H3	Cobalt transport protein
HP TPASS_0231	6333017	B2S2H8	RNA pseudouridylate synthase
HP TPASS_0245	6333539	B2S2J2	P-loop containing nucleoside triphosphate hydrolases
HP TPASS_0246	6333538	B2S2J3	von Willebrand factor, type A(adhesive plasma glycoprotein)
HP TPASS_0253	6333526	B2S2K1	Polymer forming cytoskeletal
HP TPASS_0259	6333527	B2S2K7	Peptidoglycan binding(LysM domain- bacterial cell wall degradation)
HP TPASS_0260	6333524	B2S2K8	SH3-like domain, bacterial-type
HP TPASS_0263	6332801	B2S2L1	Fibronectin, type III
HP TPASS_0267	6333000	B2S2L5	Polymer forming cytoskeletal
HP TPASS_0268	6333517	B2S2L6	Tetratricopeptide repeat containing protein
HP TPASS_0269	6333513	B2S2L7	Methylthiotransferase, N-terminal(Radical SAM enzyme)
HP TPASS_0282	6332995	B2S2N0	Tetratricopeptide repeat containing protein
HP TPASS_0285	6333505	B2S2N3	Iron-Sulfer cluster binding protein(SPASM)
HP TPASS_0289	6332992	B2S2N7	S-adenosyl-L-methionine-dependent methyltransferases
HP TPASS_0290	6333501	B2S2N8	Haloacid dehalogenase
HP TPASS_0291	6333499	B2S2N9	FMN-dependent dehydrogenase
HP TPASS_0296	6333498	B2S2P4	Dephospho-CoA kinase
HP TPASS_0297	6333495	B2S2P5	sporulation and cell division repeat protein
HP TPASS_0301	6332987	B2S2P9	Branched chain Amino acid ABC transporter(Permease)
HP TPASS_0302	6333496	B2S2Q0	Branched chain Amino acid ABC transporter(Permease)
HP TPASS_0304	6333493	B2S2Q2	Peptidase MA
HP TPASS_0307	6332982	B2S2Q5	PASTA domain containing protein(penicillin binding- serine/threonine kinase)
HP TPASS_0310	6332983	B2S2Q8	single-stranded DNA-binding protein
HP TPASS_0333	6333459	B2S2T0	outer membrane lipoprotein carrier protein (LolA)
HP TPASS_0334	6332972	B2S2T1	DNA-binding helix-turn-helix protein(transcriptional regulator)
HP TPASS_0335	6333460	B2S2T2	CAAX amino terminal protease(Self Immunity)
HP TPASS_0339	6333457	B2S2T6	RNA pseudouridylate synthase
HP TPASS_0348	6332968	B2S2U5	Heptaprenyl diphosphate synthase
HP TPASS_0352	6332965	B2S2U9	Transcriptional Coactivator p15
HP TPASS_0358	6333387	B2S2V5	Glycosyl hydrolase
HP TPASS_0369	6332954	B2S2W6	Tetratricopeptide repeat containing protein
HP TPASS_0371	6333425	B2S2W8	4-(cytidine 5'-diphospho)-2-C-methyl-D-erythritol kinase
HP TPASS_0373	6332951	B2S2X0	tRNA(Ile)-lysidine synthase
HP TPASS_0374	6333405	B2S2X1	Tetratricopeptide repeat containing protein
HP TPASS_0381	6333396	B2S2X8	Integral membrane protein
HP TPASS_0384	6333390	B2S2Y1	S-adenosyl-L-methionine-dependent methyltransferases
HP TPASS_0385	6332927	B2S2Y2	Cell division protein FtsL (Septum formation initiator)
HP TPASS_0392	6333379	B2S2Y9	Tetratricopeptide repeat containing protein
HP TPASS_0404	6333523	B2S301	Metallo-beta-lactamase
HP TPASS_0412	6332827	B2S309	PUR-alpha/beta/gamma DNA/RNA-binding protein
HP TPASS_0421	6333062	B2S318	Tetratricopeptide repeat containing protein
HP TPASS_0423	6333060	B2S320	P-loop containing nucleoside triphosphate hydrolases
HP TPASS_0431	6333579	B2S328	Pantothenate kinase, type III
HP TPASS_0436	6333546	B2S332	Phosphoesterase(DHH family)
HP TPASS_0438	6333091	B2S334	Non-canonical purine NTP pyrophosphatase
HP TPASS_0441	6333698	B2S337	Inorganic polyphosphate/ATP-NAD kinase
HP TPASS_0444	6333752	B2S340	Peptidoglycan binding(LysM domain),peptidase M23
HP TPASS_0447	6333695	B2S343	Tetratricopeptide repeat containing protein
HP TPASS_0449	6333086	B2S345	Tetratricopeptide repeat containing protein
HP TPASS_0458	6333084	B2S354	Chromosome segregation and condensation protein
HP TPASS_0459	6333689	B2S355	RNA pseudouridylate synthase
HP TPASS_0460	6333688	B2S356	Tetratricopeptide repeat containing protein
HP TPASS_0461	6333083	B2S357	DNA-binding helix-turn-helix protein(transcriptional regulator)
HP TPASS_0464	6333686	B2S360	tRNA (guanine-N(7)-)-methyltransferase
HP TPASS_0468	6333027	B2S364	Tetratricopeptide repeat containing protein
HP TPASS_0470	6333673	B2S365	Tetratricopeptide repeat containing protein
HP TPASS_0471	6333682	B2S366	Tetratricopeptide repeat containing protein
HP TPASS_0474	6333683	B2S369	DNA-binding regulatory protein(Trasncriptional regulator)
HP TPASS_0484	6333678	B2S379	FecR protein(regulation of iron dicitrate transport)
HP TPASS_0487	6333676	B2S382	Quinoprotein alcohol dehydrogenase
HP TPASS_0489	6333074	B2S384	Metallo-beta-lactamase
HP TPASS_0494	6332850	B2S389	Zinc ribbon domain containing protein
HP TPASS_0496	6333713	B2S390	Tetratricopeptide repeat containing protein
HP TPASS_0502	6333709	B2S396	Ankyrin repeat protein(protein binding)
HP TPASS_0512	6333669	B2S3A6	2-C-methyl-D-erythritol 2,4-cyclodiphosphate synthase
HP TPASS_0515	6333671	B2S3A9	Organic solvent tolerance protein
HP TPASS_0518	6333730	B2S3B2	Thiamin pyrophosphokinase
HP TPASS_0522	6332853	B2S3B5	Colicin V production protein
HP TPASS_0534	6333737	B2S3C6	V-type ATP synthase (subunit C)
HP TPASS_0544	6332856	B2S3D6	Endonuclease/Exonuclease/phosphatase
HP TPASS_0548	6333092	B2S3E0	Tetratricopeptide repeat containing protein
HP TPASS_0558	6333675	B2S3F0	Nickel/cobalt transporter, high-affinity
HP TPASS_0561	6332860	B2S3F3	TPM family (TLP18.3, Psb32 and MOLO-1 founding proteins of phosphatase)
HP TPASS_0563	6333116	B2S3F5	DnaJ domain-containing protein(molecular chaperon)
HP TPASS_0565	6333702	B2S3F7	SGNH hydrolase
HP TPASS_0567	6333260	B2S3F9	MgtE N-terminal domain containing protein(flagellar protein)
HP TPASS_0572	6332874	B2S3G2	TPM family(TLP18.3, Psb32 and MOLO-1 founding proteins of phosphatase)
HP TPASS_0580	6333280	B2S3G4	FMN-binding domain(Ferric reductase)
HP TPASS_0582	6333797	B2S3H1	Permease FtsX-like
HP TPASS_0588	6333269	B2S3H3	Permease FtsX-like
HP TPASS_0592	6332921	B2S3H9	DNA-directed DNA polymerase III delta subunit
HP TPASS_0599	6333138	B2S3I3	D-alanyl-D-alanine carboxypeptidase
HP TPASS_0608	6333867	B2S3I9	Zinc finger domain containing protein(DNA binding)
HP TPASS_0612	6333155	B2S3J8	ARM repeat containing protein(intracellular signalling and cytoskeletal regulation)
HP TPASS_0613	6333336	B2S3K2	Fe-S cluster assembly protein SufB
HP TPASS_0622	6332909	B2S3K3	Fe-S cluster assembly protein SufB
HP TPASS_0624	6332843	B2S3L2	Tetratricopeptide repeat containing protein
HP TPASS_0625	6333262	B2S3L4	Outer membrane protein, OmpA
HP TPASS_0636	6332923	B2S3L5	Tetratricopeptide repeat containing protein
HP TPASS_0648	6333773	B2S3M5	DNA repair protein RecO(Recombination)
HP TPASS_0651	6333776	B2S3N7	Tetratricopeptide repeat containing protein
HP TPASS_0674	6332788	B2S3P0	Metal-dependent phosphohydrolase, (7TM intracellular domain)
HP TPASS_0675	6333351	B2S3R3	Smr domain containing protein(DNA mismatch repair)
HP TPASS_0691	6332917	B2S3R4	Pheromone shutdown, TraB
HP TPASS_0702	6333746	B2S3T0	Prokaryotic chromosome segregation/condensation protein ScpA
HP TPASS_0699	6333718	B2S3T8	Transcriptional regulator, MerR family
HP TPASS_0706	6333365	B2S3U1	Peptidase M23
HP TPASS_0710	6333662	B2S3U5	Peptidase M23
HP TPASS_0719	6333069	B2S3U9	Jag_N family protein
HP TPASS_0730	6333796	B2S3V8	Flagellar biosynthesis protein, FliO
HP TPASS_0731	6333510	B2S3W9	CDP-diacylglycerol—glycerol-3-phosphate 3-phosphatidyltransferase
HP TPASS_0733	6333500	B2S3X0	NUDIX hydrolase
HP TPASS_0738	6333488	B2S3X2	Outer membrane protein (beta-barrel domain)
HP TPASS_0739	6333471	B2S3X7	Iojap/ribosomal silencing factor
HP TPASS_0740	6333287	B2S3X8	Cell envelope-related transcriptional attenuator
HP TPASS_0741	6332971	B2S3X9	Metal dependent phosphohydrolases with conserved 'HD' moti
HP TPASS_0750	6333824	B2S3Y0	nicotinate-nucleotide adenylyltransferase
HP TPASS_0752	6333218	B2S3Y9	von Willebrand factor, type A(adhesive plasma glycoprotein)
HP TPASS_0764	6333596	B2S3Z1	Sporulation and Cell division repeat protein
HP TPASS_0771	6333854	B2S403	Metal dependent phosphohydrolases with conserved 'HD' motif
HP TPASS_0776	6333834	B2S410	Sodium-dependent phosphate transport protein
HP TPASS_0777	6333848	B2S415	Phosphoribosyltransferases
HP TPASS_0782	6333666	B2S416	Zinc finger protein(DNA binding)
HP TPASS_0784	6333829	B2S421	Peptidase M23
HP TPASS_0785	6332804	B2S423	Lipopolysaccharide assembly, LptC
HP TPASS_0796	6333271	B2S424	Organic solvent tolerance(N terminal)
HP TPASS_0803	6333380	B2S435	Thiamine biosynthesis lipoprotein ApbE
HP TPASS_0815	6333371	B2S442	Phosphoesterase(DHH family)
HP TPASS_0820	6332940	B2S453	Acyl-CoA N-acyltransferase
HP TPASS_0822	6332941	B2S458	Tetratricopeptide repeat containing protein
HP TPASS_0826	6332943	B2S460	Mechanosensitive ion channel
HP TPASS_0832	6333406	B2S464	DisA bacterial checkpoint controller nucleotide-binding
HP TPASS_0840	6333412	B2S470	Sporulation and spore germination
HP TPASS_0846	6333419	B2S478	Major facilitator superfamily domain, general substrate transporter
HP TPASS_0851	6333426	B2S484	Cell division protein ZapA
HP TPASS_0854	6333428	B2S489	UDP-3-O-acylglucosamine N-acyltransferase
HP TPASS_0860	6332961	B2S492	HAMP domain-containing protein(regulation of phosphorylation or methylation of homodimeric receptors)
HP TPASS_0864	6332962	B2S498	Tetratricopeptide repeat containing protein
HP TPASS_0875	6333650	B2S4A2	Peptidase family M23 (LysM domain)
HP TPASS_0876	6333657	B2S4B2	ATP-binding protein
HP TPASS_0877	6333357	B2S4B3	Glycoprotease
HP TPASS_0879	6333063	B2S4B4	Metal dependent phosphohydrolases with conserved 'HD' motif
HP TPASS_0882	6332835	B2S4B6	ABC transporter
HP TPASS_0883	6333652	B2S4B9	ARM repeat containing protein(intracellular signalling and cytoskeletal regulation)
HP TPASS_0884	6333655	B2S4C0	Permease YjgP/YjgQ
HP TPASS_0893	6333646	B2S4C1	Permease YjgP/YjgQ
HP TPASS_0894	6333059	B2S4D0	Ribosome maturation factor RimP
HP TPASS_0899	6333640	B2S4D1	NYN domain, limkain-b1-type
HP TPASS_0900	6333058	B2S4D6	PD-(D/E)XK nuclease
HP TPASS_0901	6333638	B2S4D7	PD-(D/E)XK nuclease
HP TPASS_0906	6333635	B2S4D8	Multi antimicrobial extrusion protein
HP TPASS_0907	6333636	B2S4E3	KH domain containing protein (RNA binding)
HP TPASS_0911	6333054	B2S4E4	Ribosome maturation factor RimM
HP TPASS_0912	6333634	B2S4E8	Flagellar biosynthetic protein flhb
HP TPASS_0913	6332830	B2S4E9	Metal dependent phosphohydrolases with conserved 'HD' motif
HP TPASS_0915	6333052	B2S4F0	Restriction endonuclease, type II
HP TPASS_0920	6333051	B2S4F2	Tetratricopeptide repeat containing protein
HP TPASS_0923	6333048	B2S4F7	Tetratricopeptide repeat containing protein
HP TPASS_0931	6333617	B2S4G0	PEGA domain-containing protein
HP TPASS_0932	6333619	B2S4G8	Alpha-alpha trehalase
HP TPASS_0937	6333609	B2S4H4	Calcineurin-like phosphoesterase
HP TPASS_0942	6333611	B2S4H9	flagellar protein(FlgN)
HP TPASS_0944	6333040	B2S4I1	Tetratricopeptide repeat containing protein
HP TPASS_0954	6332869	B2S4J1	Tetratricopeptide repeat containing protein
HP TPASS_0959	6332970	B2S4J6	Rod binding protein(flagellar protein)
HP TPASS_0962	6333182	B2S4J9	Permease FtsX-like(efflux ABC transporter)
HP TPASS_0963	6333217	B2S4K0	Macrolide export ATP-binding/permease protein
HP TPASS_0972	6333285	B2S4K9	Macrolide export ATP-binding/permease protein
HP TPASS_0975	6333284	B2S4L2	rRNA small subunit methyltransferase I
HP TPASS_0977	6333282	B2S4L4	NIF3 (NGG1p interacting factor 3)
HP TPASS_0979	6332912	B2S4L6	TatD related DNase
HP TPASS_0986	6333008	B2S4M3	Multidrug resistance efflux transporter EmrE
HP TPASS_0988	6333049	B2S4M5	Multiple antibiotic resistance (MarC)-related
HP TPASS_0990	6333035	B2S4M7	Tetratricopeptide repeat containing protein
HP TPASS_0994	6333256	B2S4N1	TatD related DNase
HP TPASS_1018	6333868	B2S4Q5	2',3'-cyclic-nucleotide 2'-phosphodiesterase
HP TPASS_1029	6333264	B2S4R6	RNA binding protein
HP TPASS_1032	6333263	B2S4R9	Transcription antitermination protein nusG
HP TPASS_1033	6333866	B2S4S0	Patatin-like phospholipase
HP TPASS_1034	6333278	B2S4S1	Sodium/calcium exchanger protein

## Results and Discussion

**Fig 1:**
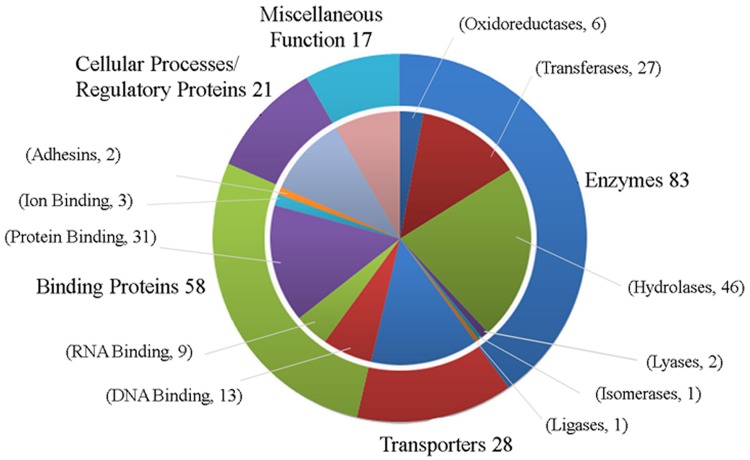
Classification of 207 HPs into various groups by utilizing the functional annotation results of various bioinformatics tools. The chart shows that there are 83 enzymes, 28 proteins involve in transportation, 58 binding proteins, 21 proteins involved in cellular processes like transcription, translation, replication etc. and 17 showing miscellaneous functions among 207 HPs from *T*. *pallidum ssp*. *pallidum*.

The genome of the SS14 strain was sequenced to high accuracy by Matejková et al., [2] in 2008 using oligonucleotide array strategy. But errors in key features such as start codons (alternate or otherwise) and stop codons (due to sequencing errors) were observed. Recently, the complete genome sequence of the TPA Mexico, A strain was reported by Pětrošová et al., [28] using the Illumina sequencing technique. However, a recent report on resequencing of *T*. *pallidum ssp*. *pallidum* strains Nichols and SS14 has identified errors in 11.5% of all annotated genes and subsequently corrected [29]. Hence, we assume that the available genome sequence of *T*. *pallidum ssp*. *pallidum* in the database is free from experimental sequencing errors. Extensive sequence analysis of all 444 HPs based on the above mentioned tools helped us to precisely assign function to 207 HPs with high confidence (Table 1). We have also predicted functions for 237 HPs with low level of confidence (S9 Table). We annotated the function of these HPs using protein classification databases such as CATH, Superfamily, Pfam, PANTHER, SYSTERS. Recent studies pertaining to experimental analysis of *T*. *pallidum ssp*. *pallidum genome* (Nichols) have provided us with solid evidences that support most of the predictions of this work [30]. All of these studies are performed using Nichols strain which shows slight variations from SS14 strain of *T*. *pallidum ssp*. *pallidum* [2]. Besides slight variations in some regions, we have found substantive correlation with data provided by these studies with that of predicted function in the present work. We categorized all these 207 HPs in various functional classes that contain 83 enzymes, 58 binding proteins, 28 transporters, 31 proteins involved in various cellular processes like regulation mechanisms, and 17 proteins exhibiting miscellaneous functions (Fig 1). Various functional classes of these classified HPs are described below.

### Enzymes

Enzymes play vital role in many leading biochemical processes. About 40% of annotated HPs are enzymes. *T*. *pallidum ssp*. *pallidum* is an obligate parasite therefore it solely depends on the host for most of its nutritional requirements [4]. Enzymes may facilitate its survival in the host by carrying out various cellular processes making it viable for the course of infection in the host.

We found six oxidoreductases among these HPs of *T*. *pallidum ssp*. *pallidum*. These enzymes presumably play an essential role in the pathogenesis. B2S298 (HP TPASS_0151) is NADH-quinone reductase (NQR2/RnfD) which regulates expression of virulence factors in *Vibrio cholerae* [31]. It is also involved in sodium translocation and electron transport [31]. Most of the oxidoreductases are involved in iron-sulphur cluster transport [31].

There are 27 HPs predicted as transferases. Many members of this class are involved in lipid biosynthesis, RNA processes and other significant cellular processes thus responsible for bacterial pathogenesis and virulence. There are various kinases such as B2S2P4 (HP TPASS_0296), which take part in coenzyme A biosynthesis [32]. B2S1Z8 (HP TPASS_0050) is predicted to be phosphoribosyl transferase. Members of PRTase family are involved in DNA processing and nucleotide metabolism [33]. Titz et al., [30] provided a similar function for the TP0050 gene product in Nichols strain of *T*. *pallidum ssp*. *pallidum* in their study which shows a significant similarity with HP TPASS_0050. B2S2Q5 (HP TPASS_0307) is a PASTA domain containing protein which is found in penicillin binding proteins and serine/threonine kinases [34]. McKevitt et al., [35] in their study of *T*. *pallidum ssp*. *pallidum* (Nichols strain) antigens predicted TP0307 as conserved hypothetical protein. This domain has special affinity for β-lactam antibiotics [34]. They characterized TP0750, TP0494 as conserved HPs [35]. In the present work, we have successfully assigned functions to their homologues in SS14 strain i.e. HP TPASS_0750 (B2S3Y0) and HP TPASS_0494 (B2S389) as nicotinate-nucleotide adenylyltransferase and zinc ribbon domain containing protein, respectively. B2S389 (HP TPASS_0494) and B2S3H9 (HP TPASS_0592) exhibit DNA directed polymerase activity, hence proving their role in bacterial pathogenesis by facilitating regulatory processes. B2S492 (HP TPASS_0860) is HAMP domain containing protein which is a characteristic domain of signal transduction proteins and helps in signal conversion [36].

The third class of enzymes is hydrolases. There are more than 50% proteins in all characterized enzymes representing this class of enzymes. The majority of representative proteins of hydrolase class are membrane bound proteins involved in various significant processes such transmembrane transport, metal ion binding, cell wall degradation, thus associated with various virulence factors. There is a number proteins having peptidase activity that contains LysM domain, responsible for cell wall degradation in prokaryotes [37] which helps various transmembrane transporters to carry out their functions. There are six phosphohydrolases in this group. They contain conserved HD motif which holds the specific characteristic of signal transduction systems [38] and have metal ion binding property [39]. We found B2S4K0 (HP TPASS_0963) and B2S4K9 (HP TPASS_0972) which exhibit antibiotic resistance capacity and are involved in macrolide antibiotic transportation [40]. Titz et al., [30] predicted TP0936, a counterpart of HP TPASS_0963 in the Nichols strain as ABC transporter and depicted its involvement in membrane biogenesis. We predicted HP TPASS_0444 (B2S340) as peptidoglycan-binding protein. Homologue of HP TPASS_0444 in the Nichols strain (TP0444) is predicted as conserved HP in the above mentioned study. We have successfully assigned function to the homologue of TP0877 in SS14 strain (HP TPASS_0877) as glycoprotease which is characterized as conserved HP in the gene expression analysis as done by Smajs et al., [41].

Lyases also play a key role in bacterial pathogenesis as they are involved in various biosynthesis processes. B2S3A6 (HP TPASS_0512) shows 2-C-methyl-D-erythritol 2, 4-cyclodiphosphate synthase activity and is involved in isoprenoid synthesis. It may be acting as a potential drug target [42].

### Transporters

Transporter proteins are involved in transportation of nutrients, that are helpful in various metabolic processes, and hence survival of the organism. These proteins also facilitate the transfer of virulence factors and are directly involved in infection [43]. We found 28 proteins having functions as transporters possibly involved in transportation of metal ions, virulence factors and biosynthesis assembly proteins. Some of HPs are the members of ABC transporter class proteins. B2S3C6 (HP TPASS_0534) is V-type ATP synthase (subunit C) which may be involved in ATP synthesis hence may be involved in providing energy for various metabolic processes of bacterial pathogen [44]. B2S3F9 (HP TPASS_0567) is MgtE N-terminal domain containing protein and helps in magnesium transport [45]. McKevitt et al and Smajs et al characterized its counterpart (TP0567) as HPs in their experimental studies [35,41]. Similarly, B2S3G4 (HP TPASS_0580) is FMN-binding domain protein which is found to be involved in the electron transfer pathway [46]. Titz et al., [30] predicted the gene product of Nichols strain (TP0580) as ABC transporter whereas Smajs et al., [41] characterized it as conserved hypothetical integral membrane protein. B2S3L4 (HP TPASS_0625) is an outer membrane protein (OmpA) which works as a receptor for T-even like phages. It also acts as a porin protein with low permeability allowing penetration of small solutes [47]. B2S460 (HP TPASS_0826) is predicted as mechanosensitive ion channel which allows efflux of solvent and solutes in cytoplasm hence making its role significant in survival of pathogen [48]. B2S478 (HP TPASS_0846) contains major facilitator superfamily domain and is a representative of a class of membrane transporters which are involved in transportation of sugars, amino acids, drugs, various metabolites and varieties of ions [49]. B2S4D8 (HP TPASS_0906) and B2S4M3 (HP TPASS_0986) are multidrug transporters and exhibit multiple drug resistance capability thus making the pathogen viable against drugs [50]. A detailed understanding of the functional mechanism of all these transporters will be helping to discover effective drugs against them.

### Binding proteins

We have characterized 58 proteins as binding proteins out of 207 functionally annotated HPs. We have further divided these into 13 DNA binding, nine RNA binding, 31 protein binding, three ion binding and two adhesion proteins. The DNA and RNA binding proteins are involved in various cellular and regulatory processes such as transcription, translation and recombination and thus playing a vital role in the survival and propagation of pathogen in the host. 31 HPs are the protein binding in nature, and 29 of them are tetratricopeptide repeat (TPR) containing proteins. TPR containing proteins are involved in protein-protein interactions and thus plays an important role in virulence [51]. B2S214 (HP TPASS_0066) and B2S215 (HP TPASS_0067) are tetratricopeptide repeat containing proteins. Titz et al., [30] predicted their homologues in Nichols strain (TP0066 and TP0067) to be involved in DNA metabolism. Tetratricopeptide repeat containing proteins are involved in various metabolic and regulatory processes [51]. Homologues of this protein predicted with tetrapeptide repeats in the present work are characterized as HP by McKevitt and Smajs group [35,41]. Therefore, proteins showing 100% similarity may be considered exhibiting similar functions for Nichols strain and indicating experimental evidence. We found that B2S2J3 (HP TPASS_0246) and B2S3Y9 (HP TPASS_0752) are showing similarity with von Willebrand factor with a type A domain which is found to be responsible for various blood disorders [52–54]. Association of type A domain makes it liable to be involved in various significant activities such as cell adhesion and immune defense [55]. Thus, such HPs may be possible therapeutic targets because they are involved in the bacterial pathogenesis by helping in cell adhesion and immune defense mechanism.

### Cellular processes/regulatory proteins

There are 21 HPs presumably involved in various cellular and regulatory mechanisms, and are important for the pathogenesis of *T*. *pallidum ssp*. *pallidum*. Most of these proteins are involved in cell division, chromosome segregation and condensation, sporulation, intercellular signaling and various flagellar proteins involved in transport activity. These proteins may also be important for bacterial pathogenesis and can be treated as possible drug targets [56]. B2S2P5 (HP TPASS_0297) is found to be presumably involved in sporulation and cell division. Titz et al., [30] predicted involvement of its counterpart TP0297 (Nichols strain) in the cell wall metabolism. B2S3T0 (HP TPASS_0702) is prokaryotic chromosome segregation/condensation protein ScpA whereas its homologue in Nichols strain (TP0702) was characterized as a HP in the study done by Smajs et al on *T*. *pallidum ssp*. *pallidum* transcriptome [41].

### Proteins with miscellaneous functions

We found 17 HPs exhibiting miscellaneous functions such as cell signaling, solvent tolerance proteins, etc. B2S234 (HP TPASS_0086) is a PilZ domain containing protein that serves as the receptor for cyclic di-GMP which act as secondary messenger for bacteria [57,58]. Cyclic di-GMP is involved in regulation of exo-polysaccharide synthesis, motility of bacteria, gene expression and host-pathogen interaction [57,58]. Hence, these HPs may also be considered to be significant in the pathogenesis of *T*. *pallidum ssp*. *pallidum*. B2S3A9 (HP TPASS_0515) and B2S424 (HP TPASS_0796) are organic solvent tolerance proteins responsible for antibiotic resistance [59]. Smajs et al., [41] characterized its homologue in the Nichols strain (TP0796) as conserved HP. B2S3B5 (HP TPASS_0522) is a colicin V production protein that is a bacterial toxin which disrupts the membrane potential of other sensitive cell thus leading to their death [60]. B2S3F5 (HP TPASS_0563) is a DnaJ domain containing protein which is an exclusive feature of hsp40 family of molecular chaperons [61]. These molecular chaperons are involved in various significant processes such as protein folding, polypeptide translocation and protein degradation [61]. Our knowledge of these HPs will be helpful in the field of the drug discovery by completing the mosaic of knowledge regarding the host-pathogen interaction especially in the case of *T*. *pallidum ssp*. *pallidum*.

We compared the group of HPs successfully annotated with high confidence (Table 1) with those of unannotated genes (Table S9). For the comparison, we considered several characteristics features such as average gene length, the number of predicted protein- protein interactions, gene expression level and predicted antigens. Surprisingly, there is a relative difference between average gene lengths of the HPs of both groups was observed. The average length of polypeptides chain, not annotated, are less than 40 amino acids, which corresponding to the gene length of 120 bps. Whereas, in the group of HPs predicted with a high level of confidence (n = 207) the average gene length is relatively high. We can infer that the relatively smaller gene lengths have affected the confidence level of this group.

We further used STRING [27] to predict the protein-protein interactions. While comparing both groups for the number of predicted protein-protein interactions, we found no such characteristic difference that could affect the confidence level of function prediction. For instance, string predicted 10 functional partners for the protein HP TPASS_0017 (B2S1W5) whereas it predicted 4 functional partners for the protein HP TPASS_0004 (B2S1V4) which is an HP of the group for which functions are assigned with low level of confidence. It predicted only two functional partners for the HP TPASS_0022 (B2S1X0) which is from first group whereas it predicted 10 functional partners for the HP TPASS_0008 (B2S1V7) which is an HP from second group.

We checked the expression level of genes from both groups on the basis of study of Smajs et al. [41]. We did not find any such correlations for the gene expression levels in this study. On the other hand, we checked the number of predicted antigens using the investigation of McKevitt et al. [35] for *T*. *pallidum* antigens. We found 17 predicted antigens in the group of HPs for which functions are predicted with a high-level of confidence. Whereas, against the expectations, we found a relatively higher number of predicted antigens i.e., 24 in the second group. The comparison done between both the groups considering characteristics such as gene length, predicted protein—protein interactions, gene expression levels and predicted antigens established no characteristic difference except for the gene length that is relatively low in the second group (n = 237). We should notice, although, that no differences between the group of genes with predicted function and the group of genes with a less accurate predicted function is here observed if we compare these results with previously published experimental studies [35,41]. This may suggest that the degree of prediction accuracy does not necessarily allow to univocally identify functional genes and has to be taken with caution.

### Virulent proteins

Gram negative pathogens are frequently evolved to modify the features like increase motility, cell adhesion and to tackle with immune response of the host, thus increasing their virulence inside the host environment [62]. We have used VICMpred and Virulentpred servers to predict virulence factors in this group of 444 HPs. There are 19 HPs (out of 207) found to be virulent on the basis of the consensus sequence analysis (Table 2). It was already hypothesized that targeting virulence factor provides a better therapeutic intervention against bacterial pathogenesis [63]. The predicted HPs having virulent characteristics provide a powerful target-based therapies to clear an existing infection and are further considered as an adjunct therapy to existing antibiotics, or potentiators of the host immune response [64]. The progress reported recently a proof of concept for antivirulence molecules at the preclinical stages should allow the antivirulence concept to become a reality as a new antibacterial approach.

**Table 2:** List of HPs with virulence factors in *T*. *pallidum ssp*. *pallidum*.

Protein Name	Uniprot ID	VICMPred tool	Virulentpred tool
HP TPASS_0022	B2S1X0	Virulent	Virulent
HP TPASS_0304	B2S2Q2	Virulent	Virulent
HP TPASS_0444	B2S340	Virulent	Non Virulent
HP TPASS_0474	B2S369	Virulent	Non Virulent
HP TPASS_0484	B2S379	Virulent	Virulent
HP TPASS_0512	B2S3A6	Virulent	Non Virulent
HP TPASS_0515	B2S3A9	Virulent	Virulent
HP TPASS_0534	B2S3C6	Virulent	Virulent
HP TPASS_0612	B2S3K2	Virulent	Non Virulent
HP TPASS_0622	B2S3L2	Virulent	Virulent
HP TPASS_0675	B2S3R4	Virulent	Virulent
HP TPASS_0706	B2S3U5	Virulent	Non Virulent
HP TPASS_0710	B2S3U9	Virulent	Non Virulent
HP TPASS_0782	B2S421	Virulent	Non Virulent
HP TPASS_0796	B2S435	Virulent	Non Virulent
HP TPASS_0851	B2S489	Virulent	Virulent
HP TPASS_0864	B2S4A2	Virulent	Non Virulent
HP TPASS_0893	B2S4D0	Virulent	Virulent
HP TPASS_0900	B2S4D7	Virulent	Virulent
HP TPASS_0911	B2S4E8	Virulent	Virulent

## Conclusions

Functional annotation of 444 HPs from *T*. *pallidum ssp*. *pallidum* has been carried out using various *in silico* approaches and functions have been assigned to 207 HPs with high confidence. Performance assessment of bioinformatics tools was carried out using ROC analysis and reported in terms of accuracy and sensitivity of the predicting tools. We are not considering the HPs annotated with low level of confidence. Our prediction is showing functional importance of the HPs in the survival of the pathogen in the host. Our study facilitates a rapid identification of the hidden function of HPs which is potential therapeutic targets and may play a significant role in better understanding of host-pathogen interactions. Once these HPs are established as a novel drug/vaccine targets, further research for new inhibitors and vaccines can be conducted.

## Supporting Information

S1 Table List of computed physicochemical properties of 444 HPs from *T*. *pallidum ssp*. *pallidum*. (DOC)

S2 Table List of predicted subcellular localizations of 444 HPs from *T*. *pallidum ssp*. *pallidum*. (DOC)

S3 Table List of predicted results of Blast, STRING, HMMER, SMART and INTERPROSCAN for 444 HPs from *T*. *pallidum ssp*. *pallidum*. (DOC)

S4 Table List of predicted results of CATH, SUPERFAMILY, PANTHER, CDART, Pfam, SYSTERS and ProtoNet for 444 HPs from *T*. *pallidum ssp*. *pallidum*. (DOC)

S5 Table List of predicted virulence factors from 444 HPs from *T*. *pallidum ssp*. *pallidum* by using VICMPred and Virulentpred. (DOC)

S6 Table List of annotated function of 100 proteins with known function from *T*. *pallidum ssp*. *pallidum* using BLASTp, HMMER, SMART and INTERPROSCAN for ROC analysis. (DOC)

S7 Table List of functionally annotated domain of 100 proteins with known function from *T*. *pallidum ssp*. *pallidum* by CATH, SUPERFAMILY, PANTHER, CDART, Pfam, SYSTERS, and ProtoNet for ROC analysis. (DOC)

S8 Table List of accuracy, sensitivity, specificity and ROC area of various bioinformatics. (DOC)

S9 Table Functionally annotated HPs from *T*. *pallidum ssp*. *pallidum* with low level of confidence. (DOCX)

## References

[1] McGill MA, Edmondson DG, Carroll JA, Cook RG, Orkiszewski RS, Norris SJ (2010) Characterization and serologic analysis of the Treponema pallidum proteome. Infection and immunity 78: 2631–2643. 10.1128/IAI.00173-10 20385758 PMC2876534

[2] Matejkova P, Strouhal M, Smajs D, Norris SJ, Palzkill T, Petrosino JF, et al (2008) Complete genome sequence of Treponema pallidum ssp. pallidum strain SS14 determined with oligonucleotide arrays. BMC Microbiol 8: 76 10.1186/1471-2180-8-76 18482458 PMC2408589

[3] Fraser CM, Norris SJ, Weinstock GM, White O, Sutton GG, Dodson R, et al (1998) Complete genome sequence of Treponema pallidum, the syphilis spirochete. Science 281: 375–388. 9665876 10.1126/science.281.5375.375

[4] Peeling RW, Hook EW 3rd (2006) The pathogenesis of syphilis: the Great Mimicker, revisited. The Journal of pathology 208: 224–232. 16362988 10.1002/path.1903

[5] Paster BJ, Dewhirst FE (2000) Phylogenetic foundation of spirochetes. J Mol Microbiol Biotechnol 2: 341–344. 11075904

[6] Hertel M, Matter D, Schmidt-Westhausen AM, Bornstein MM (2014) Oral syphilis: a series of 5 cases. J Oral Maxillofac Surg 72: 338–345. 10.1016/j.joms.2013.07.015 24045192

[7] Singh AE, Romanowski B (1999) Syphilis: review with emphasis on clinical, epidemiologic, and some biologic features. Clin Microbiol Rev 12: 187–209. 10194456 10.1128/cmr.12.2.187 PMC88914

[8] Abell E, Marks R, Jones EW (1975) Secondary syphilis: a clinico-pathological review. Br J Dermatol 93: 53–61. 1191529 10.1111/j.1365-2133.1975.tb06476.x

[9] Stamm LV, Kerner TC Jr., Bankaitis VA, Bassford PJ Jr. (1983) Identification and preliminary characterization of Treponema pallidum protein antigens expressed in Escherichia coli. Infection and immunity 41: 709–721. 6347894 10.1128/iai.41.2.709-721.1983 PMC264700

[10] Desler C, Suravajhala P, Sanderhoff M, Rasmussen M, Rasmussen L (2009) In Silico screening for functional candidates amongst hypothetical proteins. BMC Bioinformatics 10: 289 10.1186/1471-2105-10-289 19754976 PMC2758874

[11] Kumar K, Prakash A, Tasleem M, Islam A, Ahmad F, Hassan MI (2014) Functional annotation of putative hypothetical proteins from Candida dubliniensis. Gene 543: 93–100. 10.1016/j.gene.2014.03.060 24704023

[12] Shahbaaz M, Ahmad F, Imtaiyaz Hassan M (2014) Structure-based functional annotation of putative conserved proteins having lyase activity from Haemophilus influenzae. 3 Biotech: 1–20. 10.1007/s13205-014-0231-z PMC4434415 28324295

[13] Kumar K, Prakash A, Islam A, Ahmad F, Hassan MI (2014) Structure based Functional Annotation of Hypothetical Proteins from Candida dubliniensis: A Quest for Novel Drug Target. 3Biotech (In Press). 10.1007/s13205-014-0256-3 PMC4522726 28324558

[14] Sinha A, Ahmad F, Hassan MI (2014) Structure Based Functional Annotation of Putative Conserved Proteins from Treponema pallidum: Search for a Potential Drug Target. Letters in Drug Design & Discovery 12: 46–59.

[15] Shahbaaz M, Hassan MI, Ahmad F (2013) Functional annotation of conserved hypothetical proteins from Haemophilus influenzae Rd KW20. PloS one 8: e84263 10.1371/journal.pone.0084263 24391926 PMC3877243

[16] Hassan MI, Kumar V, Singh TP, Yadav S (2007) Structural model of human PSA: a target for prostate cancer therapy. Chem Biol Drug Des 70: 261–267. 17718721 10.1111/j.1747-0285.2007.00553.x

[17] Hassan MI, Kumar V, Somvanshi RK, Dey S, Singh TP, Yadav S (2007) Structure-guided design of peptidic ligand for human prostate specific antigen. J Pept Sci 13: 849–855. 17890654 10.1002/psc.911

[18] Thakur P, Kumar J, Ray D, Anjum F, Hassan MI (2013) Search of potential inhibitor against New Delhi metallo-beta-lactamase 1 from a series of antibacterial natural compounds using docking approach. J Nat Sci Biol Med. 10.4103/0976-9668.107260 PMC3633303 23633835

[19] Thakur PK, Hassan I (2011) Discovering a potent small molecule inhibitor for gankyrin using de novo drug design approach. Int J Comput Biol Drug Des 4: 373–386. 10.1504/IJCBDD.2011.044404 22199037

[20] Thakur PK, Prakash A, Khan P, Fleming RE, Waheed A, Ahmad F, et al (2013) Identification of Interfacial Residues Involved in Hepcidin-Ferroportin Interaction. Lett Drug Des Discov 11: 363–374.

[21] Sinha A, Ahmad F, Hassan MI (2014) Structure Based Functional Annotation of Putative Conserved Proteins from Treponema pallidum: Search for a Potential Drug Target. Letters in Drug Design & Discovery (In Press).

[22] Mazandu GK, Mulder NJ (2012) Function Prediction and Analysis of Mycobacterium tuberculosis Hypothetical Proteins. Int J Mol Sci 13: 7283–7302. 10.3390/ijms13067283 22837694 PMC3397526

[23] Vetrivel U, Subramanian G, Dorairaj S (2011) A novel in silico approach to identify potential therapeutic targets in human bacterial pathogens. Hugo J 5: 25–34. 10.1007/s11568-011-9152-7 23205162 PMC3238024

[24] Zheng LL, Li YX, Ding J, Guo XK, Feng KY, Wang YJ, et al (2012) A comparison of computational methods for identifying virulence factors. PLoS One 7: e42517 10.1371/journal.pone.0042517 22880014 PMC3411817

[25] Saha S, Raghava GP (2006) VICMpred: an SVM-based method for the prediction of functional proteins of Gram-negative bacteria using amino acid patterns and composition. Genomics Proteomics Bioinformatics 4: 42–47. 16689701 10.1016/S1672-0229(06)60015-6 PMC5054027

[26] Garg A, Gupta D (2008) VirulentPred: a SVM based prediction method for virulent proteins in bacterial pathogens. BMC Bioinformatics 9: 62 10.1186/1471-2105-9-62 18226234 PMC2254373

[27] Franceschini A, Szklarczyk D, Frankild S, Kuhn M, Simonovic M, Roth A, et al (2013) STRING v9.1: protein-protein interaction networks, with increased coverage and integration. Nucleic Acids Res 41: D808–815. 10.1093/nar/gks1094 23203871 PMC3531103

[28] Petrosova H, Zobanikova M, Cejkova D, Mikalova L, Pospisilova P, Strouhal M, et al (2012) Whole genome sequence of Treponema pallidum ssp. pallidum, strain Mexico A, suggests recombination between yaws and syphilis strains. PLoS Negl Trop Dis 6: e1832 10.1371/journal.pntd.0001832 23029591 PMC3447947

[29] Petrosova H, Pospisilova P, Strouhal M, Cejkova D, Zobanikova M, Mikalova L, et al (2013) Resequencing of Treponema pallidum ssp. pallidum strains Nichols and SS14: correction of sequencing errors resulted in increased separation of syphilis treponeme subclusters. PloS one 8: e74319 10.1371/journal.pone.0074319 24058545 PMC3769245

[30] Titz B, Rajagopala SV, Goll J, Hauser R, McKevitt MT, Palzkill T, et al (2008) The binary protein interactome of Treponema pallidum—the syphilis spirochete. PLoS One 3: e2292 10.1371/journal.pone.0002292 18509523 PMC2386257

[31] Hase CC, Mekalanos JJ (1999) Effects of changes in membrane sodium flux on virulence gene expression in Vibrio cholerae. Proceedings of the National Academy of Sciences of the United States of America 96: 3183–3187. 10077658 10.1073/pnas.96.6.3183 PMC15916

[32] Obmolova G, Teplyakov A, Bonander N, Eisenstein E, Howard AJ, Gilliland GL (2001) Crystal structure of dephospho-coenzyme A kinase from Haemophilus influenzae. Journal of structural biology 136: 119–125. 11886213 10.1006/jsbi.2001.4428

[33] Anantharaman V, Iyer LM, Aravind L (2012) Ter-dependent stress response systems: novel pathways related to metal sensing, production of a nucleoside-like metabolite, and DNA-processing. Molecular bioSystems 8: 3142–3165. 10.1039/c2mb25239b 23044854 PMC4104200

[34] Yeats C, Finn RD, Bateman A (2002) The PASTA domain: a beta-lactam-binding domain. Trends in biochemical sciences 27: 438 12217513 10.1016/s0968-0004(02)02164-3

[35] McKevitt M, Brinkman MB, McLoughlin M, Perez C, Howell JK, Weinstock GM, et al (2005) Genome scale identification of Treponema pallidum antigens. Infection and immunity 73: 4445–4450. 15972547 10.1128/IAI.73.7.4445-4450.2005 PMC1168556

[36] Stewart V (2014) The HAMP signal-conversion domain: static two-state or dynamic three-state? Molecular microbiology 91: 853–857. 10.1111/mmi.12516 24417364

[37] Joris B, Englebert S, Chu CP, Kariyama R, Daneo-Moore L, Shockman GD, et al (1992) Modular design of the Enterococcus hirae muramidase-2 and Streptococcus faecalis autolysin. FEMS microbiology letters 70: 257–264. 1352512 10.1016/0378-1097(92)90707-u

[38] Galperin MY, Natale DA, Aravind L, Koonin EV (1999) A specialized version of the HD hydrolase domain implicated in signal transduction. Journal of molecular microbiology and biotechnology 1: 303–305. 10943560 PMC5330256

[39] Aravind L, Koonin EV (1998) The HD domain defines a new superfamily of metal-dependent phosphohydrolases. Trends in biochemical sciences 23: 469–472. 9868367 10.1016/s0968-0004(98)01293-6

[40] Lin HT, Bavro VN, Barrera NP, Frankish HM, Velamakanni S, van Veen HW, et al (2009) MacB ABC transporter is a dimer whose ATPase activity and macrolide-binding capacity are regulated by the membrane fusion protein MacA. J Biol Chem 284: 1145–1154. 10.1074/jbc.M806964200 18955484 PMC2613632

[41] Smajs D, McKevitt M, Howell JK, Norris SJ, Cai WW, Palzkill T, et al (2005) Transcriptome of Treponema pallidum: gene expression profile during experimental rabbit infection. Journal of bacteriology 187: 1866–1874. 15716460 10.1128/JB.187.5.1866-1874.2005 PMC1063989

[42] Kishida H, Wada T, Unzai S, Kuzuyama T, Takagi M, Terada T, et al (2003) Structure and catalytic mechanism of 2-C-methyl-D-erythritol 2,4-cyclodiphosphate (MECDP) synthase, an enzyme in the non-mevalonate pathway of isoprenoid synthesis. Acta crystallographica Section D, Biological crystallography 59: 23–31. 12499535 10.1107/s0907444902017705

[43] Franke K, Nguyen M, Hartl A, Dahse HM, Vogl G, Wurzner R, et al (2006) The vesicle transport protein Vac1p is required for virulence of Candida albicans. Microbiology 152: 3111–3121. 17005990 10.1099/mic.0.29115-0

[44] Rappas M, Niwa H, Zhang X (2004) Mechanisms of ATPases—a multi-disciplinary approach. Curr Protein Pept Sci 5: 89–105. 15078220 10.2174/1389203043486874

[45] Hattori M, Tanaka Y, Fukai S, Ishitani R, Nureki O (2007) Crystal structure of the MgtE Mg2+ transporter. Nature 448: 1072–1075. 17700703 10.1038/nature06093

[46] Liepinsh E, Kitamura M, Murakami T, Nakaya T, Otting G (1997) Pathway of chymotrypsin evolution suggested by the structure of the FMN-binding protein from Desulfovibrio vulgaris (Miyazaki F). Nature structural biology 4: 975–979. 9406543 10.1038/nsb1297-975

[47] MacIntyre S, Henning U (1990) The role of the mature part of secretory proteins in translocation across the plasma membrane and in regulation of their synthesis in Escherichia coli. Biochimie 72: 157–167. 1974149 10.1016/0300-9084(90)90141-3

[48] Naismith JH, Booth IR (2012) Bacterial mechanosensitive channels—MscS: evolution's solution to creating sensitivity in function. Annual review of biophysics 41: 157–177. 10.1146/annurev-biophys-101211-113227 22404681 PMC3378650

[49] Pao SS, Paulsen IT, Saier MH Jr. (1998) Major facilitator superfamily. Microbiology and molecular biology reviews: MMBR 62: 1–34. 9529885 10.1128/mmbr.62.1.1-34.1998 PMC98904

[50] Ninio S, Rotem D, Schuldiner S (2001) Functional analysis of novel multidrug transporters from human pathogens. The Journal of biological chemistry 276: 48250–48256. 11574548 10.1074/jbc.M108231200

[51] Cerveny L, Straskova A, Dankova V, Hartlova A, Ceckova M, Staud F, et al (2013) Tetratricopeptide repeat motifs in the world of bacterial pathogens: role in virulence mechanisms. Infection and immunity 81: 629–635. 10.1128/IAI.01035-12 23264049 PMC3584863

[52] Ruggeri ZM, Ware J (1993) von Willebrand factor. FASEB J 7: 308–316. 8440408 10.1096/fasebj.7.2.8440408

[53] Ahmad F, Jan R, Kannan M, Obser T, Hassan MI, Oyen F, et al (2013) Characterisation of mutations and molecular studies of type 2 von Willebrand disease. Thromb Haemost 109: 39–46. 10.1160/TH12-07-0475 23179108

[54] Hassan MI, Saxena A, Ahmad F (2012) Structure and function of von Willebrand factor. Blood Coagul Fibrinolysis 23: 11–22. 10.1097/MBC.0b013e32834cb35d 22089939

[55] Colombatti A, Bonaldo P, Doliana R (1993) Type A modules: interacting domains found in several non-fibrillar collagens and in other extracellular matrix proteins. Matrix 13: 297–306. 8412987 10.1016/s0934-8832(11)80025-9

[56] Singer HM, Kuhne C, Deditius JA, Hughes KT, Erhardt M (2014) The Salmonella Spi1 virulence regulatory protein HilD directly activates transcription of the flagellar master operon flhDC. J Bacteriol 196: 1448–1457. 10.1128/JB.01438-13 24488311 PMC3993337

[57] Amikam D, Galperin MY (2006) PilZ domain is part of the bacterial c-di-GMP binding protein. Bioinformatics 22: 3–6. 16249258 10.1093/bioinformatics/bti739

[58] Ryjenkov DA, Simm R, Romling U, Gomelsky M (2006) The PilZ domain is a receptor for the second messenger c-di-GMP: the PilZ domain protein YcgR controls motility in enterobacteria. J Biol Chem 281: 30310–30314. 16920715 10.1074/jbc.C600179200

[59] Pourahmad Jaktaji R, Ebadi R, Karimi M (2012) Study of Organic Solvent Tolerance and Increased Antibiotic Resistance Properties in E. coli gyrA Mutants. Iranian journal of pharmaceutical research: IJPR 11: 595–600. 24250484 PMC3832178

[60] Yang CC, Konisky J (1984) Colicin V-treated Escherichia coli does not generate membrane potential. J Bacteriol 158: 757–759. 6373733 10.1128/jb.158.2.757-759.1984 PMC215499

[61] Frydman J (2001) Folding of newly translated proteins in vivo: the role of molecular chaperones. Annu Rev Biochem 70: 603–647. 11395418 10.1146/annurev.biochem.70.1.603

[62] Livorsi DJ, Stenehjem E, Stephens DS (2011) Virulence factors of gram-negative bacteria in sepsis with a focus on Neisseria meningitidis. Contributions to microbiology 17: 31–47. 10.1159/000324008 21659746

[63] Clatworthy AE, Pierson E, Hung DT (2007) Targeting virulence: a new paradigm for antimicrobial therapy. Nat Chem Biol 3: 541–548. 17710100 10.1038/nchembio.2007.24

[64] Marra A (2006) Targeting virulence for antibacterial chemotherapy: identifying and characterising virulence factors for lead discovery. Drugs R D 7: 1–16. 16620133 10.2165/00126839-200607010-00001

