# Correction: Identification of Functional Candidates amongst Hypothetical Proteins of *Treponema pallidum ssp*. *pallidum*

**DOI:** 10.1371/journal.pone.0197452

**Published:** 2018-05-14

**Authors:** Ahmad Abu Turab Naqvi, Mohd Shahbaaz, Faizan Ahmad, Md. Imtaiyaz Hassan

The cells in Table 1 are misaligned, leading to the assignment of Functions to the incorrect Uniprot IDs, GeneIDs, and Protein names. Please see the corrected Table 1 here.

**Table 1:** Functionally annotated HPs from *T*. *pallidum ssp*. *pallidum*.

S. No	Protein name	GeneID	Uniprot ID	Function
1.	HP TPASS_0017	6333127	B2S1W5	Tetratricopeptide repeat containing protein
2.	HP TPASS_0022	6333189	B2S1X0	Helicase C terminal domain protein
3.	HP TPASS_0024	6333763	B2S1X2	Potassium ion(K+) transporter
4.	HP TPASS_0025	6332893	B2S1X3	Peptidase M16(Metalloenzyme)
5.	HP TPASS_0042	6333174	B2S1Z0	Peptidoglycan binding(LysM domain- bacterial cell wall degradation)
6.	HP TPASS_0046	6332886	B2S1Z4	PSP1 C-terminal(polymerase suppressor 1)
7.	HP TPASS_0048	6333172	B2S1Z6	Polymer forming cytoskeletal
8.	HP TPASS_0049	6332885	B2S1Z7	Peptidoglycan hydrolase(Peptidase M23)(LytM domain)
9.	HP TPASS_0050	6333168	B2S1Z8	Phosphoribosyl transferase(PRTase)
10.	HP TPASS_0054	6333745	B2S202	RNA 2’-O ribose methyltransferase(Substrate binding)
11.	HP TPASS_0055	6332884	B2S203	Oxaloacetate decarboxylase (gamma subunit)
12.	HP TPASS_0064	6332880	B2S212	Alpha-ketoacid dehydrogenase kinase(N terminal)
13.	HP TPASS_0065	6333159	B2S213	S-adenosyl-L-methionine-dependent methyltransferases
14.	HP TPASS_0066	6333156	B2S214	Tetratricopeptide repeat containing protein
15.	HP TPASS_0067	6332879	B2S215	Tetratricopeptide repeat containing protein
16.	HP TPASS_0068	6333820	B2S216	Ribosomal RNA large subunit methyltransferase N (Radical SAM enzyme)
17.	HP TPASS_0072	6333819	B2S220	Glutaredoxin
18.	HP TPASS_0073	6333821	B2S221	Metal dependent phosphohydrolases with conserved 'HD' motif
19.	HP TPASS_0079	6333203	B2S227	Xanthine dehydrogenase(Molibdoprotein binding)
20.	HP TPASS_0081	6332890	B2S229	Xanthine dehydrogenase(Molibdoprotein binding, FAD binding)
21.	HP TPASS_0083	6333811	B2S231	glycosyl hydrolase
22.	HP TPASS_0084	6333817	B2S232	Thioredoxin
23.	HP TPASS_0086	6333164	B2S234	PilZ domain containing protein(c-di-GMP binding)
24.	HP TPASS_0095	6332871	B2S243	Tetratricopeptide repeat containing protein
25.	HP TPASS_0121	6333137	B2S269	Lysine-2,3-aminomutase
26.	HP TPASS_0123	6332867	B2S271	Tetratricopeptide repeat containing protein
27.	HP TPASS_0126	6333787	B2S274	Outer membrane protein (beta-barrel domain)
28.	HP TPASS_0139	6333134	B2S287	Potassium ion(K+) transporter(NAD(P) binding)
29.	HP TPASS_0151	6332899	B2S298	NADH-quinone reductase(NQR2/RnfD)
30.	HP TPASS_0153	6333195	B2S2A0	Acid phosphatase/vanadium-dependent haloperoxidase
31.	HP TPASS_0154	6333194	B2S2A1	RNA pseudouridylate synthase
32.	HP TPASS_0156	6333198	B2S2A3	4-hydroxybenzoyl-CoA thioesterase
33.	HP TPASS_0157	6333190	B2S2A4	Glycerol-3-phosphate O-acyltransferase
34.	HP TPASS_0158	6333188	B2S2A5	Haloacid dehalogenase
35.	HP TPASS_0181	6333597	B2S2C8	Septum formation initiator
36.	HP TPASS_0182	6333036	B2S2C9	Telomere recombination(Sua5_yciO_yrdC family)
37.	HP TPASS_0223	6333564	B2S2H0	Aspartate aminotransferase (EC 2.6.1.1))
38.	HP TPASS_0226	6333555	B2S2H3	Cobalt transport protein
39.	HP TPASS_0231	6333017	B2S2H8	RNA pseudouridylate synthase
40.	HP TPASS_0245	6333539	B2S2J2	P-loop containing nucleoside triphosphate hydrolases
41.	HP TPASS_0246	6333538	B2S2J3	von Willebrand factor, type A(adhesive plasma glycoprotein)
42.	HP TPASS_0253	6333526	B2S2K1	Polymer forming cytoskeletal
43.	HP TPASS_0259	6333527	B2S2K7	Peptidoglycan binding(LysM domain- bacterial cell wall degradation)
44.	HP TPASS_0260	6333524	B2S2K8	SH3-like domain, bacterial-type
45.	HP TPASS_0263	6332801	B2S2L1	Fibronectin, type III
46.	HP TPASS_0267	6333000	B2S2L5	Polymer forming cytoskeletal
47.	HP TPASS_0268	6333517	B2S2L6	Tetratricopeptide repeat containing protein
48.	HP TPASS_0269	6333513	B2S2L7	Methylthiotransferase, N-terminal(Radical SAM enzyme)
49.	HP TPASS_0282	6332995	B2S2N0	Tetratricopeptide repeat containing protein
50.	HP TPASS_0285	6333505	B2S2N3	Iron-Sulfer cluster binding protein(SPASM)
51.	HP TPASS_0289	6332992	B2S2N7	S-adenosyl-L-methionine-dependent methyltransferases
52.	HP TPASS_0290	6333501	B2S2N8	Haloacid dehalogenase
53.	HP TPASS_0291	6333499	B2S2N9	FMN-dependent dehydrogenase
54.	HP TPASS_0296	6333498	B2S2P4	Dephospho-CoA kinase
55.	HP TPASS_0297	6333495	B2S2P5	sporulation and cell division repeat protein
56.	HP TPASS_0301	6332987	B2S2P9	Branched chain Amino acid ABC transporter(Permease)
57.	HP TPASS_0302	6333496	B2S2Q0	Branched chain Amino acid ABC transporter(Permease)
58.	HP TPASS_0304	6333493	B2S2Q2	Peptidase MA
59.	HP TPASS_0307	6332982	B2S2Q5	PASTA domain containing protein(penicillin binding- serine/threonine kinase)
60.	HP TPASS_0310	6332983	B2S2Q8	single-stranded DNA-binding protein
61.	HP TPASS_0333	6333459	B2S2T0	outer membrane lipoprotein carrier protein (LolA)
62.	HP TPASS_0334	6332972	B2S2T1	DNA-binding helix-turn-helix protein(transcriptional regulator)
63.	HP TPASS_0335	6333460	B2S2T2	CAAX amino terminal protease(Self Immunity)
64.	HP TPASS_0339	6333457	B2S2T6	RNA pseudouridylate synthase
65.	HP TPASS_0348	6332968	B2S2U5	Heptaprenyl diphosphate synthase
66.	HP TPASS_0352	6332965	B2S2U9	Transcriptional Coactivator p15
67.	HP TPASS_0358	6333387	B2S2V5	Glycosyl hydrolase
68.	HP TPASS_0369	6332954	B2S2W6	Tetratricopeptide repeat containing protein
69.	HP TPASS_0371	6333425	B2S2W8	4-(cytidine 5'-diphospho)-2-C-methyl-D-erythritol kinase
70.	HP TPASS_0373	6332951	B2S2X0	tRNA(Ile)-lysidine synthase
71.	HP TPASS_0374	6333405	B2S2X1	Tetratricopeptide repeat containing protein
72.	HP TPASS_0381	6333396	B2S2X8	Integral membrane protein
73.	HP TPASS_0384	6333390	B2S2Y1	S-adenosyl-L-methionine-dependent methyltransferases
74.	HP TPASS_0385	6332927	B2S2Y2	Cell division protein FtsL (Septum formation initiator)
75.	HP TPASS_0392	6333379	B2S2Y9	Tetratricopeptide repeat containing protein
76.	HP TPASS_0404	6333523	B2S301	Metallo-beta-lactamase
77.	HP TPASS_0412	6332827	B2S309	PUR-alpha/beta/gamma DNA/RNA-binding protein
78.	HP TPASS_0421	6333062	B2S318	Tetratricopeptide repeat containing protein
79.	HP TPASS_0423	6333060	B2S320	P-loop containing nucleoside triphosphate hydrolases
80.	HP TPASS_0431	6333579	B2S328	Pantothenate kinase, type III
81.	HP TPASS_0436	6333546	B2S332	Phosphoesterase(DHH family)
82.	HP TPASS_0438	6333091	B2S334	Non-canonical purine NTP pyrophosphatase
83.	HP TPASS_0441	6333698	B2S337	Inorganic polyphosphate/ATP-NAD kinase
84.	HP TPASS_0444	6333752	B2S340	Peptidoglycan binding(LysM domain),peptidase M23
85.	HP TPASS_0447	6333695	B2S343	Tetratricopeptide repeat containing protein
86.	HP TPASS_0449	6333086	B2S345	Tetratricopeptide repeat containing protein
87.	HP TPASS_0458	6333084	B2S354	Chromosome segregation and condensation protein
88.	HP TPASS_0459	6333689	B2S355	RNA pseudouridylate synthase
89.	HP TPASS_0460	6333688	B2S356	Tetratricopeptide repeat containing protein
90.	HP TPASS_0461	6333083	B2S357	DNA-binding helix-turn-helix protein(transcriptional regulator)
91.	HP TPASS_0464	6333686	B2S360	tRNA (guanine-N(7)-)-methyltransferase
92.	HP TPASS_0468	6333027	B2S364	Tetratricopeptide repeat containing protein
93.	HP TPASS_0470	6333673	B2S365	Tetratricopeptide repeat containing protein
94.	HP TPASS_0471	6333682	B2S366	Tetratricopeptide repeat containing protein
95.	HP TPASS_0474	6333683	B2S369	DNA-binding regulatory protein(Trasncriptional regulator)
96.	HP TPASS_0484	6333678	B2S379	FecR protein(regulation of iron dicitrate transport)
97.	HP TPASS_0487	6333676	B2S382	Quinoprotein alcohol dehydrogenase
98.	HP TPASS_0489	6333074	B2S384	Metallo-beta-lactamase
99.	HP TPASS_0494	6332850	B2S389	Zinc ribbon domain containing protein
100.	HP TPASS_0496	6333713	B2S390	Tetratricopeptide repeat containing protein
101.	HP TPASS_0502	6333709	B2S396	Ankyrin repeat protein(protein binding)
102.	HP TPASS_0512	6333669	B2S3A6	2-C-methyl-D-erythritol 2,4-cyclodiphosphate synthase
103.	HP TPASS_0515	6333671	B2S3A9	Organic solvent tolerance protein
104.	HP TPASS_0518	6333730	B2S3B2	Thiamin pyrophosphokinase
105.	HP TPASS_0522	6332853	B2S3B5	Colicin V production protein
106.	HP TPASS_0534	6333737	B2S3C6	V-type ATP synthase (subunit C)
107.	HP TPASS_0544	6332856	B2S3D6	Endonuclease/Exonuclease/phosphatase
108.	HP TPASS_0548	6333092	B2S3E0	Tetratricopeptide repeat containing protein
109.	HP TPASS_0558	6333675	B2S3F0	Nickel/cobalt transporter, high-affinity
110.	HP TPASS_0561	6332860	B2S3F3	TPM family (TLP18.3, Psb32 and MOLO-1 founding proteins of phosphatase)
111.	HP TPASS_0563	6333116	B2S3F5	DnaJ domain-containing protein(molecular chaperon)
112.	HP TPASS_0565	6333702	B2S3F7	SGNH hydrolase
113.	HP TPASS_0567	6333260	B2S3F9	MgtE N-terminal domain containing protein(flagellar protein)
114.	HP TPASS_0570	6333861	B2S3G2	TPM family (TLP18.3, Psb32 and MOLO-1 founding proteins of phosphatase)
115.	HP TPASS_0572	6332874	B2S3G4	FMN-binding domain (Ferric reductase)
116.	HP TPASS_0580	6333280	B2S3H1	Permease FtsX-like
117.	HP TPASS_0582	6333797	B2S3H3	Permease FtsX-like
118.	HP TPASS_0588	6333269	B2S3H9	DNA-directed DNA polymerase III delta subunit
119.	HP TPASS_0592	6332921	B2S3I3	D-alanyl-D-alanine carboxypeptidase
120.	HP TPASS_0599	6333138	B2S3I9	Zinc finger domain containing protein(DNA binding)
121.	HP TPASS_0608	6333867	B2S3J8	ARM repeat containing protein(intracellular signalling and cytoskeletal regulation)
122.	HP TPASS_0612	6333155	B2S3K2	Fe-S cluster assembly protein SufB
123.	HP TPASS_0613	6333336	B2S3K3	Fe-S cluster assembly protein SufB
124.	HP TPASS_0622	6332909	B2S3L2	Tetratricopeptide repeat containing protein
125.	HP TPASS_0624	6332843	B2S3L4	Outer membrane protein, OmpA
126.	HP TPASS_0625	6333262	B2S3L5	Tetratricopeptide repeat containing protein
127.	HP TPASS_0636	6332923	B2S3M5	DNA repair protein RecO(Recombination)
128.	HP TPASS_0648	6333773	B2S3N7	Tetratricopeptide repeat containing protein
129.	HP TPASS_0651	6333776	B2S3P0	Metal-dependent phosphohydrolase, (7TM intracellular domain)
130.	HP TPASS_0674	6332788	B2S3R3	Smr domain containing protein(DNA mismatch repair)
131.	HP TPASS_0675	6333351	B2S3R4	Pheromone shutdown, TraB
132.	HP TPASS_0691	6332917	B2S3T0	Prokaryotic chromosome segregation/condensation protein ScpA
133.	HP TPASS_0699	6333718	B2S3T8	Transcriptional regulator, MerR family
134.	HP TPASS_0702	6333746	B2S3U1	Peptidase M23
135.	HP TPASS_0706	6333365	B2S3U5	Peptidase M23
136.	HP TPASS_0710	6333662	B2S3U9	Jag_N family protein
137.	HP TPASS_0719	6333069	B2S3V8	Flagellar biosynthesis protein, FliO
138.	HP TPASS_0730	6333796	B2S3W9	CDP-diacylglycerol—glycerol-3-phosphate 3-phosphatidyltransferase
139.	HP TPASS_0731	6333510	B2S3X0	NUDIX hydrolase
140.	HP TPASS_0733	6333500	B2S3X2	Outer membrane protein (beta-barrel domain)
141.	HP TPASS_0738	6333488	B2S3X7	Iojap/ribosomal silencing factor
142.	HP TPASS_0739	6333471	B2S3X8	Cell envelope-related transcriptional attenuator
143.	HP TPASS_0740	6333287	B2S3X9	Metal dependent phosphohydrolases with conserved 'HD' moti
144.	HP TPASS_0741	6332971	B2S3Y0	nicotinate-nucleotide adenylyltransferase
145.	HP TPASS_0750	6333824	B2S3Y9	von Willebrand factor, type A(adhesive plasma glycoprotein)
146.	HP TPASS_0752	6333218	B2S3Z1	Sporulation and Cell division repeat protein
147.	HP TPASS_0764	6333596	B2S403	Metal dependent phosphohydrolases with conserved 'HD' motif
148.	HP TPASS_0771	6333854	B2S410	Sodium-dependent phosphate transport protein
149.	HP TPASS_0776	6333834	B2S415	Phosphoribosyltransferases
150.	HP TPASS_0777	6333848	B2S416	Zinc finger protein(DNA binding)
151.	HP TPASS_0782	6333666	B2S421	Peptidase M23
152.	HP TPASS_0784	6333829	B2S423	Lipopolysaccharide assembly, LptC
153.	HP TPASS_0785	6332804	B2S424	Organic solvent tolerance(N terminal)
154.	HP TPASS_0796	6333271	B2S435	Thiamine biosynthesis lipoprotein ApbE
155.	HP TPASS_0803	6333380	B2S442	Phosphoesterase(DHH family)
156.	HP TPASS_0815	6333371	B2S453	Acyl-CoA N-acyltransferase
157.	HP TPASS_0820	6332940	B2S458	Tetratricopeptide repeat containing protein
158.	HP TPASS_0822	6332941	B2S460	Mechanosensitive ion channel
159.	HP TPASS_0826	6332943	B2S464	DisA bacterial checkpoint controller nucleotide-binding
160.	HP TPASS_0832	6333406	B2S470	Sporulation and spore germination
161.	HP TPASS_0840	6333412	B2S478	Major facilitator superfamily domain, general substrate transporter
162.	HP TPASS_0846	6333419	B2S484	Cell division protein ZapA
163.	HP TPASS_0851	6333426	B2S489	UDP-3-O-acylglucosamine N-acyltransferase
164.	HP TPASS_0854	6333428	B2S492	HAMP domain-containing protein(regulation of phosphorylation or methylation of homodimeric receptors)
165.	HP TPASS_0860	6332961	B2S498	Tetratricopeptide repeat containing protein
166.	HP TPASS_0864	6332962	B2S4A2	Peptidase family M23 (LysM domain)
167.	HP TPASS_0875	6333650	B2S4B2	ATP-binding protein
168.	HP TPASS_0876	6333657	B2S4B3	Glycoprotease
169.	HP TPASS_0877	6333357	B2S4B4	Metal dependent phosphohydrolases with conserved 'HD' motif
170.	HP TPASS_0879	6333063	B2S4B6	ABC transporter
171.	HP TPASS_0882	6332835	B2S4B9	ARM repeat containing protein(intracellular signalling and cytoskeletal regulation)
172.	HP TPASS_0883	6333652	B2S4C0	Permease YjgP/YjgQ
173.	HP TPASS_0884	6333655	B2S4C1	Permease YjgP/YjgQ
174.	HP TPASS_0893	6333646	B2S4D0	Ribosome maturation factor RimP
175.	HP TPASS_0894	6333059	B2S4D1	NYN domain, limkain-b1-type
176.	HP TPASS_0899	6333640	B2S4D6	PD-(D/E)XK nuclease
177.	HP TPASS_0900	6333058	B2S4D7	PD-(D/E)XK nuclease
178.	HP TPASS_0901	6333638	B2S4D8	Multi antimicrobial extrusion protein
179.	HP TPASS_0906	6333635	B2S4E3	KH domain containing protein (RNA binding)
180.	HP TPASS_0907	6333636	B2S4E4	Ribosome maturation factor RimM
181.	HP TPASS_0911	6333054	B2S4E8	Flagellar biosynthetic protein flhb
182.	HP TPASS_0912	6333634	B2S4E9	Metal dependent phosphohydrolases with conserved 'HD' motif
183.	HP TPASS_0913	6332830	B2S4F0	Restriction endonuclease, type II
184.	HP TPASS_0915	6333052	B2S4F2	Tetratricopeptide repeat containing protein
185.	HP TPASS_0920	6333051	B2S4F7	Tetratricopeptide repeat containing protein
186.	HP TPASS_0923	6333048	B2S4G0	PEGA domain-containing protein
187.	HP TPASS_0931	6333617	B2S4G8	Alpha-alpha trehalase
188.	HP TPASS_0937	6333609	B2S4H4	Calcineurin-like phosphoesterase
189.	HP TPASS_0942	6333611	B2S4H9	flagellar protein(FlgN)
190.	HP TPASS_0944	6333040	B2S4I1	Tetratricopeptide repeat containing protein
191.	HP TPASS_0954	6332869	B2S4J1	Tetratricopeptide repeat containing protein
192.	HP TPASS_0959	6332970	B2S4J6	Rod binding protein(flagellar protein)
193.	HP TPASS_0962	6333182	B2S4J9	Permease FtsX-like(efflux ABC transporter)
194.	HP TPASS_0963	6333217	B2S4K0	Macrolide export ATP-binding/permease protein
195.	HP TPASS_0972	6333285	B2S4K9	Macrolide export ATP-binding/permease protein
196.	HP TPASS_0975	6333284	B2S4L2	rRNA small subunit methyltransferase I
197.	HP TPASS_0977	6333282	B2S4L4	NIF3 (NGG1p interacting factor 3)
198.	HP TPASS_0979	6332912	B2S4L6	TatD related DNase
199.	HP TPASS_0986	6333008	B2S4M3	Multidrug resistance efflux transporter EmrE
200.	HP TPASS_0988	6333049	B2S4M5	Multiple antibiotic resistance (MarC)-related
201.	HP TPASS_0990	6333035	B2S4M7	Tetratricopeptide repeat containing protein
202.	HP TPASS_0994	6333256	B2S4N1	TatD related DNase
203.	HP TPASS_1018	6333868	B2S4Q5	2',3'-cyclic-nucleotide 2'-phosphodiesterase
204.	HP TPASS_1029	6333264	B2S4R6	RNA binding protein
205.	HP TPASS_1032	6333263	B2S4R9	Transcription antitermination protein nusG
206.	HP TPASS_1033	6333866	B2S4S0	Patatin-like phospholipase
207.	HP TPASS_1034	6333278	B2S4S1	Sodium/calcium exchanger protein
