# Supplementary material for: Identification of Functional Candidates amongst Hypothetical Proteins of Treponema pallidum ssp. pallidum
Source: PLoS One. 2015 Apr 20;10(4):e0124177. doi: 10.1371/journal.pone.0124177 (PMC4403809; doi:10.1371/journal.pone.0124177)
Supplement: S1 Table — (DOC) [file pone.0124177.s001.doc]

| **Table S1: List of computed physicochemical parameters of 444 HPs from *T. pallidum ssp. pallidum*** | | | | | | | | | |
| --- | --- | --- | --- | --- | --- | --- | --- | --- | --- |
| **S. No** | **Protein name** | **Uniprot ID** | **Molecular weight, M_w_**  **(Da)** | **Theoretical PI** | **Extinction coefficient**  **(M^-1^ cm^-1^)** | **Instability Index** | | **Aliphatic index** | **Grand average of hydropathicity**  **(GRAVY)** |
|  |  |  |  |  |  | **computed** | **classification** |  |  |
|  | HP TPASS_0004 | **B2S1V4** | 16337.6 | 6.97 | 18450 | 37.08 | Stable | 90.62 | -0.326 |
|  | HP TPASS_0008 | **B2S1V7** | 10401.9 | 5.51 | 18450 | 18.31 | Stable | 87.64 | -0.284 |
|  | HP TPASS_0010 | **B2S1V8** | 4471.3 | 10.70 | 0 | 31.75 | Stable | 75.85 | -0.415 |
|  | HP TPASS_0012 | **B2S1W0** | 6837.0 | 9.10 | 19480 | 29.38 | Stable | 95.61 | 0.018 |
|  | HP TPASS_0013 | **B2S1W1** | 23872.3 | 6.49 | 20315 | 37.51 | Stable | 83.44 | -0.027 |
|  | HP TPASS_0014 | **B2S1W2** | 22940.1 | 7.81 | 18575 | 44.71 | Unstable | 85.35 | -0.287 |
|  | HP TPASS_0017 | **B2S1W5** | 36222.6 | 5.97 | 53080 | 39.98 | Stable | 106.51 | -0.146 |
|  | HP TPASS_0021 | **B2S1W9** | 10275.1 | 6.02 | 8480 | 21.98 | Stable | 117.39 | 0.373 |
|  | HP TPASS_0022 | **B2S1X0** | 80089.2 | 8.53 | 43985 | 42.68 | Unstable | 94.34 | -0.055 |
|  | HP TPASS_0024 | **B2S1X2** | 26746.1 | 5.88 | 13075 | 31.07 | Stable | 109.92 | 0.162 |
|  | HP TPASS_0025 | **B2S1X3** | 114104.8 | 6.30 | 103555 | 43.37 | Unstable | 94.35 | -0.065 |
|  | HP TPASS_0031 | **B2S1X9** | 10276.2 | 11.93 | 13980 | 25.31 | Stable | 115.33 | 0.699 |
|  | HP TPASS_0033 | **B2S1Y1** | 24058.8 | 9.63 | 49515 | 48.73 | Unstable | 130.10 | 0.872 |
|  | HP TPASS_0039 | **B2S1Y7** | 5933.6 | 6.54 | 1490 | 56.22 | Unstable | 61.92 | -0.862 |
|  | HP TPASS_0041 | **B2S1Y9** | 4391.0 | 10.95 | 2980 | 87.63 | Unstable | 70.75 | -0.593 |
|  | HP TPASS_0042 | **B2S1Z0** | 33200.3 | 5.23 | 24075 | 57.20 | Unstable | 82.81 | -0.208 |
|  | HP TPASS_0046 | **B2S1Z4** | 26872.2 | 8.36 | 16805 | 49.65 | Unstable | 82.83 | -0.177 |
|  | HP TPASS_0047 | **B2S1Z5** | 19641.6 | 9.25 | 10430 | 32.41 | Stable | 90.75 | -0.402 |
|  | HP TPASS_0048 | **B2S1Z6** | 15063.4 | 9.56 | 5960 | 29.92 | Stable | 106.85 | 0.248 |
|  | HP TPASS_0049 | **B2S1Z7** | 37497.3 | 9.53 | 41745 | 48.25 | Unstable | 76.96 | -0.319 |
|  | HP TPASS_0050 | **B2S1Z8** | 23207.7 | 7.75 | 38975 | 41.01 | Unstable | 96.98 | -0.127 |
|  | HP TPASS_0054 | **B2S202** | 35536.5 | 9.83 | 11960 | 48.57 | Unstable | 84.11 | -0.339 |
|  | HP TPASS_0055 | **B2S203** | 7934.4 | 7.85 | 5625 | 20.35 | Stable | 113.85 | 0.978 |
|  | HP TPASS_0059 | **B2S207** | 8153.3 | 8.66 | 1865 | 54.45 | Unstable | 54.80 | -0.013 |
|  | HP TPASS_0064 | **B2S212** | 22079.3 | 6.82 | 10930 | 65.89 | Unstable | 98.01 | -0.109 |
|  | HP TPASS_0065 | **B2S213** | 22594.5 | 9.83 | 16430 | 35.66 | Stable | 92.92 | -0.043 |
|  | HP TPASS_0066 | **B2S214** | 12012.9 | 9.99 | 8940 | 33.18 | Stable | 97.50 | -0.226 |
|  | HP TPASS_0067 | **B2S215** | 45755.2 | 8.53 | 63190 | 40.17 | Unstable | 82.93 | -0.473 |
|  | HP TPASS_0068 | **B2S216** | 37519.5 | 8.97 | 23335 | 31.58 | Stable | 95.26 | 0.011 |
|  | HP TPASS_0069 | **B2S217** | 19197.4 | 9.69 | 28335 | 37.97 | Stable | 99.15 | 0.229 |
|  | HP TPASS_0070 | **B2S218** | 13552.1 | 6.22 | 6210 | 35.85 | Stable | 128.12 | 0.963 |
|  | HP TPASS_0072 | **B2S220** | 10087.5 | 6.06 | 3105 | 28.83 | Stable | 84.44 | -0.218 |
|  | HP TPASS_0073 | **B2S221** | 58159.2 | 6.34 | 38070 | 46.55 | Unstable | 104.54 | -0.137 |
|  | HP TPASS_0079 | **B2S227** | 81720.8 | 7.78 | 77125 | 35.33 | Stable | 87.00 | 0.019 |
|  | HP TPASS_0081 | **B2S229** | 31611.5 | 9.64 | 26025 | 41.46 | Unstable | 100.67 | 0.038 |
|  | HP TPASS_0083 | **B2S231** | 48441.0 | 9.26 | 86540 | 40.96 | Unstable | 71.74 | -0.496 |
|  | HP TPASS_0084 | **B2S232** | 8224.2 | 4.25 | 1490 | 27.61 | Stable | 93.64 | 0.084 |
|  | HP TPASS_0086 | **B2S234** | 34814.1 | 8.33 | 13910 | 38.58 | Stable | 89.68 | 0.048 |
|  | HP TPASS_0087 | **B2S235** | 20083.2 | 9.89 | 20065 | 52.99 | Unstable | 92.63 | -0.125 |
|  | HP TPASS_0088 | **B2S236** | 23230.7 | 9.40 | 27515 | 35.37 | Stable | 83.73 | -0.316 |
|  | HP TPASS_0093 | **B2S241** | 23443.3 | 9.71 | 27555 | 51.53 | Unstable | 80.79 | 0.159 |
|  | HP TPASS_0095 | **B2S243** | 72379.6 | 9.16 | 102400 | 45.86 | Unstable | 88.90 | -0.232 |
|  | HP TPASS_0110 | **B2S258** | 65149.9 | 8.50 | 88725 | 40.41 | Unstable | 89.50 | -0.104 |
|  | HP TPASS_0118 | **B2S266** | 46702.2 | 6.35 | 17140 | 59.28 | Unstable | 94.42 | -0.246 |
|  | HP TPASS_0121 | **B2S269** | 39102.0 | 8.22 | 45670 | 49.70 | Unstable | 91.04 | 0.010 |
|  | HP TPASS_0123 | **B2S271** | 97894.1 | 6.11 | 99155 | 44.94 | Unstable | 83.49 | -0.002 |
|  | HP TPASS_0126 | **B2S274** | 31921.0 | 9.32 | 69830 | 30.75 | Stable | 69.14 | -0.300 |
|  | HP TPASS_0127 | **B2S275** | 13858.2 | 9.44 | 28670 | 19.67 | Stable | 89.68 | 0.225 |
|  | HP TPASS_0128 | **B2S276** | 11763.5 | 6.70 | 8730 | 47.26 | Unstable | 87.39 | 0.400 |
|  | HP TPASS_0129 | **B2S277** | 16857.8 | 9.43 | 10845 | 66.69 | Unstable | 68.73 | 0.072 |
|  | HP TPASS_0130 | **B2S278** | 15094.1 | 9.57 | 10345 | 47.45 | Unstable | 52.73 | -0.716 |
|  | HP TPASS_0132 | **B2S280** | 2904.4 | 12.40 | - | 108.44 | Unstable | 62.40 | -1.072 |
|  | HP TPASS_0133 | **B2S281** | 43227.0 | 6.27 | 55640 | 27.82 | Stable | 68.76 | -0.391 |
|  | HP TPASS_0134 | **B2S282** | 40131.0 | 6.87 | 65610 | 26.50 | Stable | 72.63 | -0.285 |
|  | HP TPASS_0135 | **B2S283** | 33849.1 | 10.65 | 16555 | 50.63 | Unstable | 81.73 | -0.160 |
|  | HP TPASS_0136 | **B2S284** | 50059.2 | 7.71 | 63715 | 45.75 | Unstable | 57.50 | -0.279 |
|  | HP TPASS_0137 | **B2S285** | 4787.7 | 7.98 | 1490 | 55.69 | Unstable | 127.78 | 0.753 |
|  | HP TPASS_0138 | **B2S286** | 26964.8 | 9.30 | 6085 | 32.21 | Stable | 111.92 | 0.246 |
|  | HP TPASS_0139 | **B2S287** | 25323.3 | 5.80 | 5960 | 34.09 | Stable | 113.17 | 0.129 |
|  | HP TPASS_0148 | **B2S295** | 21083.1 | 8.58 | 31565 | 27.75 | Stable | 111.82 | 0.836 |
|  | HP TPASS_0149 | **B2S296** | 21541.6 | 8.48 | 22960 | 44.15 | Unstable | 114.37 | 0.929 |
|  | HP TPASS_0150 | **B2S297** | 17288.0 | 9.44 | 16180 | 37.34 | Stable | 84.45 | -0.029 |
|  | HP TPASS_0151 | **B2S298** | 36905.3 | 9.05 | 57005 | 35.56 | Stable | 108.23 | 0.625 |
|  | HP TPASS_0153 | **B2S2A0** | 17513.3 | 9.30 | 14105 | 45.35 | Unstable | 102.27 | 0.569 |
|  | HP TPASS_0154 | **B2S2A1** | 37536.6 | 11.15 | 30075 | 51.65 | Unstable | 87.10 | -0.059 |
|  | HP TPASS_0156 | **B2S2A3** | 15366.5 | 7.83 | 22015 | 33.33 | Stable | 88.81 | -0.108 |
|  | HP TPASS_0157 | **B2S2A4** | 34468.6 | 6.91 | 20650 | 49.14 | Unstable | 97.53 | -0.361 |
|  | HP TPASS_0158 | **B2S2A5** | 26478.4 | 6.42 | 21680 | 49.26 | Unstable | 94.56 | -0.124 |
|  | HP TPASS_0159 | **B2S2A6** | 39242.9 | 8.85 | 48610 | 45.50 | Unstable | 86.17 | -0.227 |
|  | HP TPASS_0161 | **B2S2A8** | 3258.6 | 6.05 | 2980 | 67.74 | Unstable | 58.67 | -0.637 |
|  | HP TPASS_0169 | **B2S2B6** | 3616.1 | 9.28 | 11000 | 9.98 | Stable | 58.13 | 0.200 |
|  | HP TPASS_0172 | **B2S2B9** | 19543.8 | 5.23 | 18700 | 45.03 | Unstable | 73.09 | -0.382 |
|  | HP TPASS_0173 | **B2S2C0** | 25260.5 | 9.53 | 18825 | 51.68 | Unstable | 95.81 | 0.335 |
|  | HP TPASS_0174 | **B2S2C1** | 31762.8 | 9.63 | 30870 | 36.81 | Stable | 82.96 | 0.041 |
|  | HP TPASS_0175 | **B2S2C2** | 24239.1 | 8.62 | 32680 | 46.05 | Unstable | 89.62 | 0.069 |
|  | HP TPASS_0176 | **B2S2C3** | 10104.4 | 10.48 | 9970 | 26.66 | Stable | 71.02 | -0.367 |
|  | HP TPASS_0177 | **B2S2C4** | 49532.7 | 9.49 | 51255 | 55.00 | Unstable | 85.30 | -0.215 |
|  | HP TPASS_0178 | **B2S2C5** | 34683.6 | 5.90 | 20775 | 50.68 | Unstable | 94.35 | 0.025 |
|  | HP TPASS_0179 | **B2S2C6** | 66564.9 | 4.29 | 31900 | 63.28 | Unstable | 63.80 | -0.583 |
|  | HP TPASS_0180 | **B2S2C7** | 5518.4 | 7.73 | 1740 | 71.47 | Unstable | 62.12 | -0.035 |
|  | HP TPASS_0181 | **B2S2C8** | 16464.2 | 8.76 | 20190 | 28.51 | Stable | 108.78 | 0.347 |
|  | HP TPASS_0182 | **B2S2C9** | 27744.2 | 8.88 | 19075 | 48.03 | Unstable | 98.38 | 0.144 |
|  | HP TPASS_0183 | **B2S2D0** | 31059.4 | 9.44 | 24075 | 35.71 | Stable | 92.95 | -0.216 |
|  | HP TPASS_0214 | **B2S2G2** | 7005.2 | 11.03 | 1615 | 27.20 | Stable | 73.38 | -0.637 |
|  | HP TPASS_0222 | **B2S2G9** | 16224.0 | 9.37 | 13980 | 53.94 | Unstable | 119.39 | 0.126 |
|  | HP TPASS_0223 | **B2S2H0** | 47304.5 | 5.50 | 42495 | 45.99 | Unstable | 93.38 | 0.180 |
|  | HP TPASS_0224 | **B2S2H1** | 3829.6 | 9.62 | 6990 | 12.12 | Stable | 114.71 | 0.474 |
|  | HP TPASS_0226 | **B2S2H3** | 26202.9 | 9.86 | 34420 | 43.96 | Unstable | 111.23 | 0.491 |
|  | HP TPASS_0231 | **B2S2H8** | 23859.3 | 10.87 | 13325 | 34.00 | Stable | 109.81 | 0.181 |
|  | HP TPASS_0232 | **B2S2H9** | 3914.6 | 9.10 | 3105 | 45.76 | Unstable | 95.00 | 0.676 |
|  | HP TPASS_0245 | **B2S2J2** | 127557.6 | 5.67 | 113815 | 35.69 | Stable | 91.61 | -0.123 |
|  | HP TPASS_0246 | **B2S2J3** | 69813.7 | 8.90 | 83240 | 46.22 | Unstable | 82.75 | -0.358 |
|  | HP TPASS_0248 | **B2S2J5** | 14843.4 | 9.22 | 24450 | 31.45 | Stable | 83.51 | 0.045 |
|  | HP TPASS_0250a | **B2S2J7** | 8157.9 | 10.74 | 25105 | 30.35 | Stable | 128.40 | 1.013 |
|  | HP TPASS_0253 | **B2S2K1** | 14872.1 | 9.46 | 1740 | 29.70 | Stable | 88.10 | -0.055 |
|  | HP TPASS_0258 | **B2S2K6** | 28147.0 | 8.66 | 53650 | 41.26 | Unstable | 77.46 | -0.498 |
|  | HP TPASS_0259 | **B2S2K7** | 22864.4 | 4.86 | 14815 | 53.56 | Unstable | 77.78 | -0.248 |
|  | HP TPASS_0260 | **B2S2K8** | 51247.4 | 7.17 | 89645 | 43.66 | Unstable | 89.00 | -0.277 |
|  | HP TPASS_0263 | **B2S2L1** | 65845.4 | 6.96 | 110280 | 50.63 | Unstable | 86.18 | -0.199 |
|  | HP TPASS_0266 | **B2S2L4** | 4904.9 | 9.74 | 14105 | 7.17 | Stable | 101.71 | 0.259 |
|  | HP TPASS_0267 | **B2S2L5** | 13013.6 | 4.11 | 1615 | 40.27 | Unstable | 90.83 | 0.274 |
|  | HP TPASS_0268 | **B2S2L6** | 24854.5 | 8.95 | 41870 | 42.48 | Unstable | 93.73 | -0.046 |
|  | HP TPASS_0269 | **B2S2L7** | 53173.4 | 9.19 | 24130 | 43.48 | Unstable | 96.14 | 0.041 |
|  | HP TPASS_0273 | **B2S2M1** | 29742.4 | 8.97 | 65525 | 38.56 | Stable | 105.32 | 0.655 |
|  | HP TPASS_0278 | **B2S2M6** | 4831.4 | 5.59 | 11125 | 59.85 | Unstable | 65.24 | -0.676 |
|  | HP TPASS_0280 | **B2S2M8** | 4398.0 | 8.54 | 5750 | 53.79 | Unstable | 67.14 | 0.383 |
|  | HP TPASS_0281 | **B2S2M9** | 4462.2 | 8.68 | 7115 | 41.40 | Unstable | 111.19 | 0.736 |
|  | HP TPASS_0282 | **B2S2N0** | 26435.2 | 9.86 | 37150 | 39.81 | Stable | 86.32 | -0.158 |
|  | HP TPASS_0284 | **B2S2N2** | 23561.3 | 5.80 | 12545 | 41.45 | Unstable | 94.11 | 0.477 |
|  | HP TPASS_0285 | **B2S2N3** | 41255.3 | 5.63 | 57590 | 47.58 | Unstable | 89.64 | -0.075 |
|  | HP TPASS_0286 | **B2S2N4** | 13162.5 | 4.97 | 12615 | 32.50 | Stable | 115.12 | 0.648 |
|  | HP TPASS_0287 | **B2S2N5** | 24232.5 | 8.60 | 37150 | 43.54 | Unstable | 74.06 | -0.360 |
|  | HP TPASS_0289 | **B2S2N7** | 59551.5 | 6.74 | 61725 | 40.10 | Unstable | 85.82 | 0.033 |
|  | HP TPASS_0290 | **B2S2N8** | 30938.5 | 6.20 | 18255 | 35.91 | Stable | 86.93 | -0.118 |
|  | HP TPASS_0291 | **B2S2N9** | 32305.6 | 9.52 | 31900 | 26.74 | Stable | 93.79 | -0.017 |
|  | HP TPASS_0293 | **B2S2P1** | 6822.1 | 10.74 | 10095 | 29.78 | Stable | 102.41 | 0.307 |
|  | HP TPASS_0296 | **B2S2P4** | 23583.5 | 9.81 | 18950 | 42.06 | Unstable | 100.85 | -0.033 |
|  | HP TPASS_0297 | **B2S2P5** | 29580.7 | 10.24 | 24075 | 42.43 | Unstable | 76.30 | -0.409 |
|  | HP TPASS_0299 | **B2S2P7** | 7929.1 | 10.75 | 7115 | 29.71 | Stable | 59.46 | -0.336 |
|  | HP TPASS_0301 | **B2S2P9** | 40888.4 | 9.70 | 65025 | 36.34 | Stable | 112.31 | 0.821 |
|  | HP TPASS_0302 | **B2S2Q0** | 33533.7 | 9.67 | 51045 | 31.07 | Stable | 101.02 | 0.812 |
|  | HP TPASS_0304 | **B2S2Q2** | 115814.7 | 8.45 | 139550 | 38.39 | Stable | 75.45 | -0.246 |
|  | HP TPASS_0307 | **B2S2Q5** | 37094.6 | 6.19 | 18005 | 33.34 | Stable | 92.89 | 0.039 |
|  | HP TPASS_0310 | **B2S2Q8** | 13765.7 | 7.70 | 18240 | 39.78 | Stable | 88.19 | -0.029 |
|  | HP TPASS_0311 | **B2S2Q9** | 5056.8 | 7.83 | 5625 | 31.98 | Stable | 62.34 | -0.153 |
|  | HP TPASS_0312 | **B2S2R0** | 34712.0 | 9.81 | 43720 | 22.17 | Stable | 112.11 | 0.857 |
|  | HP TPASS_0314 | **B2S2R2** | 5442.3 | 9.99 | 8480 | 24.80 | Stable | 81.25 | 0.169 |
|  | HP TPASS_0315 | **B2S2R3** | 22796.3 | 8.73 | 20565 | 40.14 | Unstable | 88.00 | 0.153 |
|  | HP TPASS_0318 | **B2S2R5** | 6123.0 | 8.79 | 22250 | 10.01 | Stable | 70.85 | 0.134 |
|  | HP TPASS_0320 | **B2S2R7** | 5332.1 | 9.49 | 3105 | 71.50 | Unstable | 64.90 | 0.065 |
|  | HP TPASS_0324 | **B2S2S1** | 53603.0 | 7.23 | 65360 | 31.71 | Stable | 92.23 | 0.029 |
|  | HP TPASS_0325 | **B2S2S2** | 108468.3 | 5.36 | 120835 | 31.18 | Stable | 87.84 | -0.039 |
|  | HP TPASS_0332 | **B2S2S9** | 4642.4 | 7.71 | 3105 | 24.25 | Stable | 106.83 | 0.529 |
|  | HP TPASS_0333 | **B2S2T0** | 25558.5 | 9.65 | 17545 | 50.23 | Unstable | 94.17 | 0.099 |
|  | HP TPASS_0334 | **B2S2T1** | 45308.3 | 6.49 | 42080 | 35.92 | Stable | 87.33 | -0.286 |
|  | HP TPASS_0335 | **B2S2T2** | 19218.5 | 8.89 | 13450 | 59.24 | Unstable | 101.61 | 0.594 |
|  | HP TPASS_0338 | **B2S2T5** | 18367.2 | 4.68 | 29450 | 40.71 | Unstable | 108.89 | 0.286 |
|  | HP TPASS_0339 | **B2S2T6** | 37742.1 | 10.55 | 25035 | 38.61 | Stable | 90.94 | -0.021 |
|  | HP TPASS_0346 | **B2S2U3** | 25289.4 | 9.16 | 42650 | 21.58 | Stable | 100.77 | 0.489 |
|  | HP TPASS_0347 | **B2S2U4** | 29917.0 | 8.85 | 52075 | 30.69 | Stable | 98.59 | 0.567 |
|  | HP TPASS_0348 | **B2S2U5** | 50956.0 | 10.10 | 50515 | 42.75 | Unstable | 102.32 | 0.289 |
|  | HP TPASS_0352 | **B2S2U9** | 9662.8 | 6.56 | 26470 | 28.15 | Stable | 71.06 | -0.712 |
|  | HP TPASS_0355 | **B2S2V2** | 14526.5 | 9.62 | 14815 | 66.50 | Unstable | 58.43 | -0.641 |
|  | HP TPASS_0358 | **B2S2V5** | 59700.6 | 6.42 | 62185 | 46.47 | Unstable | 83.54 | -0.088 |
|  | HP TPASS_0359 | **B2S2V6** | 23888.3 | 5.31 | 22960 | 67.56 | Unstable | 98.44 | -0.079 |
|  | HP TPASS_0360 | **B2S2V7** | 6438.4 | 10.21 | 250 | 60.26 | Unstable | 55.52 | -0.693 |
|  | HP TPASS_0368 | **B2S2W5** | 12400.4 | 5.69 | 19480 | 59.14 | Unstable | 99.37 | -0.123 |
|  | HP TPASS_0369 | **B2S2W6** | 55859.7 | 5.99 | 35535 | 66.12 | Unstable | 66.41 | -0.607 |
|  | HP TPASS_0370 | **B2S2W7** | 15998.0 | 3.85 | 9065 | 52.36 | Unstable | 51.59 | -1.009 |
|  | HP TPASS_0371 | **B2S2W8** | 31591.5 | 9.23 | 47035 | 51.74 | Unstable | 92.58 | 0.066 |
|  | HP TPASS_0373 | **B2S2X0** | 52605.7 | 9.69 | 46505 | 42.91 | Stable | 87.59 | -0.056 |
|  | HP TPASS_0374 | **B2S2X1** | 89451.8 | 6.88 | 109710 | 48.31 | Unstable | 78.46 | -0.420 |
|  | HP TPASS_0375 | **B2S2X2** | 7353.6 | 10.30 | 7115 | 62.43 | Unstable | 103.33 | 0.670 |
|  | HP TPASS_0376 | **B2S2X3** | 33685.2 | 9.06 | 29005 | 45.10 | Unstable | 88.01 | -0.263 |
|  | HP TPASS_0377 | **B2S2X4** | 4400.4 | 8.71 | 125 | 38.27 | Stable | 140.24 | 1.051 |
|  | HP TPASS_0381 | **B2S2X8** | 27147.8 | 10.16 | 30160 | 38.92 | Stable | 135.67 | 0.902 |
|  | HP TPASS_0382 | **B2S2X9** | 4103.6 | 5.54 | 125 | 11.41 | Stable | 78.00 | -0.053 |
|  | HP TPASS_0384 | **B2S2Y1** | 40937.4 | 10.13 | 20565 | 48.26 | Unstable | 87.44 | 0.008 |
|  | HP TPASS_0385 | **B2S2Y2** | 14300.7 | 10.75 | 8480 | 40.61 | Unstable | 90.48 | -0.523 |
|  | HP TPASS_0392 | **B2S2Y9** | 37032.9 | 9.88 | 42330 | 51.03 | Unstable | 85.39 | -0.250 |
|  | HP TPASS_0404 | **B2S301** | 19490.9 | 6.28 | 18045 | 33.46 | Stable | 82.26 | -0.094 |
|  | HP TPASS_0408 | **B2S305** | 124032.7 | 5.32 | 53455 | 40.13 | Unstable | 86.40 | -0.637 |
|  | HP TPASS_0409 | **B2S306** | 7158.2 | 8.63 | 19605 | 61.29 | Unstable | 67.38 | -0.202 |
|  | HP TPASS_0412 | **B2S309** | 11929.6 | 9.81 | 1490 | 32.57 | Stable | 74.16 | -0.727 |
|  | HP TPASS_0415 | **B2S312** | 19319.2 | 9.71 | 30855 | 32.92 | Stable | 71.02 | -0.292 |
|  | HP TPASS_0420 | **B2S317** | 8256.4 | 6.01 | 11585 | 51.02 | Unstable | 82.00 | 0.073 |
|  | HP TPASS_0421 | **B2S318** | 74518.7 | 8.17 | 69010 | 36.23 | Stable | 95.27 | 0.021 |
|  | HP TPASS_0422 | **B2S319** | 37978.2 | 9.57 | 55600 | 35.20 | Stable | 81.03 | -0.108 |
|  | HP TPASS_0423 | **B2S320** | 30084.0 | 10.20 | 21220 | 61.62 | Unstable | 97.16 | -0.165 |
|  | HP TPASS_0425 | **B2S322** | 9737.1 | 7.67 | 12740 | 54.55 | Unstable | 83.95 | -0.183 |
|  | HP TPASS_0431 | **B2S328** | 28457.9 | 6.64 | 10345 | 26.81 | Stable | 112.12 | 0.466 |
|  | HP TPASS_0432 | **B2S329** | 19537.3 | 6.05 | 25815 | 44.21 | Unstable | 88.42 | 0.273 |
|  | HP TPASS_0436 | **B2S332** | 38168.3 | 6.80 | 7950 | 40.28 | Stable | 87.92 | 0.070 |
|  | HP TPASS_0437 | **B2S333** | 19892.9 | 9.54 | 23045 | 57.13 | Unstable | 82.50 | -0.132 |
|  | HP TPASS_0438 | **B2S334** | 30008.8 | 10.14 | 14940 | 46.30 | Unstable | 84.94 | -0.154 |
|  | HP TPASS_0441 | **B2S337** | 32633.6 | 8.56 | 23670 | 52.01 | Unstable | 97.25 | 0.228 |
|  | HP TPASS_0443 | **B2S339** | 33010.7 | 8.85 | 44935 | 41.91 | Unstable | 92.75 | -0.508 |
|  | HP TPASS_0444 | **B2S340** | 36588.1 | 10.19 | 30620 | 31.86 | Stable | 90.76 | -0.042 |
|  | HP TPASS_0447 | **B2S343** | 43127.2 | 7.19 | 66935 | 40.85 | Unstable | 95.83 | -0.269 |
|  | HP TPASS_0449 | **B2S345** | 22601.7 | 6.65 | 29590 | 42.77 | Unstable | 86.79 | -0.219 |
|  | HP TPASS_0451 | **B2S347** | 6624.8 | 9.62 | 2980 | 54.08 | Unstable | 91.00 | 0.235 |
|  | HP TPASS_0453 | **B2S349** | 31999.6 | 9.28 | 36120 | 39.44 | Stable | 87.00 | -0.134 |
|  | HP TPASS_0454 | **B2S350** | 24912.3 | 5.58 | 10470 | 30.13 | Stable | 96.16 | 0.110 |
|  | HP TPASS_0455 | **B2S351** | 38895.2 | 8.99 | 63955 | 44.98 | Unstable | 81.47 | -0.497 |
|  | HP TPASS_0456 | **B2S352** | 4735.4 | 8.82 | 48400 | 46.75 | Unstable | 77.85 | -0.161 |
|  | HP TPASS_0457 | **B2S353** | 65567.2 | 9.39 | 77030 | 45.71 | Unstable | 88.84 | -0.123 |
|  | HP TPASS_0458 | **B2S354** | 20775.9 | 5.84 | 6210 | 49.24 | Unstable | 99.74 | -0.084 |
|  | HP TPASS_0459 | **B2S355** | 29216.0 | 9.93 | 26525 | 38.54 | Stable | 97.43 | -0.024 |
|  | HP TPASS_0460 | **B2S356** | 25957.6 | 9.26 | 37400 | 48.78 | Unstable | 79.49 | -0.294 |
|  | HP TPASS_0461 | **B2S357** | 13028.9 | 4.83 | 12615 | 58.31 | Unstable | 82.77 | -0.215 |
|  | HP TPASS_0462 | **B2S358** | 28218.6 | 8.49 | 23795 | 25.99 | Stable | 69.25 | -0.035 |
|  | HP TPASS_0463 | **B2S359** | 11828.9 | 4.78 | 23950 | 15.56 | Stable | 56.64 | -0.763 |
|  | HP TPASS_0464 | **B2S360** | 28068.4 | 10.23 | 41035 | 60.20 | Unstable | 81.04 | -0.286 |
|  | HP TPASS_0465 | **B2S361** | 32670.4 | 6.79 | 50225 | 52.23 | Unstable | 81.03 | -0.093 |
|  | HP TPASS_0466 | **B2S362** | 44762.3 | 8.37 | 37735 | 43.65 | Unstable | 98.53 | -0.284 |
|  | HP TPASS_0467 | **B2S363** | 9614.0 | 10.03 | 10095 | 75.08 | Unstable | 61.83 | -0.811 |
|  | HP TPASS_0468 | **B2S364** | 72162.3 | 6.40 | 58010 | 37.72 | Stable | 89.23 | -0.379 |
|  | HP TPASS_0470 | **B2S365** | 36513.8 | 8.61 | 34965 | 68.30 | Unstable | 62.68 | -1.078 |
|  | HP TPASS_0471 | **B2S366** | 52933.9 | 8.50 | 39600 | 36.60 | Stable | 94.97 | -0.146 |
|  | HP TPASS_0473 | **B2S368** | 24019.6 | 6.49 | 16055 | 28.29 | Stable | 122.06 | 1.047 |
|  | HP TPASS_0474 | **B2S369** | 26325.6 | 4.95 | 21430 | 41.20 | Unstable | 87.67 | -0.264 |
|  | HP TPASS_0479 | **B2S374** | 24238.3 | 9.89 | 30605 | 17.54 | Stable | 99.64 | 0.293 |
|  | HP TPASS_0480 | **B2S375** | 18170.8 | 9.78 | 28795 | 36.96 | Stable | 129.01 | 0.855 |
|  | HP TPASS_0481 | **B2S376** | 53180.4 | 10.49 | 37400 | 45.61 | Unstable | 89.90 | -0.192 |
|  | HP TPASS_0482 | **B2S377** | 30610.8 | 8.88 | 2980 | 43.07 | Unstable | 98.69 | -0.023 |
|  | HP TPASS_0484 | **B2S379** | 75837.6 | 9.65 | 95465 | 44.63 | Unstable | 84.71 | -0.319 |
|  | HP TPASS_0487 | **B2S382** | 57120.8 | 9.64 | 52215 | 47.56 | Unstable | 75.26 | -0.334 |
|  | HP TPASS_0489 | **B2S384** | 37032.2 | 8.64 | 55265 | 34.64 | Stable | 75.06 | -0.310 |
|  | HP TPASS_0490 | **B2S385** | 7230.2 | 5.07 | 1615 | 74.09 | Unstable | 74.03 | -0.303 |
|  | HP TPASS_0491 | **B2S386** | 38363.9 | 9.62 | 33390 | 52.08 | Unstable | 94.16 | 0.172 |
|  | HP TPASS_0494 | **B2S389** | 31433.3 | 4.69 | 12295 | 66.29 | Unstable | 85.68 | -0.619 |
|  | HP TPASS_0496 | **B2S390** | 37761.3 | 9.24 | 53875 | 41.48 | Unstable | 93.43 | -0.163 |
|  | HP TPASS_0502 | **B2S396** | 32802.6 | 5.56 | 14815 | 24.95 | Stable | 98.22 | 0.112 |
|  | HP TPASS_0503 | **B2S397** | 21409.7 | 10.00 | 35535 | 52.26 | Unstable | 92.14 | -0.279 |
|  | HP TPASS_0504 | **B2S398** | 5037.5 | 8.31 | 1490 | 51.34 | Unstable | 44.78 | -1.028 |
|  | HP TPASS_0512 | **B2S3A6** | 44013.6 | 8.55 | 30660 | 46.84 | Unstable | 97.40 | 0.147 |
|  | HP TPASS_0515 | **B2S3A9** | 112617.6 | 8.99 | 176270 | 46.95 | Unstable | 78.92 | -0.284 |
|  | HP TPASS_0518 | **B2S3B2** | 25566.9 | 5.69 | 31650 | 35.29 | Stable | 85.65 | -0.034 |
|  | HP TPASS_0522 | **B2S3B5** | 17809.7 | 9.61 | 4720 | 36.54 | Stable | 138.99 | 1.104 |
|  | HP TPASS_0534 | **B2S3C6** | 38991.9 | 8.48 | 97245 | 48.24 | Unstable | 87.57 | -0.038 |
|  | HP TPASS_0535 | **B2S3C7** | 7878.6 | 4.30 | 5625 | 47.07 | Unstable | 69.86 | -0.456 |
|  | HP TPASS_0539 | **B2S3D1** | 7878.1 | 6.95 | 4595 | 40.04 | Unstable | 112.19 | 0.481 |
|  | HP TPASS_0544 | **B2S3D6** | 68364.1 | 7.68 | 55280 | 41.24 | Unstable | 79.81 | -0.235 |
|  | HP TPASS_0548 | **B2S3E0** | 47328.7 | 9.51 | 36245 | 33.95 | Stable | 75.14 | -0.135 |
|  | HP TPASS_0552 | **B2S3E4** | 20171.8 | 7.89 | 39880 | 30.35 | Stable | 83.73 | -0.341 |
|  | HP TPASS_0553 | **B2S3E5** | 45283.9 | 9.45 | 28920 | 38.11 | Stable | 113.25 | 0.722 |
|  | HP TPASS_0557 | **B2S3E9** | 27762.3 | 9.26 | 48485 | 48.08 | Unstable | 63.42 | -0.541 |
|  | HP TPASS_0558 | **B2S3F0** | 32206.3 | 10.38 | 52410 | 29.76 | Stable | 111.89 | 0.748 |
|  | HP TPASS_0561 | **B2S3F3** | 29876.0 | 9.12 | 29700 | 42.50 | Unstable | 92.40 | 0.093 |
|  | HP TPASS_0563 | **B2S3F5** | 15723.9 | 9.24 | 24075 | 65.11 | Unstable | 61.90 | -0.781 |
|  | HP TPASS_0564 | **B2S3F6** | 76524.1 | 9.01 | 72935 | 41.87 | Unstable | 106.56 | 0.240 |
|  | HP TPASS_0565 | **B2S3F7** | 45989.9 | 9.10 | 64665 | 54.73 | Unstable | 87.66 | -0.182 |
|  | HP TPASS_0567 | **B2S3F9** | 23468.0 | 5.70 | 19940 | 47.26 | Unstable | 98.54 | -0.402 |
|  | HP TPASS_0570 | **B2S3G2** | 30969.9 | 8.73 | 40825 | 43.01 | Unstable | 99.96 | 0.240 |
|  | HP TPASS_0572 | **B2S3G4** | 38633.5 | 9.93 | 36370 | 32.29 | Stable | 109.94 | 0.506 |
|  | HP TPASS_0573 | **B2S3G5** | 3379.9 | 9.82 | 1490 | 22.89 | Stable | 71.67 | -0.263 |
|  | HP TPASS_0577 | **B2S3G8** | 68151.0 | 10.03 | 40465 | 45.05 | Unstable | 88.27 | -0.213 |
|  | HP TPASS_0579 | **B2S3H0** | 29463.7 | 6.34 | 44600 | 31.42 | Stable | 63.60 | -0.664 |
|  | HP TPASS_0580 | **B2S3H1** | 47955.0 | 9.57 | 37485 | 32.91 | Stable | 110.21 | 0.387 |
|  | HP TPASS_0582 | **B2S3H3** | 54133.2 | 9.43 | 40170 | 40.99 | Unstable | 113.27 | 0.487 |
|  | HP TPASS_0583 | **B2S3H4** | 4572.1 | 8.63 | 12615 | 78.90 | Unstable | 42.93 | -0.732 |
|  | HP TPASS_0584 | **B2S3H5** | 53543.6 | 9.07 | 29590 | 36.70 | Stable | 89.64 | -0.333 |
|  | HP TPASS_0587 | **B2S3H8** | 8550.8 | 6.80 | 11585 | 44.72 | Unstable | 95.13 | -0.017 |
|  | HP TPASS_0588 | **B2S3H9** | 29124.6 | 7.15 | 26720 | 47.85 | Unstable | 106.65 | -0.038 |
|  | HP TPASS_0590 | **B2S3I1** | 4357.1 | 12.01 | 0 | 24.04 | Stable | 64.21 | -0.811 |
|  | HP TPASS_0592 | **B2S3I3** | 58268.6 | 9.46 | 116685 | 57.64 | Unstable | 83.82 | -0.454 |
|  | HP TPASS_0593 | **B2S3I4** | 75492.7 | 5.52 | 54820 | 43.52 | Unstable | 92.35 | -0.179 |
|  | HP TPASS_0594 | **B2S3I5** | 22107.8 | 9.16 | 42775 | 30.35 | Stable | 83.86 | 0.065 |
|  | HP TPASS_0598 | **B2S3I8** | 75793.7 | 8.86 | 86095 | 38.00 | Stable | 83.58 | -0.200 |
|  | HP TPASS_0599 | **B2S3I9** | 24977.6 | 5.21 | 21930 | 39.36 | Stable | 87.20 | -0.076 |
|  | HP TPASS_0607 | **B2S3J7** | 5451.2 | 10.01 | 8480 | 36.26 | Stable | 68.96 | -0.417 |
|  | HP TPASS_0608 | **B2S3J8** | 31909.8 | 8.65 | 17585 | 37.64 | Stable | 89.32 | 0.041 |
|  | HP TPASS_0612 | **B2S3K2** | 54106.3 | 6.45 | 64290 | 42.25 | Unstable | 90.96 | -0.352 |
|  | HP TPASS_0613 | **B2S3K3** | 43024.8 | 8.37 | 20900 | 34.84 | Stable | 85.50 | -0.293 |
|  | HP TPASS_0617 | **B2S3K7** | 10317.9 | 9.22 | 17085 | 37.85 | Stable | 82.61 | -0.059 |
|  | HP TPASS_0618 | **B2S3K8** | 12706.7 | 5.04 | 17210 | 24.36 | Stable | 89.41 | 0.092 |
|  | HP TPASS_0619 | **B2S3K9** | 28910.5 | 9.22 | 29045 | 35.53 | Stable | 88.11 | 0.134 |
|  | HP TPASS_0622 | **B2S3L2** | 66777.4 | 9.02 | 73730 | 36.41 | Stable | 96.73 | -0.239 |
|  | HP TPASS_0624 | **B2S3L4** | 52740.6 | 9.41 | 47120 | 39.33 | Stable | 95.55 | -0.118 |
|  | HP TPASS_0625 | **B2S3L5** | 28873.9 | 9.05 | 30495 | 56.54 | Unstable | 70.00 | -0.739 |
|  | HP TPASS_0629 | **B2S3L9** | 31271.8 | 7.75 | 66140 | 38.73 | Stable | 78.28 | -0.197 |
|  | HP TPASS_0636 | **B2S3M5** | 28126.7 | 9.10 | 42565 | 39.86 | Stable | 101.37 | 0.314 |
|  | HP TPASS_0638 | **B2S3M7** | 15394.4 | 8.66 | 7115 | 21.44 | Stable | 143.47 | 1.158 |
|  | HP TPASS_0645 | **B2S3N4** | 6437.6 | 9.21 | 7365 | 4.99 | Stable | 87.41 | 0.348 |
|  | HP TPASS_0646 | **B2S3N5** | 49328.7 | 8.40 | 57215 | 45.46 | Unstable | 91.93 | 0.087 |
|  | HP TPASS_0648 | **B2S3N7** | 77635.4 | 8.94 | 80945 | 37.25 | Stable | 90.65 | -0.331 |
|  | HP TPASS_0651 | **B2S3P0** | 88245.6 | 6.76 | 30300 | 43.05 | Unstable | 104.99 | 0.135 |
|  | HP TPASS_0656 | **B2S3P5** | 3646.2 | 9.49 | 1615 | 5.02 | Stable | 59.09 | -0.676 |
|  | HP TPASS_0661 | **B2S3Q0** | 19235.8 | 7.77 | 14690 | 41.29 | Unstable | 96.35 | -0.152 |
|  | HP TPASS_0665 | **B2S3Q4** | 28955.2 | 9.68 | 20650 | 44.06 | Unstable | 79.56 | -0.748 |
|  | HP TPASS_0666 | **B2S3Q5** | 10661.2 | 6.56 | 14565 | 26.85 | Stable | 93.40 | -0.118 |
|  | HP TPASS_0668 | **B2S3Q7** | 22466.3 | 9.59 | 22710 | 24.20 | Stable | 139.70 | 1.215 |
|  | HP TPASS_0674 | **B2S3R3** | 22440.9 | 11.63 | 15595 | 63.52 | Unstable | 77.48 | -0.497 |
|  | HP TPASS_0675 | **B2S3R4** | 37234.3 | 9.55 | 27305 | 51.30 | Unstable | 101.42 | 0.010 |
|  | HP TPASS_0676 | **B2S3R5** | 10266.3 | 4.65 | 8605 | 36.85 | Stable | 62.72 | -0.818 |
|  | HP TPASS_0677 | **B2S3R6** | 21754.9 | 6.06 | 29825 | 47.99 | Unstable | 90.35 | 0.068 |
|  | HP TPASS_0678 | **B2S3R7** | 34764.0 | 9.38 | 43025 | 64.03 | Unstable | 82.15 | -0.239 |
|  | HP TPASS_0679 | **B2S3R8** | 11586.0 | 9.74 | 8605 | 32.15 | Stable | 133.81 | 1.141 |
|  | HP TPASS_0690 | **B2S3S9** | 22604.3 | 9.07 | 24325 | 33.17 | Stable | 77.20 | -0.115 |
|  | HP TPASS_0691 | **B2S3T0** | 29230.2 | 4.82 | 31900 | 51.13 | Unstable | 110.95 | 0.085 |
|  | HP TPASS_0693 | **B2S3T2** | 47672.6 | 6.35 | 47035 | 49.67 | Unstable | 78.75 | -0.151 |
|  | HP TPASS_0697 | **B2S3T6** | 21439.9 | 8.69 | 23740 | 23.57 | Stable | 97.87 | 0.448 |
|  | HP TPASS_0698 | **B2S3T7** | 18893.7 | 9.00 | 14690 | 26.57 | Stable | 106.63 | 0.511 |
|  | HP TPASS_0699 | **B2S3T8** | 4088.5 | 4.36 | 12490 | 23.16 | Stable | 67.50 | -0.169 |
|  | HP TPASS_0700 | **B2S3T9** | 14204.1 | 4.83 | 4470 | 57.80 | Unstable | 100.54 | -0.101 |
|  | HP TPASS_0702 | **B2S3U1** | 19380.9 | 9.39 | 27055 | 38.82 | Stable | 71.74 | -0.323 |
|  | HP TPASS_0703 | **B2S3U2** | 24715.6 | 10.38 | 18115 | 46.81 | Unstable | 85.88 | -0.227 |
|  | HP TPASS_0706 | **B2S3U5** | 33622.0 | 9.82 | 21360 | 27.97 | Stable | 91.88 | 0.014 |
|  | HP TPASS_0707 | **B2S3U6** | 16909.0 | 10.73 | 28460 | 50.60 | Unstable | 103.14 | 0.466 |
|  | HP TPASS_0708 | **B2S3U7** | 17961.3 | 4.40 | 5960 | 42.46 | Unstable | 100.41 | 0.254 |
|  | HP TPASS_0710 | **B2S3U9** | 72022.6 | 8.54 | 41300 | 31.37 | Stable | 88.16 | -0.367 |
|  | HP TPASS_0711 | **B2S3V0** | 21266.7 | 9.22 | 7575 | 37.78 | Stable | 97.58 | -0.189 |
|  | HP TPASS_0719 | **B2S3V8** | 28809.3 | 8.72 | 8855 | 43.70 | Unstable | 98.36 | 0.102 |
|  | HP TPASS_0723 | **B2S3W2** | 5139.9 | 6.02 | 5500 | 57.26 | Unstable | 66.36 | -0.220 |
|  | HP TPASS_0730 | **B2S3W9** | 39798.5 | 9.66 | 34755 | 44.67 | Unstable | 100.58 | 0.366 |
|  | HP TPASS_0731 | **B2S3X0** | 27118.9 | 7.15 | 21595 | 56.69 | Unstable | 68.14 | -0.464 |
|  | HP TPASS_0733 | **B2S3X2** | 23327.4 | 8.77 | 39100 | 29.87 | Stable | 82.37 | 0.037 |
|  | HP TPASS_0738 | **B2S3X7** | 12276.1 | 6.40 | 18365 | 39.33 | Stable | 93.24 | 0.138 |
|  | HP TPASS_0739 | **B2S3X8** | 44210.6 | 5.84 | 16515 | 38.95 | Stable | 104.50 | -0.015 |
|  | HP TPASS_0740 | **B2S3X9** | 20547.8 | 7.62 | 17460 | 43.57 | Unstable | 94.04 | 0.129 |
|  | HP TPASS_0741 | **B2S3Y0** | 22649.7 | 8.71 | 35410 | 41.64 | Unstable | 85.54 | -0.148 |
|  | HP TPASS_0744 | **B2S3Y3** | 11391.2 | 7.66 | 1740 | 24.92 | Stable | 105.65 | 0.447 |
|  | HP TPASS_0747 | **B2S3Y6** | 37779.2 | 9.23 | 35535 | 32.36 | Stable | 93.78 | -0.122 |
|  | HP TPASS_0749 | **B2S3Y8** | 8606.1 | 10.30 | 8605 | 26.70 | Stable | 69.45 | -0.581 |
|  | HP TPASS_0750 | **B2S3Y9** | 25939.7 | 9.08 | 34045 | 51.27 | Unstable | 84.80 | -0.535 |
|  | HP TPASS_0752 | **B2S3Z1** | 43042.7 | 6.23 | 42860 | 36.18 | Stable | 83.24 | -0.181 |
|  | HP TPASS_0753 | **B2S3Z2** | 10330.1 | 6.01 | 3105 | 45.33 | Unstable | 102.66 | 0.581 |
|  | HP TPASS_0759 | **B2S3Z8** | 4379.2 | 7.80 | 8605 | 13.85 | Stable | 124.87 | 0.895 |
|  | HP TPASS_0761 | **B2S400** | 32411.2 | 10.01 | 21555 | 34.61 | Stable | 90.03 | -0.184 |
|  | HP TPASS_0762 | **B2S401** | 43864.2 | 9.45 | 62965 | 52.43 | Unstable | 81.32 | -0.180 |
|  | HP TPASS_0763 | **B2S402** | 36155.7 | 9.95 | 52745 | 37.17 | Stable | 111.31 | 0.198 |
|  | HP TPASS_0764 | **B2S403** | 43901.4 | 28100 | 28100 | 36.66 | Stable | 97.06 | -0.191 |
|  | HP TPASS_0766 | **B2S405** | 32877.4 | 8.87 | 26150 | 53.32 | Unstable | 89.01 | -0.240 |
|  | HP TPASS_0771 | **B2S410** | 65373.7 | 8.63 | 44140 | 39.21 | Stable | 113.10 | 0.436 |
|  | HP TPASS_0772 | **B2S411** | 30839.4 | 8.59 | 29255 | 24.94 | Stable | 74.49 | -0.360 |
|  | HP TPASS_0776 | **B2S415** | 28894.4 | 9.51 | 28710 | 48.17 | Unstable | 84.34 | 0.006 |
|  | HP TPASS_0777 | **B2S416** | 9018.2 | 6.55 | 17210 | 49.88 | Unstable | 57.84 | -0.99 |
|  | HP TPASS_0781 | **B2S420** | 16678.0 | 7.90 | 7450 | 19.05 | Stable | 98.04 | 0.042 |
|  | HP TPASS_0782 | **B2S421** | 15652.8 | 7.80 | 17085 | 28.45 | Stable | 85.03 | 0.063 |
|  | HP TPASS_0783 | **B2S422** | 41681.0 | 6.11 | 33390 | 27.32 | Stable | 91.78 | 0.166 |
|  | HP TPASS_0784 | **B2S423** | 22511.5 | 9.03 | 24325 | 34.68 | Stable | 79.90 | -0.159 |
|  | HP TPASS_0785 | **B2S424** | 26084.8 | 9.42 | 23045 | 30.53 | Stable | 83.07 | -0.353 |
|  | HP TPASS_0787 | **B2S426** | 15068.1 | 8.48 | 11960 | 38.25 | Stable | 119.86 | 1.058 |
|  | HP TPASS_0788 | **B2S427** | 33638.4 | 7.76 | 48150 | 36.87 | Stable | 88.45 | -0.219 |
|  | HP TPASS_0789 | **B2S428** | 29040.4 | 9.12 | 33140 | 27.42 | Stable | 77.79 | -0.308 |
|  | HP TPASS_0791 | **B2S430** | 8540.1 | 7.76 | 1615 | 36.23 | Stable | 130.00 | 0.448 |
|  | HP TPASS_0793 | **B2S432** | 63070.6 | 9.68 | 75875 | 42.85 | Unstable | 79.28 | -0.672 |
|  | HP TPASS_0795 | **B2S434** | 5769.6 | 10.69 | 1490 | 29.18 | Stable | 80.77 | 0.140 |
|  | HP TPASS_0796 | **B2S435** | 39064.1 | 8.06 | 28920 | 28.09 | Stable | 101.88 | 0.196 |
|  | HP TPASS_0799 | **B2S438** | 5795.7 | 10.38 | 6990 | 63.59 | Unstable | 56.60 | -1.000 |
|  | HP TPASS_0802 | **B2S441** | 12261.8 | 9.98 | 11710 | 44.79 | Unstable | 47.61 | -0.883 |
|  | HP TPASS_0803 | **B2S442** | 44577.5 | 8.37 | 28600 | 34.42 | Stable | 104.68 | 0.072 |
|  | HP TPASS_0811 | **B2S450** | 4658.4 | 8.00 | 8480 | 19.07 | Stable | 85.61 | 0.805 |
|  | HP TPASS_0813 | **B2S451** | 48022.7 | 10.05 | 38890 | 47.45 | Unstable | 95.58 | -0.031 |
|  | HP TPASS_0815 | **B2S453** | 36194.1 | 10.12 | 18380 | 40.80 | Unstable | 99.05 | 0.033 |
|  | HP TPASS_0816 | **B2S454** | 24379.1 | 8.35 | 22710 | 34.92 | Stable | 99.28 | 0.209 |
|  | HP TPASS_0818 | **B2S456** | 5572.3 | 9.14 | 13075 | 43.72 | Unstable | 62.60 | -0.026 |
|  | HP TPASS_0820 | **B2S458** | 28932.9 | 9.39 | 41620 | 37.35 | Stable | 78.28 | -0.250 |
|  | HP TPASS_0822 | **B2S460** | 33249.4 | 9.12 | 29700 | 41.37 | Unstable | 106.88 | 0.321 |
|  | HP TPASS_0825 | **B2S463** | 7260.4 | 11.88 | 1615 | 64.13 | Unstable | 64.10 | -0.936 |
|  | HP TPASS_0826 | **B2S464** | 30332.4 | 9.25 | 29910 | 36.66 | Stable | 118.24 | 0.300 |
|  | HP TPASS_0827 | **B2S465** | 47225.8 | 8.90 | 42775 | 48.78 | Unstable | 98.82 | 0.023 |
|  | HP TPASS_0829 | **B2S467** | 33779.4 | 8.77 | 35675 | 53.63 | Unstable | 76.33 | -0.527 |
|  | HP TPASS_0832 | **B2S470** | 29157.1 | 7.74 | 32095 | 55.11 | Unstable | 84.65 | 0.017 |
|  | HP TPASS_0833 | **B2S471** | 26580.6 | 9.68 | 19730 | 30.73 | Stable | 92.75 | -0.087 |
|  | HP TPASS_0836 | **B2S474** | 31035.7 | 9.67 | 27430 | 56.26 | Unstable | 82.66 | -0.221 |
|  | HP TPASS_0839 | **B2S477** | 37364.6 | 9.52 | 45880 | 35.99 | Stable | 85.46 | -0.229 |
|  | HP TPASS_0840 | **B2S478** | 82280.9 | 8.93 | 51395 | 33.16 | Stable | 111.26 | 0.453 |
|  | HP TPASS_0845 | **B2S483** | 21641.8 | 8.97 | 15845 | 51.11 | Unstable | 93.11 | -0.169 |
|  | HP TPASS_0846 | **B2S484** | 12145.9 | 5.03 | 4470 | 54.87 | Unstable | 110.83 | -0.119 |
|  | HP TPASS_0847 | **B2S485** | 12511.4 | 5.07 | 1490 | 37.27 | Stable | 101.98 | -0.356 |
|  | HP TPASS_0851 | **B2S489** | 81940.6 | 8.28 | 93835 | 40.21 | Unstable | 90.03 | -0.241 |
|  | HP TPASS_0854 | **B2S492** | 172928.5 | 6.26 | 192490 | 36.69 | Stable | 87.78 | -0.180 |
|  | HP TPASS_0855 | **B2S493** | 124700.9 | 8.58 | 88255 | 44.98 | Unstable | 87.23 | -0.385 |
|  | HP TPASS_0856 | **B2S494** | 34054.6 | 8.81 | 26315 | 33.66 | Stable | 65.48 | 0.018 |
|  | HP TPASS_0857 | **B2S495** | 11607.2 | 11.01 | 7115 | 61.66 | Unstable | 78.49 | -0.442 |
|  | HP TPASS_0858 | **B2S496** | 43417.5 | 9.00 | 36660 | 27.06 | Stable | 67.84 | -0.063 |
|  | HP TPASS_0859 | **B2S497** | 24339.9 | 11.71 | 7575 | 30.18 | Stable | 82.84 | 0.069 |
|  | HP TPASS_0860 | **B2S498** | 26315.9 | 9.95 | 49390 | 39.60 | Stable | 78.50 | -0.192 |
|  | HP TPASS_0864 | **B2S4A2** | 58514.5 | 10.71 | 30870 | 36.72 | Stable | 90.77 | -0.003 |
|  | HP TPASS_0865 | **B2S4A3** | 52609.7 | 10.01 | 65695 | 34.35 | Stable | 78.04 | -0.159 |
|  | HP TPASS_0867 | **B2S4A4** | 7215.3 | 9.61 | 25690 | 24.83 | Stable | 81.64 | -0.207 |
|  | HP TPASS_0869 | **B2S4A6** | 8735.4 | 10.92 | 19730 | 43.68 | Unstable | 78.85 | 0.237 |
|  | HP TPASS_0871 | **B2S4A8** | 5998.0 | 10.24 | 5960 | 47.98 | Unstable | 66.42 | -0.642 |
|  | HP TPASS_0873 | **B2S4B0** | 21701.6 | 4.65 | 7575 | 31.20 | Stable | 109.75 | 0.063 |
|  | HP TPASS_0874 | **B2S4B1** | 10568.3 | 9.16 | 7450 | 33.08 | Stable | 109.12 | -0.112 |
|  | HP TPASS_0875 | **B2S4B2** | 14671.8 | 4.96 | 18700 | 33.81 | Stable | 106.81 | 0.319 |
|  | HP TPASS_0876 | **B2S4B3** | 23647.3 | 9.28 | 10345 | 30.60 | Stable | 89.19 | 0.111 |
|  | HP TPASS_0877 | **B2S4B4** | 41845.4 | 5.28 | 46090 | 38.05 | Stable | 88.22 | -0.342 |
|  | HP TPASS_0878 | **B2S4B5** | 37312.7 | 9.54 | 56630 | 43.77 | Unstable | 74.37 | -0.337 |
|  | HP TPASS_0879 | **B2S4B6** | 56138.3 | 8.03 | 66280 | 44.12 | Unstable | 101.60 | 0.103 |
|  | HP TPASS_0882 | **B2S4B9** | 55969.1 | 6.27 | 32695 | 42.15 | Unstable | 99.68 | -0.148 |
|  | HP TPASS_0883 | **B2S4C0** | 42122.8 | 8.57 | 60445 | 31.66 | Stable | 107.56 | 0.539 |
|  | HP TPASS_0884 | **B2S4C1** | 40351.8 | 9.60 | 34755 | 32.81 | Stable | 122.93 | 0.623 |
|  | HP TPASS_0893 | **B2S4D0** | 16707.3 | 9.04 | 14230 | 34.90 | Stable | 101.79 | 0.377 |
|  | HP TPASS_0894 | **B2S4D1** | 38411.4 | 6.48 | 51005 | 39.46 | Stable | 81.74 | -0.419 |
|  | HP TPASS_0895 | **B2S4D2** | 18863.0 | 9.47 | 18575 | 35.61 | Stable | 96.25 | 0.033 |
|  | HP TPASS_0896 | **B2S4D3** | 5728.6 | 10.89 | 5960 | 56.64 | Unstable | 85.80 | -0.134 |
|  | HP TPASS_0899 | **B2S4D6** | 22122.4 | 8.99 | 23295 | 27.25 | Stable | 77.95 | -0.062 |
|  | HP TPASS_0900 | **B2S4D7** | 89504.3 | 8.86 | 97635 | 54.91 | Unstable | 84.05 | -0.096 |
|  | HP TPASS_0901 | **B2S4D8** | 51384.3 | 9.65 | 80050 | 31.56 | Stable | 115.34 | 0.692 |
|  | HP TPASS_0904 | **B2S4E1** | 9659.2 | 9.57 | 4595 | 81.53 | Unstable | 82.29 | -0.377 |
|  | HP TPASS_0906 | **B2S4E3** | 8686.0 | 6.08 | 2980 | 36.78 | Stable | 115.88 | 0.076 |
|  | HP TPASS_0907 | **B2S4E4** | 18945.1 | 8.21 | 10345 | 44.09 | Unstable | 107.53 | 0.293 |
|  | HP TPASS_0910 | **B2S4E7** | 33814.2 | 10.04 | 26845 | 40.37 | Unstable | 98.53 | 0.050 |
|  | HP TPASS_0911 | **B2S4E8** | 8995.5 | 8.66 | 4595 | 35.38 | Stable | 110.60 | 0.334 |
|  | HP TPASS_0912 | **B2S4E9** | 49018.3 | 5.72 | 37735 | 40.42 | Unstable | 98.61 | -0.061 |
|  | HP TPASS_0913 | **B2S4F0** | 14585.9 | 9.33 | 17085 | 52.37 | Unstable | 96.83 | -0.163 |
|  | HP TPASS_0914 | **B2S4F1** | 14572.9 | 9.08 | 14355 | 50.43 | Unstable | 82.11 | 0.216 |
|  | HP TPASS_0915 | **B2S4F2** | 50768.4 | 8.62 | 42540 | 41.21 | Unstable | 97.34 | -0.255 |
|  | HP TPASS_0916 | **B2S4F3** | 4801.4 | 7.80 | 8605 | 71.67 | Unstable | 81.19 | -0.498 |
|  | HP TPASS_0918 | **B2S4F5** | 26922.3 | 9.90 | 28460 | 42.81 | Unstable | 99.65 | 0.229 |
|  | HP TPASS_0920 | **B2S4F7** | 90162.5 | 5.86 | 109095 | 43.56 | Unstable | 91.77 | -0.154 |
|  | HP TPASS_0922 | **B2S4F9** | 32897.5 | 11.49 | 12460 | 59.19 | Unstable | 72.59 | -0.385 |
|  | HP TPASS_0923 | **B2S4G0** | 36117.4 | 8.13 | 29755 | 22.92 | Stable | 78.20 | -0.122 |
|  | HP TPASS_0927 | **B2S4G4** | 22215.0 | 9.98 | 10805 | 54.09 | Unstable | 63.27 | -0.777 |
|  | HP TPASS_0928 | **B2S4G5** | 27471.3 | 9.89 | 20525 | 43.60 | Unstable | 71.83 | -0.400 |
|  | HP TPASS_0929 | **B2S4G6** | 28192.5 | 5.14 | 18450 | 45.95 | Unstable | 79.30 | -0.211 |
|  | HP TPASS_0930 | **B2S4G7** | 64724.0 | 9.31 | 50155 | 43.24 | Unstable | 98.27 | 0.004 |
|  | HP TPASS_0931 | **B2S4G8** | 55389.7 | 8.76 | 107385 | 43.06 | Unstable | 84.85 | -0.287 |
|  | HP TPASS_0932 | **B2S4G9** | 3234.7 | 6.80 | 125 | 86.00 | Unstable | 90.67 | 0.253 |
|  | HP TPASS_0937 | **B2S4H4** | 22799.2 | 8.75 | 4970 | 28.23 | Stable | 99.34 | 0.182 |
|  | HP TPASS_0938 | **B2S4H5** | 30823.5 | 8.82 | 29045 | 50.52 | Unstable | 87.50 | -0.119 |
|  | HP TPASS_0940 | **B2S4H7** | 4929.6 | 8.63 | 1615 | 61.16 | Unstable | 42.61 | -0.385 |
|  | HP TPASS_0941 | **B2S4H8** | 19647.4 | 5.18 | 21555 | 30.84 | Stable | 95.76 | -0.112 |
|  | HP TPASS_0942 | **B2S4H9** | 17417.7 | 4.84 | 4595 | 55.34 | Unstable | 105.13 | -0.405 |
|  | HP TPASS_0944 | **B2S4I1** | 43479.3 | 9.62 | 49320 | 37.45 | Stable | 93.77 | -0.117 |
|  | HP TPASS_0950 | **B2S4I7** | 10151.9 | 11.15 | 5875 | 45.56 | Unstable | 76.81 | 0.244 |
|  | HP TPASS_0954 | **B2S4J1** | 54661.1 | 8.83 | 70500 | 39.84 | Stable | 83.81 | -0.402 |
|  | HP TPASS_0955 | **B2S4J2** | 10660.7 | 11.93 | 16500 | 54.78 | Unstable | 108.44 | 0.006 |
|  | HP TPASS_0956 | **B2S4J3** | 36005.0 | 5.85 | 43360 | 39.51 | Stable | 88.61 | -0.041 |
|  | HP TPASS_0959 | **B2S4J6** | 13902.7 | 8.89 | 5960 | 28.22 | Stable | 74.26 | -0.273 |
|  | HP TPASS_0962 | **B2S4J9** | 44605.0 | 8.77 | 28670 | 39.43 | Stable | 109.32 | 0.421 |
|  | HP TPASS_0963 | **B2S4K0** | 43578.6 | 7.22 | 17545 | 34.57 | Stable | 115.28 | 0.508 |
|  | HP TPASS_0966 | **B2S4K3** | 60246.9 | 9.19 | 69245 | 44.50 | Unstable | 89.96 | -0.248 |
|  | HP TPASS_0967 | **B2S4K4** | 56597.3 | 7.80 | 52955 | 38.96 | Stable | 79.79 | -0.417 |
|  | HP TPASS_0968 | **B2S4K5** | 60120.5 | 9.30 | 31120 | 43.57 | Unstable | 83.72 | -0.311 |
|  | HP TPASS_0969 | **B2S4K6** | 59650.9 | 8.34 | 45645 | 31.15 | Stable | 76.25 | -0.460 |
|  | HP TPASS_0970 | **B2S4K7** | 4181.8 | 11.71 | 0 | 20.26 | Stable | 71.84 | 0.021 |
|  | HP TPASS_0972 | **B2S4K9** | 48455.5 | 9.36 | 91955 | 31.66 | Stable | 96.50 | 0.494 |
|  | HP TPASS_0974 | **B2S4L1** | 10221.6 | 4.96 | 4470 | 35.84 | Stable | 98.82 | -0.238 |
|  | HP TPASS_0975 | **B2S4L2** | 29532.1 | 9.53 | 6335 | 39.80 | Stable | 101.06 | 0.085 |
|  | HP TPASS_0976 | **B2S4L3** | 49187.5 | 9.11 | 30620 | 47.23 | Unstable | 102.05 | 0.128 |
|  | HP TPASS_0977 | **B2S4L4** | 30882.2 | 6.70 | 21680 | 44.31 | Unstable | 93.57 | 0.051 |
|  | HP TPASS_0979 | **B2S4L6** | 28974.1 | 5.67 | 24660 | 54.70 | Unstable | 92.61 | -0.159 |
|  | HP TPASS_0983 | **B2S4M0** | 27194.1 | 9.87 | 31985 | 32.65 | Stable | 71.64 | -0.484 |
|  | HP TPASS_0986 | **B2S4M3** | 32365.3 | 9.61 | 30410 | 30.59 | Stable | 104.83 | 0.632 |
|  | HP TPASS_0987 | **B2S4M4** | 6478.5 | 9.74 | 4470 | 42.36 | Unstable | 99.11 | -0.164 |
|  | HP TPASS_0988 | **B2S4M5** | 30911.9 | 9.18 | 18615 | 38.75 | Stable | 107.34 | 0.413 |
|  | HP TPASS_0990 | **B2S4M7** | 116827.0 | 5.22 | 111635 | 50.44 | Unstable | 73.16 | -0.659 |
|  | HP TPASS_0992 | **B2S4M9** | 10147.4 | 9.25 | 9970 | 40.32 | Unstable | 83.37 | -0.152 |
|  | HP TPASS_0994 | **B2S4N1** | 29533.8 | 7.59 | 26525 | 48.11 | Unstable | 89.00 | -0.151 |
|  | HP TPASS_0996 | **B2S4N3** | 61485.6 | 7.56 | 88615 | 48.20 | Unstable | 80.48 | -0.520 |
|  | HP TPASS_1000 | **B2S4N7** | 25428.6 | 6.45 | 49305 | 44.14 | Unstable | 63.00 | -0.524 |
|  | HP TPASS_1001 | **B2S4N8** | 43439.6 | 7.20 | 43930 | 40.96 | Unstable | 87.84 | -0.105 |
|  | HP TPASS_1002 | **B2S4N9** | 24784.5 | 9.10 | 16515 | 32.43 | Stable | 89.05 | -0.045 |
|  | HP TPASS_1003 | **B2S4P0** | 37828.4 | 10.23 | 45630 | 41.95 | Unstable | 115.68 | 0.631 |
|  | HP TPASS_1014 | **B2S4Q1** | 71126.9 | 9.49 | 77155 | 43.94 | Unstable | 85.81 | -0.173 |
|  | HP TPASS_1018 | **B2S4Q5** | 57070.9 | 6.06 | 33265 | 41.51 | Unstable | 97.14 | -0.466 |
|  | HP TPASS_1029 | **B2S4R6** | 25186.6 | 9.70 | 9065 | 45.70 | Unstable | 78.51 | -0.364 |
|  | HP TPASS_1030 | **B2S4R7** | 12799.9 | 11.72 | 7115 | 58.21 | Unstable | 68.07 | -0.448 |
|  | HP TPASS_1032 | **B2S4R9** | 15441.6 | 7.55 | 21470 | 35.25 | Stable | 80.07 | -0.092 |
|  | HP TPASS_1033 | **B2S4S0** | 33568.8 | 9.52 | 27555 | 36.92 | Stable | 94.73 | 0.225 |
|  | HP TPASS_1034 | **B2S4S1** | 35537.3 | 9.98 | 35325 | 34.79 | Stable | 134.49 | 0.905 |
