# Supplementary material for: Identification of Functional Candidates amongst Hypothetical Proteins of Treponema pallidum ssp. pallidum
Source: PLoS One. 2015 Apr 20;10(4):e0124177. doi: 10.1371/journal.pone.0124177 (PMC4403809; doi:10.1371/journal.pone.0124177)
Supplement: S2 Table — (DOC) [file pone.0124177.s002.doc]

| **Table S2: List of predicted subcellular localizations of 444 HPs from *T. pallidum ssp. pallidum*** | | | | | | | | | |
| --- | --- | --- | --- | --- | --- | --- | --- | --- | --- |
| **S.**  **No.** | **Protein name** | **Uniprot ID** | **Sub-cellular localization** | | | **Signal Peptide**  **(SignalP)** | **Secretory Protein****(SecretomeP)** | **Tran membrane helices prediction** | |
|  |  |  |  |  |  |  |  | **HMMTOP** | **TMHMM** |
|  |  |  | **PSORT B** | **PSLpred** | **CELLO** |  |  |  |  |
|  | HP TPASS_0004 | **B2S1V4** | Unknown | Periplasmic | Cytoplasmic | No | Yes | No | No |
|  | HP TPASS_0008 | **B2S1V7** | Unknown | Cytoplasmic | Cytoplasmic | No | No | No | No |
|  | HP TPASS_0010 | **B2S1V8** | Unknown | Extracellular | Cytoplasmic | No | No | No | No |
|  | HP TPASS_0012 | **B2S1W0** | Unknown | Extracellular | Cytoplasmic | No | No | 1 TMM Helix | No |
|  | HP TPASS_0013 | **B2S1W1** | Cytoplasmic | Cytoplasmic | Cytoplasmic | Yes | No | 1 TMM Helix | No |
|  | HP TPASS_0014 | **B2S1W2** | Cytoplasmic | Cytoplasmic | Cytoplasmic | No | No | No | No |
|  | HP TPASS_0017 | **B2S1W5** | Cytoplasmic | Cytoplasmic | Cytoplasmic | No | No | No | No |
|  | HP TPASS_0021 | **B2S1W9** | Unknown | Periplasmic | Cytoplasmic | No | No | No | No |
|  | HP TPASS_0022 | **B2S1X0** | Cytoplasmic | Cytoplasmic | Cytoplasmic | No | No | No | No |
|  | HP TPASS_0024 | **B2S1X2** | Cytoplasmic | Cytoplasmic | Cytoplasmic | No | No | No | No |
|  | HP TPASS_0025 | **B2S1X3** | Cytoplasmic | Periplasmic | Cytoplasmic | No | No | No | No |
|  | HP TPASS_0031 | **B2S1X9** | Cytoplasmic Membrane | Periplasmic | Cytoplasmic | No | No | 1 TMM Helix | 1 TMM Helix |
|  | HP TPASS_0033 | **B2S1Y1** | Cytoplasmic Membrane | Inner Membrane | Inner Membrane | No | No | 5 TMM Helices | 5 TMM Helices |
|  | HP TPASS_0039 | **B2S1Y7** | Unknown | Extracellular | Cytoplasmic | No | Yes | No | No |
|  | HP TPASS_0041 | **B2S1Y9** | Unknown | Extracellular | Cytoplasmic | No | Yes | No | No |
|  | HP TPASS_0042 | **B2S1Z0** | Unknown | Extracellular | Cytoplasmic | No | No | 1 TMM Helix | 1 TMM Helix |
|  | HP TPASS_0046 | **B2S1Z4** | Cytoplasmic | Cytoplasmic | Cytoplasmic | No | No | No | No |
|  | HP TPASS_0047 | **B2S1Z5** | Cytoplasmic | Extracellular | Cytoplasmic | No | No | No | No |
|  | HP TPASS_0048 | **B2S1Z6** | Cytoplasmic | Cytoplasmic | Cytoplasmic | No | No | 1 TMM Helix | No |
|  | HP TPASS_0049 | **B2S1Z7** | Outer Membrane | Outer Membrane | Extracellular | No | Yes | No | No |
|  | HP TPASS_0050 | **B2S1Z8** | Unknown | Inner Membrane | Cytoplasmic | No | No | 1 TMM Helix | No |
|  | HP TPASS_0054 | **B2S202** | Cytoplasmic | Cytoplasmic | Cytoplasmic | No | No | No | No |
|  | HP TPASS_0055 | **B2S203** | Cytoplasmic Membrane | Cytoplasmic | Inner Membrane | No | No | 2 TMM Helices | 2 TMM Helices |
|  | HP TPASS_0059 | **B2S207** | Unknown | Extracellular | Cytoplasmic | Yes | Yes | No | No |
|  | HP TPASS_0064 | **B2S212** | Cytoplasmic | Cytoplasmic | Cytoplasmic | No | No | 1 TMM Helix | No |
|  | HP TPASS_0065 | **B2S213** | Cytoplasmic | Cytoplasmic | Cytoplasmic | No | No | 1 TMM Helix | No |
|  | HP TPASS_0066 | **B2S214** | Unknown | Extracellular | Cytoplasmic | No | No | No | No |
|  | HP TPASS_0067 | **B2S215** | Cytoplasmic | Cytoplasmic | Cytoplasmic | No | No | No | No |
|  | HP TPASS_0068 | **B2S216** | Cytoplasmic | Cytoplasmic | Cytoplasmic | No | No | No | No |
|  | HP TPASS_0069 | **B2S217** | Unknown | Cytoplasmic | Cytoplasmic | No | No | No | No |
|  | HP TPASS_0070 | **B2S218** | Cytoplasmic Membrane | Inner Membrane | Inner Membrane | No | No | 4 TMM Helices | 3 TMM Helices |
|  | HP TPASS_0072 | **B2S220** | Cytoplasmic | Extracellular | Cytoplasmic | No | No | No | No |
|  | HP TPASS_0073 | **B2S221** | Cytoplasmic | Cytoplasmic | Cytoplasmic | No | No | No | No |
|  | HP TPASS_0079 | **B2S227** | Cytoplasmic | Cytoplasmic | Cytoplasmic | No | No | No | No |
|  | HP TPASS_0081 | **B2S229** | Unknown | Cytoplasmic | Cytoplasmic | No | No | No | No |
|  | HP TPASS_0083 | **B2S231** | Cytoplasmic | Periplasmic | Periplasmic | No | Yes | No | No |
|  | HP TPASS_0084 | **B2S232** | Unknown | Extracellular | Cytoplasmic | No | No | No | No |
|  | HP TPASS_0086 | **B2S234** | Cytoplasmic | Cytoplasmic | Cytoplasmic | No | No | 1 TMM Helix | No |
|  | HP TPASS_0087 | **B2S235** | Unknown | Periplasmic | Periplasmic | No | No | 1 TMM Helix | No |
|  | HP TPASS_0088 | **B2S236** | Unknown | Extracellular | Cytoplasmic | No | No | No | No |
|  | HP TPASS_0093 | **B2S241** | Unknown | Cytoplasmic | Periplasmic | No | No | No | No |
|  | HP TPASS_0095 | **B2S243** | Cytoplasmic | Cytoplasmic | Cytoplasmic | No | No | No | No |
|  | HP TPASS_0110 | **B2S258** | Unknown | Cytoplasmic | Cytoplasmic | No | No | No | No |
|  | HP TPASS_0118 | **B2S266** | Cytoplasmic | Cytoplasmic | Cytoplasmic | No | No | No | No |
|  | HP TPASS_0121 | **B2S269** | Cytoplasmic | Cytoplasmic | Cytoplasmic | No | No | No | No |
|  | HP TPASS_0123 | **B2S271** | Cytoplasmic | Cytoplasmic | Cytoplasmic | No | No | No | No |
|  | HP TPASS_0126 | **B2S274** | Unknown | Extracellular | Extracellular | No | Yes | 3 TMM Helices | 2 TMM Helices |
|  | HP TPASS_0127 | **B2S275** | Unknown | Periplasmic | Periplasmic | Yes | Yes | 1 TMM Helix | No |
|  | HP TPASS_0128 | **B2S276** | Unknown | Periplasmic | Cytoplasmic | No | Yes | No | No |
|  | HP TPASS_0129 | **B2S277** | Unknown | Periplasmic | Periplasmic | No | Yes | No | No |
|  | HP TPASS_0130 | **B2S278** | Unknown | Extracellular | Periplasmic | No | Yes | No | No |
|  | HP TPASS_0132 | **B2S280** | Unknown | Extracellular | Cytoplasmic | No | Yes | No | No |
|  | HP TPASS_0133 | **B2S281** | Unknown | Extracellular | Extracellular | No | Yes | 1 TMM Helix | No |
|  | HP TPASS_0134 | **B2S282** | Unknown | Extracellular | Extracellular | No | Yes | 1 TMM Helix | No |
|  | HP TPASS_0135 | **B2S283** | Unknown | Cytoplasmic | Periplasmic | No | Yes | 1 TMM Helix | No |
|  | HP TPASS_0136 | **B2S284** | Unknown | Extracellular | Extracellular | Yes | Yes | 1 TMM Helix | 1 TMM Helix |
|  | HP TPASS_0137 | **B2S285** | Unknown | Extracellular | Cytoplasmic | No | No | No | No |
|  | HP TPASS_0138 | **B2S286** | Cytoplasmic Membrane | Cytoplasmic | Inner Membrane | No | No | 4 TMM Helices | 2 TMM Helices |
|  | HP TPASS_0139 | **B2S287** | Cytoplasmic | Inner Membrane | Cytoplasmic | No | No | No | No |
|  | HP TPASS_0148 | **B2S295** | Cytoplasmic Membrane | Inner Membrane | Inner Membrane | No | No | 6 TMM Helices | 6 TMM Helices |
|  | HP TPASS_0149 | **B2S296** | Cytoplasmic Membrane | Inner Membrane | Inner Membrane | No | No | 4 TMM Helices | 4 TMM Helices |
|  | HP TPASS_0150 | **B2S297** | Unknown | Extracellular | Periplasmic | No | No | 2 TMM Helices | 1 TMM Helix |
|  | HP TPASS_0151 | **B2S298** | Cytoplasmic Membrane | Inner Membrane | Inner Membrane | No | No | 11 TMM Helix | 7 TMM Helices |
|  | HP TPASS_0153 | **B2S2A0** | Cytoplasmic Membrane | Cytoplasmic | Cytoplasmic | No | No | 4 TMM Helices | No |
|  | HP TPASS_0154 | **B2S2A1** | Cytoplasmic | Periplasmic | Cytoplasmic | No | Yes | 1 TMM Helix | No |
|  | HP TPASS_0156 | **B2S2A3** | Cytoplasmic | Cytoplasmic | Cytoplasmic | No | No | No | No |
|  | HP TPASS_0157 | **B2S2A4** | Cytoplasmic | Cytoplasmic | Cytoplasmic | No | No | No | No |
|  | HP TPASS_0158 | **B2S2A5** | Cytoplasmic | Cytoplasmic | Cytoplasmic | No | No | No | No |
|  | HP TPASS_0159 | **B2S2A6** | Cytoplasmic | Cytoplasmic | Cytoplasmic | Yes | No | No | No |
|  | HP TPASS_0161 | **B2S2A8** | Unknown | Extracellular | Cytoplasmic | No | Yes | No | No |
|  | HP TPASS_0169 | **B2S2B6** | Unknown | Extracellular | Cytoplasmic | No | No | No | No |
|  | HP TPASS_0172 | **B2S2B9** | Unknown | Cytoplasmic | Cytoplasmic | No | No | No | No |
|  | HP TPASS_0173 | **B2S2C0** | Cytoplasmic Membrane | Inner Membrane | Inner Membrane | No | No | 3 TMM Helices | 2 TMM Helices |
|  | HP TPASS_0174 | **B2S2C1** | Unknown | Cytoplasmic | Inner Membrane | No | No | 1 TMM Helix | 1 TMM Helix |
|  | HP TPASS_0175 | **B2S2C2** | Unknown | Cytoplasmic | Cytoplasmic | No | No | No | No |
|  | HP TPASS_0176 | **B2S2C3** | Unknown | Extracellular | Cytoplasmic | No | No | No | No |
|  | HP TPASS_0177 | **B2S2C4** | Unknown | Cytoplasmic | Cytoplasmic | No | No | No | No |
|  | HP TPASS_0178 | **B2S2C5** | Unknown | Cytoplasmic | Cytoplasmic | No | No | No | No |
|  | HP TPASS_0179 | **B2S2C6** | Extracellular | Cytoplasmic | Cytoplasmic | No | Yes | No | No |
|  | HP TPASS_0180 | **B2S2C7** | Unknown | Extracellular | Cytoplasmic | No | Yes | No | No |
|  | HP TPASS_0181 | **B2S2C8** | Unknown | Inner Membrane | Cytoplasmic | No | No | 2 TMM Helices | 2 TMM Helices |
|  | HP TPASS_0182 | **B2S2C9** | Cytoplasmic | Cytoplasmic | Cytoplasmic | No | No | No | No |
|  | HP TPASS_0183 | **B2S2D0** | Unknown | Cytoplasmic | Cytoplasmic | Yes | No | No | No |
|  | HP TPASS_0214 | **B2S2G2** | Unknown | Extracellular | Cytoplasmic | No | No | No | No |
|  | HP TPASS_0222 | **B2S2G9** | Unknown | Cytoplasmic | Cytoplasmic | Yes | No | 2 TMM Helices | 1 TMM Helix |
|  | HP TPASS_0223 | **B2S2H0** | Cytoplasmic | Cytoplasmic | Cytoplasmic | No | No | 1 TMM Helix | No |
|  | HP TPASS_0224 | **B2S2H1** | Unknown | Extracellular | Cytoplasmic | No | No | No | No |
|  | HP TPASS_0226 | **B2S2H3** | Cytoplasmic Membrane | Inner Membrane | Inner Membrane | No | No | 4 TMM Helices | 4 TMM Helices |
|  | HP TPASS_0231 | **B2S2H8** | Cytoplasmic | Cytoplasmic | Cytoplasmic | No | No | No | No |
|  | HP TPASS_0232 | **B2S2H9** | Unknown | Extracellular | Cytoplasmic | No | No | 1 TMM Helix | No |
|  | HP TPASS_0245 | **B2S2J2** | Cytoplasmic | Cytoplasmic | Cytoplasmic | No | No | 1 TMM Helix | No |
|  | HP TPASS_0246 | **B2S2J3** | Cytoplasmic | Cytoplasmic | Cytoplasmic | No | No | No | No |
|  | HP TPASS_0248 | **B2S2J5** | Unknown | Cytoplasmic | Cytoplasmic | No | No | No | No |
|  | HP TPASS_0250a | **B2S2J7** | Cytoplasmic Membrane | Extracellular | Cytoplasmic | No | No | 2 TMM Helices | 1 TMM Helix |
|  | HP TPASS_0253 | **B2S2K1** | Cytoplasmic | Cytoplasmic | Cytoplasmic | No | No | 1 TMM Helix | No |
|  | HP TPASS_0258 | **B2S2K6** | Cytoplasmic | Cytoplasmic | Cytoplasmic | No | No | No | No |
|  | HP TPASS_0259 | **B2S2K7** | Cytoplasmic | Cytoplasmic | Cytoplasmic | No | No | No | No |
|  | HP TPASS_0260 | **B2S2K8** | Cytoplasmic | Cytoplasmic | Cytoplasmic | No | No | 1 TMM Helix | 1 TMM Helix |
|  | HP TPASS_0263 | **B2S2L1** | Cytoplasmic | Cytoplasmic | Cytoplasmic | No | No | No | No |
|  | HP TPASS_0266 | **B2S2L4** | Unknown | Periplasmic | Cytoplasmic | No | No | No | No |
|  | HP TPASS_0267 | **B2S2L5** | Cytoplasmic | Cytoplasmic | Cytoplasmic | No | No | No | No |
|  | HP TPASS_0268 | **B2S2L6** | Unknown | Cytoplasmic | Cytoplasmic | No | No | 1 TMM Helix | No |
|  | HP TPASS_0269 | **B2S2L7** | Cytoplasmic | Cytoplasmic | Cytoplasmic | No | No | No | No |
|  | HP TPASS_0273 | **B2S2M1** | Cytoplasmic Membrane | Inner Membrane | Inner Membrane | No | No | 5 TMM Helices | 4 TMM Helices |
|  | HP TPASS_0278 | **B2S2M6** | Unknown | Extracellular | Cytoplasmic | No | No | No | No |
|  | HP TPASS_0280 | **B2S2M8** | Unknown | Extracellular | Cytoplasmic | No | Yes | No | No |
|  | HP TPASS_0281 | **B2S2M9** | Unknown | Periplasmic | Cytoplasmic | No | No | No | No |
|  | HP TPASS_0282 | **B2S2N0** | Unknown | Cytoplasmic | Cytoplasmic | No | No | 1 TMM Helix | 1 TMM Helix |
|  | HP TPASS_0284 | **B2S2N2** | Unknown | Cytoplasmic | Cytoplasmic | No | No | 1 TMM Helix | No |
|  | HP TPASS_0285 | **B2S2N3** | Cytoplasmic | Cytoplasmic | Cytoplasmic | No | No | No | No |
|  | HP TPASS_0286 | **B2S2N4** | Unknown | Cytoplasmic | Inner Membrane | No | No | 2 TMM Helices | No |
|  | HP TPASS_0287 | **B2S2N5** | Unknown | Extracellular | Cytoplasmic | No | No | No | No |
|  | HP TPASS_0289 | **B2S2N7** | Cytoplasmic | Cytoplasmic | Cytoplasmic | No | No | 1 TMM Helix | No |
|  | HP TPASS_0290 | **B2S2N8** | Cytoplasmic | Cytoplasmic | Cytoplasmic | No | No | No | No |
|  | HP TPASS_0291 | **B2S2N9** | Cytoplasmic | Cytoplasmic | Cytoplasmic | No | No | No | No |
|  | HP TPASS_0293 | **B2S2P1** | Unknown | Periplasmic | Cytoplasmic | No | No | No | No |
|  | HP TPASS_0296 | **B2S2P4** | Unknown | Cytoplasmic | Cytoplasmic | No | No | 1 TMM Helix | No |
|  | HP TPASS_0297 | **B2S2P5** | Unknown | Extracellular | Periplasmic | No | Yes | 1 TMM Helix | 1 TMM Helix |
|  | HP TPASS_0299 | **B2S2P7** | Unknown | Extracellular | Cytoplasmic | No | Yes | No | No |
|  | HP TPASS_0301 | **B2S2P9** | Cytoplasmic Membrane | Inner Membrane | Inner Membrane | No | No | 9 TMM Helices | 10 TMM Helices |
|  | HP TPASS_0302 | **B2S2Q0** | Cytoplasmic Membrane | Inner Membrane | Inner Membrane | Yes | No | 9 TMM Helices | 7 TMM Helices |
|  | HP TPASS_0304 | **B2S2Q2** | Unknown | Cytoplasmic | Outer Membrane | No | No | No | No |
|  | HP TPASS_0307 | **B2S2Q5** | Unknown | Cytoplasmic | Inner Membrane | No | No | 1 TMM Helix | 1 TMM Helix |
|  | HP TPASS_0310 | **B2S2Q8** | Unknown | Inner Membrane | Cytoplasmic | No | No | No | No |
|  | HP TPASS_0311 | **B2S2Q9** | Unknown | Extracellular | Cytoplasmic | No | No | No | No |
|  | HP TPASS_0312 | **B2S2R0** | Cytoplasmic Membrane | Inner Membrane | Inner Membrane | No | No | 9 TMM Helices | 7 TMM Helices |
|  | HP TPASS_0314 | **B2S2R2** | Unknown | Extracellular | Cytoplasmic | No | No | No | No |
|  | HP TPASS_0315 | **B2S2R3** | Unknown | Periplasmic | Periplasmic | No | No | No | No |
|  | HP TPASS_0318 | **B2S2R5** | Unknown | Cytoplasmic | Cytoplasmic | No | No | No | No |
|  | HP TPASS_0320 | **B2S2R7** | Unknown | Extracellular | Cytoplasmic | No | Yes | No | No |
|  | HP TPASS_0324 | **B2S2S1** | Unknown | Cytoplasmic | Cytoplasmic | No | No | 2 TMM Helices | 1 TMM Helix |
|  | HP TPASS_0325 | **B2S2S2** | Cytoplasmic Membrane | Cytoplasmic | Outer Membrane | No | No | No | No |
|  | HP TPASS_0332 | **B2S2S9** | Unknown | Extracellular | Cytoplasmic | No | No | No | No |
|  | HP TPASS_0333 | **B2S2T0** | Unknown | Periplasmic | Cytoplasmic | Yes | No | 1 TMM Helix | 1 TMM Helix |
|  | HP TPASS_0334 | **B2S2T1** | Cytoplasmic | Cytoplasmic | Cytoplasmic | No | No | 2 TMM Helices | No |
|  | HP TPASS_0335 | **B2S2T2** | Cytoplasmic Membrane | Cytoplasmic | Cytoplasmic | No | No | 4 TMM Helices | 2 TMM Helices |
|  | HP TPASS_0338 | **B2S2T5** | Cytoplasmic Membrane | Inner Membrane | Inner Membrane | No | No | 3 TMM Helices | 2 TMM Helices |
|  | HP TPASS_0339 | **B2S2T6** | Cytoplasmic | Cytoplasmic | Cytoplasmic | No | No | 3 TMM Helices | No |
|  | HP TPASS_0346 | **B2S2U3** | Unknown | Inner Membrane | Inner Membrane | Yes | No | 3 TMM Helices | 1 TMM Helix |
|  | HP TPASS_0347 | **B2S2U4** | Cytoplasmic Membrane | Inner Membrane | Inner Membrane | No | No | 6 TMM Helices | 4 TMM Helices |
|  | HP TPASS_0348 | **B2S2U5** | Cytoplasmic Membrane | Cytoplasmic | Inner Membrane | No | No | 8 TMM Helices | 5 TMM Helices |
|  | HP TPASS_0352 | **B2S2U9** | Unknown | Extracellular | Cytoplasmic | No | Yes | No | No |
|  | HP TPASS_0355 | **B2S2V2** | Unknown | Extracellular | Extracellular | No | Yes | 1 TMM Helix | No |
|  | HP TPASS_0358 | **B2S2V5** | Cytoplasmic | Cytoplasmic | Cytoplasmic | No | No | 2 TMM Helices | No |
|  | HP TPASS_0359 | **B2S2V6** | Cytoplasmic | Cytoplasmic | Cytoplasmic | No | No | No | No |
|  | HP TPASS_0360 | **B2S2V7** | Unknown | Cytoplasmic | Cytoplasmic | No | Yes | No | No |
|  | HP TPASS_0368 | **B2S2W5** | Unknown | Periplasmic | Cytoplasmic | No | No | 1 TMM Helix | 1 TMM Helix |
|  | HP TPASS_0369 | **B2S2W6** | Unknown | Cytoplasmic | Periplasmic | No | Yes | No | No |
|  | HP TPASS_0370 | **B2S2W7** | Unknown | Cytoplasmic | Cytoplasmic | No | Yes | No | No |
|  | HP TPASS_0371 | **B2S2W8** | Unknown | Cytoplasmic | Cytoplasmic | No | No | No | No |
|  | HP TPASS_0373 | **B2S2X0** | Cytoplasmic | Cytoplasmic | Cytoplasmic | No | No | 1 TMM Helix | No |
|  | HP TPASS_0374 | **B2S2X1** | Cytoplasmic | Cytoplasmic | Cytoplasmic | No | No | No | No |
|  | HP TPASS_0375 | **B2S2X2** | Unknown | Extracellular | Periplasmic | No | No | No | No |
|  | HP TPASS_0376 | **B2S2X3** | Unknown | Periplasmic | Cytoplasmic | No | No | No | No |
|  | HP TPASS_0377 | **B2S2X4** | Unknown | Cytoplasmic | Cytoplasmic | No | No | 1 TMM Helix | 1 TMM Helix |
|  | HP TPASS_0381 | **B2S2X8** | Cytoplasmic Membrane | Inner Membrane | Inner Membrane | No | No | 6 TMM Helices | 7 TMM Helices |
|  | HP TPASS_0382 | **B2S2X9** | Unknown | Extracellular | Cytoplasmic | No | No | No | No |
|  | HP TPASS_0384 | **B2S2Y1** | Cytoplasmic | Cytoplasmic | Cytoplasmic | No | No | No | No |
|  | HP TPASS_0385 | **B2S2Y2** | Unknown | Cytoplasmic | Cytoplasmic | No | No | 1 TMM Helix | 1 TMM Helix |
|  | HP TPASS_0392 | **B2S2Y9** | Unknown | Inner Membrane | Cytoplasmic | No | No | 5 TMM Helices | 2 TMM Helices |
|  | HP TPASS_0404 | **B2S301** | Unknown | Cytoplasmic | Cytoplasmic | No | No | No | No |
|  | HP TPASS_0408 | **B2S305** | Cytoplasmic | Cytoplasmic | Cytoplasmic | No | No | 1 TMM Helix | No |
|  | HP TPASS_0409 | **B2S306** | Unknown | Extracellular | Cytoplasmic | No | No | No | No |
|  | HP TPASS_0412 | **B2S309** | Cytoplasmic | Cytoplasmic | Cytoplasmic | No | No | No | No |
|  | HP TPASS_0415 | **B2S312** | Cytoplasmic | Cytoplasmic | Cytoplasmic | No | No | 2 TMM Helices | No |
|  | HP TPASS_0420 | **B2S317** | Unknown | Cytoplasmic | Cytoplasmic | No | No | No | No |
|  | HP TPASS_0421 | **B2S318** | Outer Membrane | Cytoplasmic | Outer Membrane | Yes | No | No | No |
|  | HP TPASS_0422 | **B2S319** | Unknown | Periplasmic | Outer Membrane | No | No | 1 TMM Helix | No |
|  | HP TPASS_0423 | **B2S320** | Cytoplasmic | Cytoplasmic | Cytoplasmic | No | No | No | No |
|  | HP TPASS_0425 | **B2S322** | Unknown | Cytoplasmic | Cytoplasmic | No | No | No | No |
|  | HP TPASS_0431 | **B2S328** | Unknown | Cytoplasmic | Cytoplasmic | No | No | No | No |
|  | HP TPASS_0432 | **B2S329** | Unknown | Cytoplasmic | Cytoplasmic | No | No | 1 TMM Helix | No |
|  | HP TPASS_0436 | **B2S332** | Unknown | Cytoplasmic | Cytoplasmic | No | No | No | No |
|  | HP TPASS_0437 | **B2S333** | Unknown | Periplasmic | Periplasmic | No | Yes | No | No |
|  | HP TPASS_0438 | **B2S334** | Cytoplasmic | Cytoplasmic | Cytoplasmic | No | No | 1 TMM Helix | No |
|  | HP TPASS_0441 | **B2S337** | Cytoplasmic Membrane | Cytoplasmic | Cytoplasmic | No | No | 2 TMM Helices | No |
|  | HP TPASS_0443 | **B2S339** | Cytoplasmic | Cytoplasmic | Cytoplasmic | No | No | No | No |
|  | HP TPASS_0444 | **B2S340** | Unknown | Extracellular | Periplasmic | No | Yes | 1 TMM Helix | 1 TMM Helix |
|  | HP TPASS_0447 | **B2S343** | Cytoplasmic | Cytoplasmic | Periplasmic | No | No | No | No |
|  | HP TPASS_0449 | **B2S345** | Unknown | Cytoplasmic | Periplasmic | Yes | No | 2 TMM Helices | No |
|  | HP TPASS_0451 | **B2S347** | Unknown | Periplasmic | Periplasmic | Yes | Yes | No | No |
|  | HP TPASS_0453 | **B2S349** | Unknown | Extracellular | Outer Membrane | Yes | No | 3 TMM Helices | 1 TMM Helix |
|  | HP TPASS_0454 | **B2S350** | Cytoplasmic | Cytoplasmic | Cytoplasmic | No | No | No | No |
|  | HP TPASS_0455 | **B2S351** | Unknown | Cytoplasmic | Cytoplasmic | Yes | No | 1 TMM Helix | No |
|  | HP TPASS_0456 | **B2S352** | Unknown | Extracellular | Periplasmic | Yes | Yes | No | No |
|  | HP TPASS_0457 | **B2S353** | Cytoplasmic | Cytoplasmic | Cytoplasmic | No | No | 2 TMM Helices | No |
|  | HP TPASS_0458 | **B2S354** | Cytoplasmic | Cytoplasmic | Cytoplasmic | No | No | No | No |
|  | HP TPASS_0459 | **B2S355** | Cytoplasmic | Cytoplasmic | Cytoplasmic | No | No | No | No |
|  | HP TPASS_0460 | **B2S356** | Unknown | Cytoplasmic | Cytoplasmic | No | No | No | No |
|  | HP TPASS_0461 | **B2S357** | Unknown | Cytoplasmic | Cytoplasmic | No | No | No | No |
|  | HP TPASS_0462 | **B2S358** | Extracellular | Extracellular | Extracellular | No | Yes | 1 TMM Helix | No |
|  | HP TPASS_0463 | **B2S359** | Unknown | Extracellular | Extracellular | No | Yes | No | No |
|  | HP TPASS_0464 | **B2S360** | Cytoplasmic | Cytoplasmic | Cytoplasmic | No | No | 1 TMM Helix | No |
|  | HP TPASS_0465 | **B2S361** | Unknown | Periplasmic | Periplasmic | No | No | 1 TMM Helix | 2 TMM Helices |
|  | HP TPASS_0466 | **B2S362** | Cytoplasmic | Cytoplasmic | Cytoplasmic | No | No | 1 TMM Helix | No |
|  | HP TPASS_0467 | **B2S363** | Unknown | Cytoplasmic | Cytoplasmic | No | No | No | No |
|  | HP TPASS_0468 | **B2S364** | Cytoplasmic | Periplasmic | Cytoplasmic | No | No | 1 TMM Helix | 1 TMM Helix |
|  | HP TPASS_0470 | **B2S365** | Unknown | Cytoplasmic | Cytoplasmic | Yes | No | 1 TMM Helix | No |
|  | HP TPASS_0471 | **B2S366** | Unknown | Cytoplasmic | Cytoplasmic | No | No | 1 TMM Helix | No |
|  | HP TPASS_0473 | **B2S368** | Cytoplasmic Membrane | Inner Membrane | Inner Membrane | No | No | 7 TMM Helices | 7 TMM Helices |
|  | HP TPASS_0474 | **B2S369** | Cytoplasmic | Extracellular | Cytoplasmic | No | No | No | No |
|  | HP TPASS_0479 | **B2S374** | Cytoplasmic Membrane | Cytoplasmic | Inner Membrane | No | No | 5 TMM Helices | 3 TMM Helices |
|  | HP TPASS_0480 | **B2S375** | Cytoplasmic Membrane | Inner Membrane | Inner Membrane | No | No | 3 TMM Helices | 3 TMM Helices |
|  | HP TPASS_0481 | **B2S376** | Cytoplasmic | Cytoplasmic | Cytoplasmic | No | No | 2 TMM Helices | 1 TMM Helix |
|  | HP TPASS_0482 | **B2S377** | Cytoplasmic | Cytoplasmic | Cytoplasmic | No | No | No | No |
|  | HP TPASS_0484 | **B2S379** | Unknown | Cytoplasmic | Cytoplasmic | No | No | 1 TMM Helix | 1 TMM Helix |
|  | HP TPASS_0487 | **B2S382** | Unknown | Extracellular | Cytoplasmic | No | No | 1 TMM Helix | No |
|  | HP TPASS_0489 | **B2S384** | Cytoplasmic | Cytoplasmic | Cytoplasmic | No | No | No | No |
|  | HP TPASS_0490 | **B2S385** | Cytoplasmic | Cytoplasmic | Cytoplasmic | No | No | No | No |
|  | HP TPASS_0491 | **B2S386** | Cytoplasmic Membrane | Cytoplasmic | Inner Membrane | No | No | 1 TMM Helix | 1 TMM Helix |
|  | HP TPASS_0494 | **B2S389** | Cytoplasmic | Cytoplasmic | Cytoplasmic | No | No | 1 TMM Helix | No |
|  | HP TPASS_0496 | **B2S390** | Cytoplasmic | Inner Membrane | Cytoplasmic | No | No | 3 TMM Helices | 1 TMM Helix |
|  | HP TPASS_0502 | **B2S396** | Cytoplasmic | Cytoplasmic | Cytoplasmic | No | No | 1 TMM Helix | No |
|  | HP TPASS_0503 | **B2S397** | Unknown | Inner Membrane | Cytoplasmic | No | No | No | No |
|  | HP TPASS_0504 | **B2S398** | Unknown | Extracellular | Cytoplasmic | No | No | No | No |
|  | HP TPASS_0512 | **B2S3A6** | Cytoplasmic | Cytoplasmic | Cytoplasmic | No | No | No | No |
|  | HP TPASS_0515 | **B2S3A9** | Unknown | Extracellular | Outer Membrane | No | No | 2 TMM Helices | 2 TMM Helices |
|  | HP TPASS_0518 | **B2S3B2** | Unknown | Extracellular | Cytoplasmic | No | No | No | No |
|  | HP TPASS_0522 | **B2S3B5** | Cytoplasmic Membrane | Inner Membrane | Inner Membrane | No | No | 3 TMM Helices | 4 TMM Helices |
|  | HP TPASS_0534 | **B2S3C6** | Unknown | Cytoplasmic | Cytoplasmic | No | No | 1 TMM Helix | No |
|  | HP TPASS_0535 | **B2S3C7** | Unknown | Cytoplasmic | Cytoplasmic | No | No | No | No |
|  | HP TPASS_0539 | **B2S3D1** | Unknown | Extracellular | Cytoplasmic | No | No | No | No |
|  | HP TPASS_0544 | **B2S3D6** | Unknown | Cytoplasmic | Outer Membrane | No | Yes | 1 TMM Helix | No |
|  | HP TPASS_0548 | **B2S3E0** | Unknown | Cytoplasmic | Outer Membrane | No | Yes | 3 TMM Helices | No |
|  | HP TPASS_0552 | **B2S3E4** | Unknown | Inner Membrane | Cytoplasmic | No | Yes | No | No |
|  | HP TPASS_0553 | **B2S3E5** | Cytoplasmic Membrane | Inner Membrane | Inner Membrane | No | No | 9 TMM Helices | 8 TMM Helices |
|  | HP TPASS_0557 | **B2S3E9** | Unknown | Cytoplasmic | Cytoplasmic | No | No | 1 TMM Helix | 1 TMM Helix |
|  | HP TPASS_0558 | **B2S3F0** | Cytoplasmic Membrane | Inner Membrane | Inner Membrane | Yes | No | 8 TMM Helices | 7 TMM Helices |
|  | HP TPASS_0561 | **B2S3F3** | Cytoplasmic Membrane | Cytoplasmic | Cytoplasmic | No | No | 1 TMM Helix | 1 TMM Helix |
|  | HP TPASS_0563 | **B2S3F5** | Unknown | Periplasmic | Periplasmic | No | Yes | No | No |
|  | HP TPASS_0564 | **B2S3F6** | Cytoplasmic Membrane | Inner Membrane | Inner Membrane | Yes | No | 8 TMM Helices | 8 TMM Helices |
|  | HP TPASS_0565 | **B2S3F7** | Unknown | Inner Membrane | Cytoplasmic | No | No | 1 TMM Helix | 1 TMM Helix |
|  | HP TPASS_0567 | **B2S3F9** | Cytoplasmic | Cytoplasmic | Cytoplasmic | No | No | 1 TMM Helix | 1 TMM Helix |
|  | HP TPASS_0570 | **B2S3G2** | Unknown | Inner Membrane | Inner Membrane | No | No | 1 TMM Helix | 2 TMM Helices |
|  | HP TPASS_0572 | **B2S3G4** | Cytoplasmic Membrane | Inner Membrane | Inner Membrane | No | No | 7 TMM Helices | 7 TMM Helices |
|  | HP TPASS_0573 | **B2S3G5** | Unknown | Periplasmic | Cytoplasmic | No | Yes | No | No |
|  | HP TPASS_0577 | **B2S3G8** | Cytoplasmic Membrane | Cytoplasmic | Inner Membrane | No | No | 3 TMM Helices | 2 TMM Helices |
|  | HP TPASS_0579 | **B2S3H0** | Unknown | Cytoplasmic | Cytoplasmic | Yes | No | 1 TMM Helix | No |
|  | HP TPASS_0580 | **B2S3H1** | Cytoplasmic Membrane | Inner Membrane | Inner Membrane | No | No | 6 TMM Helices | 6 TMM Helices |
|  | HP TPASS_0582 | **B2S3H3** | Cytoplasmic Membrane | Inner Membrane | Inner Membrane | Yes | No | 6 TMM Helices | 4 TMM Helices |
|  | HP TPASS_0583 | **B2S3H4** | Unknown | Extracellular | Cytoplasmic | No | Yes | No | No |
|  | HP TPASS_0584 | **B2S3H5** | Cytoplasmic | Cytoplasmic | Cytoplasmic | No | No | No | No |
|  | HP TPASS_0587 | **B2S3H8** | Unknown | Extracellular | Cytoplasmic | No | No | No | No |
|  | HP TPASS_0588 | **B2S3H9** | Cytoplasmic | Cytoplasmic | Cytoplasmic | No | No | No | No |
|  | HP TPASS_0590 | **B2S3I1** | Unknown | Extracellular | Cytoplasmic | No | No | No | No |
|  | HP TPASS_0592 | **B2S3I3** | Cytoplasmic | Cytoplasmic | Cytoplasmic | Yes | No | No | No |
|  | HP TPASS_0593 | **B2S3I4** | Cytoplasmic | Cytoplasmic | Cytoplasmic | No | No | 3 TMM Helices | 3 TMM Helices |
|  | HP TPASS_0594 | **B2S3I5** | Unknown | Periplasmic | Periplasmic | No | Yes | 2 TMM Helices | 2 TMM Helices |
|  | HP TPASS_0598 | **B2S3I8** | Unknown | Cytoplasmic | Outer  Membrane | No | No | 1 TMM Helix | No |
|  | HP TPASS_0599 | **B2S3I9** | Cytoplasmic | Cytoplasmic | Cytoplasmic | No | No | No | No |
|  | HP TPASS_0607 | **B2S3J7** | Unknown | Periplasmic | Cytoplasmic | No | No | No | No |
|  | HP TPASS_0608 | **B2S3J8** | Cytoplasmic | Cytoplasmic | Cytoplasmic | No | No | 1 TMM Helix | 1 TMM Helix |
|  | HP TPASS_0612 | **B2S3K2** | Cytoplasmic | Cytoplasmic | Cytoplasmic | No | No | No | No |
|  | HP TPASS_0613 | **B2S3K3** | Cytoplasmic | Cytoplasmic | Cytoplasmic | No | No | No | No |
|  | HP TPASS_0617 | **B2S3K7** | Unknown | Cytoplasmic | Cytoplasmic | No | No | No | No |
|  | HP TPASS_0618 | **B2S3K8** | Unknown | Cytoplasmic | Cytoplasmic | Yes | No | No | No |
|  | HP TPASS_0619 | **B2S3K9** | Unknown | Cytoplasmic | Periplasmic | No | No | No | No |
|  | HP TPASS_0622 | **B2S3L2** | Unknown | Cytoplasmic | Cytoplasmic | No | No | 1 TMM Helix | No |
|  | HP TPASS_0624 | **B2S3L4** | Outer  Membrane | Cytoplasmic | Cytoplasmic | No | No | No | No |
|  | HP TPASS_0625 | **B2S3L5** | Cytoplasmic | Periplasmic | Periplasmic | No | Yes | No | No |
|  | HP TPASS_0629 | **B2S3L9** | Cytoplasmic | Inner  Membrane | Periplasmic | No | No | No | No |
|  | HP TPASS_0636 | **B2S3M5** | Unknown | Inner  Membrane | Cytoplasmic | No | No | No | No |
|  | HP TPASS_0638 | **B2S3M7** | Cytoplasmic Membrane | Inner  Membrane | Inner  Membrane | No | No | 4 TMM Helices | 4 TMM Helices |
|  | HP TPASS_0645 | **B2S3N4** | Unknown | Inner  Membrane | Cytoplasmic | No | No | No | No |
|  | HP TPASS_0646 | **B2S3N5** | Cytoplasmic Membrane | Inner  Membrane | Inner  Membrane | No | No | 1 TMM Helix | 2 TMM Helices |
|  | HP TPASS_0648 | **B2S3N7** | Cytoplasmic | Cytoplasmic | Cytoplasmic | No | No | 1 TMM Helix | No |
|  | HP TPASS_0651 | **B2S3P0** | Cytoplasmic Membrane | Cytoplasmic | Cytoplasmic | No | No | 7 TMM Helices | 7 TMM Helices |
|  | HP TPASS_0656 | **B2S3P5** | Unknown | Extracellular | Cytoplasmic | No | No | No | No |
|  | HP TPASS_0661 | **B2S3Q0** | Cytoplasmic | Cytoplasmic | Cytoplasmic | No | No | No | No |
|  | HP TPASS_0665 | **B2S3Q4** | Cytoplasmic | Cytoplasmic | Cytoplasmic | No | No | No | No |
|  | HP TPASS_0666 | **B2S3Q5** | Unknown | Periplasmic | Cytoplasmic | No | No | No | No |
|  | HP TPASS_0668 | **B2S3Q7** | Cytoplasmic Membrane | Inner  Membrane | Inner  Membrane | No | No | 5 TMM Helices | 4 TMM Helices |
|  | HP TPASS_0674 | **B2S3R3** | Cytoplasmic | Periplasmic | Cytoplasmic | No | No | No | No |
|  | HP TPASS_0675 | **B2S3R4** | Cytoplasmic | Cytoplasmic | Cytoplasmic | No | No | 2 TMM Helices | 1 TMM Helix |
|  | HP TPASS_0676 | **B2S3R5** | Unknown | Cytoplasmic | Cytoplasmic | No | Yes | No | No |
|  | HP TPASS_0677 | **B2S3R6** | Unknown | Cytoplasmic | Cytoplasmic | No | No | No | No |
|  | HP TPASS_0678 | **B2S3R7** | Unknown | Cytoplasmic | Cytoplasmic | No | No | No | No |
|  | HP TPASS_0679 | **B2S3R8** | Cytoplasmic Membrane | Inner  Membrane | Inner  Membrane | No | No | 3 TMM Helices | 3 TMM Helices |
|  | HP TPASS_0690 | **B2S3S9** | Cytoplasmic | Cytoplasmic | Periplasmic | Yes | No | 1 TMM Helix | No |
|  | HP TPASS_0691 | **B2S3T0** | Cytoplasmic | Cytoplasmic | Cytoplasmic | No | No | No | No |
|  | HP TPASS_0693 | **B2S3T2** | Extracellular | Cytoplasmic | Outer  Membrane | No | No | No | No |
|  | HP TPASS_0697 | **B2S3T6** | Cytoplasmic Membrane | Inner  Membrane | Extracellular | Yes | No | 3 TMM Helices | 2 TMM Helices |
|  | HP TPASS_0698 | **B2S3T7** | Cytoplasmic Membrane | Inner  Membrane | Outer  Membrane | Yes | No | 4 TMM Helices | No |
|  | HP TPASS_0699 | **B2S3T8** | Unknown | Cytoplasmic | Cytoplasmic | No | No | No | No |
|  | HP TPASS_0700 | **B2S3T9** | Unknown | Cytoplasmic | Cytoplasmic | No | No | No | No |
|  | HP TPASS_0702 | **B2S3U1** | Unknown | Extracellular | Periplasmic | No | Yes | No | No |
|  | HP TPASS_0703 | **B2S3U2** | Unknown | Cytoplasmic | Cytoplasmic | No | Yes | 2 TMM Helices | No |
|  | HP TPASS_0706 | **B2S3U5** | Outer  Membrane | Cytoplasmic | Periplasmic | Yes | No | 1 TMM Helix | No |
|  | HP TPASS_0707 | **B2S3U6** | Cytoplasmic Membrane | Inner  Membrane | Inner  Membrane | No | No | 3 TMM Helices | 2 TMM Helices |
|  | HP TPASS_0708 | **B2S3U7** | Unknown | Cytoplasmic | Periplasmic | No | No | 1 TMM Helix | 2 TMM Helices |
|  | HP TPASS_0710 | **B2S3U9** | Cytoplasmic | Cytoplasmic | Cytoplasmic | No | No | 1 TMM Helix | No |
|  | HP TPASS_0711 | **B2S3V0** | Cytoplasmic | Cytoplasmic | Cytoplasmic | No | No | 1 TMM Helix | 1 TMM Helix |
|  | HP TPASS_0719 | **B2S3V8** | Unknown | Cytoplasmic | Cytoplasmic | No | No | 2 TMM Helices | 1 TMM Helix |
|  | HP TPASS_0723 | **B2S3W2** | Unknown | Cytoplasmic | Cytoplasmic | No | Yes | No | No |
|  | HP TPASS_0730 | **B2S3W9** | Cytoplasmic Membrane | Inner  Membrane | Inner  Membrane | No | No | 7 TMM Helices | 6 TMM Helices |
|  | HP TPASS_0731 | **B2S3X0** | Cytoplasmic | Cytoplasmic | Cytoplasmic | No | No | No | No |
|  | HP TPASS_0733 | **B2S3X2** | Unknown | Periplasmic | Extracellular | Yes | Yes | No | No |
|  | HP TPASS_0738 | **B2S3X7** | Cytoplasmic | Cytoplasmic | Cytoplasmic | No | No | No | No |
|  | HP TPASS_0739 | **B2S3X8** | Cytoplasmic | Cytoplasmic | Outer  Membrane | No | No | 1 TMM Helix | 1 TMM Helix |
|  | HP TPASS_0740 | **B2S3X9** | Unknown | Cytoplasmic | Cytoplasmic | No | No | No | No |
|  | HP TPASS_0741 | **B2S3Y0** | Cytoplasmic | Cytoplasmic | Cytoplasmic | No | No | No | No |
|  | HP TPASS_0744 | **B2S3Y3** | Unknown | Cytoplasmic | Cytoplasmic | No | No | No | No |
|  | HP TPASS_0747 | **B2S3Y6** | Unknown | Cytoplasmic | Outer  Membrane | No | Yes | 1 TMM Helix | No |
|  | HP TPASS_0749 | **B2S3Y8** | Unknown | Cytoplasmic | Periplasmic | No | No | No | No |
|  | HP TPASS_0750 | **B2S3Y9** | Cytoplasmic | Cytoplasmic | Cytoplasmic | Yes | No | No | No |
|  | HP TPASS_0752 | **B2S3Z1** | Unknown | Cytoplasmic | Cytoplasmic | No | No | 3 TMM Helices | 1 TMM Helix |
|  | HP TPASS_0753 | **B2S3Z2** | Cytoplasmic Membrane | Inner  Membrane | Inner  Membrane | No | No | 2 TMM Helices | 2 TMM Helices |
|  | HP TPASS_0759 | **B2S3Z8** | Unknown | Inner  Membrane | Cytoplasmic | No | No | No | No |
|  | HP TPASS_0761 | **B2S400** | Cytoplasmic Membrane | Cytoplasmic | Cytoplasmic | No | No | 4 TMM Helices | No |
|  | HP TPASS_0762 | **B2S401** | Unknown | Cytoplasmic | Cytoplasmic | No | No | No | No |
|  | HP TPASS_0763 | **B2S402** | Cytoplasmic Membrane | Inner  Membrane | Inner  Membrane | No | No | 3 TMM Helices | 2 TMM Helices |
|  | HP TPASS_0764 | **B2S403** | Cytoplasmic | Cytoplasmic | Cytoplasmic | No | No | No | No |
|  | HP TPASS_0766 | **B2S405** | Cytoplasmic | Cytoplasmic | Cytoplasmic | No | No | No | No |
|  | HP TPASS_0771 | **B2S410** | Cytoplasmic Membrane | Inner  Membrane | Inner  Membrane | No | No | 9 TMM Helices | 9 TMM Helices |
|  | HP TPASS_0772 | **B2S411** | Unknown | Cytoplasmic | Periplasmic | No | Yes | 1 TMM Helix | No |
|  | HP TPASS_0776 | **B2S415** | Cytoplasmic | Cytoplasmic | Cytoplasmic | No | No | No | No |
|  | HP TPASS_0777 | **B2S416** | Unknown | Cytoplasmic | Cytoplasmic | No | No | No | No |
|  | HP TPASS_0781 | **B2S420** | Unknown | Cytoplasmic | Cytoplasmic | No | No | No | No |
|  | HP TPASS_0782 | **B2S421** | Unknown | Cytoplasmic | Cytoplasmic | No | No | No | No |
|  | HP TPASS_0783 | **B2S422** | Unknown | Cytoplasmic | Cytoplasmic | No | Yes | 1 TMM Helix | 1 TMM Helix |
|  | HP TPASS_0784 | **B2S423** | Unknown | Cytoplasmic | Cytoplasmic | Yes | No | No | No |
|  | HP TPASS_0785 | **B2S424** | Unknown | Cytoplasmic | Cytoplasmic | No | No | 1 TMM Helix | 1 TMM Helix |
|  | HP TPASS_0787 | **B2S426** | Cytoplasmic Membrane | Inner  Membrane | Inner  Membrane | No | No | 3 TMM Helices | 3 TMM Helices |
|  | HP TPASS_0788 | **B2S427** | Cytoplasmic | Cytoplasmic | Cytoplasmic | No | No | 1 TMM Helix | 1 TMM Helix |
|  | HP TPASS_0789 | **B2S428** | Unknown | Cytoplasmic | Periplasmic | Yes | No | 1 TMM Helix | No |
|  | HP TPASS_0791 | **B2S430** | Unknown | Cytoplasmic | Cytoplasmic | No | No | No | 1 TMM Helix |
|  | HP TPASS_0793 | **B2S432** | Cytoplasmic | Cytoplasmic | Cytoplasmic | No | No | 1 TMM Helix | No |
|  | HP TPASS_0795 | **B2S434** | Unknown | Extracellular | Cytoplasmic | No | No | No | No |
|  | HP TPASS_0796 | **B2S435** | Cytoplasmic | Cytoplasmic | Cytoplasmic | No | No | 1 TMM Helix | 1 TMM Helix |
|  | HP TPASS_0799 | **B2S438** | Unknown | Periplasmic | Periplasmic | No | Yes | No | No |
|  | HP TPASS_0802 | **B2S441** | Unknown | Extracellular | Periplasmic | No | Yes | No | No |
|  | HP TPASS_0803 | **B2S442** | Unknown | Cytoplasmic | Inner  Membrane | No | No | No | No |
|  | HP TPASS_0811 | **B2S450** | Unknown | Inner  Membrane | Cytoplasmic | No | No | 1 TMM Helix | No |
|  | HP TPASS_0813 | **B2S451** | Cytoplasmic | Cytoplasmic | Cytoplasmic | No | No | No | No |
|  | HP TPASS_0815 | **B2S453** | Unknown | Cytoplasmic | Cytoplasmic | No | No | No | No |
|  | HP TPASS_0816 | **B2S454** | Unknown | Inner  Membrane | Cytoplasmic | No | No | 2 TMM Helices | No |
|  | HP TPASS_0818 | **B2S456** | Unknown | Cytoplasmic | Cytoplasmic | No | No | No | No |
|  | HP TPASS_0820 | **B2S458** | Unknown | Cytoplasmic | Cytoplasmic | No | No | No | No |
|  | HP TPASS_0822 | **B2S460** | Cytoplasmic Membrane | Inner  Membrane | Inner  Membrane | No | No | 5 TMM Helices | 4 TMM Helices |
|  | HP TPASS_0825 | **B2S463** | Unknown | Periplasmic | Cytoplasmic | No | No | No | No |
|  | HP TPASS_0826 | **B2S464** | Cytoplasmic Membrane | Inner  Membrane | Inner  Membrane | No | No | 3 TMM Helices | 3 TMM Helices |
|  | HP TPASS_0827 | **B2S465** | Cytoplasmic | Cytoplasmic | Cytoplasmic | No | No | 1 TMM Helix | No |
|  | HP TPASS_0829 | **B2S467** | Cytoplasmic | Cytoplasmic | Cytoplasmic | No | No | 3 TMM Helices | No |
|  | HP TPASS_0832 | **B2S470** | Cytoplasmic Membrane | Cytoplasmic | Periplasmic | No | No | 2 TMM Helices | 1 TMM Helix |
|  | HP TPASS_0833 | **B2S471** | Unknown | Cytoplasmic | Cytoplasmic | Yes | No | No | No |
|  | HP TPASS_0836 | **B2S474** | Unknown | Cytoplasmic | Periplasmic | No | No | No | No |
|  | HP TPASS_0839 | **B2S477** | Unknown | Cytoplasmic | Outer  Membrane | No | Yes | 1 TMM Helix | 1 TMM Helix |
|  | HP TPASS_0840 | **B2S478** | Cytoplasmic Membrane | Inner  Membrane | Inner  Membrane | No | No | 13 TMM Helices | 11 TMM Helix |
|  | HP TPASS_0845 | **B2S483** | Cytoplasmic | Cytoplasmic | Cytoplasmic | No | No | No | No |
|  | HP TPASS_0846 | **B2S484** | Cytoplasmic | Inner  Membrane | Cytoplasmic | No | No | No | No |
|  | HP TPASS_0847 | **B2S485** | Cytoplasmic | Cytoplasmic | Cytoplasmic | No | No | No | No |
|  | HP TPASS_0851 | **B2S489** | Cytoplasmic | Cytoplasmic | Cytoplasmic | No | No | No | No |
|  | HP TPASS_0854 | **B2S492** | Cytoplasmic Membrane | Cytoplasmic | Cytoplasmic | Yes | No | 4 TMM Helices | 4 TMM Helices |
|  | HP TPASS_0855 | **B2S493** | Outer  Membrane | Periplasmic | Outer  Membrane | Yes | No | No | No |
|  | HP TPASS_0856 | **B2S494** | Extracellular | Extracellular | Outer  Membrane | Yes | Yes | 2 TMM Helices | 1 TMM Helix |
|  | HP TPASS_0857 | **B2S495** | Unknown | Periplasmic | Cytoplasmic | No | No | No | No |
|  | HP TPASS_0858 | **B2S496** | Unknown | Cytoplasmic | Outer  Membrane | Yes | Yes | No | 1 TMM Helix |
|  | HP TPASS_0859 | **B2S497** | Unknown | Extracellular | Outer  Membrane | No | Yes | No | No |
|  | HP TPASS_0860 | **B2S498** | Unknown | Extracellular | Extracellular | No | Yes | No | No |
|  | HP TPASS_0864 | **B2S4A2** | Outer  Membrane | Outer  Membrane | Outer  Membrane | No | No | 1 TMM Helix | 1 TMM Helix |
|  | HP TPASS_0865 | **B2S4A3** | Outer  Membrane | Cytoplasmic | Outer  Membrane | No | No | 3 TMM Helices | 1 TMM Helix |
|  | HP TPASS_0867 | **B2S4A4** | Unknown | Inner  Membrane | Cytoplasmic | No | No | No | No |
|  | HP TPASS_0869 | **B2S4A6** | Unknown | Inner  Membrane | Cytoplasmic | No | No | 1 TMM Helix | No |
|  | HP TPASS_0871 | **B2S4A8** | Unknown | Periplasmic | Periplasmic | No | Yes | 1 TMM Helix | No |
|  | HP TPASS_0873 | **B2S4B0** | Cytoplasmic | Cytoplasmic | Cytoplasmic | No | No | No | No |
|  | HP TPASS_0874 | **B2S4B1** | Unknown | Cytoplasmic | Cytoplasmic | No | No | No | No |
|  | HP TPASS_0875 | **B2S4B2** | Cytoplasmic | Inner  Membrane | Cytoplasmic | No | No | 1 TMM Helix | No |
|  | HP TPASS_0876 | **B2S4B3** | Unknown | Cytoplasmic | Cytoplasmic | No | No | No | No |
|  | HP TPASS_0877 | **B2S4B4** | Cytoplasmic | Cytoplasmic | Cytoplasmic | No | No | No | No |
|  | HP TPASS_0878 | **B2S4B5** | Cytoplasmic | Cytoplasmic | Cytoplasmic | No | No | 1 TMM Helix | 1 TMM Helix |
|  | HP TPASS_0879 | **B2S4B6** | Cytoplasmic Membrane | Inner  Membrane | Inner  Membrane | No | No | 3 TMM Helices | 1 TMM Helix |
|  | HP TPASS_0882 | **B2S4B9** | Cytoplasmic | Cytoplasmic | Cytoplasmic | No | No | 1 TMM Helix | No |
|  | HP TPASS_0883 | **B2S4C0** | Cytoplasmic Membrane | Inner  Membrane | Inner  Membrane | No | No | 6 TMM Helices | 6 TMM Helices |
|  | HP TPASS_0884 | **B2S4C1** | Cytoplasmic Membrane | Inner  Membrane | Inner  Membrane | No | No | 6 TMM Helices | 6 TMM Helices |
|  | HP TPASS_0893 | **B2S4D0** | Unknown | Cytoplasmic | Cytoplasmic | No | No | No | No |
|  | HP TPASS_0894 | **B2S4D1** | Cytoplasmic | Cytoplasmic | Cytoplasmic | No | No | No | No |
|  | HP TPASS_0895 | **B2S4D2** | Unknown | Inner  Membrane | Cytoplasmic | Yes | No | 1 TMM Helix | 1 TMM Helix |
|  | HP TPASS_0896 | **B2S4D3** | Unknown | Outer  Membrane | Cytoplasmic | No | No | No | No |
|  | HP TPASS_0899 | **B2S4D6** | Unknown | Cytoplasmic | Cytoplasmic | No | No | No | No |
|  | HP TPASS_0900 | **B2S4D7** | Unknown | Cytoplasmic | Inner  Membrane | No | No | No | No |
|  | HP TPASS_0901 | **B2S4D8** | Cytoplasmic Membrane | Inner  Membrane | Inner  Membrane | No | No | 12 TMM Helices | 12 TMM Helices |
|  | HP TPASS_0904 | **B2S4E1** | Unknown | Inner  Membrane | Cytoplasmic | No | No | No | No |
|  | HP TPASS_0906 | **B2S4E3** | Unknown | Cytoplasmic | Cytoplasmic | No | No | No | No |
|  | HP TPASS_0907 | **B2S4E4** | Cytoplasmic | Cytoplasmic | Cytoplasmic | No | No | No | No |
|  | HP TPASS_0910 | **B2S4E7** | Unknown | Cytoplasmic | Cytoplasmic | No | No | No | No |
|  | HP TPASS_0911 | **B2S4E8** | Unknown | Cytoplasmic | Cytoplasmic | No | No | 2 TMM Helices | No |
|  | HP TPASS_0912 | **B2S4E9** | Cytoplasmic | Cytoplasmic | Cytoplasmic | No | No | 2 TMM Helices | No |
|  | HP TPASS_0913 | **B2S4F0** | Unknown | Cytoplasmic | Cytoplasmic | No | No | No | No |
|  | HP TPASS_0914 | **B2S4F1** | Unknown | Cytoplasmic | Cytoplasmic | No | No | No | No |
|  | HP TPASS_0915 | **B2S4F2** | Unknown | Cytoplasmic | Cytoplasmic | No | No | 1 TMM Helix | 1 TMM Helix |
|  | HP TPASS_0916 | **B2S4F3** | Unknown | Cytoplasmic | Cytoplasmic | No | No | No | No |
|  | HP TPASS_0918 | **B2S4F5** | Cytoplasmic Membrane | Cytoplasmic | Inner  Membrane | No | No | 4 TMM Helices | 3 TMM Helices |
|  | HP TPASS_0920 | **B2S4F7** | Unknown | Cytoplasmic | Cytoplasmic | No | No | No | No |
|  | HP TPASS_0922 | **B2S4F9** | Cytoplasmic | Cytoplasmic | Cytoplasmic | No | No | No | No |
|  | HP TPASS_0923 | **B2S4G0** | Extracellular | Cytoplasmic | Outer  Membrane | No | Yes | No | No |
|  | HP TPASS_0927 | **B2S4G4** | Unknown | Cytoplasmic | Periplasmic | No | Yes | No | No |
|  | HP TPASS_0928 | **B2S4G5** | Unknown | Periplasmic | Periplasmic | No | Yes | 1 TMM Helix | 1 TMM Helix |
|  | HP TPASS_0929 | **B2S4G6** | Unknown | Periplasmic | Cytoplasmic | No | Yes | No | No |
|  | HP TPASS_0930 | **B2S4G7** | Cytoplasmic | Inner  Membrane | Cytoplasmic | No | No | No | No |
|  | HP TPASS_0931 | **B2S4G8** | Unknown | Cytoplasmic | Cytoplasmic | No | No | No | No |
|  | HP TPASS_0932 | **B2S4G9** | Unknown | Cytoplasmic | Cytoplasmic | No | No | No | No |
|  | HP TPASS_0937 | **B2S4H4** | Unknown | Cytoplasmic | Cytoplasmic | No | No | No | No |
|  | HP TPASS_0938 | **B2S4H5** | Cytoplasmic | Cytoplasmic | Cytoplasmic | No | No | 1 TMM Helix | No |
|  | HP TPASS_0940 | **B2S4H7** | Unknown | Cytoplasmic | Cytoplasmic | No | Yes | No | No |
|  | HP TPASS_0941 | **B2S4H8** | Cytoplasmic | Cytoplasmic | Cytoplasmic | No | No | No | No |
|  | HP TPASS_0942 | **B2S4H9** | Cytoplasmic | Cytoplasmic | Cytoplasmic | No | No | No | No |
|  | HP TPASS_0944 | **B2S4I1** | Cytoplasmic | Cytoplasmic | Cytoplasmic | No | No | 2 TMM Helices | 2 TMM Helices |
|  | HP TPASS_0950 | **B2S4I7** | Unknown | Inner  Membrane | Periplasmic | No | Yes | 1 TMM Helix | No |
|  | HP TPASS_0954 | **B2S4J1** | Cytoplasmic | Cytoplasmic | Cytoplasmic | Yes | No | 3 TMM Helices | No |
|  | HP TPASS_0955 | **B2S4J2** | Unknown | Inner  Membrane | Cytoplasmic | No | No | 1 TMM Helix | No |
|  | HP TPASS_0956 | **B2S4J3** | Cytoplasmic | Cytoplasmic | Cytoplasmic | Yes | No | No | No |
|  | HP TPASS_0959 | **B2S4J6** | Unknown | Periplasmic | Periplasmic | No | Yes | No | No |
|  | HP TPASS_0962 | **B2S4J9** | Cytoplasmic Membrane | Inner  Membrane | Inner  Membrane | No | No | 4 TMM Helices | 4 TMM Helices |
|  | HP TPASS_0963 | **B2S4K0** | Cytoplasmic Membrane | Inner  Membrane | Inner  Membrane | No | No | 4 TMM Helices | 4 TMM Helices |
|  | HP TPASS_0966 | **B2S4K3** | Unknown | Cytoplasmic | Cytoplasmic | No | No | No | 1 TMM Helix |
|  | HP TPASS_0967 | **B2S4K4** | Cytoplasmic | Outer  Membrane | Cytoplasmic | No | No | No | No |
|  | HP TPASS_0968 | **B2S4K5** | Cytoplasmic | Cytoplasmic | Cytoplasmic | No | No | No | No |
|  | HP TPASS_0969 | **B2S4K6** | Outer  Membrane | Outer  Membrane | Outer  Membrane | No | No | 2 TMM Helices | No |
|  | HP TPASS_0970 | **B2S4K7** | Unknown | Cytoplasmic | Cytoplasmic | No | No | No | No |
|  | HP TPASS_0972 | **B2S4K9** | Cytoplasmic Membrane | Inner  Membrane | Inner  Membrane | No | No | 9 TMM Helices | 6 TMM Helices |
|  | HP TPASS_0974 | **B2S4L1** | Unknown | Periplasmic | Cytoplasmic | No | No | No | No |
|  | HP TPASS_0975 | **B2S4L2** | Cytoplasmic | Cytoplasmic | Cytoplasmic | No | No | No | No |
|  | HP TPASS_0976 | **B2S4L3** | Cytoplasmic Membrane | Cytoplasmic | Inner  Membrane | No | Yes | 2 TMM Helices | 2 TMM Helices |
|  | HP TPASS_0977 | **B2S4L4** | Cytoplasmic | Cytoplasmic | Cytoplasmic | No | No | No | No |
|  | HP TPASS_0979 | **B2S4L6** | Cytoplasmic | Cytoplasmic | Cytoplasmic | No | No | No | No |
|  | HP TPASS_0983 | **B2S4M0** | Unknown | Inner  Membrane | Cytoplasmic | No | Yes | No | No |
|  | HP TPASS_0986 | **B2S4M3** | Cytoplasmic Membrane | Inner  Membrane | Inner  Membrane | Yes | No | 9 TMM Helices | 6 TMM Helices |
|  | HP TPASS_0987 | **B2S4M4** | Unknown | Inner  Membrane | Cytoplasmic | No | No | No | No |
|  | HP TPASS_0988 | **B2S4M5** | Cytoplasmic Membrane | Inner  Membrane | Inner  Membrane | No | No | 6 TMM Helices | 5 TMM Helices |
|  | HP TPASS_0990 | **B2S4M7** | Outer  Membrane | Periplasmic | Cytoplasmic | No | Yes | 1 TMM Helix | No |
|  | HP TPASS_0992 | **B2S4M9** | Unknown | Outer  Membrane | Cytoplasmic | No | No | No | No |
|  | HP TPASS_0994 | **B2S4N1** | Cytoplasmic | Cytoplasmic | Cytoplasmic | No | No | No | No |
|  | HP TPASS_0996 | **B2S4N3** | Cytoplasmic | Cytoplasmic | Outer  Membrane | No | No | 1 TMM Helix | No |
|  | HP TPASS_1000 | **B2S4N7** | Cytoplasmic | Inner  Membrane | Cytoplasmic | No | No | No | No |
|  | HP TPASS_1001 | **B2S4N8** | Unknown | Cytoplasmic | Cytoplasmic | No | No | No | No |
|  | HP TPASS_1002 | **B2S4N9** | Unknown | Cytoplasmic | Cytoplasmic | Yes | No | 1 TMM Helix | No |
|  | HP TPASS_1003 | **B2S4P0** | Cytoplasmic Membrane | Inner  Membrane | Inner  Membrane | No | No | 8 TMM Helices | 8 TMM Helices |
|  | HP TPASS_1014 | **B2S4Q1** | Cytoplasmic | Cytoplasmic | Cytoplasmic | No | No | 1 TMM Helix | 1 TMM Helix |
|  | HP TPASS_1018 | **B2S4Q5** | Cytoplasmic | Cytoplasmic | Cytoplasmic | No | No | 1 TMM Helix | 1 TMM Helix |
|  | HP TPASS_1029 | **B2S4R6** | Cytoplasmic | Cytoplasmic | Cytoplasmic | No | No | No | No |
|  | HP TPASS_1030 | **B2S4R7** | Unknown | Cytoplasmic | Periplasmic | No | Yes | No | No |
|  | HP TPASS_1032 | **B2S4R9** | Unknown | Cytoplasmic | Cytoplasmic | No | No | 1 TMM Helix | 1 TMM Helix |
|  | HP TPASS_1033 | **B2S4S0** | Unknown | Cytoplasmic | Cytoplasmic | No | No | 1 TMM Helix | No |
|  | HP TPASS_1034 | **B2S4S1** | Cytoplasmic Membrane | Inner  Membrane | Inner  Membrane | No | No | 10 TMM Helices | 9 TMM Helices |
