# Supplementary material for: Identification of Functional Candidates amongst Hypothetical Proteins of Treponema pallidum ssp. pallidum
Source: PLoS One. 2015 Apr 20;10(4):e0124177. doi: 10.1371/journal.pone.0124177 (PMC4403809; doi:10.1371/journal.pone.0124177)
Supplement: S3 Table — (DOC) [file pone.0124177.s003.doc]

| **Table S3: List of predicted results of Blast, STRING, HMMER, SMART and INTERPROSCAN for 444 HPs from *T. pallidum ssp. pallidum*** | | | | | | | | |
| --- | --- | --- | --- | --- | --- | --- | --- | --- |
| **S. No** | **Protein name** | **Uniprot ID** | **Major BLAST hit** | | **Predicted functional partner**  **(STRING)** | **HMMER** | **SMART** | **INTERPROSCAN** |
|  | HP TPASS_0004 | **B2S1V4** | RNA binding protein | | recombination protein RecF | Protein of unknown function (DUF721) | PH domain-like | Protein of unknown function (DUF721) |
|  | HP TPASS_0008 | **B2S1V7** | Lipoprotein | | DNA topoisomerase (ATP-hydrolyzing) subunit A | No match found | L-aspartase | No match found |
|  | HP TPASS_0010 | **B2S1V8** | No match found | | No match found | No match found | beta and beta-prime subunits of DNA dependent RNA-polymerase | No match found |
|  | HP TPASS_0012 | **B2S1W0** | TonB dependent receptor | | ATP-dependent protease LA | No match found | Nucleotidylyl transferase | No match found |
|  | HP TPASS_0013 | **B2S1W1** | Lipoprotein | | No match found | Protein of unknown function DUF3798 | UDP-Glycosyltransferase/glycogen phosphorylase | Protein of unknown function DUF3798 |
|  | HP TPASS_0014 | **B2S1W2** | Transcriptional regulator | | No match found | Protein of unknown function DUF3798 | TRAF domain-like | Protein of unknown function DUF3798 |
|  | HP TPASS_0017 | **B2S1W5** | Tetratricopeptide repeat (TPR) containing protein | | transcription elongation factor | Tetratricopeptide repeat (TPR) containing protein | Tetratricopeptide repeat (TPR) containing protein | Tetratricopeptide repeat (TPR) containing protein |
|  | HP TPASS_0021 | **B2S1W9** | Helicase C-terminal | | sodium- and chloride- dependent transporter | No match found | Phase 1 flagellin | No match found |
|  | HP TPASS_0022 | **B2S1X0** | Helicase C-terminal | | sodium- and chloride- dependent transporter | Helicase conserved C-terminal domain | Nitrile hydratase alpha chain | Helicase conserved C-terminal domain |
|  | HP TPASS_0024 | **B2S1X2** | Trk family potassium (K+) transporter, NAD+ binding protein | | K+ transport protein | potassium (K+) transporter, NAD+ binding protein | TrkA_N, TrkA_C domain | Regulator of K+ conductance, C-terminal |
|  | HP TPASS_0025 | **B2S1X3** | Peptidase_M16C | | aminopeptidase P | Peptidase M16C associated | Peptidase M16C associated | Metalloenzyme, LuxS/M16 peptidase-like |
|  | HP TPASS_0031 | **B2S1X9** | No significant match found | | chaperonin GroEL | No match found | Thiolase-like | Transmembrane region |
|  | HP TPASS_0033 | **B2S1Y1** | Membrane protein | | 16S ribosomal RNA methyltransferase RsmE | Protein of unknown function (DUF1361) | Cysteine proteinases | Protein of unknown function DUF1361 |
|  | HP TPASS_0039 | **B2S1Y7** | No significant match found | | regulatory protein | No match found | ATP synthase (F1-ATPase), gamma subunit | No match found |
|  | HP TPASS_0041 | **B2S1Y9** | No significant match found | | No match found | No match found | Thymidylate synthase-complementing protein Thy1 | No match found |
|  | HP TPASS_0042 | **B2S1Z0** | peptidoglycan-binding lysin domain-containing protein | | lytic transglycosylase | LysM domain | LysM domain | LysM domain containing protein |
|  | HP TPASS_0046 | **B2S1Z4** | PSP1 domain-containing protein | | ribulose-phosphate 3-epimerase | PSP1 C-terminal conserved region | PSP1 domain | PSP1 C-terminal domain |
|  | HP TPASS_0047 | **B2S1Z5** | Protein of unknown function (DUF327) | | signal peptidase II-like activity | Protein of unknown function (DUF327) | Nicotinate mononucleotide:5,6-dimethylbenzimidazole phosphoribosyltransferase (CobT) | Protein of unknown function (DUF327) |
|  | HP TPASS_0048 | **B2S1Z6** | cell shape determination protein CcmA | | signal peptidase II-like activity | Polymer-forming cytoskeletal | DUF583 domain | Polymer-forming cytoskeletal |
|  | HP TPASS_0049 | **B2S1Z7** | M23/M37 peptidase domain-containing protein | | signal peptidase II-like activity | Peptidoglycan hydrolase LytM | Peptidase_M23 | Peptidase M23 |
|  | HP TPASS_0050 | **B2S1Z8** | phosphoribosyl transferase | | protoporphyrinogen oxidase | Phosphoribosyl transferase domain | Pribosyltran | Phosphoribosyl transferase |
|  | HP TPASS_0054 | **B2S202** | rRNA (adenosine-2'-O-)-methyltransferase | | methylates position 2 of adenine 2503 in 23S rRNA | RNA 2'-O ribose methyltransferase substrate binding | SpoU_methylase | RNA 2'-O ribose methyltransferase substrate binding |
|  | HP TPASS_0055 | **B2S203** | oxaloacetate decarboxylase subunit gamma | | pyruvate carboxylase subunit B | Oxaloacetate decarboxylase, gamma chain | OAD_gamma | Sodium ion-translocating decarboxylase |
|  | HP TPASS_0059 | **B2S207** | oxidation resistance protein | | replicative DNA helicase DnaB | No match found | Concanavalin A-like lectins/glucanases | No match found |
|  | HP TPASS_0064 | **B2S212** | starch synthase | | 30S ribosomal protein S6 | Mitochondrial branched-chain alpha-ketoacid dehydrogenase kinase | alpha-ketoacid dehydrogenase kinase, N-terminal domain | No match found |
|  | HP TPASS_0065 | **B2S213** | SAM dependent methyltransferase | | Specifically methylates position 2 of adenine 2503 in 23S rRNA | Methyltransferase | S-adenosyl-L-methionine-dependent methyltransferases | RNA methyltransferase |
|  | HP TPASS_0066 | **B2S214** | No significant match found | | flagellar motor switch protein FliM | Tetratricopeptide repeat (TPR) containing protein | Tetratricopeptide repeat (TPR) | No match found |
|  | HP TPASS_0067 | **B2S215** | TPR domain-containing protein | | Specifically methylates position 2 of adenine 2503 in 23S rRNA | Tetratricopeptide repeat  containing protein | Tetratricopeptide repeat  containing protein | Tetratricopeptide repeat  containing protein |
|  | HP TPASS_0068 | **B2S216** | Fe-S-cluster redox enzyme | | DNA-directed RNA polymerase subunit beta | 4Fe-4S single cluster domain | Ribosomal RNA large subunit methyltransferase N | Ribosomal RNA large subunit methyltransferase |
|  | HP TPASS_0069 | **B2S217** | N-acetylmuramoyl-L-alanine amidase | | ATP-dependent Clp protease | SH3 domain of SH3b2 type | Terminal deoxynucleotidyl transferase | No match found |
|  | HP TPASS_0070 | **B2S218** | phosphoenolpyruvate-protein phosphotransferase | | ATP-dependent Clp protease | No match found | Aldolase | Transmembrane domain |
|  | HP TPASS_0072 | **B2S220** | glutaredoxin | | ATP-dependent Clp protease, subunit B | Glutaredoxin | Glutaredoxin | glutaredoxin-related |
|  | HP TPASS_0073 | **B2S221** | HDOD domain protein | | No significant match found | Histidine kinase-, DNA gyrase B-, and HSP90-like ATPase | HATPase_c | Histidine kinase-like ATPase, ATP-binding domain |
|  | HP TPASS_0079 | **B2S227** | bifunctional aldehyde oxidase/xanthine dehydrogenase | | quinoline 2-oxidoreductase | Molybdopterin-binding domain of aldehyde dehydrogenase | Aldehyde oxidase and xanthine dehydrogenase, a/b hammerhead domain | Aldehyde oxidase/xanthine dehydrogenase, molybdopterin binding |
|  | HP TPASS_0081 | **B2S229** | FAD binding domain in molybdopterin dehydrogenase | | quinoline 2-oxidoreductase | FAD binding domain in molybdopterin dehydrogenase | FAD binding protein | Molybdopterin dehydrogenase, FAD-binding |
|  | HP TPASS_0083 | **B2S231** | glycoside hydrolase domain protein | | UDP-N-acetylmuramoylalanine--D-glutamate ligase | glycosyl hydrolase | (Trans)glycosidases | glycosyl hydrolase |
|  | HP TPASS_0084 | **B2S232** | thioredoxin family protein | | PTS system, nitrogen regulatory IIA component | No match found | Thioredoxin-like | No match found |
|  | HP TPASS_0086 | **B2S234** | PilZ domain protein | | PTS system, nitrogen regulatory IIA component | PilZ domain | PilZ domain | PilZ domain |
|  | HP TPASS_0087 | **B2S235** | No significant match found | | rRNA methylase | Protein of unknown function DUF192 | Tryptophan synthase beta subunit-like PLP-dependent enzymes | Protein of unknown function DUF192 |
|  | HP TPASS_0088 | **B2S236** | Histidine kinase | | cyclic nucleotide binding protein | No match found | Heme-dependent peroxidase | No match found |
|  | HP TPASS_0093 | **B2S241** | Transcriptional regulator | | RNA polymerase sigma-24 factor | zinc-finger domain containing | No match found | No match found |
|  | HP TPASS_0095 | **B2S243** | Tetratricopeptide repeat (TPR) containing protein | | phosphate acetyltransferase | Tetratricopeptide repeat (TPR) containing protein | Tetratricopeptide repeat (TPR) containing protein | Tetratricopeptide repeat (TPR) containing protein |
|  | HP TPASS_0110 | **B2S258** | excinuclease ABC, A subunit-related protein | | RNA polymerase sigma-54 factor | No match found | alpha/beta-Hydrolases | No match found |
|  | HP TPASS_0118 | **B2S266** | No significant match found | | amino acid ABC transporter, ATP-binding protein | Type IV pili methyl-accepting chemotaxis transducer N-term | PLC-like phosphodiesterases | No match found |
|  | HP TPASS_0121 | **B2S269** | lysine 2,3-aminomutase | | lysyl-tRNA synthetase | Lysine-2,3-aminomutase | Elp_3 domain | Lysine-2,3-aminomutase |
|  | HP TPASS_0123 | **B2S271** | tetratricopeptide repeat protein | | GTP-dependent nucleic acid-binding protein EngD | Tetratricopeptide repeat (TPR) containing protein | TPR-like | Tetratricopeptide repeat (TPR) containing protein |
|  | HP TPASS_0126 | **B2S274** | imidazole glycerol phosphate synthase subunit | | No match found | Outer membrane protein beta-barrel domain | Outer membrane protein | Outer membrane protein/outer membrane enzyme PagP , beta-barre |
|  | HP TPASS_0127 | **B2S275** | No significant match found | | No match found | Protein of unknown function (DUF2715) | Hepatitis B viral capsid | Protein of unknown function (DUF2715) |
|  | HP TPASS_0128 | **B2S276** | No significant match found | | No match found | No match found | Nucleoplasmin-like/VP (viral coat and capsid proteins) | No match found |
|  | HP TPASS_0129 | **B2S277** | glutamate 5-kinase | | No match found | No match found | Pyruvate kinase C-terminal domain-like | No match found |
|  | HP TPASS_0130 | **B2S278** | Repeat protein K | | protein TprD | No match found | Nitrous oxide reductase, N-terminal domain | No match found |
|  | HP TPASS_0132 | **B2S280** | lantibiotic dehydratase domain-containing protein | | No match found | No match found | No match found | No match found |
|  | HP TPASS_0133 | **B2S281** | Outer membrane protein | | No match found | No match found | DNA/RNA polymerases | Outer membrane protein |
|  | HP TPASS_0134 | **B2S282** | Outer membrane protein | | No match found | No match found | WD40 repeat-like | Outer membrane protein |
|  | HP TPASS_0135 | **B2S283** | No significant match found | | No match found | No match found | Sec7 domain | No match found |
|  | HP TPASS_0136 | **B2S284** | Outer membrane protein | | No match found | No match found | alpha/beta-Hydrolases | Outer membrane protein |
|  | HP TPASS_0137 | **B2S285** | No significant match found | | No match found | No match found | No match found | No match found |
|  | HP TPASS_0138 | **B2S286** | PAS/PAC sensor protein | | K+ transport protein | 7tm Chemosensory receptor | PYP-like sensor domain | Transmembrane domain |
|  | HP TPASS_0139 | **B2S287** | Trk family potassium (K+) transporter, NAD+ binding protein | | K+ transport protein | TrkA-N domain (K+ transport protein) | TrkA_N domain  (K+ transport protein) | Regulator of K+ conductance, N-terminal |
|  | HP TPASS_0148 | **B2S295** | Membrane protein | | possible alpha-amylase 1 | No match found | Trypsin-like serine proteases | Membrane protein |
|  | HP TPASS_0149 | **B2S296** | Membrane protein | | possible alpha-amylase 1 | Regulatory protein YrvL | ITPase-like | Membrane protein |
|  | HP TPASS_0150 | **B2S297** | PTS fructose transporter subunit IIA | | nitrogen fixation protein | No match found | AhpD-like | No match found |
|  | HP TPASS_0151 | **B2S298** | NADH dehydrogenase (ubiquinone), subunit RnfD | | nitrogen fixation protein | NQR2, RnfD, RnfE family | NQR2, RnfD, RnfE domain | NADH-quinone reductase NQR2/RnfD |
|  | HP TPASS_0153 | **B2S2A0** | acid phosphatase/vanadium-dependenthaloperoxidase related protein | nitrogen fixation protein | | PAP2 family | Acid phosphatase/Vanadium-dependent haloperoxidase | Acid phosphatase/vanadium-dependent haloperoxidase |
|  | HP TPASS_0154 | **B2S2A1** | RNA pseudouridylate synthase | | fibronectin binding protein | RNA pseudouridylate synthase | PseudoU_synth_2 domain | Pseudouridine synthase |
|  | HP TPASS_0156 | **B2S2A3** | 4-hydroxybenzoyl-CoA thioesterase | | prolyl-tRNA synthetase | Thioesterase | 4HBT domain | Acyl-CoA thioester hydrolase YbgC/YbaW family |
|  | HP TPASS_0157 | **B2S2A4** | glycerol-3-phosphate O-acyltransferase | | possible lysophosphatidic acid acyltransferase | Glycerol-3-phosphate (1)-acyltransferase | acyltransferase | Phospholipid/glycerol acyltransferase |
|  | HP TPASS_0158 | **B2S2A5** | HAD-superfamily hydrolase | | prolyl-tRNA synthetase | Haloacid dehalogenase-like hydrolase | Hydrolase | Haloacid dehalogenase-like hydrolase |
|  | HP TPASS_0159 | **B2S2A6** | glutamate--cysteine ligase | | No match found | No match found | PLP-dependent transferases | No match found |
|  | HP TPASS_0161 | **B2S2A8** | peptidoglycan glycosyltransferase | | No match found | No match found | No match found | No match found |
|  | HP TPASS_0169 | **B2S2B6** | No significant match found | | protein Pfs  (Responsible for cleavage of the glycosidic bond in both 5'-methylthioadenosine) | No match found | No match found | No match found |
|  | HP TPASS_0172 | **B2S2B9** | diguanylate cyclase | | No match found | GGDEF domain | Photosynthetic reaction centre, H-chain, cytoplasmic domain | No match found |
|  | HP TPASS_0173 | **B2S2C0** | diguanylate cyclase | | No match found | Uncharacterized protein conserved in bacteria (DUF2140) | Actin-like ATPase domain | No match found |
|  | HP TPASS_0174 | **B2S2C1** | protein of unknown function DUF115 | | No match found | protein of unknown function DUF115 | (Trans)glycosidases | No match found |
|  | HP TPASS_0175 | **B2S2C2** | protein of unknown function DUF115 | | No match found | protein of unknown function DUF115 | Bcr-Abl oncoprotein oligomerization domain | Protein of unknown function DUF115 |
|  | HP TPASS_0176 | **B2S2C3** | ABC transporter ATP-binding protein | | No match found | No match found | SMAD/FHA domain | No match found |
|  | HP TPASS_0177 | **B2S2C4** | protein of unknown function DUF115 | | No match found | protein of unknown function DUF115 | Clavaminate synthase-like | protein of unknown function DUF115 |
|  | HP TPASS_0178 | **B2S2C5** | Aconitate hydratase | | No match found | No match found | P-loop containing nucleoside triphosphate hydrolases | No match found |
|  | HP TPASS_0179 | **B2S2C6** | No significant match found | | No match found | No match found | Fe-only hydrogenase | No match found |
|  | HP TPASS_0180 | **B2S2C7** | dioxygenases related to 2-nitropropane dioxygenase | | No match found | No match found | Aspartate/ornithine carbamoyltransferase | No match found |
|  | HP TPASS_0181 | **B2S2C8** | septum formation initiator | | Bifunctional enzyme that catalyzes the formation of 4- diphosphocytidyl-2 | Septum formation initiator | Divlc domain | Septum formation initiator |
|  | HP TPASS_0182 | **B2S2C9** | Sua5/YciO/YrdC/YwlC family protein  (Translation factor) | | 30S ribosomal protein S2 | Telomere recombination | Sua5_yciO_yrdC domain | DHBP synthase RibB-like alpha/beta domain |
|  | HP TPASS_0183 | **B2S2D0** | testican-3 isoform 4 | | SsrA-binding protein | Domain of unknown function (DUF3876) | Thioesterase/thiol ester dehydrase-isomerase | No match found |
|  | HP TPASS_0214 | **B2S2G2** | No significant match found | | 50S ribosomal protein L17 | Desulfoferrodoxin, N-terminal domain | Tryptophan synthase beta subunit-like PLP-dependent enzymes | No match found |
|  | HP TPASS_0222 | **B2S2G9** | methyltransferase domain protein | | D,D-carboxypeptidase | Domain of unknown function (DUF4200) | Phospholipase D/nuclease | No match found |
|  | HP TPASS_0223 | **B2S2H0** | pyridoxal phosphate-dependent aspartate aminotransferase | | N-acetylphosphinothricin-tripetide-deacetylase | Aminotransferase class I and II | Aminotransferase | Pyridoxal phosphate-dependent transferase |
|  | HP TPASS_0224 | **B2S2H1** | No significant match found | | leucine-rich repeat protein TpLRR | Copper-binding of amyloid precursor, CuBD | No match found | No match found |
|  | HP TPASS_0226 | **B2S2H3** | cobalt transport protein | | cobalt ABC transporter | Cobalt transport protein | No match found | No match found |
|  | HP TPASS_0231 | **B2S2H8** | pseudouridine synthase, RluA family | | tRNA pseudouridine 55 synthase | RNA pseudouridylate synthase | Pseudouridine synthase | Pseudouridine synthase |
|  | HP TPASS_0232 | **B2S2H9** | No significant match found | | possible anti-sigma F factor antagonist | No match found | No match found | No match found |
|  | HP TPASS_0245 | **B2S2J2** | ATPase | | 30S ribosomal protein S7 | P-loop containing nucleoside triphosphate hydrolase | P-loop containing nucleoside triphosphate hydrolases | P-loop containing nucleoside triphosphate hydrolase |
|  | HP TPASS_0246 | **B2S2J3** | von Willebrand factor type A domain-containing protein | | N-acetylmuramoyl-L-alanine amidase | von Willebrand factor type A domain | VWA domain | von Willebrand factor, type A |
|  | HP TPASS_0248 | **B2S2J5** | sporulation and spore germination | | N-acetylmuramoyl-L-alanine amidase | Sporulation and spore germination | ALDH-like | No match found |
|  | HP TPASS_0250a | **B2S2J7** | No significant match found | | 30S ribosomal protein S20 | No match found | Multiheme cytochrome | No match found |
|  | HP TPASS_0253 | **B2S2K1** | polymer-forming cytoskeletal family protein | | apolipoprotein N-acyltransferase | Polymer-forming cytoskeletal | Head and neck region of the ectodomain of NDV fusion glycoprotein | Polymer-forming cytoskeletal |
|  | HP TPASS_0258 | **B2S2K6** | signal peptide protein | | thymidylate kinase | No match found | Snake toxin-like | No match found |
|  | HP TPASS_0259 | **B2S2K7** | LysM domain-containing protein | | protein Smf | LysM domain | LysM domain | LysM domain |
|  | HP TPASS_0260 | **B2S2K8** | SH3 type 3 domain-containing protein | | catabolite gene activator | Bacterial SH3 domain | SH3 type 3 domain | No match found |
|  | HP TPASS_0263 | **B2S2L1** | fibronectin type III domain-containing protein | | deoxyribose-phosphate aldolase | Fibronectin type III | FN3 domain | Fibronectin type 3 domain |
|  | HP TPASS_0266 | **B2S2L4** | No significant match found | | amino acid ABC transporter | No match found | Thiamin pyrophosphokinase, catalytic domain | No match found |
|  | HP TPASS_0267 | **B2S2L5** | Polymer-forming cytoskeletal | | polynucleotide adenylyltransferase | Polymer-forming cytoskeletal | RNA polymerase subunit RPB10 | Polymer-forming cytoskeletal |
|  | HP TPASS_0268 | **B2S2L6** | TPR domain-containing protein | | polynucleotide adenylyltransferase | Tetratricopeptide repeat | Tetratricopeptide repeat | Tetratricopeptide repeat |
|  | HP TPASS_0269 | **B2S2L7** | MiaB-like tRNA modifying enzyme | | tRNA delta(2)-isopentenylpyrophosphate transferase | MiaB-like tRNA modifying enzyme | Elp3 domain | Methylthiotransferase, N-terminal |
|  | HP TPASS_0273 | **B2S2M1** | membrane protein | | chromosome partitioning protein | No match found | No match found | membrane-bound protein |
|  | HP TPASS_0278 | **B2S2M6** | No significant match found | | bifunctional cytidylate kinase | No match found | Protein kinase-like | No match found |
|  | HP TPASS_0280 | **B2S2M8** | amidohydrolase | | bifunctional cytidylate kinase | No match found | Pectin lyase-like | No match found |
|  | HP TPASS_0281 | **B2S2M9** | No significant match found | | bifunctional cytidylate kinase | No match found | Class II aaRS and biotin synthetases | No match found |
|  | HP TPASS_0282 | **B2S2N0** | TPR domain-containing protein | | ribosomal protein S1 | Tetratricopeptide repeat | No match found | Tetratricopeptide repeat |
|  | HP TPASS_0284 | **B2S2N2** | radical SAM additional 4Fe4S-binding SPASM domain-containing protein | | spore coat polysaccharide biosynthesis protein F | Spore Coat Polysaccharide Biosynthesis Protein SpsA; Chain A | Lysozyme-like | No match found |
|  | HP TPASS_0285 | **B2S2N3** | spiro-SPASM protein | | spore coat polysaccharide biosynthesis protein F | spiro-SPASM protein | Elp3 domain | 4Fe4S-binding SPASM domain |
|  | HP TPASS_0286 | **B2S2N4** | glycosyl transferase family | | spore coat polysaccharide biosynthesis protein F | No match found | Nucleotide-diphospho-sugar transferases | No match found |
|  | HP TPASS_0287 | **B2S2N5** | SAC3/GANP family protein | | spore coat polysaccharide biosynthesis protein F | No match found | Glyceraldehyde-3-phosphate dehydrogenase-like, C-terminal domain | No match found |
|  | HP TPASS_0289 | **B2S2N7** | cytidylyltransferase domain-containing protein | | spore coat polysaccharide biosynthesis protein F | S-adenosyl-L-methionine-dependent methyltransferases | Methyltransferase | Methyltransferase |
|  | HP TPASS_0290 | **B2S2N8** | Cof-like hydrolase | | phosphoribosylpyrophosphate synthetase | haloacid dehalogenase-like hydrolase | Hydorlase | haloacid dehalogenase-like hydrolase |
|  | HP TPASS_0291 | **B2S2N9** | FMN-dependent family dehydrogenase | | outer membrane protein TpN50 | FMN-dependent dehydrogenase | FMN-dependent dehydrogenase | FMN-dependent dehydrogenase |
|  | HP TPASS_0293 | **B2S2P1** | coat F domain-containing protein | | phosphoribosylpyrophosphate synthetase | No match found | UBC-like | No match found |
|  | HP TPASS_0296 | **B2S2P4** | dephospho-CoA kinase | | 4'-phosphopantetheinyl transferase | Dephospho-CoA kinase | Dephospho-CoA kinase | Dephospho-CoA kinase |
|  | HP TPASS_0297 | **B2S2P5** | sporulation and cell division repeat protein | | Catalyzes the phosphorylation of the 3'-hydroxyl group of dephosphocoenzymA | Sporulation related domain | Sporulation related domain | Sporulation-related domain |
|  | HP TPASS_0299 | **B2S2P7** | sugar ABC superfamily ATP binding cassette transporter | | ribose/galactose ABC transporter, ATP-binding protein | No match found | GHMP Kinase, C-terminal domain | No match found |
|  | HP TPASS_0301 | **B2S2P9** | branched-chain amino acid ABC transporter permease | | ribose/galactose ABC transporter | Branched-chain amino acid transport system / permease component | BPD_transp_2 domain | ABC transporter, permease |
|  | HP TPASS_0302 | **B2S2Q0** | branched-chain amino acid ABC transporter permease | | ribose/galactose ABC transporter | Branched-chain amino acid transport system / permease component | BPD_transp_2 domain | ABC transporter, permease |
|  | HP TPASS_0304 | **B2S2Q2** | DNA mismatch repair protein MutL | | DNA mismatch repair protein MutL | Peptidase MA superfamily | Quinoprotein alcohol dehydrogenase-like | Peptidase MA superfamily |
|  | HP TPASS_0307 | **B2S2Q5** | PASTA domain containing protein | | amino acid ABC transporter | PASTA domain | PASTA domain | PASTA domain |
|  | HP TPASS_0310 | **B2S2Q8** | single-stranded DNA-binding protein | | amino acid ABC transporter | single-stranded DNA-binding protein | single-stranded DNA-binding protein | single-stranded DNA-binding protein |
|  | HP TPASS_0311 | **B2S2Q9** | No significant match found | | No match found | No match found | Fe-only hydrogenase | No match found |
|  | HP TPASS_0312 | **B2S2R0** | Membrane protein | | No match found | Protein of unknown function DUF368 | Protein of unknown function DUF368 | Protein of unknown function DUF368 |
|  | HP TPASS_0314 | **B2S2R2** | No significant match found | | No match found | Protein of unknown function DUF2715 | Subtilisin-like | Protein of unknown function DUF2715 |
|  | HP TPASS_0315 | **B2S2R3** | type I L-asparaginase | | No match found | Protein of unknown function DUF2715 | Fe,Mn superoxide dismutase (SOD), C-terminal domain | Protein of unknown function DUF2715 |
|  | HP TPASS_0318 | **B2S2R5** | No significant match found | | membrane lipoprotein | No match found | alpha/beta-Hydrolases | No match found |
|  | HP TPASS_0320 | **B2S2R7** | No significant match found | | membrane lipoprotein | No match found | No match found | No match found |
|  | HP TPASS_0324 | **B2S2S1** | outer membrane protein | | outer membrane protein | No match found | Porin | No match found |
|  | HP TPASS_0325 | **B2S2S2** | outer membrane protein | | outer membrane protein | Family of unknown function (DUF490) | Purine and uridine phosphorylases | Family of unknown function (DUF490) |
|  | HP TPASS_0332 | **B2S2S9** | No significant match found | | 6-phosphogluconate dehydrogenase | No match found | **BRCT domain** | No match found |
|  | HP TPASS_0333 | **B2S2T0** | outer membrane lipoprotein carrier protein LolA | | No significant match found | outer membrane lipoprotein carrier protein LolA | Outer membrane lipoprotein carrier protein LolA | Outer membrane lipoprotein carrier protein LolA |
|  | HP TPASS_0334 | **B2S2T1** | DNA-binding helix-turn-helix protein, transcriptional regulator | | rod shape-determining protein | lambda repressor-like DNA-binding domain | HTH_XRE family  DNA binding protein | DNA-binding domain |
|  | HP TPASS_0335 | **B2S2T2** | CAAX amino terminal protease | | No significant match found | CAAX protease self-immunity | Abi domain  (CAAX protease self-immunity) | CAAX protease self-immunity |
|  | HP TPASS_0338 | **B2S2T5** | No significant match found | | dimethyladenosine transferase | Mid2 like cell wall stress sensor | GHMP Kinase, C-terminal domain | No match found |
|  | HP TPASS_0339 | **B2S2T6** | RluA family pseudouridine synthase | | tRNA pseudouridine synthase A | RNA pseudouridylate synthase | RNA pseudouridylate synthase | Pseudouridine synthase, RluC/RluD |
|  | HP TPASS_0346 | **B2S2U3** | Lipoprtein | | phospho-N-acetylmuramoyl-pentapeptide-transferase | Protein of unknown function DUF2715 | OMPA-like  (Outer membrane protein) | Protein of unknown function DUF2715 |
|  | HP TPASS_0347 | **B2S2U4** | Lipoprtein | | phospho-N-acetylmuramoyl-pentapeptide-transferase | Protein of unknown function DUF2715 | DNA-binding domain | Protein of unknown function DUF2715 |
|  | HP TPASS_0348 | **B2S2U5** | heptaprenyl diphosphate synthase | | peptidyl-prolyl cis-trans isomerase | Heptaprenyl diphosphate synthase | Heptaprenyl diphosphate synthase | Heptaprenyl diphosphate synthase component I |
|  | HP TPASS_0352 | **B2S2U9** | transcriptional Coactivator p15 | | gamma-glutamyl kinase | Transcriptional Coactivator p15 (PC4) | HydB/Nqo4-like | Transcriptional coactivator p15 |
|  | HP TPASS_0355 | **B2S2V2** | CDP-alcohol phosphatidyltransferase | | RNA-binding protein | No match found | PX domain | No match found |
|  | HP TPASS_0358 | **B2S2V5** | glycosyl hydrolase | | No significant match found | Glycosyl hydrolase | Glycosyl Hydrolase | Glycosyl hydrolase |
|  | HP TPASS_0359 | **B2S2V6** | 1-deoxy-D-xylulose 5-phosphate reductoisomerase | | No significant match found | No match found | DNA-binding domain | No match found |
|  | HP TPASS_0360 | **B2S2V7** | molecular chaperone DnaK | | No match found | Topoisomerase DNA binding C4 zinc finger | No match found | No match found |
|  | HP TPASS_0368 | **B2S2W5** | ECF subfamily RNA polymerase sigma-24 factor | | chromosome segregation protein | No match found | Fibronectin type III | No match found |
|  | HP TPASS_0369 | **B2S2W6** | tetratricopeptide repeat protein | | chromosome segregation protein | tetratricopeptide repeat protein | alpha/beta-Hydrolases | Outer membrane lipoprotein |
|  | HP TPASS_0370 | **B2S2W7** | No significant match found | |  | Rhabdovirus spike glycoprotein | Armadillo repeat | No match found |
|  | HP TPASS_0371 | **B2S2W8** | 4-(cytidine 5'-diphospho)-2-C-methyl-D-erythritol kinase | | 4-diphosphocytidyl-2-C-methyl-D-erythritol kinase | 4-(cytidine 5'-diphospho)-2-C-methyl-D-erythritol kinase | 4-diphosphocytidyl-2-C-methyl-D-erythritol kinase | 4-diphosphocytidyl-2-C-methyl-D-erythritol kinase |
|  | HP TPASS_0373 | **B2S2X0** | tRNA(Ile)-lysidine synthase | | lysidine synthase | tRNA-Ile-lysidine synthetase | tRNA(Ile)-lysidine synthase | Lysidine-tRNA(Ile) synthetase |
|  | HP TPASS_0374 | **B2S2X1** | Tetratricopeptide repeat | | 50S ribosomal protein | Tetratricopeptide repeat | Protein prenylyltransferase | Tetratricopeptide-like helical |
|  | HP TPASS_0375 | **B2S2X2** | No significant match found | | Ligates lysine onto the cytidine present at position 34 of the AUA codon | No match found | Homeodomain-like | No match found |
|  | HP TPASS_0376 | **B2S2X3** | Lipoprotein | | flagellar protein | No match found | hiamin diphosphate-binding fold (THDP-binding) | No match found |
|  | HP TPASS_0377 | **B2S2X4** | flagellar basal body-associated protein FliL | | flagellar protein | No match found | PreATP-grasp domain | No match found |
|  | HP TPASS_0381 | **B2S2X8** | Membrane protein | | UDP-N-acetylmuramoylalanyl-D-glutamyl-2, 6-diaminopimelate--D-alanyl-D-alanine | Integral membrane protein | Integral membrane protein | Membrane bound protein |
|  | HP TPASS_0382 | **B2S2X9** | No significant match found | | cell division protein | No match found | No match found | No match found |
|  | HP TPASS_0384 | **B2S2Y1** | S-adenosyl-methyltransferase | | Exhibits S-adenosyl-L-methionine-dependent methyltransferase activity | 16S rRNA (cytosine(1402)-N(4))-methyltransferase | Methyltransferase | Ribosomal RNA small subunit methyltransferase H |
|  | HP TPASS_0385 | **B2S2Y2** | cell division protein FtsL | | cell division protein | Cell division protein FtsL  (Septum formation initiator) | ABC transporter involved in vitamin B12 uptake, BtuC | No match found |
|  | HP TPASS_0392 | **B2S2Y9** | tetratricopeptide repeat protein | | cell division protein | tetratricopeptide repeat protein | tetratricopeptide repeat protein | Tetratricopeptide repeat-containing domain |
|  | HP TPASS_0404 | **B2S301** | metallo-beta-lactamase | | glutamate racemase | Metallo-beta-lactamase | Metallo-hydrolase/oxidoreductase | Beta-lactamase |
|  | HP TPASS_0408 | **B2S305** | chromosome segregation ATPase domain protein | | ankyrin | Apolipoprotein | Homeodomain-like | No match found |
|  | HP TPASS_0409 | **B2S306** | No significant match found | | phosphoglycolate phosphatase | No match found | Cytochrome P450 | No match found |
|  | HP TPASS_0412 | **B2S309** | PUR-alpha/beta/gamma DNA/RNA-binding protein | | phosphoglucomutase | PurA ssDNA and RNA-binding protein | DNA/RNA-binding protein | No match found |
|  | HP TPASS_0415 | **B2S312** | pyrroline-5-carboxylate synthetase-like protein | | D-alanine glycine permease | No match found | Cobalamin (vitamin B12)-dependent enzymes | No match found |
|  | HP TPASS_0420 | **B2S317** | acid phosphatase | | survival protein | No match found | Terpenoid synthases | No match found |
|  | HP TPASS_0421 | **B2S318** | tetratricopeptide repeat protein | | transcription factor | tetratricopeptide repeat protein | TPR-like | Tetratricopeptide repeat-containing domain |
|  | HP TPASS_0422 | **B2S319** | tyrosine phosphatase | | No match found | No match found | Formate dehydrogenase/DMSO reductase, domains 1-3 | No match found |
|  | HP TPASS_0423 | **B2S320** | peptide ABC transporter ATPase | | No match found | P-loop containing nucleotide triphosphate hydrolases | P-loop containing nucleoside triphosphate hydrolases | P-loop containing nucleoside triphosphate hydrolase |
|  | HP TPASS_0425 | **B2S322** | GTP cyclohydrolase | | V-type ATP synthase subunit E | No match found | Heme-dependent peroxidases | No match found |
|  | HP TPASS_0431 | **B2S328** | pantothenate kinase, type III | | lipopolysaccharide core biosynthesis protein | Type III pantothenate kinase | Type III pantothenate kinase | Type III pantothenate kinase |
|  | HP TPASS_0432 | **B2S329** | serine/threonine protein kinase Sgk2 | | Catalyzes the phosphorylation of pantothenate | No match found | Golgi alpha-mannosidase II | No match found |
|  | HP TPASS_0436 | **B2S332** | Phosphoesterase | | DNA polymerase I | Phosphoesterase | DHH superfamily protein, subfamily 1 | Phosphoesterase, DHHA1 |
|  | HP TPASS_0437 | **B2S333** | multidrug resistance outer membrane protein | | No match found | No match found | HAD-like | No match found |
|  | HP TPASS_0438 | **B2S334** | RdgB/HAM1 family non-canonical purine NTP pyrophosphatase | | nucleoside-diphosphate kinase | Phosphatase | Nucleoside-triphosphatase | Non-canonical purine NTP pyrophosphatase |
|  | HP TPASS_0441 | **B2S337** | Inorganic polyphosphate/ATP-NAD kinase | | NAD synthetase | ATP-NAD kinase | Inorganic polyphosphate/ATP-NAD kinase | Inorganic polyphosphate/ATP-NAD kinase |
|  | HP TPASS_0443 | **B2S339** | transcriptional regulator | | DNA repair protein | No match found | Lipoxigenase | No match found |
|  | HP TPASS_0444 | **B2S340** | LysM/M23/M37 peptidase | | DNA repair protein | Peptidase | LysM peptidase | Peptidase M23 |
|  | HP TPASS_0447 | **B2S343** | tetratricopeptide repeat protein | | 4-hydroxy-3-methylbut-2-en-1-yl diphosphate synthase | tetratricopeptide repeat protein | prenylyltransferase | tetratricopeptide repeat protein |
|  | HP TPASS_0449 | **B2S345** | tetratricopeptide repeat protein | | uracil phosphoribosyltransferase | tetratricopeptide repeat protein | tetratricopeptide repeat protein | tetratricopeptide repeat protein |
|  | HP TPASS_0451 | **B2S347** | No significant match found | | uracil phosphoribosyltransferase | No match found | No match found | No match found |
|  | HP TPASS_0453 | **B2S349** | sugar ABC transporter substrate-binding protein | | isoleucyl-tRNA synthetas | No match found | No match found | No match found |
|  | HP TPASS_0454 | **B2S350** | type IV pilus assembly PilZ | | No match found | No match found | CheY-like | No match found |
|  | HP TPASS_0455 | **B2S351** | No significant match found | | transcription-repair coupling factor | No match found | Fibronectin type III | No match found |
|  | HP TPASS_0456 | **B2S352** | No significant match found | | 50S ribosomal protein | No match found | Clavaminate synthase-like | No match found |
|  | HP TPASS_0457 | **B2S353** | DNA polymerase III subunit delta | | No match found | No match found | No match found | No match found |
|  | HP TPASS_0458 | **B2S354** | segregation and condensation protein | | chromosome segregation protein | segregation and condensation protein | Segregation and condensation protein | Segregation and condensation protein |
|  | HP TPASS_0459 | **B2S355** | RsuA family pseudouridine synthase | | GTP-binding protein EngA | RNA pseudouridylate synthase | Zinc finger domain | Pseudouridine synthase, RsuA/RluB/E/F |
|  | HP TPASS_0460 | **B2S356** | tetratricopeptide repeat protein | | No match found | tetratricopeptide repeat protein | tetratricopeptide repeat protein | tetratricopeptide repeat protein |
|  | HP TPASS_0461 | **B2S357** | transcriptional regulator | | No match found | lambda repressor-like DNA-binding domain | HTH_XRE domain  (DNA binding) | lambda repressor-like DNA-binding domain |
|  | HP TPASS_0462 | **B2S358** | Outer membrane protein | | No match found | No match found | Subtilisin-like | No match found |
|  | HP TPASS_0463 | **B2S359** | Outer membrane protein | | No match found | No match found | Heme-dependent peroxidases | No match found |
|  | HP TPASS_0464 | **B2S360** | tRNA (guanine-N(7)-)-methyltransferase | | Catalyzes the formation of N(7)-methylguanine at position 46 (m7G46) in tRNA | S-adenosyl-L-methionine-dependent methyltransferase | tRNA (guanine-N(7)-)-methyltransferase | tRNA (guanine-N(7)-)-methyltransferase |
|  | HP TPASS_0465 | **B2S361** | peptidase M23 | | Catalyzes the formation of N(7)-methylguanine at position 46 (m7G46) in tRNA | No match found | Pleckstrin-homology domain (PH domain) | No match found |
|  | HP TPASS_0466 | **B2S362** | No significant match found | | No match found | No match found | "Helical backbone" metal receptor | No match found |
|  | HP TPASS_0467 | **B2S363** | No significant match found | | No match found | No match found | Clavaminate synthase-like | No match found |
|  | HP TPASS_0468 | **B2S364** | tetratricopeptide repeat protein | | periplasmic serine protease | tetratricopeptide repeat protein | tetratricopeptide repeat protein | tetratricopeptide repeat protein |
|  | HP TPASS_0470 | **B2S365** | tetratricopeptide repeat protein | | No match found | tetratricopeptide repeat protein | tetratricopeptide repeat protein | tetratricopeptide repeat protein |
|  | HP TPASS_0471 | **B2S366** | tetratricopeptide repeat protein | | apolipoprotein N-acyltransferase | tetratricopeptide repeat protein | tetratricopeptide repeat protein | tetratricopeptide repeat protein |
|  | HP TPASS_0473 | **B2S368** | ribose/galactose ABC transporter permease | | excinuclease ABC subunit C | Positive regulator of sigma(E), RseC/MucC | Immunoglobulin | No match found |
|  | HP TPASS_0474 | **B2S369** | YebC/PmpR family DNA-binding regulatory protein | | aspartyl-tRNA synthetase | DNA-binding regulatory protein, YebC/PmpR family | YebC-like | transcriptional regulatory protein |
|  | HP TPASS_0479 | **B2S374** | ABC transporter ATP-binding protein | | glucose-6-phosphate 1-dehydrogenase | Protein of unknown function (DUF2715) | Nucleotide-binding domain | Protein of unknown function (DUF2715) |
|  | HP TPASS_0480 | **B2S375** | Membrane protein | | No match found | No match found | Pyruvoyl dependent aspartate decarboxylase | No match found |
|  | HP TPASS_0481 | **B2S376** | chromosome segregation ATPase | | No match found | Divergent CCT motif | beta and beta-prime subunits of DNA dependent RNA-polymerase | No match found |
|  | HP TPASS_0482 | **B2S377** | No significant match found | | No match found | CHASE3 domain | 4-helical cytokine | No match found |
|  | HP TPASS_0484 | **B2S379** | FecR protein | | fibronectin-binding protein | FecR protein | FecR protein | FecR protein |
|  | HP TPASS_0487 | **B2S382** | phosphoribosylaminoimidazole-succinocarboxamide synthase | | antigen, p83/100 | Quinoprotein alcohol dehydrogenase | Tricorn protease N-terminal domain | Quinonprotein alcohol dehydrogenase |
|  | HP TPASS_0489 | **B2S384** | Metallo-beta-lactamase family protein | | RNA polymerase sigma factor RpoD | Metallo-beta-lactamase | Metallo-beta-lactamase | Beta-lactamase-like |
|  | HP TPASS_0490 | **B2S385** | antirepressor protein | | No match found | No match found | Formate/glycerate dehydrogenase catalytic domain-like | No match found |
|  | HP TPASS_0491 | **B2S386** | aminodeoxychorismate lyase | | RNA polymerase sigma factor | YceG family protein | YceG family protein | YceG family protein |
|  | HP TPASS_0494 | **B2S389** | zinc ribbon domain | | DNA primase | zinc ribbon domain | L domain-like | Putative zinc ribbon domain |
|  | HP TPASS_0496 | **B2S390** | TPR domain-containing protein | | rod shape-determining protein | tetratricopeptide repeat protein | tetratricopeptide repeat protein | TPR domain-containing protein |
|  | HP TPASS_0502 | **B2S396** | ankyrin repeat protein | | ankyrin | ankyrin repeat protein | ankyrin repeat protein | Ankyrin repeat-containing domain |
|  | HP TPASS_0503 | **B2S397** | Uridine kinases | | rod shape-determining protein | No match found | DHH phosphoesterases | No match found |
|  | HP TPASS_0504 | **B2S398** | No significant match found | | No match found | No match found | No match found | No match found |
|  | HP TPASS_0512 | **B2S3A6** | bifunctional 2-C-methyl-D-erythritol 4-phosphate cytidylyltransferase/2-C-methyl-D-erythritol 2,4-cyclodiphosphate synthase | | 2-C-methyl-D-erythritol from CTP and 2-C-methyl- D-erythritol 4-phosphate | 2-C-methyl-D-erythritol 2,4-cyclodiphosphate synthase | Bifunctional enzyme IspD/ispF | 2-C-methyl-D-erythritol 2,4-cyclodiphosphate synthase |
|  | HP TPASS_0515 | **B2S3A9** | Organic solvent tolerance protein | | excinuclease ABC subunit A | Organic solvent tolerance protein | Organic solvent tolerance protein | Organic solvent tolerance protein |
|  | HP TPASS_0518 | **B2S3B2** | thiamine pyrophosphokinase | | phosphomethypyrimidine kinase | Thiamin pyrophosphokinase | Thiamine pyrophosphokinase | Thiamine pyrophosphokinase |
|  | HP TPASS_0522 | **B2S3B5** | colicin V production CvpA | | undecaprenyldiphospho-muramoylpentapeptide beta-N-acetylglucosaminyltransferase | Colicin V production protein | Colicin V production protein | Colicin V production protein |
|  | HP TPASS_0534 | **B2S3C6** | vacuolar-type ATPase subunit C | | V-type ATPase, subunit I | V-type ATP synthase subunit C | Thymidylate synthase/dCMP hydroxymethylase | ATPase, V0 complex, c/d subunit |
|  | HP TPASS_0535 | **B2S3C7** | E3 ubiquitin-protein ligase | | No match found | No match found | No match found | No match found |
|  | HP TPASS_0539 | **B2S3D1** | RTA-like protein | | phosphoglycerate kinase | No match found | No match found | No match found |
|  | HP TPASS_0544 | **B2S3D6** | endonuclease/exonuclease/phosphatase family protein | | methylgalactoside ABC transporter, periplasmic galactose-binding protein | Endonuclease/Exonuclease/phosphatase | Endonuclease/Exonuclease/phosphatase | Endonuclease/exonuclease/phosphatase |
|  | HP TPASS_0548 | **B2S3E0** | Outer membrane protein transport protein | | penicillin tolerance protein | Outer membrane protein transport protein | TPR-like | Outer membrane protein transport protein |
|  | HP TPASS_0552 | **B2S3E4** | foldase PrsA | | phosphatase | Matrix protein (MA), p15 | Ribosomal protein L13 | No match found |
|  | HP TPASS_0553 | **B2S3E5** | Membrane protein | | phosphoglycolate phosphatase | Domain of unknown function DUF20 | Domain of unknown function DUF20 | No match found |
|  | HP TPASS_0557 | **B2S3E9** | ABC transporter substrate-binding protein | | membrane protein | Protein of unknown function DUF1007 | EF-hand | Protein of unknown function DUF1007 |
|  | HP TPASS_0558 | **B2S3F0** | NiCoT family nickel (Ni2+)-cobalt (Co2+) transporter | | No match found | High-affinity nickel-transport protein | High-affinity nickel-transport protein | Nickel/cobalt transporter, high-affinity |
|  | HP TPASS_0561 | **B2S3F3** | beta-propeller domain of methanol dehydrogenase type protein | | flagellar biosynthetic protein | TLP18.3, Psb32 and MOLO-1 founding proteins of phosphatase | No match found | No match found |
|  | HP TPASS_0563 | **B2S3F5** | DnaJ domain-containing protein | | spore coat polysaccharide biosynthesis protein | DnaJ domain | DnaJ molecular chaperon homology domain | DnaJ molecular chaperon homology domain |
|  | HP TPASS_0564 | **B2S3F6** | No significant match found | | spore coat polysaccharide biosynthesis protein | No match found | E2 regulatory, transactivation domain | No match found |
|  | HP TPASS_0565 | **B2S3F7** | GDSL-like lipase/acylhydrolase domain-containing protein | | alginate O-acetylation protein | Protein of unknown function (DUF459) | SGNH hydrolase | Protein of unknown function (DUF459) |
|  | HP TPASS_0567 | **B2S3F9** | flagellar protein FlbB | | flagellar hook protein | No match found | Glyceraldehyde-3-phosphate dehydrogenase-like, C-terminal domain | No match found |
|  | HP TPASS_0570 | **B2S3G2** | Membrane protein | | No match found | TLP18.3, Psb32 and MOLO-1 founding proteins of phosphatase | Cu,Zn superoxide dismutase-like | TPM domain |
|  | HP TPASS_0572 | **B2S3G4** | FMN-binding protein | | No match found | FMN-binding domain  (Ferric reductase like transmembrane component) | FMN-binding domain  (Ferric reductase like transmembrane component) | FMN-binding domain  (Ferric reductase like transmembrane component) |
|  | HP TPASS_0573 | **B2S3G5** | retinal-specific ATP-binding cassette transporter-like | | No match found | No match found | No match found | No match found |
|  | HP TPASS_0577 | **B2S3G8** | Peptidase S8 and S53 subtilisin kexin sedolisin | | peptide chain release factor | No match found | Carboxypeptidase regulatory domain-like | No match found |
|  | HP TPASS_0579 | **B2S3H0** | No significant match found | | integral membrane protein | No match found | Argininosuccinate synthetase | No match found |
|  | HP TPASS_0580 | **B2S3H1** | MacB-like periplasmic core domain protein | | ABC transporter | FtsX-like permease family | Lipoprotein releasing system, permease protein | Permease FtsX-like |
|  | HP TPASS_0582 | **B2S3H3** | MacB-like periplasmic core domain protein | | integral membrane protein | FtsX-like permease family | FtsX-like permease family | Permease FtsX-like |
|  | HP TPASS_0583 | **B2S3H4** | ubiquitin C-terminal hydrolase 3 | | No match found | No match found | No match found | No match found |
|  | HP TPASS_0584 | **B2S3H5** | glutamate-1-semialdehyde aminotransferase | | oligopeptide ABC transporter | No match found | alpha/beta-Hydrolase | No match found |
|  | HP TPASS_0587 | **B2S3H8** | DNA-directed DNA polymerase III delta subunit | | phosphocarrier protein HPr | No match found | post-AAA+ oligomerization | No match found |
|  | HP TPASS_0588 | **B2S3H9** | DNA-directed DNA polymerase III delta subunit | | DNA polymerase III subunits gamma and tau | DNA polymerase III, delta subunit | DNA polymerase III, delta subunit | DNA polymerase III, delta subunit |
|  | HP TPASS_0590 | **B2S3I1** | ribosomal protein | | No match found | No match found | Ribosomal protein L36 (RpmJ-2) | No match found |
|  | HP TPASS_0592 | **B2S3I3** | serine-type D-Ala-D-Ala carboxypeptidase | | No match found | D-alanyl-D-alanine carboxypeptidase | (Trans)glycosidase | D-alanyl-D-alanine carboxypeptidase |
|  | HP TPASS_0593 | **B2S3I4** | 3-oxoacyl-[acyl-carrier protein] reductase | | No match found | No match found | Actin-like ATPase | No match found |
|  | HP TPASS_0594 | **B2S3I5** | signal peptide protein | | adenylate kinase | Domain of unknown function DUF2147 | DNA/RNA polymerase | Domain of unknown function DUF2147 |
|  | HP TPASS_0598 | **B2S3I8** | WD domain, G-beta repeat protein | | phosphatidate cytidylyltransferase | No match found | YVTN repeat-like/Quinoprotein amine dehydrogenase | Cytochrome cd1-nitrite reductase-like, haem d1 domain |
|  | HP TPASS_0599 | **B2S3I9** | cyclic nucleotide-binding protein | | zinc protease | CpXC protein | Zinc finger domain | Zinc finger domain |
|  | HP TPASS_0607 | **B2S3J7** | No significant match found | | 30S ribosomal protein S2 | No match found | Trypsin-like serine protease | No match found |
|  | HP TPASS_0608 | **B2S3J8** | HEAT repeat protein | | 30S ribosomal protein S2 | HEAT repeats | ARM repeat | Armadillo-type fold |
|  | HP TPASS_0612 | **B2S3K2** | Fe-S cluster assembly protein SufB | | ABC transporter, ATP-binding protein | Uncharacterized protein family (UPF0051) | Protein binding protein | SUF system FeS cluster assembly, SufB |
|  | HP TPASS_0613 | **B2S3K3** | Fe-S cluster assembly protein SufB | | ABC transporter, ATP-binding protein | Uncharacterized protein family (UPF0051) | Seven-hairpin glycosidases | SUF system FeS cluster assembly, SufD |
|  | HP TPASS_0617 | **B2S3K7** | pyrogenic exotoxin B | | No match found | Protein of unknown function (DUF2715) | Bacterial adhesin | Protein of unknown function (DUF2715) |
|  | HP TPASS_0618 | **B2S3K8** | proto-oncogene c-Rel | | No match found | Protein of unknown function (DUF2715) | DNA topoisomerase I, dispensable insert domain | Protein of unknown function (DUF2715) |
|  | HP TPASS_0619 | **B2S3K9** | Lipoprotein | | No match found | Protein of unknown function (DUF2715) | Fe,Mn superoxide dismutase | Protein of unknown function (DUF2715) |
|  | HP TPASS_0622 | **B2S3L2** | tetratricopeptide repeat protein | | No match found | tetratricopeptide repeat protein | Bacterial transcritptional activator domain | Tetratricopeptide repeat-containing domain |
|  | HP TPASS_0624 | **B2S3L4** | OmpA family protein | | membrane-bound lytic murein transglycosylase D | OmpA family | OmpA family protein | Outer membrane protein, OmpA/MotB, C-terminal |
|  | HP TPASS_0625 | **B2S3L5** | tetratricopeptide repeat protein | | nicotinate phosphoribosyltransferase | tetratricopeptide repeat protein | Protein prenylyltransferase | No match found |
|  | HP TPASS_0629 | **B2S3L9** | 2-dehydro-3-deoxygluconokinase | | flagellar basal body rod modification protein | No match found | Cysteine proteinase | No match found |
|  | HP TPASS_0636 | **B2S3M5** | DNA repair protein RecO | | recombination protein RecR  (DNA repair) | Recombination protein O C terminal | beta-carbonic anhydrase | No match found |
|  | HP TPASS_0638 | **B2S3M7** | Na+-driven multidrug efflux pump | | tRNA delta(2)-isopentenylpyrophosphate transferase | Membrane MotB of proton-channel complex MotA/MotB | No match found | No match found |
|  | HP TPASS_0645 | **B2S3N4** | No significant match found | | lysyl-tRNA synthetase | No match found | Thiamin diphosphate-binding fold | No match found |
|  | HP TPASS_0646 | **B2S3N5** | NHL repeat containing protein | | seryl-tRNA synthetase | NHL repeat | (Trans)glycosidases | Six-bladed beta-propeller, TolB-like |
|  | HP TPASS_0648 | **B2S3N7** | Tetratricopeptide TPR_1 repeat-containing protein | | metalloprotease | Tetratricopeptide repeat containing protein | Tetratricopeptide repeat containing protein | Tetratricopeptide repeat containing protein |
|  | HP TPASS_0651 | **B2S3P0** | 7TM receptor with intracellular metal dependent phosphohydrolase | | metalloprotease | 7TM receptor with intracellular HD hydrolase | 7TM receptor with intracellular HD hydrolase | Metal-dependent phosphohydrolase, 7TM intracellular domain |
|  | HP TPASS_0656 | **B2S3P5** | Gamma-aminobutyrate:alpha-ketoglutarate aminotransferase | | No match found | No match found | Periplasmic binding protein-like II | No match found |
|  | HP TPASS_0661 | **B2S3Q0** | golgi trafficking protein GRIP | | flagellar hook-associated protein | FlgN-like | No match found | No match found |
|  | HP TPASS_0665 | **B2S3Q4** | peptidase T | | uridine kinase | No match found | Acyl-CoA N-acyltransferase | No match found |
|  | HP TPASS_0666 | **B2S3Q5** | ABC multidrug transporter | | No match found | No match found | Subdomain of clathrin and coatomer appendage domain | No match found |
|  | HP TPASS_0668 | **B2S3Q7** | YGGT family protein | | DNA polymerase III subunit alpha | YGGT family | YGGT family | No match found |
|  | HP TPASS_0674 | **B2S3R3** | Smr domain protein | | glutamyl-tRNA synthetase | Smr domain | Smr domain | Smr protein/MutS2 C-terminal |
|  | HP TPASS_0675 | **B2S3R4** | GumN protein | | No match found | TraB family | GumN protein | Pheromone shutdown, TraB |
|  | HP TPASS_0676 | **B2S3R5** | carbon monoxide dehydrogenase | | No match found | No match found | P-loop containing nucleoside triphosphate hydrolase | No match found |
|  | HP TPASS_0677 | **B2S3R6** | RIO1-domain-containing protein | | No match found | No match found | Protein kinase | No match found |
|  | HP TPASS_0678 | **B2S3R7** | glutamate-1-semialdehyde aminotransferase | | No match found | No match found | PLP-dependent transferase | No match found |
|  | HP TPASS_0679 | **B2S3R8** | C-C motif chemokine 20 isoform 1 precursor | | No match found | 7TM diverse intracellular signalling | Cyclin-like | No match found |
|  | HP TPASS_0690 | **B2S3S9** | No significant match found | | recombinase A | No match found | Subtilisin-like | No match found |
|  | HP TPASS_0691 | **B2S3T0** | ScpA/B protein | | chromosomal partition during cell division | ScpA/B protein | ScpA/B protein | Prokaryotic chromosome segregation/condensation protein ScpA |
|  | HP TPASS_0693 | **B2S3T2** | O-succinylbenzoate-CoA ligase | | 5,10-methenyltetrahydrofolate synthetase | No match found | Substrate-binding domain of HMG-CoA reductase | No match found |
|  | HP TPASS_0697 | **B2S3T6** | 2-polyprenylphenol hydroxylase | | nicotinamidase | Protein of unknown function (DUF2715) | FAD-linked oxidase | Protein of unknown function (DUF2715) |
|  | HP TPASS_0698 | **B2S3T7** | Lipoprotein | | No match found | Protein of unknown function (DUF2715) | Acyl-CoA dehydrogene | Protein of unknown function (DUF2715) |
|  | HP TPASS_0699 | **B2S3T8** | No significant match found | | No match found | MerR HTH family regulatory protein | DNA-binding domain | No match found |
|  | HP TPASS_0700 | **B2S3T9** | UBA/THIF-type NAD/FAD binding protein | | DNA-directed RNA polymerase | No match found | Metallo-hydrolase/oxidoreductase | No match found |
|  | HP TPASS_0702 | **B2S3U1** | M23B subfamily peptidase | | single-stranded-DNA-specific exonuclease | Peptidase family M23 | Peptidase M23 | Peptidase M23 |
|  | HP TPASS_0703 | **B2S3U2** | M23B subfamily peptidase | | single-stranded-DNA-specific exonuclease | No match found | Clavaminate synthase | No match found |
|  | HP TPASS_0706 | **B2S3U5** | M23/M37 peptidase domain-containing protein | | carboxyl-terminal protease | Peptidase family M23 | Peptidase M23 | Peptidase M23 |
|  | HP TPASS_0707 | **B2S3U6** | No significant match found | | RNA polymerase sigma factor WhiG | No match found | "Helical backbone" metal receptor | No match found |
|  | HP TPASS_0708 | **B2S3U7** | rhomboid family protein | | RNA polymerase sigma factor WhiG | Protein of unknown function (DUF3918) | Enolase C-terminal domain | No match found |
|  | HP TPASS_0710 | **B2S3U9** | polymerase | | RNA polymerase sigma factor WhiG | Jag N-terminus | JagN domain | Jag N-terminus |
|  | HP TPASS_0711 | **B2S3V0** | ABC transporter-like protein | | No match found | Protein of unknown function (DUF1128) | Ribonuclease H | No match found |
|  | HP TPASS_0719 | **B2S3V8** | Flagellar biosynthesis protein, FliO | | Flagellar biosynthesis protein | Flagellar biosynthesis protein, FliO | Flagellar biosynthesis protein, FliO | Flagellar biosynthesis protein, FliO |
|  | HP TPASS_0723 | **B2S3W2** | ribosomal RNA large subunit methyltransferase | | No match found | No match found | No match found | No match found |
|  | HP TPASS_0730 | **B2S3W9** | CDP-diacylglycerol--glycerol-3-phosphate 3-phosphatidyltransferase | | CDP-diacylglycerol--glycerol-3-phosphate 3-phosphatidyltransferase | CDP-alcohol phosphatidyltransferase | CDP-alcohol phosphatidyltransferase | CDP-alcohol phosphatidyltransferase |
|  | HP TPASS_0731 | **B2S3X0** | NUDIX hydrolase | | treponemal aqueous protein | NUDIX domain | NUDIX domain | NUDIX hydrolase domain |
|  | HP TPASS_0733 | **B2S3X2** | glucan synthase-like protein | | methylenetetrahydrofolate dehydrogenase | Outer membrane protein beta-barrel domain | OMPA-like | Outer membrane protein/outer membrane enzyme PagP , beta-barrel |
|  | HP TPASS_0738 | **B2S3X7** | iojap-like ribosome-associated protein | | 50S ribosomal protein | Iojap/YbeB-like | No match found | Protein Iojap/ribosomal silencing factor RsfS |
|  | HP TPASS_0739 | **B2S3X8** | LytR family transcriptional regulator | | No match found | LytR cell envelope-related transcriptional attenuator | cell envelope-related transcriptional attenuator domain | Cell envelope-related transcriptional attenuator |
|  | HP TPASS_0740 | **B2S3X9** | nicotinate-nucleotide adenylyltransferase | | reversible adenylation of nicotinate mononucleotide | HD domain | Metal dependent phosphohydrolases with conserved 'HD' moti | Metal dependent phosphohydrolases with conserved 'HD' moti |
|  | HP TPASS_0741 | **B2S3Y0** | nicotinate-nucleotide adenylyltransferase | | reversible adenylation of nicotinate mononucleotide | nicotinate (nicotinamide) nucleotide adenylyltransferase | nicotinate-nucleotide adenylyltransferase | nicotinate-nucleotide adenylyltransferase |
|  | HP TPASS_0744 | **B2S3Y3** | Ribosomal protein | | 50S ribosomal protein | Protein of unknown function DUF464 | FMN-dependent nitroreductase | Protein of unknown function DUF464 |
|  | HP TPASS_0747 | **B2S3Y6** | RNA modification GTPase TrmE | | pyruvate phosphate dikinase | No match found | GroEL equatorial domain | No match found |
|  | HP TPASS_0749 | **B2S3Y8** | RNA polymerase beta subunit | | cytoplasmic filament protein A | No match found | (Trans)glycosidases | No match found |
|  | HP TPASS_0750 | **B2S3Y9** | von Willebrand factor A | | laminin-binding protein | von Willebrand factor type A domain | von Willebrand factor type A domain | von Willebrand factor, type A |
|  | HP TPASS_0752 | **B2S3Z1** | sporulation and cell division repeat protein | | methylthiolation of N6- (dimethylallyl)adenosine | Sporulation related domain | Prim-pol domain | No match found |
|  | HP TPASS_0753 | **B2S3Z2** | major facilitator transporter | | PTS system, nitrogen regulatory IIA component | Nucleoside transporter | TRADD, N-terminal domain | No match found |
|  | HP TPASS_0759 | **B2S3Z8** | No significant match found | | penicillin-binding protein | No match found | No match found | No match found |
|  | HP TPASS_0761 | **B2S400** | No significant match found | | penicillin-binding protein | Protein of unknown function DUF115 | No match found | No match found |
|  | HP TPASS_0762 | **B2S401** | No significant match found | | penicillin-binding protein | sialyltransferase cstii | PLP-dependent transferase | No match found |
|  | HP TPASS_0763 | **B2S402** | TPR repeat-containing response regulator | | No match found | Tetratricopeptide repeat | No match found | No match found |
|  | HP TPASS_0764 | **B2S403** | HD domain-containing protein | | S-adenosylmethionine synthetase | HD domain | Metal dependent phosphohydrolases with conserved 'HD' motif | Metal dependent phosphohydrolases with conserved 'HD' motif |
|  | HP TPASS_0766 | **B2S405** | No significant match found | | elongation factor G | Mitochondrial fission regulator | (Trans)glycosidases | No match found |
|  | HP TPASS_0771 | **B2S410** | Na/Pi cotransporter family protein | | sodium/proton-dependent alanine transporter | Na+/Pi-cotransporter | Na+/Pi-cotransporter | Sodium-dependent phosphate transport protein |
|  | HP TPASS_0772 | **B2S411** | LysR family transcriptional regulator | | integral membrane protein | FliG N-terminal domain | No match found | No match found |
|  | HP TPASS_0776 | **B2S415** | phosphoribosyltransferase | | No match found | Phosphoribosyl transferase domain | Phosphoribosyltransferases | No match found |
|  | HP TPASS_0777 | **B2S416** | histidyl-tRNA synthetase | | No match found | Zinc finger, ZZ type | DNA/RNA polymerase | No match found |
|  | HP TPASS_0781 | **B2S420** | peptidase M23 | | NAD synthetase | LAGLIDADG-like domain | No match found | No match found |
|  | HP TPASS_0782 | **B2S421** | peptidase M23 | | No match found | Peptidase family M23 | No match found | No match found |
|  | HP TPASS_0783 | **B2S422** | Pyrrolo-quinoline quinone | | No match found | No match found | No match found | Quinoprotein amine dehydrogenase, beta chain-like |
|  | HP TPASS_0784 | **B2S423** | LPS ABC transporter, LPS-binding protein LptC | | ABC transporter | Lipopolysaccharide-assembly, LptC-related | FAD-linked reductase | Lipopolysaccharide assembly, LptC-related |
|  | HP TPASS_0785 | **B2S424** | OstA-like protein | | ABC transporter | OstA-like protein | OstA-like protein | Organic solvent tolerance-like, N-terminal |
|  | HP TPASS_0787 | **B2S426** | cation diffusion facilitator family transporter | | No match found | No match found | Galactose-binding domain | No match found |
|  | HP TPASS_0788 | **B2S427** | SPFH domain-containing protein | | No match found | No match found | No match found | No match found |
|  | HP TPASS_0789 | **B2S428** | sigma E regulatory protein MucB/RseB | | No match found | No match found | No match found | No match found |
|  | HP TPASS_0791 | **B2S430** | tRNA (Guanine37-N(1)-) methyltransferase | | flagellar filament 33 kDa core protein | No match found | (Trans)glycosidases | No match found |
|  | HP TPASS_0793 | **B2S432** | No significant match found | | flagellar filament 33 kDa core protein | No match found | ARM repeat | No match found |
|  | HP TPASS_0795 | **B2S434** | No significant match found | | S-adenosylmethionine synthetase | No match found | No match found | No match found |
|  | HP TPASS_0796 | **B2S435** | thiamine biosynthesis protein ApbE | | conversion of aminoimidazole ribotide (AIR), a purine intermediate, to the 4-amino-5-hydroxymethyl-2- methyl pyrimidine (HMP) moiety of thiamine | ApbE family  (Thiamine biosynthesis lipoprotein) | Thiamine biosynthesis lipoprotein ApbE | Thiamine biosynthesis lipoprotein ApbE |
|  | HP TPASS_0799 | **B2S438** | lytic transglycosylase subunit | | methionyl-tRNA synthetase | No match found | No match found | No match found |
|  | HP TPASS_0802 | **B2S441** | glycerol kinase protein | | ATP-dependent Clp protease, subunit A | No match found | No match found | No match found |
|  | HP TPASS_0803 | **B2S442** | DHH family phosphoesterase | | sugar ABC transporter, ATP-binding protein | DHH phosphoesterases | DHH phosphoesterases | Phosphoesterase, RecJ-like |
|  | HP TPASS_0811 | **B2S450** | No significant match found | | No match found | No match found | No match found | No match found |
|  | HP TPASS_0813 | **B2S451** | small-conductance mechanosensitive channel | | thioredoxin reductase | No match found | Mitochondrial glycoprotein MAM33-like | No match found |
|  | HP TPASS_0815 | **B2S453** | acetyltransferase, GNAT family | | thioredoxin reductase | Acetyltransferase (GNAT) family | Acetyltransferase (GNAT) family | Acyl-CoA N-acyltransferas |
|  | HP TPASS_0816 | **B2S454** | plasma membrane sodium ion/proton antiporter Sod2 | | No match found | No match found | UBC-like | No match found |
|  | HP TPASS_0818 | **B2S456** | PTS fructose transporter subunit IIA, partial | | reversible conversion of 2- phosphoglycerate into phosphoenolpyruvate | No match found | Caspase-like | No match found |
|  | HP TPASS_0820 | **B2S458** | Tetratricopeptide repeat containing protein | | ribonuclease Z | Tetratricopeptide repeat containing protein | Tetratricopeptide repeat containing protein | Tetratricopeptide repeat containing protein |
|  | HP TPASS_0822 | **B2S460** | MscS family small conductance mechanosenstive ion channel | | desulfoferrodoxin | Mechanosensitive ion channel | Mechanosensitive ion channel | Mechanosensitive ion channel MscS, C-terminal |
|  | HP TPASS_0825 | **B2S463** | No significant match found | | tRNA pseudouridine synthase A | No match found | No match found | No match found |
|  | HP TPASS_0826 | **B2S464** | Membrane protein | | 4'-phosphopantetheinyl transferase | DisA bacterial checkpoint controller nucleotide-binding | DisA bacterial checkpoint controller nucleotide-binding | DNA integrity scanning protein, DisA, N-terminal |
|  | HP TPASS_0827 | **B2S465** | YbbR-like protein | | 4'-phosphopantetheinyl transferase | YbbR-like protein | YbbR-like protein | YbbR-like protein |
|  | HP TPASS_0829 | **B2S467** | Membrane protein | | tRNA pseudouridine synthase A | Protein of unknown function DUF2225 | RNA 3'-terminal phosphate cyclase | Protein of unknown function DUF2225 |
|  | HP TPASS_0832 | **B2S470** | sporulation/spore germination protein | | arginyl-tRNA synthetase | Sporulation and spore germination | Sporulation and spore germination | Sporulation and spore germination |
|  | HP TPASS_0833 | **B2S471** | LPXTG-motif cell wall anchor domain-containing protein | | No match found | No match found | No match found | No match found |
|  | HP TPASS_0836 | **B2S474** | pilus modification protein PilQ | | threonyl-tRNA synthetase | No match found | Nucleoside hydrolase | No match found |
|  | HP TPASS_0839 | **B2S477** | transposase | | integral membrane protein | Domain of unknown function (DUF4098) | Ferritin-like | No match found |
|  | HP TPASS_0840 | **B2S478** | MFS transporter | | integral membrane protein | MFS general substrate transport | ARM repeat | Major facilitator superfamily domain, general substrate transporter |
|  | HP TPASS_0845 | **B2S483** | YaiI/YqxD family protein | | 50S ribosomal protein | YaiI/YqxD famil | Protein of unknown function UPF0178 | Protein of unknown function UPF0178 |
|  | HP TPASS_0846 | **B2S484** | cell division protein ZapA | | 50S ribosomal protein | Cell division protein ZapA | Cell division protein ZapA | Cell division protein ZapA |
|  | HP TPASS_0847 | **B2S485** | Maf transcription factor | | 50S ribosomal protein | Protein of unknown function (DUF904) | No match found | No match found |
|  | HP TPASS_0851 | **B2S489** | No significant match found | | translation initiation factor IF-3 | Cocaine and amphetamine regulated transcript protein (CART) | Trimeric LpxA-like enzymes | Trimeric LpxA-like |
|  | HP TPASS_0854 | **B2S492** | HAMP domain-containing protein | | anti-sigma F factor antagonis | HAMP domain | HAMP linker domain | HAMP linker domain |
|  | HP TPASS_0855 | **B2S493** | Outer membrane protein | | ATP binding protein | No match found | Protein prenylyltransferase | No match found |
|  | HP TPASS_0856 | **B2S494** | Outer membrane protein | | No match found | Outer membrane protein transport protein | FYVE/PHD zinc finger | No match found |
|  | HP TPASS_0857 | **B2S495** | (E)-beta-farnesene synthase-like | | No match found | No match found | MHC antigen-recognition domain | No match found |
|  | HP TPASS_0858 | **B2S496** | Outer membrane protein | | No match found | No match found | Carbamoyl phosphate synthetase, small subunit | No match found |
|  | HP TPASS_0859 | **B2S497** | Outer membrane protein | | No match found | No match found | Serpin like | No match found |
|  | HP TPASS_0860 | **B2S498** | Tetratricopeptide repeat containing protein | | No match found | Tetratricopeptide repeat containing protein | UBC-like | No match found |
|  | HP TPASS_0864 | **B2S4A2** | LysM/M23/M37 peptidase | | hydrolysis of both 2',3'-cyclic AMP and 2',3'-cyclic GMP | Peptidase family M23  (LysM domain) | Peptidase family M23  (LysM domain) | Peptidase family M23  (LysM domain) |
|  | HP TPASS_0865 | **B2S4A3** | Outer membrane protein | | No match found | No match found | TPR-like | No match found |
|  | HP TPASS_0867 | **B2S4A4** | PHD-finger domain-containing protein | | flagellar filament 34.5 kDa core protein | No match found | DNA polymerase beta | No match found |
|  | HP TPASS_0869 | **B2S4A6** | cAMP-binding protein | | flagellar filament 31 kDa core protein | No match found | Metalloproteases ("zincins"), | No match found |
|  | HP TPASS_0871 | **B2S4A8** | No significant match found | | No match found | No match found | Porin | No match found |
|  | HP TPASS_0873 | **B2S4B0** | Ubiquitin carboxyl-terminal hydrolase | | flagellar hook-associated protein | tRNA nucleotidyltransferase | No match found | No match found |
|  | HP TPASS_0874 | **B2S4B1** | ABC transporter | | flagellar hook-associated protein | No match found | Nucleic acid-binding protein | No match found |
|  | HP TPASS_0875 | **B2S4B2** | ATP-binding protein | | alanine racemase | P-loop containing nucleotide triphosphate hydrolases | ATP-binding protein | RNA threonylcarbamoyl adenosine modification protein TsaE |
|  | HP TPASS_0876 | **B2S4B3** | glycoprotease family protein | | DNA-binding/iron metalloprotein/AP endonuclease | Glycoprotease | Glycoprotease | Glycoprotease |
|  | HP TPASS_0877 | **B2S4B4** | phosphohydrolase | | sensory transduction histidine kinase | HD domain  (Phosphohydrolase) | Metal dependent phosphohydrolases with conserved 'HD' motif | Metal dependent phosphohydrolases with conserved 'HD' motif |
|  | HP TPASS_0878 | **B2S4B5** | lysosomal alpha-mannosidase-like | | ABC transporter | No match found | Nudix | No match found |
|  | HP TPASS_0879 | **B2S4B6** | ABC transporter | | ABC transporter | ABC transport system | ABC transport system | ABC-type uncharacterised transport system |
|  | HP TPASS_0882 | **B2S4B9** | PBS lyase HEAT-like repeat protein | | translation elongation factor P | HEAT repeats | ARM repeat | Armadillo-type fold |
|  | HP TPASS_0883 | **B2S4C0** | permease, YjgP/YjgQ family | | integral membrane protein | Permease YjgP/YjgQ | Permease YjgP/YjgQ | Permease YjgP/YjgQ |
|  | HP TPASS_0884 | **B2S4C1** | permease, YjgP/YjgQ family | | integral membrane protein | permease YjgP/YjgQ family | Permease YjgP/YjgQ | Permease YjgP/YjgQ |
|  | HP TPASS_0893 | **B2S4D0** | Ribosome maturation factor | | maturation of 30S ribosomal subunits | YhbC-like, N-terminal domain | Ribosome maturation factor rimP | Ribosome maturation factor RimP |
|  | HP TPASS_0894 | **B2S4D1** | NYN domain, limkain-b1-type | | ATP-dependent nuclease, subunit A | NYN domain, limkain-b1-type | Class II aaRS and biotin synthetases | NYN domain, limkain-b1-type |
|  | HP TPASS_0895 | **B2S4D2** | CrcB family protein | | ATP-dependent nuclease, subunit A | Domain of unknown function DUF302 | P-loop containing nucleoside triphosphate hydrolase | Domain of unknown function DUF302 |
|  | HP TPASS_0896 | **B2S4D3** | ATP synthase CF1 alpha subunit | | No match found | No match found | alpha/beta-Hydrolase | No match found |
|  | HP TPASS_0899 | **B2S4D6** | PD-(D/E)XK nuclease family protein | | ATP-dependent nuclease, subunit A | PD-(D/E)XK nuclease superfamily | ARM repeat | PD-(D/E)XK nuclease |
|  | HP TPASS_0900 | **B2S4D7** | PD-(D/E)XK nuclease family protein | | ATP-dependent nuclease, subunit A | PD-(D/E)XK nuclease superfamily | P-loop containing nucleoside triphosphate hydrolase | PD-(D/E)XK nuclease superfamily |
|  | HP TPASS_0901 | **B2S4D8** | MATE family multi antimicrobial extrusion protein | | ATP-dependent nuclease, subunit A | MatE | Mate efflux family protein | Multi antimicrobial extrusion protein |
|  | HP TPASS_0904 | **B2S4E1** | sialic acid synthase | | ribosomal protein S16 | No match found | ARM repeat | No match found |
|  | HP TPASS_0906 | **B2S4E3** | KH domain containing protein | | ribosomal protein S16 | KH domain | FAH | K homology domain, prokaryotic type |
|  | HP TPASS_0907 | **B2S4E4** | 16S rRNA processing protein RimM | | Essential for efficient processing of 16S rRNA | RimM N-terminal domain  (Ribosome maturation factor) | Ribosome maturation factor rimM | Ribosome maturation factor RimM |
|  | HP TPASS_0910 | **B2S4E7** | glycosyltransferase-like protein | | tRNA (guanine-N(1)-)-methyltransferase | No match found | No match found | No match found |
|  | HP TPASS_0911 | **B2S4E8** | FlhB domain containing protein | | No match found | FlhB HrpN YscU SpaS Family | FlhB HrpN YscU SpaS Family | Type III secretion system substrate exporter FlhB-like |
|  | HP TPASS_0912 | **B2S4E9** | HD-GYP domain-containing protein | | No match found | HD domain | Metal dependent phosphohydrolases with conserved 'HD' motif | Metal dependent phosphohydrolases with conserved 'HD' motif |
|  | HP TPASS_0913 | **B2S4F0** | endonuclease | | No match found | Restriction endonuclease like | Restriction endonuclease-like | Restriction endonuclease type II-like |
|  | HP TPASS_0914 | **B2S4F1** | Fe-S oxidoreductase | | Mg2+ transport protein | No match found | Anticodon-binding domain of a subclass of class I aminoacyl-tRNA synthetase | No match found |
|  | HP TPASS_0915 | **B2S4F2** | Tetratricopeptide repeat  containing protein | | Mg2+ transport protein | Tetratricopeptide repeat  containing protein | TPR domain-containing protein | Tetratricopeptide repeat-containing domain |
|  | HP TPASS_0916 | **B2S4F3** | iron-only hydrogenase maturation protein | | Mg2+ transport protein | NADH-ubiquinone oxidoreductase-F iron-sulfur binding region | P-loop containing nucleoside triphosphate hydrolase | No match found |
|  | HP TPASS_0918 | **B2S4F5** | phosphatidate cytidylyltransferase | | integral membrane protein | No match found | GTPase activation domain | No match found |
|  | HP TPASS_0920 | **B2S4F7** | TPR domain  containing protein | | NADH oxidase | Tetratricopeptide repeat  containing protein | TPR repeat-containing protein | Tetratricopeptide repeat-containing domain |
|  | HP TPASS_0922 | **B2S4F9** | No significant match found | | No match found | No match found | TGS-like | No match found |
|  | HP TPASS_0923 | **B2S4G0** | PEGA domain-containing protein | | No match found | PEGA domain | PEGA domain | PEGA domain |
|  | HP TPASS_0927 | **B2S4G4** | AraC family transcriptional regulator | | signal peptidase I | No match found | No match found | No match found |
|  | HP TPASS_0928 | **B2S4G5** | PpiC-type peptidyl-prolyl cis-trans isomerase | | No match found | No match found | DNA primase core | No match found |
|  | HP TPASS_0929 | **B2S4G6** | PpiC-type peptidyl-prolyl cis-trans isomerase | | No match found | PPIC-type PPIASE domain | FKBP-like | No match found |
|  | HP TPASS_0930 | **B2S4G7** | No significant match found | | No match found | sialyltransferase cstii | GroES-like | Protein of unknown function DUF115 |
|  | HP TPASS_0931 | **B2S4G8** | Alpha-alpha trehalase | | UDP-N-acetylmuramoylalanyl-D-glutamate--2,6-diaminopimelate ligase | Trehalase | Trehalase | Trehalase |
|  | HP TPASS_0932 | **B2S4G9** | Crp/Fnr family transcription regulator | | No match found | No match found | No match found | No match found |
|  | HP TPASS_0937 | **B2S4H4** | phosphodiesterase | | pyruvate oxidoreductase | Calcineurin-like phosphoesterase | Phosphodiesterase | Calcineurin-like phosphoesterase |
|  | HP TPASS_0938 | **B2S4H5** | NAD dependent epimerase/dehydratase family protein | | pyruvate oxidoreductase | No match found | Nucleoplasmin-like | No match found |
|  | HP TPASS_0940 | **B2S4H7** | No significant match found | | pyruvate oxidoreductase | No match found | No match found | No match found |
|  | HP TPASS_0941 | **B2S4H8** | NADPH-protochlorophyllide oxidoreductase | | No match found | No match found | Pyrrolidone carboxyl peptidase | No match found |
|  | HP TPASS_0942 | **B2S4H9** | FlgN protein | | flagellar protein | FlgN protein | FlgN protein | FlgN-like domain |
|  | HP TPASS_0944 | **B2S4I1** | Tetratricopeptide repeat  containing protein | | flagellar protein | Tetratricopeptide repeat  containing protein | TPR-like | Tetratricopeptide-like helical |
|  | HP TPASS_0950 | **B2S4I7** | glutamate dehydrogenase | | 50S ribosomal protein | No match found | Guanido kinase | No match found |
|  | HP TPASS_0954 | **B2S4J1** | Tetratricopeptide repeat  containing protein | | pheromone shutdown protein | Tetratricopeptide repeat  containing protein | Tetratricopeptide repeat  containing protein | Tetratricopeptide repeat-containing domain |
|  | HP TPASS_0955 | **B2S4J2** | No significant match found | | pheromone shutdown protein | No match found | Cysteine proteinase | No match found |
|  | HP TPASS_0956 | **B2S4J3** | Lipoprotein | | dicarboxylate transporter | No match found | "Helical backbone" metal receptor | No match found |
|  | HP TPASS_0959 | **B2S4J6** | flagellar protein | | flagellar basal body rod protein | Rod binding protein | Rod binding protein |  |
|  | HP TPASS_0962 | **B2S4J9** | efflux ABC transporter, permease protein | | ABC transporter | FtsX-like permease family | FtsX-like permease family | Permease FtsX-like |
|  | HP TPASS_0963 | **B2S4K0** | macrolide export ATP-binding/permease protein | | ABC transporter | FtsX-like permease family | Macrolide export ATP-binding/permease protein | Permease FtsX-like |
|  | HP TPASS_0966 | **B2S4K3** | Outer membrane protein | | possible membrane fusion protein | No match found | Second domain of Mu2 adaptin subunit (ap50) of ap2 adaptor | No match found |
|  | HP TPASS_0967 | **B2S4K4** | Outer membrane protein | | possible membrane fusion protein | Protein of unknown function (DUF3238) | Immunoglobulin-like beta-sandwich | No match found |
|  | HP TPASS_0968 | **B2S4K5** | chemotaxis protein | | possible membrane fusion protein | Cleavage and polyadenylation factor 2 C-terminal | Immunoglobulin-like beta-sandwich | No match found |
|  | HP TPASS_0969 | **B2S4K6** | Outer membrane protein | | ABC transporter | No match found | Outer membrane efflux protein | No match found |
|  | HP TPASS_0970 | **B2S4K7** | No significant match found | | No match found | No match found | Ribonuclease H-like | No match found |
|  | HP TPASS_0972 | **B2S4K9** | iron permease FTR1 domain protein | | membrane antigen, pathogen-specific | Iron permease FTR1 family | Iron permease FTR1 family | Macrolide export ATP-binding/permease protein |
|  | HP TPASS_0974 | **B2S4L1** | Anti-sigma-28 factor, FlgM | | RNA polymerase sigma factor | Valyl tRNA synthetase tRNA binding arm | Molybdenum cofactor-binding domain | No match found |
|  | HP TPASS_0975 | **B2S4L2** | 16S rRNA methyltransferase | | diphosphate--fructose-6-phosphate 1-phosphotransferase | Methyltransferase | Ribosomal RNA small subunit methyltransferase I | rRNA small subunit methyltransferase I |
|  | HP TPASS_0976 | **B2S4L3** | Prolyl oligopeptidase family | | signal peptidase II | No match found | alpha/beta-Hydrolase | No match found |
|  | HP TPASS_0977 | **B2S4L4** | YbgI/family dinuclear metal center protein | | signal peptidase II | NIF3 (NGG1p interacting factor 3) | NIF3 (NGG1p interacting factor 3) | dinuclear metal center protein |
|  | HP TPASS_0979 | **B2S4L6** | TatD family hydrolase | | histidine phosphokinase/phophatase | TatD related DNase | TatD related DNase | TatD related DNase |
|  | HP TPASS_0983 | **B2S4M0** | iron ABC transporter ATP-binding protein | | sensory transduction histidine kinase | No match found | FMN-linked oxidoreductase | No match found |
|  | HP TPASS_0986 | **B2S4M3** | DMT superfamily drug/metabolite transporter | | integral membrane protein | Multidrug resistance efflux transporter EmrE | EamA-like transporter family | Drug/metabolite transporter |
|  | HP TPASS_0987 | **B2S4M4** | No significant match found | | integral membrane protein | No match found | No match found | No match found |
|  | HP TPASS_0988 | **B2S4M5** | MarC family multiple antibiotic resistance transporter | | integral membrane protein | integral membrane protein | integral membrane protein | Multiple antibiotic resistance (MarC)-related |
|  | HP TPASS_0990 | **B2S4M7** | Tetratricopeptide repeat  containing protein | | rubredoxin | Tetratricopeptide repeat  containing protein | Tetratricopeptide repeat  containing protein | Tetratricopeptide repeat  containing protein |
|  | HP TPASS_0992 | **B2S4M9** | L-Ala-D/L-Glu epimerase | | rubredoxin | No match found | Galactose-binding domain | No match found |
|  | HP TPASS_0994 | **B2S4N1** | magnesium (Mg2+)-dependent deoxyribonuclease | | flagellar hook-basal body protein | TatD related DNase | TatD family hydrolase | Deoxyribonuclease, TatD-related |
|  | HP TPASS_0996 | **B2S4N3** | cyclic nucleotide-binding domain protein | | protease IV | P22_AR N-terminal domain | ARM repeat | No match found |
|  | HP TPASS_1000 | **B2S4N7** | No significant match found | | cell division protein | L,D-transpeptidase catalytic domain | Lumazine synthase | No match found |
|  | HP TPASS_1001 | **B2S4N8** | trypsin-like serine protease | | No match found | Protein of unknown function (DUF4035) | FAD/NAD(P)-binding domain | No match found |
|  | HP TPASS_1002 | **B2S4N9** | Protein of unknown function DUF2259, secreted | | No match found | secreted protein | Galactose mutarotase-like | Protein of unknown function DUF2259, secreted |
|  | HP TPASS_1003 | **B2S4P0** | Polyketide Synthase III | | No match found | No match found | Six-hairpin glycosidase | No match found |
|  | HP TPASS_1014 | **B2S4Q1** | tetratricopeptide repeat protein | | transcription antitermination protein | Tetratricopeptide repeat  containing protein | TPR-like | No match found |
|  | HP TPASS_1018 | **B2S4Q5** | 2',3'-cyclic-nucleotide 2'-phosphodiesterase | | Catalyzes the hydrolysis of both 2',3'-cyclic AMP and 2',3'-cyclic GMP into 3'-AMP and 3'-GMP, respectively, at the 3'- terminal of RNA | K homology RNA-binding domain | 2',3'-cyclic-nucleotide 2'-phosphodiesterase | K homology RNA-binding domain |
|  | HP TPASS_1029 | **B2S4R6** | RNA binding protein | | RNA-binding domain-containing protein | RNA binding domain | RNA binding domain | RNA binding domain |
|  | HP TPASS_1030 | **B2S4R7** | eukaryotic translation initiation factor 4 gamma 1-like | | No match found | No match found | No match found | No match found |
|  | HP TPASS_1032 | **B2S4R9** | Membrane protein | | DUF1312 domain containing protein | Protein of unknown function (DUF1312) | Thioesterase/thiol ester dehydrase-isomerase | Protein of unknown function (DUF1312 |
|  | HP TPASS_1033 | **B2S4S0** | patatin family phospholipase | | integral membrane protein | Patatin-like phospholipase | Patatin-like phospholipase | Patatin-like phospholipase |
|  | HP TPASS_1034 | **B2S4S1** | CaCA family calcium (Ca2+):cation antiporter | | sodium/proton-dependent alanine transporter | Sodium/calcium exchanger protein | Sodium/calcium exchanger protein | Sodium/potassium/calcium exchanger |
