# Supplementary material for: Identification of Functional Candidates amongst Hypothetical Proteins of Treponema pallidum ssp. pallidum
Source: PLoS One. 2015 Apr 20;10(4):e0124177. doi: 10.1371/journal.pone.0124177 (PMC4403809; doi:10.1371/journal.pone.0124177)
Supplement: S4 Table — (DOC) [file pone.0124177.s004.doc]

| **Table S4: List of predicted results of CATH, SUPERFAMILY, Panther, CDART, Pfam, SYSTERS and ProtoNet for 444 HPs from *T. pallidum ssp. pallidum*** | | | | | | | | | | |
| --- | --- | --- | --- | --- | --- | --- | --- | --- | --- | --- |
| **S.No** | **Protein name** | **Uniprot ID** | **CATH** | **SUPER**  **FAMILY** | **PANTHER** | | **CDART** | **Pfam**  **(family/Domain)** | **SYSTERS**  **(Cluster** | **ProtoNet**  **Cluster**  **(cluster name)** |
|  | HP TPASS_0004 | **B2S1V4** | No hit found | No hit found | No hit found | | Peptidase C19 superfamily | IL10 fam ily  (Interleukin 10) | Cluster 113940  (Glycosyltransferase) | Cluster 3918086  Cluster Name: Protein of unknown function DUF721  (Binding protein) |
|  | HP TPASS_0008 | **B2S1V7** | No hit found | No hit found | No hit found | | No hit found | No hit found | Cluster 59427 | Cluster 3642808  Cluster Name: Treponema pallidum  (Binding protein ) |
|  | HP TPASS_0010 | **B2S1V8** | No hit found | No hit found | No hit found | | No hit found | No hit found | Cluster 18356 | No hit found |
|  | HP TPASS_0012 | **B2S1W0** | No hit found | No hit found | No hit found | | No hit found | No hit found | Cluster 18357 | Cluster 462813  Name: Treponema pallidum |
|  | HP TPASS_0013 | **B2S1W1** | No hit found | No hit found | No hit found | | Ribosomal protein P1_P2_ L12p superfamily | DUF3798 family | Cluster 150537  Ribosomal protein S1 | Cluster 4107493 |
|  | HP TPASS_0014 | **B2S1W2** | No hit found | No hit found | No hit found | | Ribosomal protein P1_P2_ L12p superfamily | Sushi_2 family  (Beta-2-glycoprotein-1 fifth domain) | Cluster 142471  Flagellar biosynthesis protein flhF | Cluster 3642808  Cluster Name: Treponema pallidum  (Binding protein ) |
|  | HP TPASS_0017 | **B2S1W5** | Bardet-Biedl syndrome 4 protein -like domain | TPR like | Transcription initiation factor iiic (tfiiic), polypeptide 3 | | TPR superfamily | TPR family | Cluster 136427  TPR-domain containing protein | Cluster 3951334  Cluster Name: Tetratricopeptide region |
|  | HP TPASS_0021 | **B2S1W9** | No hit found | LigA subunit of an aromatic-ring-opening dioxygenase LigAB | No hit found | | No hit found | No hit found | Cluster 141786  oxidoreductase | Cluster 588993  Cluster Name: Treponema pallidum |
|  | HP TPASS_0022 | **B2S1X0** | No hit found | No hit found | No hit found | | Helicase_C_3 superfamily | Helicase_C_3 family | Cluster 97666  thiol:disulfide interchange protein | Cluster 3082548  Cluster Name: Treponema |
|  | HP TPASS_0024 | **B2S1X2** | NAD(P)-binding Rossmann-like Domain | NAD(P)-binding Rossmann-fold domains | Potassium/proton antiporter | | TrkA_N superfamily | TrkA_N family | Cluster 141753  Trk transporter NAD+ binding protein-K+ transport | Cluster 4153677  Cluster Name: Regulator of K+ conductance, N-terminal |
|  | HP TPASS_0025 | **B2S1X3** | Protease -like domain | LuxS/MPP-like metallohydrolase | Metalloprotease | | Peptidase_M16 superfamily | Peptidase_M16 family | Cluster 140382  Zinc metalloprotease | Cluster 4154048  Cluster Name: Cytochrome Bc1 Complex; Chain A, domain 1  (metal binding) |
|  | HP TPASS_0031 | **B2S1X9** | No hit found | No hit found | No hit found | | No hit found | No hit found | Cluster 89859  Multidrug resistance protein | Cluster 421566  Cluster Name: Treponema pallidum |
|  | HP TPASS_0033 | **B2S1Y1** | No hit found | VPS9 domain | No hit found | | Sulfatase superfamily | DUF1361 family | Cluster 101257  Sensory transduction histidine kinase | Cluster 4136071  Cluster Name: Treponema pallidum |
|  | HP TPASS_0039 | **B2S1Y7** | No hit found | No hit found | No hit found | | No hit found | No hit found | Cluster 156602  Transcriptional regulator | Cluster 752124  Cluster Name: Treponema pallidum |
|  | HP TPASS_0041 | **B2S1Y9** | No hit found | No hit found | No hit found | | No hit found | No hit found | Cluster 18400 | Cluster 588996  Cluster Name: Treponema pallidum |
|  | HP TPASS_0042 | **B2S1Z0** | Invasion-associated protein p60 -like domain | LysM domain | No hit found | | LysM superfamily | LysM family | Cluster 147703  LysM domain protein | Cluster 3848320  Cluster Name: Treponema  Peptidoglycan-binding Lysin |
|  | HP TPASS_0046 | **B2S1Z4** | No hit found | No hit found | Ribosomal large subunit pseudouridine synthase b | | PSP1 superfamily | PSP1 family | Cluster 144896  PSP1 C-terminal conserved region | Cluster 4025251  Cluster Name: PSP1 C-terminal conserved region |
|  | HP TPASS_0047 | **B2S1Z5** | No hit found | TM1646-like | No hit found | | Alpha_adapticC2 superfamily | Orn_DAP_Arg_deC family  (Pyridoxal-dependent decarboxylase, C-terminal sheet domain) | Cluster 130554 | Cluster 3864506  Cluster Name: Protein of unknown function DUF327 |
|  | HP TPASS_0048 | **B2S1Z6** | No hit found | Trimeric LpxA-like enzymes | No hit found | | ABC_ATPase superfamily | Bactofilin  (Polymer-forming cytoskeletal) | Cluster 151496  (Prokaryotic membrane lipoprotein lipid attachment site) | Cluster 4028173  Cluster Name: Protein of unknown function DUF583 |
|  | HP TPASS_0049 | **B2S1Z7** | Lipoprotein -like domain | Peptidoglycan hydrolase LytM | Peptidase-related | | Peptidase_M23 | Peptidase_M23 family | Cluster 138456  (Peptidase, M23/M37 family protein ) | Cluster 4141397  Cluster Name: Peptidase M23B |
|  | HP TPASS_0050 | **B2S1Z8** | Phosphoribosyltransferase -like domain | Phosphoribosyltransferases (PRTases) | No hit found | | PRTases_typeI superfamily  (Phosphoribosyl transferase) | Pribosyltran family  (Phosphoribosyl transferase domain) | Cluster 129585  (phosphoribosyltransferase) | Cluster 3975134  Cluster Name: Phosphoribosyltransferase  (Transferase activity) |
|  | HP TPASS_0054 | **B2S202** | 23S rRNA (guanosine-2'-O-)-methyltransferase RlmB -like domain | RNA 2'-O ribose methyltransferase substrate binding domain | Rna methyltransferase | | SpoU_sub_bind superfamily  (RNA 2'-O ribose methyltransferase substrate binding) | SpoU_sub_bind family  (RNA 2'-O ribose methyltransferase substrate binding) | Cluster 143099  (tRNA/RRNA methyltransferase protein) | Cluster 3751893  Cluster Name: TRNA/rRNA methyltransferase  (RNA 2-O ribose methyltransferase) |
|  | HP TPASS_0055 | **B2S203** | No hit found | No hit found | No hit found | | OAD_gamma superfamily  (Oxaloacetate decarboxylase) | OAD_gamma family  (Oxaloacetate decarboxylase, gamma chain) | Cluster 155356  (Oxaloacetate decarboxylase gamma chain (EC 4.1.1.3)) | Cluster 373529  Cluster Name: Oxaloacetate decarboxylase activity |
|  | HP TPASS_0059 | **B2S207** | No hit found | No hit found | No hit found | | No hit found | No hit found | Cluster 130126  Acyloxyacyl hydrolase | Cluster 4313448 |
|  | HP TPASS_0064 | **B2S212** | No hit found | alpha-ketoacid dehydrogenase kinase, N-terminal domain | No hit found | | No hit found | BCDHK_Adom3 family  (Mitochondrial branched-chain alpha-ketoacid dehydrogenase kinase) | Cluster 96416  (ARM repeat fold) | Cluster 3642696  (Binding protein ) |
|  | HP TPASS_0065 | **B2S213** | Ribosomal RNA small subunit methyltransferase D -like domain | S-adenosyl-L-methionine-dependent methyltransferases | Methyltransferase | | AdoMet_MTases  Superfamily  (S-adenosylmethionine-dependent methyltransferases) | FlhE family  (Flagellar protein FlhE) | Cluster 142947  (Methyltransferase (EC 2.1.1.-)) | Cluster 4136223  (Methyltransferase) |
|  | HP TPASS_0066 | **B2S214** | No hit found | TPR-like | No hit found | |  | TPR_2 family  (Tetratricopeptide repeat) | Cluster 119996  (TPR containing protein) | Cluster 4078427  (Binding protein ) |
|  | HP TPASS_0067 | **B2S215** | Slr2048 protein -like domain | TPR-like | Tetratricopeptide repeat protein, tpr | | TPR superfamily | TPR family | Cluster 126653  (TPR-repeat-containing protein) | Cluster 4053800  Cluster Name: TPR repeat |
|  | HP TPASS_0068 | **B2S216** | No hit found | Radical SAM enzymes | Radical sam domain-containing protein | | Radical_SAM  superfamily | Radical_SAM | Cluster 114513  (Fe-S-cluster redox enzyme) | Cluster 4146805  (RNA methyltransferase activity) |
|  | HP TPASS_0069 | **B2S217** | No hit found | No hit found | No hit found | | No hit found | No hit found | Cluster 62246  (integral membrane protein) | Cluster 3603570  Cluster Name: Treponema |
|  | HP TPASS_0070 | **B2S218** | No hit found | No hit found | No hit found | | No hit found | No hit found | Cluster 18468 | Cluster 3689541  Cluster Name: Treponema |
|  | HP TPASS_0072 | **B2S220** | Regulatory protein spx -like domain | Thioredoxin-like | No hit found | | Antibiotic_NAT superfamily  (Aminoglycoside 3-N-acetyltransferase) | Glutaredoxin family | Cluster 128760  (Glutaredoxin) | Cluster 4071603  Cluster Name: Uncharacterised conserved protein UCP037291, glutaredoxin-related (Disulfide oxidoreductase activity) |
|  | HP TPASS_0073 | **B2S221** | No hit found | HD-domain/PDEase-like | No hit found | | HDc superfamily  (Metal dependent phosphohydrolases with conserved 'HD' motif) | HDOD family | Cluster 140364  (hydrolase) | Cluster 4120990  Cluster Name: Metal-dependent hydrolase HDOD |
|  | HP TPASS_0079 | **B2S227** | Xanthine dehydrogenase -like domain | CO dehydrogenase molybdoprotein N-domain-like | Xanthine dehydrogenase | | Ald_Xan_dh_C2 superfamily  (Molybdopterin-binding domain of aldehyde dehydrogenase) | Ald_Xan_dh_C2 family  (Molybdopterin-binding domain of aldehyde dehydrogenase) | Cluster 143899  (Xanthine dehydrogenase) | Cluster 2708312  Cluster Name: CO dehydrogenase molybdoprotein N-domain-like |
|  | HP TPASS_0081 | **B2S229** | UDP-N-acetylenolpyruvoylglucosamine reductase -like domain | FAD-binding/transporter-associated domain-like | Xanthine dehydrogenase | | FAD_binding_4 superfamily | FAD_binding_5  (FAD binding domain in molybdopterin dehydrogenase) | Cluster 143901  (xanthine dehydrogenase yagS, FAD binding subunit (EC 1.1.1.204)) | Cluster 3863893  Cluster Name: Molybdopterin dehydrogenase, FAD-binding |
|  | HP TPASS_0083 | **B2S231** | No hit found | (Trans)glycosidases | No hit found | | DUF4015 superfamily  (glycosyl hydrolase domain) | DUF4015  superfamily  (domain) | Cluster 115787  (Glycosyltransferase) | Cluster 4094597 |
|  | HP TPASS_0084 | **B2S232** | No hit found | Thioredoxin-like | No hit found | | No hit found | No hit found | Cluster 140878  (Glutaredoxin-like protein) | Cluster 281230  Cluster Name: Treponema pallidum |
|  | HP TPASS_0086 | **B2S234** | No hit found | PilZ domain-like | No hit found | | PilZ superfamily | PilZ family | Cluster 122879  (TonB-dependent receptor protein) | Cluster 3866293  Cluster Name: Type IV pilus assembly PilZ |
|  | HP TPASS_0087 | **B2S235** | No hit found | No hit found | No hit found | | Lipoprotein_16 superfamily | DUF192 family  () | Cluster 156200  (Prokaryotic membrane lipoprotein lipid attachment site) | Cluster 4139460  Cluster Name: Protein of unknown function DUF192 |
|  | HP TPASS_0088 | **B2S236** | No hit found | No hit found | No hit found | | No hit found | No hit found | Cluster 127733  (Hydrolase related to 2-haloalkanoic acid dehalogenase) | Cluster 3978650 |
|  | HP TPASS_0093 | **B2S241** | No hit found | No hit found | No hit found | | zf-HC2 family  (zinc-finger) | No hit found | Cluster 18493 | Cluster 614863  Cluster Name: Treponema pallidum |
|  | HP TPASS_0095 | **B2S243** | Ogt protein -like domain | TPR like | Tetratricopeptide repeat protein, tpr | | TPR superfamily | TPR family | Cluster 126653 (TPR-containing protein) | Cluster 2574289  Cluster Name: Treponema  (Tetratricopeptide region) |
|  | HP TPASS_0110 | **B2S258** | No hit found | No hit found | No hit found | | No hit found | No hit found | Cluster 18509 | Cluster 2667617  Cluster Name: Treponema |
|  | HP TPASS_0118 | **B2S266** | No hit found | Tropomyosin | No hit found | | No hit found | No hit found | Cluster 139398  (Chromosome segregation protein) | Cluster 3670998  Cluster Name: Treponema |
|  | HP TPASS_0121 | **B2S269** | No hit found | Radical SAM enzymes | L-lysine 2,3-aminomutase | | LAM_C  (Lysine-2,3-aminomutase) | LAM_C  (Lysine-2,3-aminomutase) | Cluster 133235  (L-lysine 2,3-aminomutase) | Cluster 4031921  (Radical SAM) |
|  | HP TPASS_0123 | **B2S271** | Response regulator aspartate phosphatase A -like domain | TPR-like | No hit found | | No hit found | TPR family | Cluster 141541  (TPR containing protein) | Cluster 398069  Cluster Name: Treponema pallidum  (TPR) |
|  | HP TPASS_0126 | **B2S274** | No hit found | OMPA-like | No hit found | | Csy2_I-F Superfamily  (CRISPR/Cas system-associated RAMP superfamily protein) | OMP_b-br  (Outer membrane protein beta-barrel domain) | Cluster 120021 | Cluster 3822473  Cluster Name: Treponema |
|  | HP TPASS_0127 | **B2S275** | No hit found | No hit found | No hit found | | DUF2715 superfamily | DUF2715 family | Cluster 156020 | Cluster 4140084  (Cluster Name: Treponema pallidum) |
|  | HP TPASS_0128 | **B2S276** | No hit found | No hit found | No hit found | | No hit found | No hit found | Cluster 18531 | Cluster 705795 |
|  | HP TPASS_0129 | **B2S277** | No hit found | No hit found | No hit found | | No hit found | No hit found | Cluster 18532 | Cluster 3785509 |
|  | HP TPASS_0130 | **B2S278** | No hit found | No hit found | No hit found | | No hit found | No hit found | Cluster 124340  (Flagellar motor protein MotA) | Cluster 4313299 |
|  | HP TPASS_0132 | **B2S280** | No hit found | No hit found | No hit found | | No hit found | No hit found | No hit found | No hit found |
|  | HP TPASS_0133 | **B2S281** | No hit found | No hit found | No hit found | | No hit found | No hit found | Cluster 133274  (Prokaryotic membrane lipoprotein lipid attachment site) | Cluster 4078211  Cluster Name: Treponema pallidum |
|  | HP TPASS_0134 | **B2S282** | No hit found | beta-lactamase inhibitor protein-II, BLIP-II | No hit found | | No hit found | Kelch_4  (Galactose oxidase, central domain) | Cluster 133274  (Prokaryotic membrane lipoprotein lipid attachment site) | Cluster 4078211  Cluster Name: Treponema pallidum |
|  | HP TPASS_0135 | **B2S283** | No hit found | No hit found | No hit found | | No hit found | No hit found | Cluster 129295  (Transcription factor) | Cluster 3134681  Cluster Name: Treponema |
|  | HP TPASS_0136 | **B2S284** | No hit found | No hit found | No hit found | | No hit found | No hit found | Cluster 133274  (Prokaryotic membrane lipoprotein lipid attachment site) | Cluster 4078211  Cluster Name: Treponema pallidum |
|  | HP TPASS_0137 | **B2S285** | No hit found | No hit found | No hit found | | No hit found | No hit found | Cluster 18546 | Cluster 801538  Cluster Name: Treponema pallidum |
|  | HP TPASS_0138 | **B2S286** | No hit found | No hit found | No hit found | | No hit found | 7tm_7 family  (7tm Chemosensory receptor) | Cluster 111571  (ABC transporter (ATP-binding protein)) | Cluster 3332989  Cluster Name: Treponema |
|  | HP TPASS_0139 | **B2S287** | Glutathione-regulated potassium-efflux system -like domain | NAD(P)-binding Rossmann-fold domain | Potassium/proton antiporter-related | | TrkA_N superfamily | TrkA_N | Cluster 141753  (K+ transport systems, NAD-binding component) | Cluster 4153677  Cluster Name: Regulator of K+ conductance, N-terminal |
|  | HP TPASS_0148 | **B2S295** | No hit found | No hit found | No hit found | | No hit found | No hit found | Cluster 146390  (Aldo/keto reductase) | Cluster 443612  Cluster Name: Treponema pallidum |
|  | HP TPASS_0149 | **B2S296** | No hit found | No hit found | No hit found | | No hit found | YrvL  (Regulatory protein) | Cluster 153833  (Phosphatidylglycerol/phosphatidylinositol transfer protein) | Cluster 3665744  (Cluster Name: Treponema) |
|  | HP TPASS_0150 | **B2S297** | No hit found | No hit found | No hit found | | No hit found | No hit found | Cluster 144325  (Hydrogenase expression/formation protein) | Cluster 4543070 |
|  | HP TPASS_0151 | **B2S298** | No hit found | No hit found | No hit found | | NQR2_RnfD_RnfE Superfamily | NQR2_RnfD_RnfE | Cluster 155350  (Na(+)-translocating NADH-quinone reductase subunit B (EC 1.6.5.-)) | Cluster 3462035  Cluster Name: NADH-quinone reductase NQR2/RnfD |
|  | HP TPASS_0153 | **B2S2A0** | No hit found | Acid phosphatase/Vanadium-dependent haloperoxidase | Acid phosphatase/vanadium-dependent haloperoxidase-related protein | | Cu_amine_oxidN1 Superfamily  (Copper amine oxidases) | PAP2 family | Cluster 146209  (Acid phosphatase/vanadium-dependent haloperoxidase) | Cluster 3984991  Cluster Name: Acid phosphatase/vanadium-dependent haloperoxidase related |
|  | HP TPASS_0154 | **B2S2A1** | No hit found | Pseudouridine synthase | | RNA PSEUDOURIDYLATE SYNTHASE FAMILY PROTEIN | PseudoU_synth  superfamily  (RNA pseudouridylate synthase) | PseudoU_synth_2  Family  (RNA pseudouridylate synthase) | Cluster 137205  (Ribosomal large subunit pseudouridine synthase C (EC 4.2.1.70)) | Cluster 4082684  Cluster Name: Pseudouridine synthase, RsuA and RluB/C/D/E/F |
|  | HP TPASS_0156 | **B2S2A3** | 1,4-dihydroxy-2-naphthoyl-CoA hydrolase -like domain | Thioesterase/thiol ester dehydrase-isomerase | Thioesterase-like protein | | hot_dog Superfamily  (4-hydroxybenzoyl-CoA thioesterase like) | 4HBT  (Thioesterase superfamily) | Cluster 149567  (Thioesterase) | Cluster 4118274  Cluster Name: 4-hydroxybenzoyl-CoA thioesterase |
|  | HP TPASS_0157 | **B2S2A4** | 1-acyl-sn-glycerol-3-phosphate acyltransferase -like domain | Glycerol-3-phosphate (1)-acyltransferase | No hit found | | hot_dog Superfamily  (4-hydroxybenzoyl-CoA thioesterase like) | Acyltransferase family | Cluster 116237  (Glycerol-3-phosphate O-acyltransferase,) | Cluster 3320570  Cluster Name: Phospholipid/glycerol acyltransferase |
|  | HP TPASS_0158 | **B2S2A5** | Suppressor of disruption of TFIIS -like domain | HAD-like | Haloacid dehalogenase-like hydrolase | | HAD superfamily  (Haloacid dehalogenase-like hydrolase) | HAD_2 family  (Haloacid dehalogenase-like hydrolase) | Cluster 147698  (Hydrolase) | Cluster 4098414  luster Name: Pyrimidine 5-nucleotidase |
|  | HP TPASS_0159 | **B2S2A6** | No hit found | No hit found | No hit found | | No hit found | No hit found | Cluster 18591 | Cluster 3172258  Cluster Name: Treponema |
|  | HP TPASS_0161 | **B2S2A8** | No hit found | No hit found | No hit found | | No hit found | No hit found | Cluster 18596 | No hit found |
|  | HP TPASS_0169 | **B2S2B6** | No hit found | No hit found | No hit found | | No hit found | No hit found | Cluster 18604 | No hit found |
|  | HP TPASS_0172 | **B2S2B9** | No hit found | Nucleotide cyclase | No hit found | | Nucleotidyl_cyc_III Superfamily | GGDEF domain | Cluster 18623 | Cluster 3734220  (Diguanylate cyclase) |
|  | HP TPASS_0173 | **B2S2C0** | No hit found | No hit found | No hit found | | No hit found | DUF2140 family | Cluster 145798  (Sugar transporter related protein) | Cluster 776221  Cluster Name: Treponema pallidum |
|  | HP TPASS_0174 | **B2S2C1** | No hit found | No hit found | No hit found | | No hit found | Protein of unknown function DUF115 |  | Cluster 4054221  Cluster Name: Protein of unknown function DUF115 |
|  | HP TPASS_0175 | **B2S2C2** | No hit found | No hit found | No hit found | | TPR superfamily | MAF_flag10 family  Protein of unknown function DUF115 | Cluster 151949  (TPR containing protein) | Cluster 4058338  Cluster Name: Protein of unknown function DUF115 |
|  | HP TPASS_0176 | **B2S2C3** | No hit found | No hit found | No hit found | | No hit found | No hit found | Cluster 139982  (endonuclease activity) | Cluster 4253105 |
|  | HP TPASS_0177 | **B2S2C4** | No hit found | No hit found | No hit found | | TPPK_C Superfamily  (Thiamine pyrophosphokinase C terminal) | MAF_flag10 family  Protein of unknown function DUF115 | Cluster 18628 | Cluster 3050869  Cluster Name: Treponema |
|  | HP TPASS_0178 | **B2S2C5** | No hit found | No hit found | No hit found | | LETM1 Superfamily  (inner mitochondrial membrane proteins) | No hit found | Cluster 18630 | Cluster 3636231  Cluster Name: Treponema |
|  | HP TPASS_0179 | **B2S2C6** | No hit found | No hit found | No hit found | | No hit found | No hit found | Cluster 140264  (Methyl-CpG binding) | Cluster 4016076 |
|  | HP TPASS_0180 | **B2S2C7** | No hit found | No hit found | No hit found | | No hit found | No hit found | Cluster 18635 | Cluster 398177  Cluster Name: Treponema pallidum |
|  | HP TPASS_0181 | **B2S2C8** | No hit found | Methylated DNA-protein cysteine methyltransferase domain | No hit found | | DivIC superfamily  (Septum formation initiator) | DivIC family  (Septum formation initiator) | Cluster 120038  (Septum formation initiator) | Cluster 3607700  Cluster Name: Septum formation initiator |
|  | HP TPASS_0182 | **B2S2C9** | tRNA threonylcarbamoyladenosine biosynthesis -like domain | YrdC/RibB | Sua5 family  (Telomere recombination) | | Sua5_yciO_yrdC superfamily  (Telomere recombination) | Sua5_yciO_yrdC family  (Telomere recombination) | Cluster 90461  (Telomere recombination) | Cluster 3978790  Cluster Name: Sua5/YciO/YrdC/YwlC |
|  | HP TPASS_0183 | **B2S2D0** | No hit found | No hit found | No hit found | | No hit found | DUF3876 family | Cluster 143377  (protease) | Cluster 3249630  Cluster Name: Treponema |
|  | HP TPASS_0214 | **B2S2G2** | No hit found | No hit found | No hit found | | No hit found | Desulfoferrod_N family  (Desulfoferrodoxin, N-terminal domain) | Cluster 18660 | Cluster 4044715 |
|  | HP TPASS_0222 | **B2S2G9** | No hit found | No hit found | No hit found | | No hit found | FadA family  (Adhesion protein) | Cluster 138176  (DNA double-strand break repair rad50 ATPase) | Cluster 4125709 |
|  | HP TPASS_0223 | **B2S2H0** | Aspartate aminotransferase -like domain | PLP-dependent transferases | Aminotransferase related | | AAT_I Superfamily  (Aspartate aminotransferase (AAT) superfamily (fold type I)) | Aminotran_1_2 family  (Aminotransferase class I and II) | Cluster 127509  (Aspartate aminotransferase (EC 2.6.1.1)) | Cluster 3139156  Cluster Name: Aminotransferase, class I and II |
|  | HP TPASS_0224 | **B2S2H1** | No hit found | No hit found | No hit found | | No hit found | No hit found | Cluster 18664 | No hit found |
|  | HP TPASS_0226 | **B2S2H3** | No hit found | No hit found | No hit found | | CbiQ superfamily  (Cobalt transport protein) | CbiQ family  (Cobalt transport protein) | Cluster 136756 | Cluster 856684  Cluster Name: Treponema pallidum |
|  | HP TPASS_0231 | **B2S2H8** | No hit found | Pseudouridine synthase | Rna pseudouridylate synthase family protein | | PseudoU_synthsuperfamily  (pseudouridylate synthase) | PseudoU_synth_2 family  (RNA pseudouridylate synthase) | Cluster 104528  (Pseudouridine synthase) | Cluster 4188164  Cluster Name: Pseudouridine synthase, RsuA and RluB/C/D/E/F |
|  | HP TPASS_0232 | **B2S2H9** | No hit found | No hit found | No hit found | | No hit found | No hit found | Cluster 18670 | No hit found |
|  | HP TPASS_0245 | **B2S2J2** | Midasin -like domain | P-loop containing nucleoside triphosphate hydrolases | Midasin-related | | AAA Superfamily  (ATPases) | AAA_5 family  (AAA domain (dynein-related subfamily)) | Cluster 139803  (AAA ATPase) | Cluster 2673249 |
|  | HP TPASS_0246 | **B2S2J3** | Collagen alpha-3(VI) chain -like domain | [vWA-like](http://supfam.cs.bris.ac.uk/SUPERFAMILY/cgi-bin/scop.cgi?sunid=53300) | Von willebrand factor, type a domain containing | | vWFA superfamily  Von Willebrand factor type A (vWA) | VWA family  (von Willebrand factor type A domain) | Cluster 18679  (von Willebrand factor, type A) | Cluster 3106512  Cluster Name: Von Willebrand factor, type A |
|  | HP TPASS_0248 | **B2S2J5** | No hit found | No hit found | No hit found | | No hit found | Germane  (Sporulation and spore germination) | Cluster 18681 | Cluster 4074834  Cluster Name: Treponema |
|  | HP TPASS_0250a | **B2S2J7** | No hit found | No hit found | No hit found | | No hit found | No hit found | Cluster 18683 | Cluster 662571  Cluster Name: Treponema pallidum |
|  | HP TPASS_0253 | **B2S2K1** | No hit found | No hit found | No hit found | | ABC_ATPase Superfamily  (ATP-binding cassette transporter nucleotide-binding domain) | Bactofilin family  (Polymer-forming cytoskeletal) | Cluster 151496  (Prokaryotic membrane lipoprotein lipid attachment site) | Cluster 4166010 |
|  | HP TPASS_0258 | **B2S2K6** | No hit found | Snake toxin-like | No hit found | | No hit found | No hit found | Cluster 137796  (Polysaccharide deacetylase) | Cluster 3851409 |
|  | HP TPASS_0259 | **B2S2K7** | No hit found | LysM domain | No hit found | | LysM  superfamily | LysM family | Cluster 147703  (LysM domain protein) | Cluster 3692921  Cluster Name: Peptidoglycan-binding lysin domain |
|  | HP TPASS_0260 | **B2S2K8** | No hit found | No hit found | No hit found | | SH3_3 superfamily  (Bacterial SH3 domain) | SH3_3 family  (Bacterial SH3 domain) | Cluster 18689 | Cluster 3764561 |
|  | HP TPASS_0263 | **B2S2L1** | C-reactive protein -like domain | Fibronectin type III | No hit found | | FN3 superfamily  (Fibronectin type 3 domain) | fn3 family  (Fibronectin type III domain) | Cluster 115690  (Fibronectin, type III) | Cluster 3647362  Cluster Name: Fibronectin type III |
|  | HP TPASS_0266 | **B2S2L4** | No hit found | No hit found | No hit found | | No hit found | No hit found | Cluster 18692 | No hit found |
|  | HP TPASS_0267 | **B2S2L5** | No hit found | No hit found | No hit found | | ABC_ATPase Superfamily  (ATP-binding cassette transporter nucleotide-binding domain) | Bactofilin family  (Polymer-forming cytoskeletal) | Cluster 129873  (Glucose 6-phosphate dehydrogenase assembly protein OPCA) | Cluster 4166010 |
|  | HP TPASS_0268 | **B2S2L6** | Bardet-Biedl syndrome 4 protein -like domain | Tetratricopeptide repeat (TPR) | Tetratricopeptide repeat protein, tpr | | TPR superfamily | TPR_16 family | Cluster 136427  (O-linked N-acetylglucosamine transferase) | Cluster 3710181 |
|  | HP TPASS_0269 | **B2S2L7** | (Dimethylallyl)adenosine tRNA -like domain | Radical SAM enzymes | Threonylcarbamoyladenosine trna methylthiotransferase | | Radical_SAM superfamily | Radical_SAM | Cluster 145170  (methyltransferase activity) | Cluster 4160521 |
|  | HP TPASS_0273 | **B2S2M1** | No hit found | No hit found | No hit found | | No hit found | No hit found | Cluster 120382  (Double-stranded beta-helix fold enzyme) | Cluster 507011  Cluster Name: Treponema pallidum |
|  | HP TPASS_0278 | **B2S2M6** | No hit found | No hit found | No hit found | | No hit found | No hit found | Cluster 18707 | Cluster 801950  Cluster Name: Treponema pallidum |
|  | HP TPASS_0280 | **B2S2M8** | No hit found | No hit found | No hit found | | No hit found | No hit found | Cluster 18710 | Cluster 313769  Cluster Name: Treponema pallidum |
|  | HP TPASS_0281 | **B2S2M9** | No hit found | No hit found | No hit found | | No hit found | No hit found | Cluster 18711 | Cluster 4810538  Cluster Name: Hydrophobin 2 |
|  | HP TPASS_0282 | **B2S2N0** | MxaK protein -like domain | TPR-like | Tetratricopeptide repeat protein, tpr | | TPR superfamily | TPR_12 family | Cluster 109313  (O-linked GlcNAc transferase) | Cluster 4111853  Cluster Name: TPR repeat  (Serine Threonine Protein Phosphatase 5, Tetratricopeptide repeat) |
|  | HP TPASS_0284 | **B2S2N2** | No hit found | Nucleotide-diphospho-sugar transferases | No hit found | | No hit found | NTP_transf_3 family  (MobA-like NTP transferase domain) | Cluster 138509  (RNA binding protein) | Cluster 333276  Cluster Name: Treponema pallidum |
|  | HP TPASS_0285 | **B2S2N3** | No hit found | Radical SAM enzymes | No hit found | | SPASM  (Iron-sulfur cluster-binding domain) | SPASM  (Iron-sulfur cluster-binding domain) | Cluster 116793  (Radical SAM) | Cluster 3496484  Cluster Name: Radical SAM |
|  | HP TPASS_0286 | **B2S2N4** | No hit found | No hit found | No hit found | | No hit found | No hit found | Cluster 134987 | Cluster 4150279 |
|  | HP TPASS_0287 | **B2S2N5** | No hit found | Nucleotide-diphospho-sugar transferases | No hit found | | No hit found | No hit found | Cluster 139085  (Heat shock protein Hsp70) | Cluster 3684154 |
|  | HP TPASS_0289 | **B2S2N7** | No hit found | S-adenosyl-L-methionine-dependent methyltransferases | Hexaprenyldihydroxybenzoate methyltransferase | | AdoMet_MTases Superfamily  (S-adenosylmethionine-dependent methyltransferases) | Methyltransf_23 family  (Methyltransferase domain) | Cluster 145982  (S-adenosylmethionine-dependent methyltransferase) | Cluster 4126992 |
|  | HP TPASS_0290 | **B2S2N8** | No hit found | HAD like | Flavin mononucleotide phosphatase ybji-related | | HAD_like Superfamily  (Haloacid dehalogenase-like hydrolase) | Hydrolase_3 family  (haloacid dehalogenase-like hydrolase) | Cluster 139305  (HAD phosphatase superfamily protein) | Cluster 4205687  (Cluster Name: HAD superfamily hydrolase-like, type 3) |
|  | HP TPASS_0291 | **B2S2N9** | Tryptophan synthase -like domain | FMN-linked oxidoreductases | (S)-2-hydroxy-acid oxidase-related | | TIM_phosphate_binding Superfamily  () | FMN_dh family  (FMN-dependent dehydrogenase) | Cluster 139396  (FMN-dependent alpha-hydroxy acid dehydrogenase) | Cluster 4147549  Cluster Name: Alpha-hydroxy acid dehydrogenase, FMN-dependent |
|  | HP TPASS_0293 | **B2S2P1** | No hit found | No hit found | No hit found | | PI-PLCc_GDPD_SF Superfamily  (Catalytic domain of phosphoinositide-specific phospholipase C-like phosphodiesterases superfamily) | No hit found | Cluster 141896  (NosX protein required for nitrous oxide reduction) | Cluster 652052  Cluster Name: Treponema pallidum |
|  | HP TPASS_0296 | **B2S2P4** | Dephospho-CoA kinase -like domain | P-loop containing nucleoside triphosphate hydrolases | Dephospho-coa kinase-related | | NK superfamily  (Nucleoside/nucleotide kinase (NK)) | CoaE family  (Dephospho-CoA kinase) | Cluster 90686  (Dephospho-CoA kinase (EC 2.7.1.24) (Dephosphocoenzyme A kinase)) | Cluster 3278533  Cluster Name: Dephospho-CoA kinase activity |
|  | HP TPASS_0297 | **B2S2P5** | DedD protein -like domain | Sporulation related repeat | No hit found | | SPOR superfamily  (Sporulation related domain) | SPOR family  (Sporulation related domain) | Cluster 78967  (Sporulation related) | Cluster 4058588  Cluster Name: Sporulation related repeat (SPOR, Pfam 05036) |
|  | HP TPASS_0299 | **B2S2P7** | No hit found | No hit found | No hit found | | No hit found | No hit found | Cluster 18721 | Cluster 706304 |
|  | HP TPASS_0301 | **B2S2P9** | No hit found | No hit found | No hit found | | TM_PBP1_branched-chain-AA_like Superfamily  (ATP-Binding Cassette (ABC) transporters) | BPD_transp_2 family  (Branched-chain amino acid transport system / permease component) | Cluster 143829  (ABC transporter, membrane spanning protein) | Cluster 4151015  Cluster Name: Bacterial inner-membrane translocator |
|  | HP TPASS_0302 | **B2S2Q0** | No hit found | No hit found | Abc transporter permease protein | | TM_PBP1_branched-chain-AA_like Superfamily  (ATP-Binding Cassette (ABC) transporters) | BPD_transp_2 family  (Branched-chain amino acid transport system / permease component) | Cluster 143830  (ABC transporter, membrane spanning protein) | Cluster 3328548  Cluster Name: Bacterial inner-membrane translocator |
|  | HP TPASS_0304 | **B2S2Q2** | Tricorn protease -like domain | DPP6 N-terminal domain-like | No hit found | | GluZincin  (Peptidase Gluzincin family (thermolysin-like proteinases, TLPs)) | Peptidase_MA_2  (Peptidase MA superfamily) | Cluster 116340  (Peptidase M, neutral zinc metallopeptidases, zinc-binding site) | Cluster 3809179 |
|  | HP TPASS_0307 | **B2S2Q5** | No hit found | No hit found | No hit found | | PASTA  (PASTA domain) | PASTA  (PASTA domain) | Cluster 99213  (serine/threonine protein kinase) | Cluster 4095676  Cluster Name: PASTA |
|  | HP TPASS_0310 | **B2S2Q8** | Single-stranded DNA-binding protein -like domain | Single strand DNA-binding domain, SSB | Single-stranded dna-binding protein, mitochondrial | | RPA_2b-aaRSs_OBF_like Superfamily  (Replication protein A, class 2b aminoacyl-tRNA synthetases, and related proteins with oligonucleotide/oligosaccharide (OB) fold) | SSB family  (Single-strand binding protein family) | Cluster 88578  (Single-strand binding protein) | Cluster 4129282  Cluster Name: Single-strand DNA-binding |
|  | HP TPASS_0311 | **B2S2Q9** | No hit found | No hit found | No hit found | | No hit found | Optomotor-blind family  (Optomotor-blind protein) | Cluster 136421 | Cluster 686107  Cluster Name: Treponema pallidum |
|  | HP TPASS_0312 | **B2S2R0** | No hit found | No hit found | No hit found | | E1-E2 ATPase superfamily | DUF368 family | Cluster 128631 | Cluster 4099633 |
|  | HP TPASS_0314 | **B2S2R2** | No hit found | No hit found | No hit found | | DUF2715  (Protein of unknown function) | DUF2715  (Protein of unknown function) | Cluster 156019 | Cluster 4140084  Cluster Name: Treponema pallidum |
|  | HP TPASS_0315 | **B2S2R3** | No hit found | No hit found | No hit found | | Protein of unknown function DUF2715 | Protein of unknown function DUF2715 | Cluster 157940  (integral membrane protein) | Cluster 4140084  Cluster Name: Treponema pallidum |
|  | HP TPASS_0318 | **B2S2R5** | No hit found | No hit found | No hit found | | No hit found | No hit found | Cluster 18731 | Cluster 776409  Cluster Name: Treponema pallidum |
|  | HP TPASS_0320 | **B2S2R7** | No hit found | No hit found | No hit found | | No hit found | No hit found | Cluster 18735 | Cluster 4765645  (Adhesion) |
|  | HP TPASS_0324 | **B2S2S1** | No hit found | No hit found | No hit found | | No hit found | No hit found | Cluster 146586  (Fe-S oxidoreductase) | Cluster 838640  Cluster Name: Treponema pallidum |
|  | HP TPASS_0325 | **B2S2S2** | No hit found | No hit found | No hit found | | AsmA_2 superfamily | DUF490  Family of unknown function | Cluster 60870  (Outer membrane component of multidrug efflux pump) | Cluster 3837251  Cluster Name: Protein of unknown function DUF490 |
|  | HP TPASS_0332 | **B2S2S9** | No hit found | No hit found | No hit found | | No hit found | No hit found | Cluster 18749 | No hit found |
|  | HP TPASS_0333 | **B2S2T0** | Outer-membrane lipoprotein carrier protein -like domain | Outer-membrane lipoproteins carrier protein LolA | No hit found | | LolA superfamily  (Outer membrane lipoprotein carrier protein) | LolA family  (Outer membrane lipoprotein carrier protein) | Cluster 152443  (Outer membrane lipoprotein-sorting protein) | Cluster 3793189  Cluster Name: Outer membrane lipoprotein carrier protein LolA |
|  | HP TPASS_0334 | **B2S2T1** | DNA-binding protein -like domain | lambda repressor-like DNA-binding domains | No hit found | | HTH_XRE superfamily  (DNA binding proteins) | HTH_25 family  (Helix-turn-helix domain) | Cluster 150263  (Helix turn helix motif: DNA binding) | Cluster 4180464  Cluster Name: Helix-turn-helix type 3 |
|  | HP TPASS_0335 | **B2S2T2** | No hit found | No hit found | No hit found | | Abi superfamily  (CAAX protease self-immunity) | Abi family  (CAAX protease self-immunity) | Cluster 18754  (Abortive infection protein) | Cluster 3923119  Cluster Name: Treponema |
|  | HP TPASS_0338 | **B2S2T5** | No hit found | Photosystem II reaction center protein I, PsbI | No hit found | | No hit found | Mid2 family  (Mid2 like cell wall stress sensor) | Cluster 126161  (oxidoreductase protein (EC 1.-.-.-)) | Cluster 4128581 |
|  | HP TPASS_0339 | **B2S2T6** | No hit found | Pseudouridine synthase | Ribosomal large subunit pseudouridine synthase d | | PseudoU_synth  superfamily  ( pseudouridylate synthase) | PseudoU_synth_2  Family  (RNA pseudouridylate synthase) | Cluster 136780  (Ribosomal large subunit pseudouridine synthase D (EC 4.2.1.70)) | Cluster 4188164  Cluster Name: Pseudouridine synthase, RsuA and RluB/C/D/E/F |
|  | HP TPASS_0346 | **B2S2U3** | No hit found | No hit found | No hit found | | Protein of unknown function DUF2715 | Protein of unknown function DUF2715 | Cluster 156020 | Cluster 4140084  Cluster Name: Treponema pallidum |
|  | HP TPASS_0347 | **B2S2U4** | No hit found | No hit found | No hit found | | Protein of unknown function DUF2715 | Protein of unknown function DUF2715 | Cluster 156020 | Cluster 4140084  Cluster Name: Treponema pallidum |
|  | HP TPASS_0348 | **B2S2U5** | No hit found | No hit found | No hit found | | Hpre_diP_synt_I superfamily  (Heptaprenyl diphosphate synthase component I) | Hpre_diP_synt_I family  (Heptaprenyl diphosphate synthase component I) | Cluster 115457  (ABC-type transporter, permease components) | Cluster 4140453  Cluster Name: Heptaprenyl diphosphate synthase component I |
|  | HP TPASS_0352 | **B2S2U9** | No hit found | No hit found | No hit found | | No hit found | PC4 family  (Transcriptional Coactivator p15) | Cluster 33756  (NADH dehydrogenase subunit 6) | Cluster 4158392 |
|  | HP TPASS_0355 | **B2S2V2** | No hit found | No hit found | No hit found | | No hit found | No hit found | Cluster 128583  (Nuclear receptor coactivator) | Cluster 4140084  Cluster Name: Treponema pallidum |
|  | HP TPASS_0358 | **B2S2V5** | Alpha-mannosidase -like domain | Glycoside hydrolase/deacetylase | No hit found | | GH38-57_N_LamB_YdjC_SF Superfamily  (Catalytic domain of glycoside hydrolase (GH) families 38 and 57) | Glyco_hydro_57  (Glycosyl hydrolase family 57) | Cluster 83477  (Alpha-galactosidase (Hydrolase)) | Cluster 4049162  (Glycoside hydrolase/deacetylase) |
|  | HP TPASS_0359 | **B2S2V6** | No hit found | No hit found | No hit found | | No hit found | No hit found | Cluster 131955  (Zn-finger, RING) | Cluster 3431974  Cluster Name: Treponema |
|  | HP TPASS_0360 | **B2S2V7** | No hit found | CSL zinc finger | No hit found | | No hit found | zf-CSL family  (CSL zinc finger) | Cluster 129603  (zinc-finger domain) | Cluster 4051074 |
|  | HP TPASS_0368 | **B2S2W5** | No hit found | No hit found | No hit found | | No hit found | No hit found | Cluster 139775  (Zn-finger, C2H2 type) | Cluster 3964564  Cluster Name: Treponema |
|  | HP TPASS_0369 | **B2S2W6** | No hit found | TPR-like | No hit found | | TrbI superfamily  (Bacterial conjugation TrbI-like protein) | YfiO family  (Outer membrane lipoprotein) | Cluster 154926  (TPR containing protein) | Cluster 3093680  Cluster Name: Treponema |
|  | HP TPASS_0370 | **B2S2W7** | No hit found | No hit found | No hit found | | K_trans superfamily  (K+ potassium transporter) | No hit found | Cluster 156100  (copper ion binding) | Cluster 463337  Cluster Name: Treponema pallidum |
|  | HP TPASS_0371 | **B2S2W8** | 4-diphosphocytidyl-2-C-methyl-D-erythritol kinase -like domain | GHMP Kinase, N-terminal domain | 4-diphosphocytidyl-2-c-methyl-d-erythritol kinase, chloroplastic | | GHMP_kinases_N superfaamily  (GHMP kinases N terminal domain) | GHMP_kinases_N  (GHMP kinases N terminal domain) | Cluster 148064  (4-diphosphocytidyl-2-C-methyl-D-erythritol kinase (EC 2.7.1.148)) | Cluster 4114602  Cluster Name: 4-diphosphocytidyl-2C-methyl-D-erythritol kinase |
|  | HP TPASS_0373 | **B2S2X0** | tRNA(Ile)-lysidine synthase -like domain | tRNA-Ile-lysidine synthetase, TilS, C-terminal domain | Atpases of the pp superfamily-related | | Adenine nucleotide alpha hydrolases superfamily | ATP_bind_3  (PP-loop family) | Cluster 142622  (ATPase of the PP-loop superfamily) | Cluster 4128393  Cluster Name: Lysidine-tRNA(Ile) synthetase, N-terminal |
|  | HP TPASS_0374 | **B2S2X1** | Pre-mRNA-splicing factor CLF1 -like domain | Tetratricopeptide repeat (TPR) | No hit found | | No hit found | TPR family | Cluster 18794  (TPR like ) | Cluster 2802225  Cluster Name: Treponema |
|  | HP TPASS_0375 | **B2S2X2** | No hit found | No hit found | No hit found | | No hit found | No hit found | Cluster 18795 | Cluster 4782485 |
|  | HP TPASS_0376 | **B2S2X3** | No hit found | No hit found | No hit found | | No hit found | No hit found | Cluster 151080  (disulfide bond isomerase) | Cluster 3700962  Cluster Name: Treponema |
|  | HP TPASS_0377 | **B2S2X4** | No hit found | No hit found | No hit found | | No hit found | No hit found | Cluster 63058  (Prokaryotic membrane lipoprotein lipid attachment site) | Cluster 1608606  (Cluster Name: Treponema pallidum) |
|  | HP TPASS_0381 | **B2S2X8** | No hit found | No hit found | No hit found | | Intg_mem_TP0381 superfamily  (Integral membrane protein) | Intg_mem_TP0381 family  (Integral membrane protein) | Cluster 68228  (ABC transporter membrane-spanning permease-amino acid transport) | Cluster 3777859 |
|  | HP TPASS_0382 | **B2S2X9** | No hit found | No hit found | No hit found | | No hit found | No hit found | Cluster 18799 | No hit found |
|  | HP TPASS_0384 | **B2S2Y1** | Ribosomal RNA small subunit methyltransferase H -like domain | S-adenosyl-L-methionine-dependent methyltransferases | Methyltransferase-like protein 15-related | | Methyltransf_5 superfamily | Methyltransf_5 family | Cluster 129839  (S-adenosyl-methyltransferase mraW (EC 2.1.1.-)) | Cluster 3996220  Cluster Name: Bacterial methyltransferase |
|  | HP TPASS_0385 | **B2S2Y2** | No hit found | ABC transporter transmembrane region | No hit found | | No hit found | FtsL family  (Cell division protein) | Cluster 48444  (GCN5-related N-acetyltransferase) | Cluster 4134977 |
|  | HP TPASS_0392 | **B2S2Y9** | Superkiller protein 3 -like domain | TPR-like | Tetratricopeptide repeat protein, tpr | | TPR superfamily | TPR superfamily | Cluster 136427 O-linked N-acetylglucosamine transferase | Cluster 3867008  (Tetratricopeptide region) |
|  | HP TPASS_0404 | **B2S301** | Hydroxyacylglutathione hydrolase -like domain | Metallo-hydrolase/oxidoreductase | No hit found | | No hit found | No hit found | Cluster 97640  (Phosphoglycerate/bisphosphoglycerate mutase) | Cluster 3651136  Cluster Name: Treponema |
|  | HP TPASS_0408 | **B2S305** | No hit found | Apolipoprotein A-I | No hit found | | No hit found | Apolipoprotein family  (Apolipoprotein A1/A4/E domain) | Cluster 110345  (Zn-finger) | Cluster 4300388 |
|  | HP TPASS_0409 | **B2S306** | No hit found | No hit found | No hit found | | No hit found | No hit found | Cluster 127166  (Hedgehog amino-terminal signaling domain) | Cluster 4684398 |
|  | HP TPASS_0412 | **B2S309** | No hit found | No hit found | No hit found | | GluZincin Superfamily | PurA family  (ssDNA and RNA-binding protein) | Cluster 122868  (PUR-alpha/beta/gamma, DNA/RNA-binding) | Cluster 3867122  Cluster Name: PUR-alpha/beta/gamma, DNA/RNA-binding |
|  | HP TPASS_0415 | **B2S312** | No hit found | No hit found | No hit found | | No hit found | No hit found | Cluster 142804  (Sterile alpha motif SAM) | Cluster 443959  Cluster Name: Treponema pallidum |
|  | HP TPASS_0420 | **B2S317** | No hit found | No hit found | No hit found | | No hit found | No hit found | Cluster 18823 | Cluster 615688Cluster Name: Treponema pallidum |
|  | HP TPASS_0421 | **B2S318** | Tripartite motif protein 32 -like domain | TPR like | Trim/rbcc (ring finger, b-box and coiled coil domains-containing) | | TPR super family | TPR family | Cluster 126687  (TPR like ) | Cluster 4036652  Cluster Name: Six-bladed beta-propeller, TolB-like |
|  | HP TPASS_0422 | **B2S319** | No hit found | No hit found | No hit found | | No hit found | No hit found | Cluster 93099  (Phosphoenolpyruvate-dependent sugar phosphotransferase system, EIIA 2) | Cluster 3253844  Cluster Name: Treponema |
|  | HP TPASS_0423 | **B2S320** | Lipid A export ATP-binding/permease protein MsbA -like domain | P-loop containing nucleoside triphosphate hydrolase | No hit found | | ABC_ATPase Superfamily  (ATP-binding cassette transporter nucleotide-binding domain) | AAA_17 family  (ATPases) | Cluster 137470  (ATPases) | Cluster 3677495  Cluster Name: Protein of unknown function DUF322 |
|  | HP TPASS_0425 | **B2S322** | No hit found | No hit found | No hit found | | No hit found | No hit found | Cluster 18827 | Cluster 507206  Cluster Name: Treponema pallidum |
|  | HP TPASS_0431 | **B2S328** | Type III pantothenate kinase -like domain | Actin-like ATPase domain | No hit found | | Pan_kinase superfamily  (Type III pantothenate kinase) | Pan_kinase family  (Type III pantothenate kinase) | Cluster 151538  (Bordetella pertussis Bvg accessory factor) | Cluster 4134483  Cluster Name: Bordetella pertussis Bvg accessory factor  (Pantothenate kinase) |
|  | HP TPASS_0432 | **B2S329** | No hit found | No hit found | No hit found | | No hit found | No hit found | Cluster 154504  (HMG-I and HMG-Y DNA-binding domain) | Cluster 4150849  Cluster Name: Treponema |
|  | HP TPASS_0436 | **B2S332** | 50S ribosomal protein L9 -like domain | DHH phosphoesterases | Trna-nucleotidyltransferase/poly(a) polymerase family member | | DHH superfamily | DHH family | Cluster 142143  (Phosphoesterase, DHHA1) | Cluster 4145529  Cluster Name: Phosphoesterase, RecJ-like |
|  | HP TPASS_0437 | **B2S333** | No hit found | No hit found | No hit found | | No hit found | No hit found | Cluster 18835 | Cluster 3566215  Cluster Name: Treponema |
|  | HP TPASS_0438 | **B2S334** | Non-canonical purine NTP pyrophosphatase -like domain | ITPase-like | Inosine triphosphate pyrophosphatase | | Maf_Ham1 superfamily  (nucleotide binding protein) | Ham1p_like | Cluster 145945  (Interleukin-5 receptor alpha) | Cluster 4100280  Cluster Name: Ham1-like protein  (Acting on acid anhydrides) |
|  | HP TPASS_0441 | **B2S337** | Inorganic polyphosphate/ATP-NAD kinase -like domain | NAD kinase/diacylglycerol kinase-like | Nad kinase | | NAD_kinase superfamily | NAD_kinase family | Cluster 146632  (inorganic polyphosphate/ATP-NAD kinase (EC 2.7.1.23)) | Cluster 4136219  Cluster Name: Probable inorganic polyphosphate/atp-NAD kinase; domain 2 |
|  | HP TPASS_0443 | **B2S339** | No hit found | No hit found | No hit found | | No hit found | No hit found | Cluster 134984  (Desulfoferrodoxin, ferrous iron-binding region) | Cluster 4194551 |
|  | HP TPASS_0444 | **B2S340** | Invasion-associated protein p60 -like domain | LysM domain | Murein hydrolase activator envc | | LysM domain | LysM domain | Cluster 95472  (Peptidoglycan-binding LysM) | Cluster 4093645  Cluster Name: Peptidoglycan-binding Lysin subgroup |
|  | HP TPASS_0447 | **B2S343** | Tetratricopeptide repeat protein -like domain | TPR like | No hit found | | No hit found | TPR_11 family | Cluster 18841  (TPR containing protein) | Cluster 4153415  (Serine Threonine Protein Phosphatase 5, Tetratricopeptide repeat) |
|  | HP TPASS_0449 | **B2S345** | Bacteriophage N4 adsorption protein A -like domain | TPR-like | Tetratricopeptide repeat protein, tpr | | TPR superfamily | TPR family | Cluster 126125  (O-linked N-acetylglucosamine transferase) | Cluster 3431754  Cluster Name: Treponema  (TPR) |
|  | HP TPASS_0451 | **B2S347** | No hit found | No hit found | No hit found | | No hit found | No hit found | Cluster 18843 | Cluster 4648260 |
|  | HP TPASS_0453 | **B2S349** | No hit found | No hit found | No hit found | | No hit found | No hit found | Cluster 123088  (Exodeoxyribonuclease V, gamma) | Cluster 3254024  Cluster Name: Treponema |
|  | HP TPASS_0454 | **B2S350** | No hit found | CheY-like | No hit found | | REC superfamily  (Signal receiver domain) | No hit found | Cluster 111328  (Response regulator receiver) | Cluster 3416065  Cluster Name: Treponema |
|  | HP TPASS_0455 | **B2S351** | No hit found | Atu1913-like | No hit found | | No hit found | No hit found | Cluster 122874 | Cluster 4260580 |
|  | HP TPASS_0456 | **B2S352** | No hit found | No hit found | No hit found | | No hit found | No hit found | Cluster 30043  (DNA binding protein) | Cluster 3589536  Cluster Name: Treponema |
|  | HP TPASS_0457 | **B2S353** | No hit found | No hit found | No hit found | | No hit found | No hit found | Cluster 30043  (DNA binding protein) | Cluster 3570033 |
|  | HP TPASS_0458 | **B2S354** | Segregation and condensation protein B -like domain | "Winged helix" DNA-binding domain |  | | DUF387 superfamily  (transcriptional regulators (Ypuh-like)) | DUF387 family  (transcriptional regulators (Ypuh-like)) | Cluster 142721  (regulation of transcription, DNA-dependen) | Cluster 3979682  Cluster Name: Prokaryotic chromosome segregation and condensation protein ScpB |
|  | HP TPASS_0459 | **B2S355** | 30S ribosomal protein S4 -like domain | Pseudouridine synthase | Ribosomal large subunit pseudouridine synthase b-related | | PseudoU_synthsuperfamily  (RNA pseudouridylate synthase) | PseudoU_synth_2family  (RNA pseudouridylate synthase) | Cluster 141200  (ribosomal pseudouridine synthase (EC 4.2.1.70)) | Cluster 4152309  Cluster Name: Pseudouridine synthase, RsuA and RluB/E/F |
|  | HP TPASS_0460 | **B2S356** | Bardet-Biedl syndrome 4 protein -like domain | TPR-like | Tetratricopeptide repeat protein, tpr | | TPR superfamily | TPR family | Cluster 99844  (TPR) | Cluster 4108596  Cluster Name: TPR repeat |
|  | HP TPASS_0461 | **B2S357** | DNA-binding protein -like domain | lambda repressor-like DNA-binding domains |  | | HTH_XRE superfamily  (Helix-turn-helix XRE-family like protein) | HTH_3 family  (Helix-turn-helix) | Cluster 146072  (Helix-turn-helix motif) | Cluster 4129341  Cluster Name: Helix-turn-helix type 3 |
|  | HP TPASS_0462 | **B2S358** | No hit found | No hit found | No hit found | | No hit found | No hit found | Cluster 133274 | Cluster 4078211  (Cluster Name: Treponema pallidum) |
|  | HP TPASS_0463 | **B2S359** | No hit found | No hit found | No hit found | | No hit found | No hit found | Cluster 133274  (Prokaryotic membrane lipoprotein lipid attachment site) | Cluster 4078211  (Cluster Name: Treponema pallidum) |
|  | HP TPASS_0464 | **B2S360** | tRNA (guanine-N(7)-)-methyltransferase -like domain | S-adenosyl-L-methionine-dependent methyltransferases | Trna (guanine-n(7)-)-methyltransferase | | AdoMetr_Mtases superfamily  (S-adenosylmethionine-dependent methyltransferases) | Methyltransf_4 family  (Putative methyltransferase) | Cluster 143554  (ABC transporter, periplasmic iron-compound-binding protein) | Cluster 4167817  Cluster Name: TRNA (guanine-N(7)-)-methyltransferase |
|  | HP TPASS_0465 | **B2S361** | No hit found | No hit found | No hit found | | No hit found | No hit found | Cluster 151015  (kinase activity) | Cluster 3931471  Cluster Name: Treponema  (Binding protein) |
|  | HP TPASS_0466 | **B2S362** | No hit found | No hit found | No hit found | | No hit found | No hit found | Cluster 133085  (DNA helicase) | Cluster 706448  Cluster Name: Treponema pallidum |
|  | HP TPASS_0467 | **B2S363** | No hit found | No hit found | No hit found | | No hit found | No hit found | Cluster 138616  (Zn-finger, C2H2 type) | Cluster 615788  Cluster Name: Treponema pallidum |
|  | HP TPASS_0468 | **B2S364** | Ogt protein -like domain | TPR-like | Tetratricopeptide repeat protein, tpr | | TPR superfamily | TPR family | Cluster 136427  (TPR-domain containing protein) | Cluster 4092293  Cluster Name: TPR repeat |
|  | HP TPASS_0470 | **B2S365** | Superkiller protein 3 -like domain | TPR-like | Tetratricopeptide repeat protein, tpr | | TPR superfamily | TPR superfamily | Cluster 136490 (TPR) | Cluster 3467383  Cluster Name: Treponema  (TPR) |
|  | HP TPASS_0471 | **B2S366** | No hit found | TPR-like | Tetratricopeptide repeat protein, tpr | | TPR superfamily | TPR superfamily | Cluster 136899  (TPR) | Cluster 3577596  Cluster Name: DNA restriction-modification system |
|  | HP TPASS_0473 | **B2S368** | No hit found | No hit found | No hit found | | No hit found | RseC_MucC family  (Positive regulator of sigma(E)) | Cluster 18854 | Cluster 4080564Cluster Name: Treponema |
|  | HP TPASS_0474 | **B2S369** | UPF0082 protein At2g25830 -like domain | YebC-like | Transcriptional regulatory protein yebc-related | | Transcrip_reg superfamily  (Transcriptional regulator) | Transcrip_reg family  (Transcriptional regulator) | Cluster 152835  (YebC like) | Cluster 4120159  (YebC like) |
|  | HP TPASS_0479 | **B2S374** | No hit found | Autotransporter | No hit found | | Protein of unknown function (DUF2715) | Protein of unknown function (DUF2715) | Cluster 156020 | Cluster 4140084  Cluster Name: Treponema pallidum |
|  | HP TPASS_0480 | **B2S375** | No hit found | No hit found | No hit found | | ApoLp-III_like Superfamily  (Apolipophorin-III) | No hit found | Cluster 18857 | Cluster 4057862 |
|  | HP TPASS_0481 | **B2S376** | No hit found | No hit found | No hit found | | ApoLp-III_like Superfamily  (Apolipophorin-III) | CCT_2 family  (Divergent CCT motif) | Cluster 79003  () | Cluster 2787041 |
|  | HP TPASS_0482 | **B2S377** | No hit found | No hit found | No hit found | | ApoLp-III_like Superfamily  (Apolipophorin-III) | CHASE3  domain | Cluster 137519 (dehydrogenase) | Cluster 398493  Cluster Name: Treponema pallidum |
|  | HP TPASS_0484 | **B2S379** | No hit found | No hit found | No hit found | | FecR superfamily | FecR domain | Cluster 143100  (23S ribosomal RNA methyltransferase (EC 2.1.1.-)) | Cluster 4071305 |
|  | HP TPASS_0487 | **B2S382** | No hit found | Quinoprotein alcohol dehydrogenase-like | No hit found | | No hit found | HEAT repeats | Cluster 83620  (G-protein beta WD-40 repeat) | Cluster 3375777  Cluster Name: Quinoprotein alcohol dehydrogenase-like |
|  | HP TPASS_0489 | **B2S384** | Hydroxyacylglutathione hydrolase -like domain | Metallo-hydrolase/oxidoreductase | Ribonuclease Z | | Lactamase_B superfamily | Lactamase_B_2 family | Cluster 149634  (Metallo-beta-lactamase superfamily protein) | Cluster 4122352  Cluster Name: Beta-lactamase-like |
|  | HP TPASS_0490 | **B2S385** | No hit found | No hit found | No hit found | | No hit found | No hit found | Cluster 156912  (DNA maturase B) | Cluster 4524287  Cluster Name: Cytochrome p450 |
|  | HP TPASS_0491 | **B2S386** | No hit found | No hit found | Yceg | | YceG superfamily | YceG family | Cluster 152447  (Aminodeoxychrorismate lyase) | Cluster 4125861  Cluster Name: Aminodeoxychorismate lyase |
|  | HP TPASS_0494 | **B2S389** | No hit found | No hit found | No hit found | | ApoLp-III_like Superfamily  (Apolipophorin-III) | DUF164  (zinc ribbon domain) | Cluster 110345  (Zinc finger) | Cluster 4118956 |
|  | HP TPASS_0496 | **B2S390** | Slr2048 protein -like domain | TPR-like | Tetratricopeptide repeat protein, tpr | | TPR superfamily | TPR family | Cluster 108626  (O-linked N-acetylglucosamine transferase) | Cluster 4084609  (TPR) |
|  | HP TPASS_0502 | **B2S396** | Mib2 protein -like domain | Ankyrin repeat | Ankyrin repeat protein | | ANK superfamily | Ank_2 family | Cluster 96385  (Ankyrin repeats) | Cluster 4149077  (Ankyrin repeat) |
|  | HP TPASS_0503 | **B2S397** | No hit found | No hit found | No hit found | | No hit found | No hit found | Cluster 88463  (GCN5-related N-acetyltransferase) | Cluster 3483457  Cluster Name: Treponema  (Acyl-CoA N-acyltransferase) |
|  | HP TPASS_0504 | **B2S398** | No hit found | No hit found | No hit found | | No hit found | No hit found | Cluster 18922 | No hit found |
|  | HP TPASS_0512 | **B2S3A6** | 2-C-methyl-D-erythritol 2,4-cyclodiphosphate -like domain | Nucleotide-diphospho-sugar transferases | : 2-c-methyl-d-erythritol 4-phosphate cytidylyltransferase, chloroplastic | | MECDP_synthase Superfamily  (2-C-methyl-D-erythritol-2,4-cyclodiphosphate synthase) | IspD family  (2-C-methyl-D-erythritol 4-phosphate cytidylyltransferase) | Cluster 142896  (4-diphosphocytidyl-2C-methyl-D-erythritol synthase) | Cluster 4161259  Cluster Name: 2-C-methyl-D-erythritol 4-phosphate cytidylyltransferase |
|  | HP TPASS_0515 | **B2S3A9** | No hit found | No hit found | No hit found | | OstA_C superfamily  (Organic solvent tolerance protein) | OstA_C family  (Organic solvent tolerance protein) | Cluster 122881  (Organic solvent tolerance protein) | Cluster 3466459  Cluster Name: Response to organic substance |
|  | HP TPASS_0518 | **B2S3B2** | Thiamine pyrophosphokinase -like domain | Thiamin pyrophosphokinase, catalytic domain | No hit found | | TPK superfamily  (Thiamin pyrophosphokinase, catalytic domain) | TPK_catalytic family  (Thiamin pyrophosphokinase, catalytic domain) | Cluster 150121  (Thiamin pyrophosphokinase, catalytic domain) | Cluster 4136084  Cluster Name: Thiamin pyrophosphokinase |
|  | HP TPASS_0522 | **B2S3B5** | No hit found | No hit found | No hit found | | Colicin_V superfamily  (Colicin V production protein) | Colicin_V family  (Colicin V production protein) | Cluster 154958  (Colicin V production protein) | Cluster 3625939  Cluster Name: Toxin biosynthetic process  (Colicin V production protein) |
|  | HP TPASS_0534 | **B2S3C6** | No hit found | V-type ATP synthase subunit C | No hit found | | No hit found | No hit found | Cluster 153388  (V-type ATP synthase subunit C (EC 3.6.3.14)) | Cluster 507311  Cluster Name: V-type ATP synthase subunit C |
|  | HP TPASS_0535 | **B2S3C7** | No hit found | No hit found | No hit found | | No hit found | ATP-synt_B  Family  (ATP synthase B/B’) | Cluster 142845  (UvrD/REP helicase) | Cluster 4563858 |
|  | HP TPASS_0539 | **B2S3D1** | No hit found | No hit found | No hit found | | No hit found | No hit found | Cluster 18945 | Cluster 281847  Cluster Name: Treponema pallidum |
|  | HP TPASS_0544 | **B2S3D6** | Inpp5b protein -like domain | DNase I-like | No hit found | | EEP superfamily  (Endonuclease/Exonuclease/phosphatase family) | Exo_endo_phos family  (Endonuclease/Exonuclease/phosphatase family) | Cluster 128184  (Endonuclease/exonuclease/phosphatase) | Cluster 4076007  Cluster Name: DNase I-like |
|  | HP TPASS_0548 | **B2S3E0** | No hit found | TPR-like | No hit found | | TPR superfamily | TPR family | Cluster 140031  (TPR containing ptrotein) | Cluster 3609927 |
|  | HP TPASS_0552 | **B2S3E4** | No hit found | No hit found | No hit found | | No hit found | Gag_MA family  (Matrix protein (MA)) | Cluster 125354  (ATPase) | Cluster 3221413  Cluster Name: Treponema |
|  | HP TPASS_0553 | **B2S3E5** | No hit found | No hit found | Transmembrane protein | | GAF domain | No hit found | Cluster 140156  (Membrane protein) | Cluster 4146314 |
|  | HP TPASS_0557 | **B2S3E9** | No hit found | No hit found | No hit found | | NicO superfamily  (High-affinity nickel-transport protein) | zf-CHCC  (Zinc finger domain) | Cluster 156272 | Cluster 4119811  ([Homoserine kinase](http://www.expasy.org/enzyme/2.7.1.39)) |
|  | HP TPASS_0558 | **B2S3F0** | No hit found | No hit found | No hit found | | NicO superfamily  (High-affinity nickel-transport protein) | NicO family  (High-affinity nickel-transport protein) | Cluster 150572  (High-affinity nickel-transport protein) | Cluster 4173000  Cluster Name: Nickel/cobalt transporter, high-affinity |
|  | HP TPASS_0561 | **B2S3F3** | No hit found | No hit found | No hit found | | TPM superfamily  (TLP18.3, Psb32 and MOLO-1 founding proteins of phosphatase) | TPM family  (TLP18.3, Psb32 and MOLO-1 founding proteins of phosphatase) | Cluster 150440  (Mitochondrial substrate carrie) | Cluster 4156723 |
|  | HP TPASS_0563 | **B2S3F5** | DNAJ protein -like domain | Chaperone J-domain | No hit found | | DnaJ superfamily | DnaJ domain | Cluster 113125  (Molecular chaperone, DnaJ family (contain C-term. Zn finger domain)) | Cluster 4080075  Cluster Name: Heat shock protein binding |
|  | HP TPASS_0564 | **B2S3F6** | No hit found | No hit found | No hit found | | No hit found | No hit found | Cluster 18963 | Cluster 2974818  Cluster Name: Treponema |
|  | HP TPASS_0565 | **B2S3F7** | Isoamyl acetate-hydrolyzing esterase -like domain | SGNH hydrolase | No hit found | | SGNH_hydrolase superfamily | No hit found | Cluster 139717  (NAD-dependent formate dehydrogenase alpha subunit protein (EC 1.2.1.2)) | Cluster 4026282  (SGNH hydrolase-type) |
|  | HP TPASS_0567 | **B2S3F9** | No hit found | MgtE N-terminal domain-like | No hit found | | MgtE_N superfamily  (MgtE intracellular N domain) | MgtE_N family  (MgtE intracellular N domain) | Cluster 130925  (flageller protein) | Cluster 4061113  Cluster Name: Flagellum |
|  | HP TPASS_0570 | **B2S3G2** | No hit found | No hit found | No hit found | | TPM superfamily  (TLP18.3, Psb32 and MOLO-1 founding proteins of phosphatase) | TPM family  (TLP18.3, Psb32 and MOLO-1 founding proteins of phosphatase) | Cluster 152102 | Cluster 4159982 |
|  | HP TPASS_0572 | **B2S3G4** | No hit found | No hit found | No hit found | | Ferric_reduct family  (Ferric reductase like transmembrane component) | Ferric_reduct family  (Ferric reductase like transmembrane component) | Cluster 129281  (FMN binding domain) | Cluster 4047101  Cluster Name: FAD binding |
|  | HP TPASS_0573 | **B2S3G5** | No hit found | No hit found | No hit found | | No hit found | No hit found | Cluster 18965 | No hit found |
|  | HP TPASS_0577 | **B2S3G8** | No hit found | Carboxypeptidase regulatory domain-like | No hit found | | No hit found | No hit found | Cluster 118729  (ATP binding) | Cluster 3203780  Cluster Name: Treponema |
|  | HP TPASS_0579 | **B2S3H0** | No hit found | No hit found | No hit found | | No hit found | No hit found | Cluster 113106  (Heat shock protein DnaJ, N-terminal) | Cluster 4065722  Cluster Name: Treponema |
|  | HP TPASS_0580 | **B2S3H1** | No hit found | No hit found | Lipoprotein-releasing system transmembrane protein lole | | FtsX superfamily  (FtsX-like permease family) | FtsX family  (FtsX-like permease family) | Cluster 141137  (Lipoprotein releasing system transmembrane protein lolE) | Cluster 4163048  Cluster Name: Lipoprotein releasing system, transmembrane protein, LolC/E family |
|  | HP TPASS_0582 | **B2S3H3** | No hit found | Multidrug efflux transporter AcrB transmembrane domain | Lipoprotein-releasing system transmembrane protein lole | | FtsX superfamily  (FtsX-like permease family) | FtsX family  (FtsX-like permease family) | Cluster 141137  (Lipoprotein releasing system transmembrane protein lolE) | Cluster 4163048  Cluster Name: Lipoprotein releasing system, transmembrane protein, LolC/E family |
|  | HP TPASS_0583 | **B2S3H4** | No hit found | No hit found | No hit found | | No hit found | No hit found | Cluster 18973 | Cluster 333630  Cluster Name: Treponema pallidum |
|  | HP TPASS_0584 | **B2S3H5** | No hit found | NinB | No hit found | | No hit found | Baculo_PEP_C  (Baculovirus polyhedron envelope protein, PEP, C terminus) | Cluster 108217  (sporulation related) | Cluster 2709769  Cluster Name: Treponema |
|  | HP TPASS_0587 | **B2S3H8** | No hit found | DNA polymerase III clamp loader subunits, C-terminal domain | No hit found | | No hit found | No hit found | Cluster 18980 | Cluster 4491782  Cluster Name: DNA polymerase III, delta |
|  | HP TPASS_0588 | **B2S3H9** | No hit found | P-loop containing nucleoside triphosphate hydrolases | No hit found | | DNA_pol3_delta  superfamily  (DNA polymerase III, delta subunit) | DNA_pol3_delta  (DNA polymerase III, delta subunit) | Cluster 152121  (DNA polymerase III delta subunit) | Cluster 3834784  Cluster Name: DNA polymerase III, delta |
|  | HP TPASS_0590 | **B2S3I1** | No hit found | No hit found | No hit found | | No hit found | No hit found | Cluster 59236  (Ribosomal protein) | No hit found |
|  | HP TPASS_0592 | **B2S3I3** | No hit found | Hedgehog/DD-peptidase | No hit found | | Peptidase_M15_4  superfamily  (D-alanyl-D-alanine carboxypeptidase) | Peptidase_M15_4  family  (D-alanyl-D-alanine carboxypeptidase) | Cluster 103027  (Peptidase M15B and M15C) | Cluster 4094238 |
|  | HP TPASS_0593 | **B2S3I4** | No hit found | No hit found | No hit found | | DRE_TIM_metallolyase Superfamily | No hit found | Cluster 18982 | Cluster 3119649  Cluster Name: Treponema |
|  | HP TPASS_0594 | **B2S3I5** | No hit found | No hit found | No hit found | | EIIC_GAT superfamily  (PTS system sugar-specific permease component) | No hit found | Cluster 157432  (phosphoenolpyruvate-dependent sugar phosphotransferase system) | Cluster 4064324 |
|  | HP TPASS_0598 | **B2S3I8** | Bub3 protein -like domain | C-terminal (heme d1) domain of cytochrome cd1-nitrite reductase | No hit found | | No hit found | No hit found | Cluster 128424  (WD repeat protein) | Cluster 2817746  Cluster Name: Treponema  (Methylamine Dehydrogenase; Chain H) |
|  | HP TPASS_0599 | **B2S3I9** | No hit found | No hit found | No hit found | | CpXC domain | CpXC domain | Cluster 18986  (Zn-finger, C2H2 type) | Cluster 3207749  Cluster Name: Treponema  (Zn-finger, C2H2 like) |
|  | HP TPASS_0607 | **B2S3J7** | No hit found | No hit found | No hit found | | No hit found | No hit found | Cluster 18998 | Cluster 463540  Cluster Name: Treponema pallidum |
|  | HP TPASS_0608 | **B2S3J8** | 26S proteasome regulatory subunit RPN2 -like domain | ARM repeat | No hit found | | No hit found | HEAT_2 family  (HEAT repeats) | Cluster 18999  (ARM repeat fold) | Cluster 3024622  (Armadillo-type fold) |
|  | HP TPASS_0612 | **B2S3K2** | No hit found | Stabilizer of iron transporter SufD | Fes cluster assembly protein suf | | Autotransporter superfamily | Cytochrome_CBB3  Family  (Cytochrome C oxidase, cbb3-type, subunit III) | Cluster 141538  (SufD protein) | Cluster 4076178  Cluster Name: SUF system FeS cluster assembly, SufBD |
|  | HP TPASS_0613 | **B2S3K3** | No hit found | Stabilizer of iron transporter SufD | Fes cluster assembly protein suf | | Autotransporter superfamily | No hit found | Cluster 141538  (SufD protein) | Cluster 3998922  Cluster Name: SUF system FeS cluster assembly, SufBD |
|  | HP TPASS_0617 | **B2S3K7** | No hit found | No hit found | No hit found | | Protein of unknown function (DUF2715) | Protein of unknown function (DUF2715) | Cluster 156019 | Cluster 4140084  Cluster Name: Treponema pallidum |
|  | HP TPASS_0618 | **B2S3K8** | No hit found | No hit found | No hit found | | Protein of unknown function (DUF2715) | Protein of unknown function (DUF2715) | Cluster 132834  (Inulin fructotransferase (EC 2.4.1.93)) | Cluster 4140084  Cluster Name: Treponema pallidum |
|  | HP TPASS_0619 | **B2S3K9** | No hit found | No hit found | No hit found | | Protein of unknown function (DUF2715) | Protein of unknown function (DUF2715) | Cluster 157940  (Integral membrane protein) | Cluster 4140084  Cluster Name: Treponema pallidum |
|  | HP TPASS_0622 | **B2S3L2** | Mitochondria fission 1 protein -like domain | TPR like | Tetratricopeptide repeat protein, tpr | | TPR superfamily | TPR family | Cluster 47268  (TPR containing protein) | Cluster 2676446  Cluster Name: Treponema  (Tetratricopeptide repeat) |
|  | HP TPASS_0624 | **B2S3L4** | Outer membrane protein -like domain | OmpA-like | Outer membrane protein, ompa-related | | OmpA_C like superfamily  (Peptidoglycan binding domain) | OmpA family | Cluster 92512  (OmpA/MotB domain) | Cluster 3740351  Cluster Name: OmpA-like |
|  | HP TPASS_0625 | **B2S3L5** | No hit found | TPR-like | No hit found | | No hit found | TPR family | Cluster 19007  (TPR like) | Cluster 3700961  Cluster Name: Treponema |
|  | HP TPASS_0629 | **B2S3L9** | No hit found | No hit found | No hit found | | No hit found | No hit found | Cluster 19010 | Cluster 4037298 |
|  | HP TPASS_0636 | **B2S3M5** | No hit found | ArfGap/RecO-like zinc finger | No hit found | | RecO_C superfamily  (Recombination protein O C terminal) | RecO_C family  (Recombination protein O C terminal) | Cluster 47269  (Recombination protein O, RecO) | Cluster 3114818  (Recombination protein O, RecO) |
|  | HP TPASS_0638 | **B2S3M7** | No hit found | Cytochrome c oxidase subunit III-like | No hit found | | No hit found | MotB_plug family  (Membrane MotB of proton-channel complex MotA/MotB) | Cluster 19014 | Cluster 3732019  Cluster Name: Treponema |
|  | HP TPASS_0645 | **B2S3N4** | No hit found | No hit found | No hit found | | No hit found | No hit found | Cluster 19018 | No hit found |
|  | HP TPASS_0646 | **B2S3N5** | Tripartite motif protein 32 -like domain | YWTD domain | No hit found | | No hit found | NHL repeats | Cluster 137946  (Tripartite motif protein 3 (RING finger protein 22)) | Cluster 3789835  Cluster Name: TolB, C-terminal domain  ( Neuraminidase) |
|  | HP TPASS_0648 | **B2S3N7** | Superkiller protein 3 -like domain | TPR like | Tetratricopeptide repeat protein, tpr | | TPR superfamily | TPR family | Cluster 101313  (TPR like ) | Cluster 3570849  Cluster Name: TPR repeat |
|  | HP TPASS_0651 | **B2S3P0** | Ribonuclease Y -like domain | HD-domain/PDEase-like | No hit found | | 7TM-7TMR_HD superfamily  (7TM receptor with intracellular HD hydrolase) | 7TM-7TMR_HD family  (7TM receptor with intracellular HD hydrolase) | Cluster 130943  (Metal-dependent phosphohydrolase, HD region) | Cluster 4067410  Cluster Name: Metal-dependent phosphohydrolase, 7TM intracellular region |
|  | HP TPASS_0656 | **B2S3P5** | No hit found | No hit found | No hit found | | No hit found | No hit found | Cluster 19023 | No hit found |
|  | HP TPASS_0661 | **B2S3Q0** | No hit found | FlgN-like | No hit found | | No hit found | No hit found | Cluster 136839  (Tyrosine protein kinase) | Cluster 3723092  Cluster Name: Treponema |
|  | HP TPASS_0665 | **B2S3Q4** | No hit found | No hit found | No hit found | | No hit found | No hit found | Cluster 19025 | Cluster 3903096 |
|  | HP TPASS_0666 | **B2S3Q5** | No hit found | No hit found | No hit found | | No hit found | No hit found | Cluster 127037  (Heat shock protein DnaJ, N-terminal) | Cluster 422779  Cluster Name: Treponema pallidum |
|  | HP TPASS_0668 | **B2S3Q7** | No hit found | Nuclear receptor ligand-binding domain | No hit found | | YGGT superfamily | YGGT family | Cluster 154128  (Bacterial regulatory protein, LysR) | Cluster 3986183 |
|  | HP TPASS_0674 | **B2S3R3** | No hit found | SMR domain like | No hit found | | Smr superfamily | Smr domain | Cluster 147430  (SMR/MUTS family protein) | Cluster 4062048  Cluster Name: Smr protein/MutS2 C-terminal |
|  | HP TPASS_0675 | **B2S3R4** | No hit found | No hit found | No hit found | | TraB superfamily | TraB family | Cluster 113511  (kinase) | Cluster 3337702  Cluster Name: GumN |
|  | HP TPASS_0676 | **B2S3R5** | No hit found | No hit found | No hit found | | No hit found | No hit found | Cluster 137715  (membrane related protein) | Cluster 4161234 |
|  | HP TPASS_0677 | **B2S3R6** | No hit found | No hit found | No hit found | | No hit found | No hit found | Cluster 155119  (Glutamine synthetase adenylyltransferase glnE (EC 2.7.7.42)) | Cluster 3619680Cluster Name: Treponema |
|  | HP TPASS_0678 | **B2S3R7** | No hit found | PLP-dependent transferases | No hit found | | No hit found | Acetyltransf_6 family  (Acetyltransferase (GNAT) domain) | Cluster 142260  (Nucleoprotein) | Cluster 3983871 |
|  | HP TPASS_0679 | **B2S3R8** | No hit found | No hit found | No hit found | | No hit found | 7TMR-DISM_7TM family  (7TM diverse intracellular signalling) | Cluster 136102  (ABC-type transporter, permease) | Cluster 4442971 |
|  | HP TPASS_0690 | **B2S3S9** | No hit found | No hit found | No hit found | | No hit found | No hit found | Cluster 115920  (regulation of transcription, DNA-dependent) | Cluster 3816281 |
|  | HP TPASS_0691 | **B2S3T0** | No hit found | "Winged helix" DNA-binding domain | No hit found | | ScpA_ScpB superfamily  (chromosomal partition during cell division) | ScpA_ScpB family | Cluster 143281  (Bipartite response regulator, C-terminal effector) | Cluster 4138545  Cluster Name: Prokaryotic chromosome segregation and condensation protein ScpA |
|  | HP TPASS_0693 | **B2S3T2** | No hit found | No hit found | No hit found | | No hit found | No hit found | Cluster 89835  (Phosphopantetheinyl transferase) | Cluster 4140762 |
|  | HP TPASS_0697 | **B2S3T6** | No hit found | No hit found | No hit found | | Protein of unknown function (DUF2715) | Protein of unknown function (DUF2715) | Cluster 156925  (Phage portal protein, lambda family) | Cluster 4140084  Cluster Name: Treponema pallidum |
|  | HP TPASS_0698 | **B2S3T7** | No hit found | No hit found | No hit found | | Protein of unknown function (DUF2715) | Protein of unknown function (DUF2715) | Cluster 24419  (Lipase) | Cluster 4140084  Cluster Name: Treponema pallidum |
|  | HP TPASS_0699 | **B2S3T8** | No hit found | No hit found | No hit found | | No hit found | MerR_1 family  (MerR HTH family regulatory protein) | Cluster 143291  (Transcriptional regulator, MerR family) | No hit found |
|  | HP TPASS_0700 | **B2S3T9** | No hit found | No hit found | No hit found | | No hit found | No hit found | Cluster 19046 | Cluster 3891900  Cluster Name: Treponema |
|  | HP TPASS_0702 | **B2S3U1** | Lipoprotein -like domain | Duplicated hybrid motif | Peptidase-related | | Peptidase_M23 superfamily | Peptidase_M23 family | Cluster 102704  Peptidase M23B | Cluster 4184571  Cluster Name: Peptidase M23B |
|  | HP TPASS_0703 | **B2S3U2** | No hit found | No hit found | No hit found | | No hit found | No hit found | Cluster 141328  (Ribosomal protein S11) | Cluster 802382  Cluster Name: Treponema pallidum |
|  | HP TPASS_0706 | **B2S3U5** | Lipoprotein -like domain | Duplicated hybrid motif | Peptidase-related | | Peptidase_M23 superfamily | Peptidase_M23 family | Cluster 138458  Peptidase M23B | Cluster 4184571  Cluster Name: Peptidase M23B |
|  | HP TPASS_0707 | **B2S3U6** | No hit found | ClpP/crotonase | No hit found | | No hit found | No hit found | Cluster 148681  (Cytochrome c assembly protein) | Cluster 838807  Cluster Name: Treponema pallidum |
|  | HP TPASS_0708 | **B2S3U7** | No hit found | No hit found | No hit found | | No hit found | No hit found | Cluster 128015  (Zn-finger, ZZ type) | Cluster 296216 |
|  | HP TPASS_0710 | **B2S3U9** | RNA-binding protein -like domain | Cell-division inhibitor MinC, C-terminal domain | No hit found | | Jag_N superfamily | Jag_N family | Cluster 139398  (Chromosome segregation protein) | Cluster 4169838 |
|  | HP TPASS_0711 | **B2S3V0** | No hit found | No hit found | No hit found | | No hit found | No hit found | Cluster 138083  (Hydrolase) | Cluster 4212589  Cluster Name: Integral to membrane |
|  | HP TPASS_0719 | **B2S3V8** | No hit found | No hit found | No hit found | | FliO superfamily  (Flagellar biosynthesis protein, FliO) | FliO family  (Flagellar biosynthesis protein, FliO) | No hit found | Cluster 3656865  Cluster Name: Flagellar biosynthesis protein, FliO |
|  | HP TPASS_0723 | **B2S3W2** | No hit found | No hit found | No hit found | | No hit found | No hit found | Cluster 19064 | Cluster 4660939 |
|  | HP TPASS_0730 | **B2S3W9** | No hit found | No hit found | Cdp-diacylglycerol--glycerol-3-phosphate 3-phosphatidyltransferase-related | | CDP-OH_P_transf superfamily  (CDP-alcohol phosphatidyltransferase) | CDP-OH_P_transf family  (CDP-alcohol phosphatidyltransferase) | Cluster 146750 (CDP-diacylglycerol--GLYCEROL-3-PHOSPHATE 3-phosphatidyltransferase) | Cluster 3355292  Cluster Name: CDP-alcohol phosphatidyltransferase |
|  | HP TPASS_0731 | **B2S3X0** | RNA pyrophosphohydrolase -like domain | Nudix | Udp/adp-sugar pyrophosphatase | | Nudix_hydrolase superfamily | NUDIX domain | Cluster 147294  (NTP pyrophosphatase) | Cluster 4174362  Cluster Name: Nucleoside Triphosphate Pyrophosphohydrolase |
|  | HP TPASS_0733 | **B2S3X2** | Outer membrane protein -like domain | OMPA like | No hit found | | OMP_b-brl superfamily  (Outer membrane protein beta-barrel domain) | OMP_b-brl family  (Outer membrane protein beta-barrel domain) | Cluster 155363  (Outer membrane protein) | Cluster 3822473  Cluster Name: Treponema |
|  | HP TPASS_0738 | **B2S3X7** | ATPase synthesis protein 25, mitochondrial -like domain | Nucleotidetransferase | Mitochondrial assembly of ribosomal large subunit protein 1 | | Oligomerisation superfamily  (oligomerization of ATP synthase subunit 9 into a ring structure) | Oligomerisation domain | Cluster 153016  (Iojap-related protein) | Cluster 4170010  Cluster Name: Iojap-related protein |
|  | HP TPASS_0739 | **B2S3X8** | No hit found | No hit found | No hit found | | LytR_cpsA_psr superfamily  (Cell envelope-related transcriptional attenuator domain) | LytR_cpsA_psr family  (Cell envelope-related transcriptional attenuator domain) | Cluster 113616  (Cell envelope-related transcriptional attenuator) | Cluster 4125386 |
|  | HP TPASS_0740 | **B2S3X9** | CRISPR-associated helicase Cas3 -like domain | HD-domain/PDEase-like | No hit found | | HDc superfamily  (Metal dependent phosphohydrolases with conserved 'HD' motif) | HD domain | Cluster 126106  (Metal-dependent phosphohydrolase, HD subdomain) | Cluster 4075414  (Metal-dependent phosphohydrolase, HD region) |
|  | HP TPASS_0741 | **B2S3Y0** | Phosphopantetheine adenylyltransferase -like domain | Nucleotidylyl transferase | Nicotinamide mononucleotide adenylyltransferase | | Nt_trans superfamily  (nucleotidyl transferase) | CTP_transf_2 family  (Cytidylyltransferase) | Cluster 150173  (nicotinate-nucleotide adenylyltransferase (EC 2.7.7.18)) | Cluster 4163185  Cluster Name: Probable nicotinate-nucleotide adenylyltransferase |
|  | HP TPASS_0744 | **B2S3Y3** | No hit found | TM1457 like | No hit found | | Protein of unknown function (DUF464) | Protein of unknown function (DUF464) | Cluster 156046  (ribosomal protein) | Cluster 3988221 |
|  | HP TPASS_0747 | **B2S3Y6** | No hit found | No hit found | No hit found | | No hit found | No hit found | Cluster 139819  (ATP-dependent helicase) | Cluster 528461  Cluster Name: Treponema pallidum |
|  | HP TPASS_0749 | **B2S3Y8** | No hit found | No hit found | No hit found | | No hit found | No hit found | Cluster 19069 | Cluster 374918  Cluster Name: Treponema pallidum |
|  | HP TPASS_0750 | **B2S3Y9** | Collagen alpha-3(VI) chain -like domain | vWA-like | No hit found | | vWFA superfamily  (Von Willebrand factor type A) | VWA family  (von Willebrand factor type A domain) | Cluster 120154  (von Willebrand factor, type A) | Cluster 2998529  (Von Willebrand factor, type A) |
|  | HP TPASS_0752 | **B2S3Z1** | No hit found | Sporulation related repeat | No hit found | | No hit found | SPOR family  (Sporulation related domain) | Cluster 3474432 | Cluster 3474432 |
|  | HP TPASS_0753 | **B2S3Z2** | No hit found | No hit found | No hit found | | No hit found | No hit found | Cluster 134583  (Glutaconyl-CoA decarboxylase delta subunit (EC 4.1.1.70)) | Cluster 3892129  Cluster Name: Treponema |
|  | HP TPASS_0759 | **B2S3Z8** | No hit found | No hit found | No hit found | | No hit found | No hit found | Cluster 19080 | No hit found |
|  | HP TPASS_0761 | **B2S400** | No hit found | No hit found | No hit found | | HDc superfamily  (Metal dependent phosphohydrolases with conserved 'HD' motif) | Clathrin_H_link family | Cluster 145565  (Nucleic acid-binding OB-fold) | Cluster 4087527 |
|  | HP TPASS_0762 | **B2S401** | No hit found | No hit found | No hit found | | Protein of unknown function DUF115 | Protein of unknown function DUF115 | Cluster 143156  (Polysialic acid transport protein KpsD) | Cluster 4054221 |
|  | HP TPASS_0763 | **B2S402** | No hit found | No hit found | No hit found | | No hit found | TPR family | Cluster 137240  (Serine/threonine protein kinase) | Cluster 3376018  Cluster Name: Treponema |
|  | HP TPASS_0764 | **B2S403** | Response regulator -like domain | HD-domain/PDEase-like | No hit found | | HDc superfamily  (Metal dependent phosphohydrolases with conserved 'HD' motif) | HD domain | Cluster 111108  Metal-dependent phosphohydrolase, HD region | Cluster 4144731  Cluster Name: Metal-dependent phosphohydrolase, HD region, subdomain |
|  | HP TPASS_0766 | **B2S405** | No hit found | No hit found | No hit found | | No hit found | Mito_fiss_reg family  (Mitochondrial fission regulator) | Cluster 121160  (Pyrimidine dimer DNA glycosylase) | Cluster 3692883  Cluster Name: Treponema |
|  | HP TPASS_0771 | **B2S410** | No hit found | PhoU-like | Solute carrier family 34 (sodium phosphate), member 2-related | | Na_Pi_cotrans superfamily  (Na+/Pi-cotransporter) | Na_Pi_cotrans family  (Na+/Pi-cotransporter) | Cluster 111193  (transporter activity) | Cluster 4128774Cluster Name: Sodium-dependent phosphate transmembrane transporter activity |
|  | HP TPASS_0772 | **B2S411** | No hit found | No hit found | No hit found | | No hit found | No hit found | Cluster 120158  (H+-transporting two-sector ATPase, alpha/beta subunit, central region) | Cluster 3969173  Cluster Name: Treponema |
|  | HP TPASS_0776 | **B2S415** | Phosphoribosyltransferase -like domain | PRTase like | Amidophosphoribosyltransferase | | PRTase_typeI superfamily(Phosphoribosyl transferase (PRT)-type I domain) | Pribosyltran family  (Phosphoribosyl transferase domain) | Cluster 149960  (Phosphoribosyltransferase) | Cluster 4092595  Cluster Name: Phosphoribosyltransferase |
|  | HP TPASS_0777 | **B2S416** | No hit found | ZZ domain | No hit found | | Mut7-C RNAse domain | ZZ family  (Zinc finger, ZZ type) | Cluster 113601  (zinc finger protein) | Cluster 314166  Cluster Name: Treponema pallidum |
|  | HP TPASS_0781 | **B2S420** | No hit found | No hit found | No hit found | | No hit found | No hit found | Cluster 104459  (von Willebrand factor, type A) | Cluster 281918  Cluster Name: Treponema pallidum |
|  | HP TPASS_0782 | **B2S421** | Lipoprotein -like domain | Duplicated hybrid motif | No hit found | | No hit found | Peptidase_M23 family | Cluster 139248  (Peptidase M23B) | Cluster 3642644  Cluster Name: Treponema |
|  | HP TPASS_0783 | **B2S422** | Nbas protein -like domain | YVTN repeat-like/Quinoprotein amine dehydrogenase | No hit found | | No hit found | No hit found | Cluster 91922  (G-protein beta WD-40 repeat) | Cluster 2939761  Cluster Name: Treponema |
|  | HP TPASS_0784 | **B2S423** | No hit found | No hit found | No hit found | | No hit found | LptC family  (Lipopolysaccharide-assembly, LptC-related) | Cluster 81448  (Beta-lactamase-like) | Cluster 3752851 |
|  | HP TPASS_0785 | **B2S424** | LPS-assembly protein lptD -like domain | No hit found | No hit found | | OstA superfamily | OstA family | Cluster 137358  (Ankyrin repeat) | Cluster 3732510  Cluster Name: OstA-like protein |
|  | HP TPASS_0787 | **B2S426** | No hit found | No hit found | No hit found | | No hit found | No hit found | Cluster 137639  (Amino acid/polyamine transporter II) | Cluster 4127389  Cluster Name: Treponema |
|  | HP TPASS_0788 | **B2S427** | No hit found | No hit found | No hit found | | No hit found | No hit found | Cluster 150555  (transcriptional regulator) | Cluster 3172058  Cluster Name: Treponema |
|  | HP TPASS_0789 | **B2S428** | Outer-membrane lipoprotein carrier protein -like domain | No hit found | No hit found | | No hit found | GHBP family  (Growth hormone receptor binding) | Cluster 135388 | Cluster 4099909 |
|  | HP TPASS_0791 | **B2S430** | No hit found | GckA/TtuD-like | No hit found | | No hit found | No hit found | Cluster 113378  (Multidrug resistance protein) | Cluster 544534  Cluster Name: Treponema pallidum |
|  | HP TPASS_0793 | **B2S432** | No hit found | No hit found | No hit found | | No hit found | No hit found | Cluster 114660  (Transcriptional regulator) | Cluster 2756117  Cluster Name: Treponema |
|  | HP TPASS_0795 | **B2S434** | No hit found | No hit found | No hit found | | No hit found | No hit found | Cluster 19111 | No hit found |
|  | HP TPASS_0796 | **B2S435** | No hit found | ApbE-like | Thiamine biosynthesis lipoprotein apbe | | ApbE superfamily | ApbE family | Cluster 141895  (Thiamine biosynthesis lipoprotein APBE) | Cluster 4160818  Cluster Name: ApbE-like lipoprotein |
|  | HP TPASS_0799 | **B2S438** | No hit found | No hit found | No hit found | | No hit found | No hit found | Cluster 19114 | Cluster 544535  Cluster Name: Treponema pallidum |
|  | HP TPASS_0802 | **B2S441** | No hit found | No hit found | No hit found | | No hit found | No hit found | Cluster 97331  (Ankyrin repeat) | Cluster 444193  Cluster Name: Treponema pallidum |
|  | HP TPASS_0803 | **B2S442** | 50S ribosomal protein L9 -like domain | DHH phosphoesterases | No hit found | | DHH superfamily  (phosphoesterase) | DHH family | Cluster 79672  (Phosphoesterase, RecJ-like) | Cluster 2801194  Cluster Name: Phosphoesterase, RecJ-like |
|  | HP TPASS_0811 | **B2S450** | No hit found | No hit found | No hit found | | No hit found | No hit found | Cluster 19129 | Cluster 296304  Cluster Name: Treponema pallidum |
|  | HP TPASS_0813 | **B2S451** | No hit found | No hit found | No hit found | | No hit found | No hit found | Cluster 19131 | Cluster 3789012 |
|  | HP TPASS_0815 | **B2S453** | Mycothiol acetyltransferase -like domain | Acyl-CoA N-acyltransferases (Nat) | No hit found | | NAT_SF superfamily  (N-Acyltransferase superfamily) | FR47 family | Cluster 150587  (GCN5-related N-acetyltransferase) | Cluster 4082776  Cluster Name: N-acetyltransferase activity |
|  | HP TPASS_0816 | **B2S454** | No hit found | No hit found | No hit found | | No hit found | No hit found | Cluster 152498  ((Nucleoside deoxyribosyltransferase-I) (EC 2.4.2.6)) | Cluster 3091585  Cluster Name: Treponema |
|  | HP TPASS_0818 | **B2S456** | No hit found | No hit found | No hit found | | No hit found | No hit found | Cluster 157393  (Esterase/lipase/thioesterase) | Cluster 857143  Cluster Name: Treponema pallidum |
|  | HP TPASS_0820 | **B2S458** | Bardet-Biedl syndrome 4 protein -like domain | TPR like | Tetratricopeptide repeat protein, tpr | | TPR superfamily | TPR family | Cluster 136428  (TPR containing protein) | Cluster 3710181  (Tetratricopeptide repeat) |
|  | HP TPASS_0822 | **B2S460** | Lin1012 protein -like domain | Mechanosensitive channel protein MscS (YggB), C-terminal domain | Small-conductance mechanosensitive channel | | MS_channel superfamily  (Mechanosensitive ion channel) | MS_channel family  (Mechanosensitive ion channel) | Cluster 140192  (Mechanosensitive ion channel) | Cluster 4167964  Cluster Name: Mechanosensitive channel protein MscS (YggB), transmembrane region |
|  | HP TPASS_0825 | **B2S463** | No hit found | No hit found | No hit found | | No hit found | No hit found | Cluster 19136 | Cluster 4457122 |
|  | HP TPASS_0826 | **B2S464** | DNA integrity scanning protein DisA -like domain | YojJ like | No hit found | | DisA_N superfamily  (DisA bacterial checkpoint controller nucleotide-binding) | DisA_N family  (DisA bacterial checkpoint controller nucleotide-binding) | Cluster 151366  (DNA binding) | Cluster 4034503  (DNA integrity scanning protein, DisA, N-termina) |
|  | HP TPASS_0827 | **B2S465** | No hit found | No hit found | No hit found | | YbbR superfamily | YbbR family | Cluster 47270 | Cluster 3975889 |
|  | HP TPASS_0829 | **B2S467** | No hit found | EF-G C-terminal domain-like | No hit found | | CAP_ED superfamily  (effector domain of the CAP family of transcription factors) | EFG_C  Family  (Elongation factor G C-terminus) | Cluster 78264  (TPR) | Cluster 3182909 |
|  | HP TPASS_0832 | **B2S470** | No hit found | No hit found | No hit found | | Germane superfamily  (Sporulation and spore germination) | Germane family  (Sporulation and spore germination) | Cluster 144215  (hydroxyethylthiazole kinase (EC 2.7.1.50)) | Cluster 3790485  (GerMN domain) |
|  | HP TPASS_0833 | **B2S471** | No hit found | No hit found | No hit found | | No hit found | No hit found | Cluster 19139 | Cluster 3879962  Cluster Name: Treponema |
|  | HP TPASS_0836 | **B2S474** | No hit found | No hit found | No hit found | | No hit found | No hit found | Cluster 102281  (Ornithine carbamoyltransferase (EC 2.1.3.3)) | Cluster 3997944 |
|  | HP TPASS_0839 | **B2S477** | No hit found | No hit found | No hit found | | No hit found | No hit found | Cluster 95486  (Glycosyltransferase) | Cluster 4141242 |
|  | HP TPASS_0840 | **B2S478** | Proline/betaine transporter -like domain | MFS general substrate transporter | Major facilitator superfamily domain-containing protein-related | | MFS superfamily  (Major Facilitator Superfamily) | MFS_1 family  (Major Facilitator Superfamily) | Cluster 145051  (Major Facilitator Superfamily) | Cluster 3786193  Cluster Name: Leucine-rich Repeat Variant  (Major facilitator superfamily, general substrate transporter) |
|  | HP TPASS_0845 | **B2S483** | No hit found | No hit found | No hit found | | Mur_ligase superfamily | No hit found | Cluster 133239 | Cluster 3880203 |
|  | HP TPASS_0846 | **B2S484** | No hit found | Cell division protein ZapA-like | No hit found | | ZapA superfamily  (Cell division protein ZapA) | ZapA family  (Cell division protein ZapA) | Cluster 19150 | Cluster 3714904  Cluster Name: Cell division protein ZapA-like |
|  | HP TPASS_0847 | **B2S485** | No hit found | N-terminal coiled coil domain from apc | No hit found | | No hit found | Tropomyosin_1 family | Cluster 136995  (Methyl-accepting chemotaxis protein) | Cluster 3647966  Cluster Name: Treponema |
|  | HP TPASS_0851 | **B2S489** | UDP-3-O-acylglucosamine N-acyltransferase -like domain | Trimeric LpxA-like enzymes | No hit found | | LpxD superfamily  (UDP-3-O-[3-hydroxymyristoyl] glucosamine N-acyltransferase) | CART family  (Cocaine and amphetamine regulated transcript protein) | Cluster 141273  (translation initiation factor) | Cluster 4324261  Cluster Name: Trimeric LpxA-like |
|  | HP TPASS_0854 | **B2S492** | Neuraminidase -like domain | Sialidases (neuraminidases) | Cytoskeletal protein | | PP2Cc superfamily  (Serine/threonine phosphatases) | SpoIIE  Stage II sporulation protein E | Cluster 94036  (Histidine kinase, HAMP region) | Cluster 4175091  Cluster Name: Sporulation stage II, protein E C-terminal |
|  | HP TPASS_0855 | **B2S493** | No hit found | TPR-like | No hit found | | No hit found | No hit found | Cluster 110345  (Zinc finger domain) | Cluster 2719694  Cluster Name: Treponema |
|  | HP TPASS_0856 | **B2S494** | No hit found | No hit found | No hit found | | Toluene_X superfamily | No hit found | Cluster 83194  (Adenosine deaminase) | Cluster 3609927 |
|  | HP TPASS_0857 | **B2S495** | No hit found | No hit found | No hit found | | Bac_rhodopsin  Superfamily  (Bacteriorhodopsin-like protein) | No hit found | No hit found | Cluster 281933  Cluster Name: Treponema pallidum |
|  | HP TPASS_0858 | **B2S496** | No hit found | No hit found | No hit found | | Toluene_X superfamily | No hit found | Cluster 151180 | Cluster 3609927 |
|  | HP TPASS_0859 | **B2S497** | No hit found | No hit found | No hit found | | Toluene_X superfamily | No hit found | Cluster 151180 | Cluster 3609927 |
|  | HP TPASS_0860 | **B2S498** | No hit found | TPR-like | No hit found | | Toluene_X superfamily | No hit found | Cluster 140031  (TPR like) | Cluster 3609927 |
|  | HP TPASS_0864 | **B2S4A2** | Lipoprotein -like domain | LysM domain | Peptidase-related | | Peptidase M23 superfamily | Peptidase M23 family | Cluster 138458  (Peptidase M23) | Cluster 4184571  Cluster Name: Peptidase M23B |
|  | HP TPASS_0865 | **B2S4A3** | No hit found | TPR like | No hit found | | Toluene_X superfamily | No hit found | Cluster 140031  (TPR containing protein) | Cluster 3609927 |
|  | HP TPASS_0867 | **B2S4A4** | No hit found | No hit found | No hit found | | No hit found | No hit found | Cluster 19160 | Cluster 802456  Cluster Name: Treponema pallidum |
|  | HP TPASS_0869 | **B2S4A6** | No hit found | Retrovirus zinc finger-like domain | No hit found | | No hit found | No hit found | Cluster 19161 | Cluster 857148  Cluster Name: Treponema pallidum |
|  | HP TPASS_0871 | **B2S4A8** | No hit found | No hit found | No hit found | | No hit found | No hit found | Cluster 19163 | Cluster 4489429 |
|  | HP TPASS_0873 | **B2S4B0** | No hit found | No hit found | No hit found | | No hit found | No hit found | Cluster 92552  (alcohol dehydrogenase) | Cluster 3354795  Cluster Name: Treponema |
|  | HP TPASS_0874 | **B2S4B1** | No hit found | No hit found | No hit found | | No hit found | No hit found | Cluster 122871 | Cluster 3946937 |
|  | HP TPASS_0875 | **B2S4B2** | Replication-associated protein -like domain | P-loop containing nucleoside triphosphate hydrolase | No hit found | | AAA superfamily  (ATPases) | No hit found | Cluster 149840  (ATP binding protein) | Cluster 4166012  (ATPase) |
|  | HP TPASS_0876 | **B2S4B3** | Acetate kinase -like domain | Actin-like ATPase domain | O-sialoglycoprotein endopeptidase | | BadF/BadG/BcrA/BcrD ATPase family | Peptidase_M22 family  (Glycoprotease family) | Cluster 143405  (Peptidase M22, glycoprotease) | Cluster 4133040  Cluster Name: Peptidase M22, glycoprotease |
|  | HP TPASS_0877 | **B2S4B4** | Response regulator -like domain | HD-domain/PDEase-like | Diguanylate cyclase yedq-related | | HDc superfamily  (Metal dependent phosphohydrolases with conserved 'HD' motif) | HD domain | Cluster 109058  Metal-dependent phosphohydrolase, HD region | Cluster 4158669  Cluster Name: Metal-dependent phosphohydrolase, HD region, subdomain |
|  | HP TPASS_0878 | **B2S4B5** | No hit found | Glycoside hydrolase/deacetylase | No hit found | | Thiredoxin like superfamily | No hit found | Cluster 19167 | Cluster 3589517  Cluster Name: Treponema |
|  | HP TPASS_0879 | **B2S4B6** | No hit found | No hit found | No hit found | | Thiredoxin like superfamily | ABC_transp_aux family  (ABC-type uncharacterized transport system) | Cluster 133368  (Gliding motility protein GldG) | Cluster 2863986  Cluster Name: Treponema |
|  | HP TPASS_0882 | **B2S4B9** | Deoxyhypusine hydroxylase -like domain | ARM repeat | No hit found | | No hit found | HEAT_2 family | Cluster 122880  (ARM repeat fold) | Cluster 4136200  (ARM repeat) |
|  | HP TPASS_0883 | **B2S4C0** | No hit found | No hit found | No hit found | | YjgP_YjgQ superfamily  (permease YjgP/YjgQ) | YjgP_YjgQ family  (permease YjgP/YjgQ) | Cluster 126096  (permease) | Cluster 3874754  Cluster Name: Predicted permease YjgP/YjgQ |
|  | HP TPASS_0884 | **B2S4C1** | No hit found | No hit found | No hit found | | YjgP_YjgQ superfamily  (permease YjgP/YjgQ) | YjgP_YjgQ superfamily  (permease YjgP/YjgQ) | Cluster 141589  (sugar porter activity) | Cluster 3842831  Cluster Name: Predicted permease YjgP/YjgQ |
|  | HP TPASS_0893 | **B2S4D0** | No hit found | YhbC-like, N-terminal domain | No hit found | | Sm_like superfamily  () | No hit found | Cluster 78159 | Cluster 3780149 |
|  | HP TPASS_0894 | **B2S4D1** | Limkain-b1 -like domain | Nucleic acid-binding proteins | No hit found | | OHA superfamily  (OST-HTH Associated domain) | NYN domain | Cluster 129815  (Nucleic acid-binding OB-fold) | Cluster 4153712 |
|  | HP TPASS_0895 | **B2S4D2** | No hit found | TT1751-like | No hit found | | Beta_lactamase superfamily | Domain of unknown function DUF302 | Cluster 150848  (Formyl transferase, N-terminal) | Cluster 4140589  (TATA-Binding Protein) |
|  | HP TPASS_0896 | **B2S4D3** | No hit found | No hit found | No hit found | | No hit found | No hit found | Cluster 135229  (Peptidase M14, carboxypeptidase A) | Cluster 351698  Cluster Name: Treponema pallidum |
|  | HP TPASS_0899 | **B2S4D6** | No hit found | Restriction endonuclease-like | No hit found | | CRISPR/Cas system-associated protein Cas4 | PDDEXK_1  (PD-(D/E)XK nuclease superfamily) | Cluster 142182  (chemotaxis protein) | Cluster 2072226  Cluster Name: Treponema pallidum |
|  | HP TPASS_0900 | **B2S4D7** | No hit found | P-loop containing nucleoside triphosphate hydrolase | No hit found | | CRISPR/Cas system-associated protein Cas4 | PDDEXK_1  (PD-(D/E)XK nuclease superfamily) | Cluster 129487  (ATP-dependent nuclease) | Cluster 2651531  Cluster Name: Treponema |
|  | HP TPASS_0901 | **B2S4D8** | No hit found | No hit found | Multidrug resistance protein | | MATE_like superfamily  (Multidrug and toxic compound extrusion family) | MatE family | Cluster 138315  (Multi antimicrobial extrusion protein MatE) | Cluster 4047472  Cluster Name: Multi antimicrobial extrusion protein MatE |
|  | HP TPASS_0904 | **B2S4E1** | No hit found | No hit found | No hit found | | No hit found | No hit found | Cluster 19186 | Cluster 776728  Cluster Name: Treponema pallidum |
|  | HP TPASS_0906 | **B2S4E3** | No hit found | Prokaryotic type KH domain (KH-domain type II) | No hit found | | KH-II superfamily  (K homology RNA-binding domain, type II) | KH_4 family  (KH domain) | Cluster 151660  (KH domain protein) | Cluster 4074170  Cluster Name: Prokaryotic type KH domain (KH-domain type II) |
|  | HP TPASS_0907 | **B2S4E4** | No hit found | Translation proteins  (RimM N-terminal domain-like) | No hit found | | RimM superfamily  (RimM N-terminal domain) | RimM family  (RimM N-terminal domain) | Cluster 113239 | Cluster 3849471  Cluster Name: Ribosomal small subunit biogenesis |
|  | HP TPASS_0910 | **B2S4E7** | No hit found | No hit found | No hit found | | No hit found | No hit found | Cluster 126699  (Cytochrome P450 aromatase) | Cluster 3716460  Cluster Name: Treponema |
|  | HP TPASS_0911 | **B2S4E8** | Flagellar biosynthetic protein flhB -like domain | EscU C-terminal domain-like | Flagellar biosynthetic protein flhb | | Bac_export_2  (FlhB HrpN YscU SpaS Family) | Bac_export_2  (FlhB HrpN YscU SpaS Family) | Cluster 155073  (FlhB (Flagellar biosynthetic protein)) | Cluster 4066018  Cluster Name: Type III secretion exporter |
|  | HP TPASS_0912 | **B2S4E9** | Response regulator -like domain | HD-domain/PDEase-like | Diguanylate cyclase yedq-related | | HDc superfamily | HD domain | Cluster 137731  (Metal-dependent phosphohydrolase, HD subdomain) | Cluster 4144731  Cluster Name: Metal-dependent phosphohydrolase, HD region, subdomain |
|  | HP TPASS_0913 | **B2S4F0** | UPF0102 protein MCA0184 -like domain | Restriction endonuclease-like | No hit found | | RNAse_H like superfamily | No hit found | Cluster 155972  (Endonuclease (EC 3.1.-.-)) | Cluster 4170667  (Restriction endonuclease, type II-like, core) |
|  | HP TPASS_0914 | **B2S4F1** | No hit found | No hit found | No hit found | | No hit found | No hit found | Cluster 122878 | Cluster 4070729 |
|  | HP TPASS_0915 | **B2S4F2** | Bardet-Biedl syndrome 4 protein -like domain | TPR-like | Tetratricopeptide repeat protein, tpr | | TPR superfamily | TPR family | Cluster 128428  (TPR) | Cluster 3406828  Cluster Name: DNA restriction-modification system  (PTR) |
|  | HP TPASS_0916 | **B2S4F3** | No hit found | No hit found | No hit found | | No hit found | No hit found | Cluster 19190 | No hit found |
|  | HP TPASS_0918 | **B2S4F5** | No hit found | No hit found | Phytol kinase 1, chloroplastic | | SEC59 superfamily  (Dolichol kinase [Lipid metabolism]) | No hit found | Cluster 59238  (Integral membrane protein) | Cluster 3996349  Cluster Name: Phosphatidate cytidylyltransferase |
|  | HP TPASS_0920 | **B2S4F7** | No hit found | TPR like | Tetratricopeptide repeat protein, tpr | | TPR superfamily | TPR family | Cluster 78741  (TPR like) | Cluster 2588096  Cluster Name: Treponema  (TPR) |
|  | HP TPASS_0922 | **B2S4F9** | No hit found | T-antigen specific domain-like | No hit found | | No hit found | No hit found | Cluster 19192 | Cluster 444289  Cluster Name: Treponema pallidum |
|  | HP TPASS_0923 | **B2S4G0** | No hit found | Carboxypeptidase regulatory domain-like | No hit found | | Peptidase_M14NE-CP-C_like Superfamily | PEGA domain | Cluster 137945  (Peptidase M14, carboxypeptidase A) | Cluster 2867922  Cluster Name: Treponema |
|  | HP TPASS_0927 | **B2S4G4** | No hit found | No hit found | No hit found | | No hit found | No hit found | Cluster 46421  (DNA binding protein) | Cluster 3762540  Cluster Name: Treponema |
|  | HP TPASS_0928 | **B2S4G5** | No hit found | Triger factor/SurA peptide-binding domain-like | No hit found | | No hit found | No hit found | Cluster 126797  (GTP-binding signal recognition particle (SRP54) G-domain) | Cluster 4042096 |
|  | HP TPASS_0929 | **B2S4G6** | No hit found | FKBP immunophilin/proline isomerase | No hit found | | No hit found | No hit found | Cluster 86450  (TPR) | Cluster 3181546  Cluster Name: Treponema |
|  | HP TPASS_0930 | **B2S4G7** | No hit found | No hit found | No hit found | | TPPK_C superfamily  (Thiamine pyrophosphokinase C terminal) | Protein of unknown function DUF115 | Cluster 146028  (Phosphate transporter) | Cluster 2796689  Cluster Name: Protein of unknown function DUF115 |
|  | HP TPASS_0931 | **B2S4G8** | Ribosomal protein S12 methylthiotransferase RimO -like domain | Six-hairpin glycosidases | Mannosyl-oligosaccharide glucosidase | | Trehalase superfamily | Trehalase family | Cluster 153172  (Glycoside transferase, six-hairpin) | Cluster 3617250  Cluster Name: Alpha,alpha-trehalase activity |
|  | HP TPASS_0932 | **B2S4G9** | No hit found | No hit found | No hit found | | No hit found | No hit found | Cluster 19202 | No hit found |
|  | HP TPASS_0937 | **B2S4H4** | No hit found | Metallo-dependent phosphatases | T: VACUOLAR SORTING PROTEIN VPS29 | | MPP superfamily  (metallophosphatase superfamily) | Metallophos_2  (Calcineurin-like phosphoesterase superfamily domain) | Cluster 147763  (Metallo-phosphoesterase) | Cluster 4122123  (Metallophosphoesterase) |
|  | HP TPASS_0938 | **B2S4H5** | No hit found | No hit found | No hit found | | No hit found | No hit found | Cluster 125800  (1-aminocyclopropane-1-carboxylate oxidase-like protein) | Cluster 3606702  Cluster Name: Treponema |
|  | HP TPASS_0940 | **B2S4H7** | No hit found | No hit found | No hit found | | No hit found | No hit found | Cluster 19206 | Cluster 4578050 |
|  | HP TPASS_0941 | **B2S4H8** | No hit found | No hit found | No hit found | | No hit found | No hit found | Cluster 148058  (Galactosyl transferase) | Cluster 422878  Cluster Name: Treponema pallidum |
|  | HP TPASS_0942 | **B2S4H9** | No hit found | FlgN-like | No hit found | | No hit found | FlgN protein | Cluster 147025  (uroporphyrin-III C-methyltransferase (EC 2.1.1.107)) | Cluster 3569301  Cluster Name: Treponema |
|  | HP TPASS_0944 | **B2S4I1** | Ctr9 protein -like domain | TPR-like | No hit found | | No hit found | TPR_11 family | Cluster 115669  (TPR) | Cluster 3519702  (TPR) |
|  | HP TPASS_0950 | **B2S4I7** | No hit found | No hit found | No hit found | | No hit found | No hit found | Cluster 19214 | Cluster 4462064 |
|  | HP TPASS_0954 | **B2S4J1** | Slr2048 protein -like domain | TPR like | Tetratricopeptide repeat protein, tpr | | TPR superfamily | TPR family | Cluster 136899  (TPR containing protein) | Cluster 4064497  Cluster Name: TPR repeat |
|  | HP TPASS_0955 | **B2S4J2** | No hit found | No hit found | No hit found | | No hit found | No hit found | Cluster 89428  (mitochondrial endonuclease) | Cluster 564578  Cluster Name: Treponema pallidum |
|  | HP TPASS_0956 | **B2S4J3** | No hit found | No hit found | No hit found | | No hit found | No hit found | Cluster 93196  (Peptidase C48, SUMO/Sentrin/Ubl1) | Cluster 3743757 |
|  | HP TPASS_0959 | **B2S4J6** | No hit found | No hit found | No hit found | | Rod-binding protein | Rod-binding protein | Cluster 130197  (Flagellum-specific muramidase) | Cluster 4033538 |
|  | HP TPASS_0962 | **B2S4J9** | No hit found | No hit found | Membrane component of transporter-related | | MacB_PCD superfamily  (MacB-like periplasmic core domain) | MacB_PCD family  (MacB-like periplasmic core domain) | Cluster 139161  (permease) | Cluster 4159917  (permease) |
|  | HP TPASS_0963 | **B2S4K0** | No hit found | 2-methylcitrate dehydratase PrpD | Membrane component of transporter-related | | MacB_PCD superfamily  (MacB-like periplasmic core domain) | MacB_PCD family  (MacB-like periplasmic core domain) | Cluster 137482  (ABC transporter, permease protein) | Cluster 4159917  (permease) |
|  | HP TPASS_0966 | **B2S4K3** | No hit found | Mitotic arrest deficient-like 1, Mad1 | No hit found | | FH2 superfamily  (formin homology 2 domain) | No hit found | Cluster 143347  ((N-glycosyltransferase) | Cluster 3967731  Cluster Name: Treponema pallidum |
|  | HP TPASS_0967 | **B2S4K4** | No hit found | No hit found | No hit found | | No hit found | No hit found | Cluster 156223 | Cluster 3967731  Cluster Name: Treponema pallidum |
|  | HP TPASS_0968 | **B2S4K5** | No hit found | No hit found | No hit found | | No hit found | CPSF100_C family  (Cleavage and polyadenylation factor 2 C-terminal) | Cluster 112189  (Coiled coil protein) | Cluster 3967731  Cluster Name: Treponema pallidum |
|  | HP TPASS_0969 | **B2S4K6** | No hit found | Outer membrane efflux proteins (OEP) | No hit found | | Biotin_lipoyl_2 superfamily | No hit found | Cluster 141303  (RNA-binding region RNP-1) | Cluster 3967731  Cluster Name: Treponema pallidum |
|  | HP TPASS_0970 | **B2S4K7** | No hit found | No hit found | No hit found | | No hit found | No hit found | Cluster 19227 | No hit found |
|  | HP TPASS_0972 | **B2S4K9** | No hit found | No hit found | No hit found | | FTR1  (Iron permease FTR1 family) | FTR1 family  (Iron permease FTR1 family) | Cluster 105152  (PAS-associated, C-terminal) | Cluster 4151064  Cluster Name: Iron permease FTR1 |
|  | HP TPASS_0974 | **B2S4L1** | No hit found | Anti-sigma factor FlgM | No hit found | | No hit found | Val_tRNA-synt_C family  (Valyl tRNA synthetase tRNA binding arm) | Cluster 98504  (glycosyl transferase) | Cluster 3758437  Cluster Name: Anti-sigma-28 factor, FlgM |
|  | HP TPASS_0975 | **B2S4L2** | No hit found | Ribosomal RNA small subunit methyltransferase I -like domain | Ribosomal rna small subunit methyltransferase i | | TP_methylase family  (Tetrapyrrole (Corrin/Porphyrin) Methylases) | TP_methylase family  (Tetrapyrrole (Corrin/Porphyrin) Methylases) | Cluster 144435  (Tetrapyrrole methylase family protein) | Cluster 4162143  (Tetrapyrrole methylase) |
|  | HP TPASS_0976 | **B2S4L3** | No hit found | No hit found | No hit found | | No hit found | No hit found | Cluster 145834 (Transcriptional regulator) | Cluster 3833310  Cluster Name: Treponema |
|  | HP TPASS_0977 | **B2S4L4** | No hit found | NIF3 (NGG1p interacting factor 3)-like | Ngg1 interacting factor 3 | | NIF3 superfamily  (NIF3 (NGG1p interacting factor 3)) | NIF3 family  (NIF3 (NGG1p interacting factor 3)) | Cluster 143125  (ribosomal protein) | Cluster 3959512  Cluster Name: NIF3 (NGG1p interacting factor 3)-like |
|  | HP TPASS_0979 | **B2S4L6** | Deoxyribonuclease TatD -like domain | Metallo-dependent hydrolases | Tatd family deoxyribonuclease | | metallo-dependent_hydrolases Superfamily | TatD_DNase family | Cluster 149137  (TatD-related deoxyribonuclease) | Cluster 4150200  Cluster Name: Deoxyribonuclease, TatD Mg-dependent |
|  | HP TPASS_0983 | **B2S4M0** | No hit found | No hit found | No hit found | | No hit found | No hit found | Cluster 146401  (uridylyltransferase (EC 2.7.7.59)) | Cluster 3249828  Cluster Name: Treponema |
|  | HP TPASS_0986 | **B2S4M3** | No hit found | Multidrug resistance efflux transporter EmrE | Acyl-malonyl condensing enzyme-related | | EamA superfamily | EamA family  (EamA-like transporter family) | Cluster 143735  (Transporter, DME family) | Cluster 4176824 |
|  | HP TPASS_0987 | **B2S4M4** | No hit found | No hit found | No hit found | | No hit found | No hit found | Cluster 19235 | Cluster 639753  Cluster Name: Treponema pallidum |
|  | HP TPASS_0988 | **B2S4M5** | No hit found | No hit found | No hit found | | MarC superfamily | No hit found | Cluster 152608  (Multiple antibiotic resistance protein marC) | Cluster 4144735  Cluster Name: Multiple antibiotic resistance (MarC)-related |
|  | HP TPASS_0990 | **B2S4M7** | Bacteriophage N4 adsorption protein A -like domain | TPR like | Tetratricopeptide repeat protein, tpr | | TPR superfamily | TPR family | Cluster 116421  (TPR like) | Cluster 3505946  (TPR containing) |
|  | HP TPASS_0992 | **B2S4M9** | No hit found | No hit found | No hit found | | No hit found | No hit found | Cluster 122875 | Cluster 3948458 |
|  | HP TPASS_0994 | **B2S4N1** | Deoxyribonuclease TatD -like domain | Metallo-dependent hydrolases | Cell death-related nuclease 2 | | metallo-dependent_hydrolases Superfamily | TatD_DNase family | Cluster 149137  (TatD-related deoxyribonuclease) | Cluster 4150200  Cluster Name: Deoxyribonuclease, TatD Mg-dependent |
|  | HP TPASS_0996 | **B2S4N3** | No hit found | No hit found | No hit found | | No hit found | No hit found | Cluster 126601  (Nitrogen regulation protein ntrY (EC 2.7.3.-)) | Cluster 3978652  Cluster Name: Cyclic nucleotide-binding-like |
|  | HP TPASS_1000 | **B2S4N7** | No hit found | L,D-transpeptidase catalytic domain-like | No hit found | | No hit found | YkuD family  (L,D-transpeptidase catalytic domain) | Cluster 147873  (Glutaryl-7-ACA acylase) | Cluster 639755  Cluster Name: Treponema pallidum |
|  | HP TPASS_1001 | **B2S4N8** | No hit found | No hit found | No hit found | | No hit found | No hit found | Cluster 112259  (Zn-finger-like, PHD finger) | Cluster 3667733  Cluster Name: Treponema |
|  | HP TPASS_1002 | **B2S4N9** | No hit found | DPP6 N-terminal domain-like | No hit found | | TroA like superfamily  (Helical backbone metal receptor) | No hit found | Cluster 129998  (Transcriptional regulator) | Cluster 3837429 |
|  | HP TPASS_1003 | **B2S4P0** | No hit found | No hit found | No hit found | | No hit found | No hit found | Cluster 133228 | Cluster 3732121  Cluster Name: Treponema |
|  | HP TPASS_1014 | **B2S4Q1** | Uncharacterized protein TP_1014 -like domain | No hit found | No hit found | | No hit found | No hit found | Cluster 141289  (Histidine kinase) | Cluster 3072310  (TPR containing) |
|  | HP TPASS_1018 | **B2S4Q5** | Ribonuclease Y -like domain | HD-domain/PDEase-like | No hit found | | HDc superfamily | HD domain | Cluster 143890  (Metal-dependent phosphohydrolase, HD region) | Cluster 4148288  Cluster Name: 2,3-cyclic-nucleotide 2-phosphodiesterase |
|  | HP TPASS_1029 | **B2S4R6** | No hit found | RNA binding domain | No hit found | | RRM_SF superfamily  (RNA recognition motif (RRM) superfamily) | DbpA family  (RNA binding domain) | Cluster 83163  (RNA binding domain) | Cluster 4094169  Cluster Name: DbpA, RNA-binding |
|  | HP TPASS_1030 | **B2S4R7** | No hit found | No hit found | No hit found | | No hit found | Herpes_BLLF1 family  (Herpes virus major outer envelope glycoprotein) | Cluster 19259 | Cluster 373540  Cluster Name: Treponema pallidum |
|  | HP TPASS_1032 | **B2S4R9** | Transcription antitermination protein nusG -like domain | N-utilization substance G protein NusG, insert domain | No hit found | | DUF1312 superfamilu  (N-Utilization Substance G (NusG) N terminal (NGN)) | DUF1312 familyu | Cluster 156229  (TonB-dependent receptor protein) | Cluster 4066877 |
|  | HP TPASS_1033 | **B2S4S0** | Lysophospholipase -like domain | FabD/lysophospholipase-like | Neuropathy target esterase/swiss cheese(d.melanogaster) | | Patatin_and_cPLA2 Superfamily  (Patatins and Phospholipases) | Patatin family  (Patatin-like phospholipase) | Cluster 140485  (Patatin) | Cluster 4157051  Cluster Name: Patatin |
|  | HP TPASS_1034 | **B2S4S1** | No hit found | No hit found | Inner membrane protein yrbg | | Na_Ca_ex superfamily  (Sodium/calcium exchanger protein) | Na_Ca_ex family  (Sodium/calcium exchanger protein) | Cluster 143288  (K+-dependent Na+/Ca+ exchanger related-protein) | Cluster 4105215  Cluster Name: K+-dependent Na+/Ca+ exchanger-like |
