# Supplementary material for: Identification of Functional Candidates amongst Hypothetical Proteins of Treponema pallidum ssp. pallidum
Source: PLoS One. 2015 Apr 20;10(4):e0124177. doi: 10.1371/journal.pone.0124177 (PMC4403809; doi:10.1371/journal.pone.0124177)
Supplement: S6 Table — (DOC) [file pone.0124177.s006.doc]

| **Table S6: List of annotated function of 100 proteins with known function from *T. pallidum* *ssp. pallidum* using BLASTp, HMMER, SMART and INTERPROSCAN for ROC analysis.** | | | | | | | |
| --- | --- | --- | --- | --- | --- | --- | --- |
| **S. No.** | **Accession number** | **UNIPROT ID** | **Protein Name** | **Blast** | **HMMER** | **SMART** | **INTERPROSCAN** |
|  | [YP_001933009.1](http://www.ncbi.nlm.nih.gov/protein/189025237) | B2S1V1 | chromosomal replication initiation protein | chromosomal replication initiator protein DnaA  1(5) | chromosomal replication initiator protein DnaA  1(5) | Chromosomal replication initiator protein dnaA  1(5) | Chromosomal replication initiator protein DnaA  1(5) |
|  | [YP_001933010.1](http://www.ncbi.nlm.nih.gov/protein/189025238) | **B2S1V2** | DNA-directed DNA polymerase beta subunit | DNA-directed DNA polymerase III subunit beta  1(5) | DNA polymerase III, beta subunit  1(5) | DNA polymerase III subunit beta  1(5) | DNA polymerase III beta subunit  1(5) |
|  | [YP_001933011.1](http://www.ncbi.nlm.nih.gov/protein/189025239) | **B2S1V3** | recombination protein RecF | DNA replication and repair protein RecF  1(5) | DNA replication and repair protein RecF  1(5) | DNA replication and repair protein recF  1(5) | DNA replication and repair protein RecF  1(5) |
|  | [YP_001933013.1](http://www.ncbi.nlm.nih.gov/protein/189025241) | B2S1V5 | DNA topoisomerase (ATP-hydrolyzing) subunit A  (DNA gyrase) | DNA gyrase, subunit A  1(5) | DNA gyrase/topoisomerase IV, subunit A  1(5) | DNA gyrase subunit A  1(5) | DNA gyrase/topoisomerase IV, subunit A  1(5) |
|  | [YP_001933021.1](http://www.ncbi.nlm.nih.gov/protein/189025249) | **B2S1W3** | phenylalanyl-tRNA synthetase subunit beta | phenylalanyl-tRNA synthetase subunit beta  1(5) | Phenylalanyl-trna Synthetase  1(4) | Phenylalanyl-tRNA synthetase beta chain  1(5) | Phenylalanyl-tRNA synthetase, class IIc, beta subunit  1(5) |
|  | [YP_001933022.1](http://www.ncbi.nlm.nih.gov/protein/189025250) | **B2S1W4** | ATP-dependent protease LA | ATP-dependent protease LA  1(5) | ATP-dependent protease Lon (La), catalytic domain  1(5) | ATP-dependent protease LA  1(5) | Lon protease  1(4) |
|  | [YP_001933024.1](http://www.ncbi.nlm.nih.gov/protein/189025252) | **B2S1W6** | transcription elongation factor | transcription elongation factor  1(5) | Tetratricopeptide repeat  1(1) | Transcription elongation factor  1(5) | Tetratricopeptide-like helical  1(1) |
|  | [YP_001933025.1](http://www.ncbi.nlm.nih.gov/protein/189025253) | **B2S1W7** | transcription elongation factor GreA, partial | transcription elongation factor GreA  1(5) | Transcription elongation factor, GreA/GreB, C-term  1(5) | Transcription elongation factor greA  1(5) | Transcription elongation factor GreA  1(5) |
|  | [YP_001933029.1](http://www.ncbi.nlm.nih.gov/protein/189025257) | **B2S1X1** | sodium- and chloride- dependent transporter | sodium- and chloride- dependent transporter  1(5) | Sodium:neurotransmitter symporter  1(4) | Sodium:neurotransmitter symporter  1(4) | Sodium:neurotransmitter symporter  1(4) |
|  | [YP_001933032.1](http://www.ncbi.nlm.nih.gov/protein/189025260) | **B2S1X4** | flagellar motor switch protein | flagellar motor switch protein FliG  1(5) | FliG N-terminal domain  1(3) | Flagellar motor switch protein  1(5) | Flagellar motor switch protein FliG  1(5) |
|  | [YP_001933033.1](http://www.ncbi.nlm.nih.gov/protein/189025261) | **B2S1X5** | hemolysin | Hemolysin  1(5) | Transporter associated domain  1(3) | Hemolysin  1(5) | Transporter-associated domain  1(3) |
|  | [YP_001933034.1](http://www.ncbi.nlm.nih.gov/protein/189025262) | **B2S1X6** | hemolysin | Hemolysin  1(5) | Transporter associated domain  1(3) | Hemolysin  1(5) | Transporter-associated domain  1(3) |
|  | [YP_001933035.1](http://www.ncbi.nlm.nih.gov/protein/189025263) | **B2S1X7** | UDP-N-acetylglucosamine 1-carboxyvinyltransferase | UDP-N-acetylglucosamine 1-carboxyvinyltransferase  1(5) | UDP-N-acetylglucosamine 1-carboxyvinyltransferase  1(5) | UDP-N-acetylglucosamine 1-carboxyvinyltransferase  1(5) | UDP-N-acetylglucosamine 1-carboxyvinyltransferase  1(5) |
|  | [YP_001933036.1](http://www.ncbi.nlm.nih.gov/protein/189025264) | **B2S1X8** | chaperonin GroEL | chaperonin GroEL  1(5) | chaperonin GroL  1(5) | 60 kDa chaperonin  1(4) | GroEL: chaperonin GroL  1(5) |
|  | [YP_001933038.1](http://www.ncbi.nlm.nih.gov/protein/189025266) | **B2S1Y0** | 16S ribosomal RNA methyltransferase RsmE | 16S ribosomal RNA methyltransferase RsmE  1(5) | RNA methyltransferase  1(4) | Ribosomal RNA small subunit methyltransferase E  1(4) | Ribosomal RNA small subunit methyltransferase E  1(4) |
|  | [YP_001933040.1](http://www.ncbi.nlm.nih.gov/protein/189025268) | **B2S1Y2** | ABC transporter, periplasmic binding protein | ABC transporter, periplasmic binding protein  1(5) | Periplasmic solute binding protein family  1(4) | Uncharacterized periplasmic metal-binding protein TP_0034  1(3) | Periplasmic solute binding protein family  1(4) |
|  | [YP_001933041.1](http://www.ncbi.nlm.nih.gov/protein/189025269) | **B2S1Y3** | ABC transporter, ATP-binding protein | ABC transporter, ATP-binding protein  1(5) | ABC transporter  1(4) | metal transport system ATP-binding protein  1(5) | ATP-binding cassette, ABC transporter-type domain  1(5) |
|  | [YP_001933042.1](http://www.ncbi.nlm.nih.gov/protein/189025270) | **B2S1Y4** | ABC transporter, permease protein | ABC transporter, permease protein  1(5) | ABC 3  transport family  1(4) | metal transport system ATP-binding protein  1(5) | ABC 3  transport family  1(4) |
|  | [YP_001933043.1](http://www.ncbi.nlm.nih.gov/protein/189025271) | **B2S1Y5** | D-specific D-2-hydroxyacid dehydrogenase | D-specific D-2-hydroxyacid dehydrogenase  1(5) | D-isomer specific 2-hydroxyacid dehydrogenase  1(5) | D-lactate dehydrogenase  1(4) | D-isomer specific 2-hydroxyacid dehydrogenase  1(5) |
|  | [YP_001933044.1](http://www.ncbi.nlm.nih.gov/protein/189025272) | **B2S1Y6** | regulatory protein PfoS/R | regulatory protein (pfoS/R)  1(5) | Phosphotransferase system, EIIC  1(3) | Regulatory protein PfoS/R  1(5) | Phosphotransferase system, EIIC  1(3) |
|  | [YP_001933046.1](http://www.ncbi.nlm.nih.gov/protein/189025274) | **B2S1Y8** | methyl-accepting chemotaxis protein | methyl-accepting chemotaxis protein  1(5) | Methyl-accepting chemotaxis protein (MCP) signalling domain  1(5) | Methyl-accepting chemotaxis protein  1(5) | Methyl-accepting chemotaxis protein (MCP) signalling domain  1(5) |
|  | [YP_001933049.1](http://www.ncbi.nlm.nih.gov/protein/189025277) | **B2S1Z1** | soluble lytic transglycosylase | soluble lytic transglycosylase  1(5) | Transglycosylase SLT domain  1(4) | soluble lytic transglycosylase  1(5) | Lytic transglycosylase-like SLT domain  1(4) |
|  | [YP_001933050.1](http://www.ncbi.nlm.nih.gov/protein/189025278) | **B2S1Z2** | tRNA uridine 5-carboxymethylaminomethyl modification enzyme GidA | tRNA uridine 5-carboxymethylaminomethyl modification enzyme GidA  1(5) | tRNA uridine 5-carboxymethylaminomethyl modification enzyme GidA  1(5) | tRNA uridine 5-carboxymethylaminomethyl modification enzyme mnmG  1(5) | tRNA uridine 5-carboxymethylaminomethyl modification enzyme GidA  1(5) |
|  | [YP_001933051.1](http://www.ncbi.nlm.nih.gov/protein/189025279) | **B2S1Z3** | adenosine deaminase | adenosine deaminase  1(5) | Adenosine/AMP deaminase  1(5) | Adenosine deaminase  1(5) | Adenosine/AMP deaminase domain  1(5) |
|  | [YP_001933057.1](http://www.ncbi.nlm.nih.gov/protein/189025285) | **B2S1Z9** | peptide chain release factor 1 | peptide chain release factor 1  1(5) | peptide chain release factor 1  1(5) | Peptide chain release factor 1  1(5) | Peptide chain release factor 1  1(5) |
|  | [YP_001933058.1](http://www.ncbi.nlm.nih.gov/protein/189025286) | **B2S200** | protoporphyrinogen oxidase | protoporphyrinogen oxidase (hemK)  1(5) | methyltransferase, HemK family  1(2) | Protoporphyrinogen oxidase  1(5) | methyltransferase, HemK family  1(2) |
|  | [YP_001933059.1](http://www.ncbi.nlm.nih.gov/protein/189025287) | **B2S201** | ribonucleotide-diphosphate reductase subunit beta | ribonucleotide-diphosphate reductase subunit beta  1(5) | Ribonucleotide reductase, small chain  1(4) | Ribonucleoside-diphosphate reductase subunit beta  1(5) | Ribonucleotide reductase small subunit  1(4) |
|  | [YP_001933062.1](http://www.ncbi.nlm.nih.gov/protein/189025290) | **B2S204** | pyruvate carboxylase subunit B | pyruvate carboxylase subunit B  1(5) | oxaloacetate decarboxylase alpha subunit  1(4) | Oxaloacetate decarboxylase, subunit alpha  1(4) | Pyruvate carboxyltransferase  1(4) |
|  | [YP_001933063.1](http://www.ncbi.nlm.nih.gov/protein/189025291) | **B2S205** | oxaloacetate decarboxylase, subunit beta | oxaloacetate decarboxylase, subunit beta (oadB)  1(5) | sodium ion-translocating decarboxylase, beta subunit  1(3) | Oxaloacetate decarboxylase, subunit beta  1(5) | sodium ion-translocating decarboxylase, beta subunit  1(3) |
|  | [YP_001933064.1](http://www.ncbi.nlm.nih.gov/protein/189025292) | **B2S206** | replicative DNA helicase DnaB | replicative DNA helicase (dnaB)  1(5) | replicative DNA helicase  1(5) | Replicative DNA helicase  1(5) | DNA helicase, DnaB-like, C-terminal  1(4) |
|  | [YP_001933066.1](http://www.ncbi.nlm.nih.gov/protein/189025294) | **B2S208** | 50S ribosomal protein L9 | 50S ribosomal protein L9  1(5) | Ribosomal Protein L9  1(4) | 50S ribosomal protein L9  1(5) | 50S ribosomal protein L9  1(5) |
|  | [YP_001933067.1](http://www.ncbi.nlm.nih.gov/protein/189025295) | **B2S209** | 30S ribosomal protein S18 | 30S ribosomal protein S18  1(5) | 30s Ribosomal Protein S18  1(5) | 30S ribosomal protein S18  1(5) | 30S ribosomal protein S18  1(5) |
|  | [YP_001933068.1](http://www.ncbi.nlm.nih.gov/protein/189025296) | **B2S210** | single-strand DNA binding protein Ssb | single-strand DNA binding protein (ssb)  1(5) | single-stranded DNA-binding protein  1(5) | Single-stranded DNA-binding protein  1(5) | single-stranded DNA-binding protein  1(5) |
|  | [YP_001933069.1](http://www.ncbi.nlm.nih.gov/protein/189025297) | **B2S211** | 30S ribosomal protein S6 | 30S ribosomal protein S6  1(5) | Ribosomal protein S6  1(4) | 30S ribosomal protein S6  1(5) | 30S ribosomal protein S6  1(5) |
|  | [YP_001933077.1](http://www.ncbi.nlm.nih.gov/protein/189025305) | **B2S219** | ATP-dependent Clp protease, subunit B | ATP-dependent Clp protease subunit B (clpB)  1(5) | ATP-dependent chaperone protein ClpB  1(5) | Chaperone protein clpB  1(5) | ATP-dependent Clp protease  1(5) |
|  | [YP_001933080.1](http://www.ncbi.nlm.nih.gov/protein/189025308) | **B2S222** | sugar ABC transporter, periplasmic binding protein | sugar ABC transporter, periplasmic binding protein  1(5) | Periplasmic binding protein-like II  1(4) | Sugar ABC transporter, periplasmic binding protein  1(5) | Bacterial extracellular solute-binding protein  1(3) |
|  | [YP_001933081.1](http://www.ncbi.nlm.nih.gov/protein/189025309) | **B2S223** | sugar ABC transporter, permease protein | sugar ABC transporter, permease protein (y4oQ)  1(5) | Binding-protein-dependent transport system inner membrane component  1(3) | Sugar ABC transporter, permease protein  1(5) | Binding-protein-dependent transport system inner membrane component  1(3) |
|  | [YP_001933082.1](http://www.ncbi.nlm.nih.gov/protein/189025310) | **B2S224** | sugar ABC transporter, permease protein | sugar ABC transporter, permease protein  1(5) | Binding-protein-dependent transport system inner membrane component  1(3) | Sugar ABC transporter, permease protein  1(5) | Binding-protein-dependent transport system inner membrane component  1(3) |
|  | [YP_001933083.1](http://www.ncbi.nlm.nih.gov/protein/189025311) | **B2S225** | capsular polysaccharide biosynthesis protein | capsular polysaccharide biosynthesis protein (cap5D)  1(5) | Polysaccharide biosynthesis protein  1(4) | Capsular polysaccharide biosynthesis protein  1(5) | Polysaccharide biosynthesis protein CapD-like  1(5) |
|  | [YP_001933084.1](http://www.ncbi.nlm.nih.gov/protein/189025312) | **B2S226** | spore coat polysaccharide biosynthesis protein,  DegT/DnrJ/EryC1/StrS family pyridoxal dependent aminotransferase | spore coat polysaccharide biosynthesis protein (spsC)  1(5) | PLP-dependent transferases  1(5) | Spore coat polysaccharide biosynthesis protein  1(5) | Pyridoxal phosphate-dependent transferase  1(5) |
|  | [YP_001933086.1](http://www.ncbi.nlm.nih.gov/protein/189025314) | **B2S228** | quinoline 2-oxidoreductase | quinoline 2-oxidoreductase  1(5) | [2Fe-2S]-binding  1(2) | Quinoline 2-oxidoreductase  1(5) | [2Fe-2S]-binding  1(2) |
|  | [YP_001933088.1](http://www.ncbi.nlm.nih.gov/protein/189025316) | **B2S230** | formate hydrogenlyase transcriptional activator FhlA | formate hydrogenlyase transcriptional activator (fhlA)  1(5) | Sigma-54 interaction domain  1(3) | Formate hydrogenlyase transcriptional activator FhlA  1(5) | Sigma-54 interaction domain  1(3) |
|  | [YP_001933091.1](http://www.ncbi.nlm.nih.gov/protein/189025319) | **B2S233** | PTS system, nitrogen regulatory IIA component | PTS system, nitrogen regulatory IIA component (ptsN-1)  1(5) | Phosphoenolpyruvate-dependent sugar phosphotransferase system, EIIA 2  1(5) | PTS system, nitrogen regulatory IIA component  1(5) | Phosphoenolpyruvate-dependent sugar phosphotransferase system, EIIA 2  1(5) |
|  | [YP_001933095.1](http://www.ncbi.nlm.nih.gov/protein/189025323) | **B2S237** | cyclic nucleotide binding protein | cyclic nucleotide binding protein  1(5) | Cyclic nucleotide-binding domain  1(5) | Cyclic nucleotide binding protein  1(5) | Cyclic nucleotide-binding domain  1(5) |
|  | [YP_001933096.1](http://www.ncbi.nlm.nih.gov/protein/189025324) | **B2S238** | UDP-N-acetylenolpyruvoylglucosamine reductase | UDP-N-acetylenolpyruvoylglucosamine reductase  1(5) | UDP-N-acetylenolpyruvoylglucosamine reductase, C-terminal domain  1(5) | UDP-N-acetylenolpyruvoylglucosamine reductase  1(5) | UDP-N-acetylenolpyruvoylglucosamine reductase  1(5) |
|  | [YP_001933097.1](http://www.ncbi.nlm.nih.gov/protein/189025325) | **B2S239** | cysteinyl-tRNA synthetase | cysteinyl-tRNA synthetase  1(5) | tRNA synthetases class I (C) catalytic domain  1(4) | Cysteinyl-tRNA synthetase  1(5) | Cysteinyl-tRNA synthetase/mycothiol ligase  1(4) |
|  | [YP_001933098.1](http://www.ncbi.nlm.nih.gov/protein/189025326) | **B2S240** | RNA polymerase sigma-24 factor | RNA polymerase sigma-24 factor  1(5) | RNA polymerase sigma factor, sigma-70 family  1(5) | RNA polymerase sigma-24 factor  1(5) | RNA polymerase sigma factor  1(5) |
|  | [YP_001933100.1](http://www.ncbi.nlm.nih.gov/protein/189025328) | **B2S242** | phosphate acetyltransferase | phosphate acetyltransferase (pta)  1(5) | phosphate acetyltransferase  1(5) | Phosphate acetyltransferase  1(5) | phosphate acetyltransferase  1(5) |
|  | [YP_001933102.1](http://www.ncbi.nlm.nih.gov/protein/189025330) | **B2S244** | dnaK suppressor | dnaK suppressor  1(5) | DnaK suppressor protein DksA, alpha-hairpin domain  1(5) | dnaK suppressor  1(5) | DksA/TraR zinc finger signature  1(3) |
|  | [YP_001933103.1](http://www.ncbi.nlm.nih.gov/protein/189025331) | **B2S245** | translation initiation factor IF-1 | translation initiation factor IF-1  1(5) | translation initiation factor IF-1  1(5) | Translation initiation factor IF-1  1(5) | translation initiation factor IF-1  1(5) |
|  | [YP_001933104.1](http://www.ncbi.nlm.nih.gov/protein/189025332) | **B2S246** | heat-shock protein | heat-shock protein  1(5) | DnaJ domain  1(3) | heat-shock protein  1(5) | DnaJ domain  1(3) |
|  | [YP_001933105.1](http://www.ncbi.nlm.nih.gov/protein/189025333) | **B2S247** | uridylate kinase | uridylate kinase  1(5) | Amino acid kinase family  1(4) | Uridylate kinase  1(5) | Uridylate kinase  1(5) |
|  | [YP_001933106.1](http://www.ncbi.nlm.nih.gov/protein/189025334) | **B2S248** | thioredoxin | Thioredoxin  1(5) | Thioredoxin-like  1(4) | Thioredoxin  1(5) | Thioredoxin domain  1(5) |
|  | [YP_001933107.1](http://www.ncbi.nlm.nih.gov/protein/189025335) | **B2S249** | cytochrome c biogenesis protein | cytochrome c biogenesis protein (ccdA)  1(5) | Cytochrome C biogenesis protein transmembrane region  1(5) | Cytochrome c biogenesis protein  1(5) | Cytochrome C biogenesis protein transmembrane region  1(5) |
|  | [YP_001933108.1](http://www.ncbi.nlm.nih.gov/protein/189025336) | **B2S250** | Rep helicase, single-stranded DNA-dependent ATPase | rep helicase, single-stranded DNA-dependent ATPase (rep)  1(5) | UvrD/REP helicase N-terminal domain  1(4) | Rep helicase, single-stranded DNA-dependent ATPase  1(5) | UvrD/REP helicase N-terminal domain  1(4) |
|  | [YP_001933109.1](http://www.ncbi.nlm.nih.gov/protein/189025337) | **B2S251** | ATP-dependent DNA helicase | ATP-dependent DNA helicase  1(5) | Helicase conserved C-terminal domain  1(4) | ATP-dependent DNA helicase  1(5) | helicase superfamily c-terminal domain  1(4) |
|  | [YP_001933110.1](http://www.ncbi.nlm.nih.gov/protein/189025338) | **B2S252** | 5'-nucleotidase | 5'-nucleotidase (ushA)  1(5) | 5'-nucleotidase, C-terminal domain  1(5) | 5'-nucleotidase1(5) | 5'-Nucleotidase, C-terminal  1(5) |
|  | [YP_001933111.1](http://www.ncbi.nlm.nih.gov/protein/189025339) | **B2S253** | DNA polymerase I | DNA polymerase I  1(5) | DNA polymerase I  1(5) | DNA polymerase I  1(5) | DNA polymerase I  1(5) |
|  | [YP_001933112.1](http://www.ncbi.nlm.nih.gov/protein/189025340) | **B2S254** | carnitine transporter | carnitine transporter  1(5) | BCCT family transporter  1(4) | carnitine transporter  1(5) | BCCT family transporter  1(4) |
|  | [YP_001933113.1](http://www.ncbi.nlm.nih.gov/protein/189025341) | **B2S255** | protein LicC | LicC protein  1(5) | Choline/ethanolamine kinase  1(5) | Protein LicC  1(5) | Choline/ethanolamine kinase  1(5) |
|  | [YP_001933114.1](http://www.ncbi.nlm.nih.gov/protein/189025342) | **B2S256** | diphosphate--fructose-6-phosphate 1-phosphotransferase | diphosphate--fructose-6-phosphate 1-phosphotransferase  1(5) | Phosphofructokinase  1(5) | Pyrophosphate--fructose 6-phosphate 1-phosphotransferase  1(5) | Pyrophosphate-dependent phosphofructokinase  1(5) |
|  | [YP_001933115.1](http://www.ncbi.nlm.nih.gov/protein/189025343) | **B2S257** | rRNA methylase | rRNA methylase  1(5) | SpoU rRNA Methylase family  1(5) | rRNA methylase  1(5) | rRNA Methylase  1(5) |
|  | [YP_001933117.1](http://www.ncbi.nlm.nih.gov/protein/189025345) | **B2S259** | RNA polymerase sigma-54 factor | RNA polymerase sigma-54 factor  1(5) | RNA polymerase sigma-54 factor  1(5) | RNA polymerase sigma-54 factor  1(5) | RNA polymerase sigma factor 54  1(5) |
| 1. ) | [YP_001933118.1](http://www.ncbi.nlm.nih.gov/protein/189025346) | **B2S260** | aminopeptidase C | aminopeptidase C (pepC)  1(5) | Peptidase C1-like family  1(4) | Aminopeptidase C  1(5) | Peptidase C1-like family  1(4) |
|  | [YP_001933119.1](http://www.ncbi.nlm.nih.gov/protein/189025347) | **B2S261** | Lambda CII stability-governing protein, HflK protein | lambda CII stability-governing protein (hflK)  1(5) | HflK protein  1(5) | Protein hflK  1(5) | HflK protein  1(5) |
|  | [YP_001933120.1](http://www.ncbi.nlm.nih.gov/protein/189025348) | **B2S262** | Lambda CII stability-governing protein | lambda CII stability-governing protein (hflK)  1(5) | HflK protein  1(5) | Protein hflK  1(5) | HflK protein  1(5) |
|  | [YP_001933121.1](http://www.ncbi.nlm.nih.gov/protein/189025349) | **B2S263** | phosphomethypyrimidine kinase | phosphomethypyrimidine kinase  1(5) | Phosphomethylpyrimidine kinase  1(5) | Phosphomethypyrimidine kinase  1(5) | Phosphomethylpyrimidine kinase  1(5) |
|  | [YP_001933122.1](http://www.ncbi.nlm.nih.gov/protein/189025350) | **B2S264** | excinuclease ABC subunit B | excinuclease ABC subunit B  1(5) | Ultra-violet resistance protein B  1(5) | UvrABC system protein B  1(5) | UvrABC system, subunit B  1(5) |
|  | [YP_001933125.1](http://www.ncbi.nlm.nih.gov/protein/189025353) | **B2S267** | amino acid ABC transporter, permease protein | amino acid ABC transporter, permease protein (yaeE)  1(5) | Binding-protein-dependent transport system inner membrane component  1(4) | Amino acid ABC transporter, permease protein  1(5) | Binding-protein-dependent transport system inner membrane component  1(4) |
|  | [YP_001933126.1](http://www.ncbi.nlm.nih.gov/protein/189025354) | **B2S268** | amino acid ABC transporter, ATP-binding protein | amino acid ABC transporter ATP-binding protein  1(5) | ABC transporter  1(4) | Amino acid ABC transporter, ATP-binding protein  1(5) | ATP-binding cassette, ABC transporter-type domain  1(5) |
|  | [YP_001933128.1](http://www.ncbi.nlm.nih.gov/protein/189025356) | **B2S270** | phosphoenolpyruvate carboxykinase | phosphoenolpyruvate carboxykinase  1(5) | Phosphoenolpyruvate carboxykinase  1(5) | Phosphoenolpyruvate carboxykinase [GTP]  1(5) | Phosphoenolpyruvate carboxykinase  1(5) |
|  | [YP_001933130.1](http://www.ncbi.nlm.nih.gov/protein/189025358) | **B2S272** | GTP-dependent nucleic acid-binding protein EngD | GTP-dependent nucleic acid-binding protein EngD  1(5) | GTP-binding protein YchF  1(4) | GTP-binding protein  1(4) | GTP-binding protein YchF  1(4) |
|  | [YP_001933131.1](http://www.ncbi.nlm.nih.gov/protein/189025359) | **B2S273** | exodeoxyribonuclease | exodeoxyribonuclease (exoA)  1(5) | exodeoxyribonuclease III  1(5) | Exodeoxyribonuclease  1(5) | exodeoxyribonuclease III  1(5) |
|  | [YP_001933146.1](http://www.ncbi.nlm.nih.gov/protein/189025374) | **B2S288** | K+ transport protein | K+ transport protein (ntpJ)  1(5) | potassium uptake protein, TrkH family  1(5) | Cation transport protein  1(3) | Cation transport protein  1(3) |
|  | [YP_001933147.1](http://www.ncbi.nlm.nih.gov/protein/189025375) | **B2S289** | methylated-DNA-protein-cysteine S-methyltransferase | methylated-DNA-protein-cysteine S-methyltransferase (dat)  1(5) | methylated-DNA--[protein]-cysteine S-methyltransferase  1(5) | Methylated-DNA-protein-cysteine S-methyltransferase  1(5) | Methylated-DNA--[protein]-cysteine S-methyltransferase  1(5) |
|  | [YP_001933165.1](http://www.ncbi.nlm.nih.gov/protein/189025393) | **B2S2A7** | prolyl-tRNA synthetase | prolyl-tRNA synthetase  1(5) | tRNA synthetase class II core domain (G, H, P, S and T)  1(4) | Prolyl-tRNA synthetase  1(5) | Prolyl-tRNA synthetase  1(5) |
|  | [YP_001933167.1](http://www.ncbi.nlm.nih.gov/protein/189025395) | **B2S2A9** | Holliday junction DNA helicase RuvB | Holliday junction DNA helicase RuvB  1(5) | Holliday junction DNA helicase RuvB  1(5) | Holliday junction ATP-dependent DNA helicase ruvB  1(5) | Holliday junction ATP-dependent DNA helicase ruvB  1(5) |
|  | [YP_001933168.1](http://www.ncbi.nlm.nih.gov/protein/189025396) | **B2S2B0** | ABC transporter, periplasmic binding protein | ABC transporter, periplasmic binding protein (troA)  1(5) | Periplasmic solute binding protein family  1(4) | Periplasmic zinc-binding protein troA  1(4) | Periplasmic solute binding protein family  1(4) |
|  | [YP_001933169.1](http://www.ncbi.nlm.nih.gov/protein/189025397) | **B2S2B1** | ABC transporter, ATP-binding protein | ABC transporter, ATP-binding protein (troB)  1(5) | ABC transporter ATPase domain-like  1(4) | Zinc transport system ATP-binding protein troB  1(5) | ABC transporter  1(4) |
|  | [YP_001933170.1](http://www.ncbi.nlm.nih.gov/protein/189025398) | **B2S2B2** | ABC transporter, permease protein | ABC transporter, permease protein (troC)  1(5) | ABC transporter involved in vitamin B12 uptake  1(4) | Zinc transport system ATP-binding protein troB  1(5) | ABC 3 transport family  1(4) |
|  | [YP_001933171.1](http://www.ncbi.nlm.nih.gov/protein/189025399) | **B2S2B3** | ABC transporter, permease protein | ABC transporter, permease protein (troD)  1(5) | ABC transporter involved in vitamin B12 uptake  1(4) | ABC transporter, permease protein  1(5) | ABC 3 transport family  1(4) |
|  | [YP_001933172.1](http://www.ncbi.nlm.nih.gov/protein/189025400) | **B2S2B4** | cation-activated repressor protein | cation-activated repressor protein (troR)  1(5) | Iron dependent repressor  1(4) | Cation-activated repressor protein  1(5) | Iron dependent repressor  1(4) |
|  | [YP_001933173.1](http://www.ncbi.nlm.nih.gov/protein/189025401) | **B2S2B5** | phosphoglyceromutase | Phosphoglyceromutase  1(5) | Phosphoglycerate mutase-like  1(4) | 2,3-bisphosphoglycerate-dependent phosphoglycerate mutase  1(5) | 2,3-bisphosphoglycerate-dependent phosphoglycerate mutase  1(5) |
|  | [YP_001933175.1](http://www.ncbi.nlm.nih.gov/protein/189025403) | **B2S2B7** | protein Pfs | pfs protein (pfs)  1(5) | Purine and uridine phosphorylases  1(4) | MTA/SAH nucleosidase  1(4) | Nucleoside phosphorylase  1(5) |
|  | [YP_001933176.1](http://www.ncbi.nlm.nih.gov/protein/189025404) | **B2S2B8** | lipoprotein, 15 kDa | lipoprotein, 15 kDa (tpp15)  1(5) | FMN-binding domain  1(3) | Lipoprotein, 15 kDa  1(5) | FMN-binding domain  1(3) |
|  | [YP_001933189.1](http://www.ncbi.nlm.nih.gov/protein/189025417) | **B2S2D1** | SsrA-binding protein | SsrA-binding protein  1(5) | SsrA-binding protein  1(5) | SsrA-binding protein  1(5) | SsrA-binding protein  1(5) |
|  | [YP_001933190.1](http://www.ncbi.nlm.nih.gov/protein/189025418) | **B2S2D2** | signal peptidase I | signal peptidase I (sip)  1(5) | signal peptidase I  1(5) | Signal peptidase I  1(5) | signal peptidase I  1(5) |
|  | [YP_001933191.1](http://www.ncbi.nlm.nih.gov/protein/189025419) | **B2S2D3** | oxygen-independent coproporphyrinogen III oxidase | oxygen-independent coproporphyrinogen III oxidase  1(5) | Oxygen-independent coproporphyrinogen III oxidase HemN  1(5) | oxygen-independent coproporphyrinogen III oxidase  1(5) | HemN C-terminal domain  1(4) |
|  | [YP_001933192.1](http://www.ncbi.nlm.nih.gov/protein/189025420) | **B2S2D4** | elongation factor Tu | elongation factor Tu  1(5) | Elongation factor Tu GTP binding domain  1(5) | Elongation factor Tu  1(5) | Elongation factor Tu [tuf]  1(5) |
|  | [YP_001933193.1](http://www.ncbi.nlm.nih.gov/protein/189025421) | **B2S2D5** | 30S ribosomal protein S10 | 30S ribosomal protein S10  1(5) | Ribosomal protein S10  1(4) | 30S ribosomal protein S10  1(5) | 30S ribosomal protein S10 [rpsJ]  1(5) |
|  | [YP_001933194.1](http://www.ncbi.nlm.nih.gov/protein/189025422) | **B2S2D6** | 50S ribosomal protein L3 | 50S ribosomal protein L3  1(5) | 50S ribosomal protein L3  1(5) | 50S ribosomal protein L3  1(5) | 50S ribosomal protein L3 [rplC]  1(5) |
|  | [YP_001933195.1](http://www.ncbi.nlm.nih.gov/protein/189025423) | **B2S2D7** | 50S ribosomal protein L4 | 50S ribosomal protein L4  1(5) | 50S ribosomal protein L4  1(5) | 50S ribosomal protein L4  1(5) | 50S ribosomal protein L4  1(5) |
|  | [YP_001933196.1](http://www.ncbi.nlm.nih.gov/protein/189025424) | **B2S2D8** | 50S ribosomal protein L23 | 50S ribosomal protein L23  1(5) | Ribosomal protein L23  1(4) | 50S ribosomal protein L23  1(5) | 50S ribosomal protein L23 [rplW]  1(5) |
|  | [YP_001933197.1](http://www.ncbi.nlm.nih.gov/protein/189025425) | **B2S2D9** | 50S ribosomal protein L2 | 50S ribosomal protein L2  1(5) | ribosomal protein L2  1(4) | 50S ribosomal protein L2  1(5) | 50S ribosomal protein L2 [rplB]  1(5) |
|  | [YP_001933198.1](http://www.ncbi.nlm.nih.gov/protein/189025426) | **B2S2E0** | 30S ribosomal protein S19 | 30S ribosomal protein S19  1(5) | Ribosomal protein S19  1(4) | 30S ribosomal protein S19  1(5) | 30S ribosomal protein S19 [rpsS]  1(5) |
|  | [YP_001933199.1](http://www.ncbi.nlm.nih.gov/protein/189025427) | **B2S2E1** | 50S ribosomal protein L22 | 50S ribosomal protein L22  1(5) | Ribosomal protein L22  1(4) | 50S ribosomal protein L22  1(5) | 50S ribosomal protein L22 [rplV]  1(5) |
|  | [YP_001933200.1](http://www.ncbi.nlm.nih.gov/protein/189025428) | **B2S2E2** | 30S ribosomal protein S3 | 30S ribosomal protein S3  1(5) | ribosomal protein S3  1(4) | 30S ribosomal protein S3  1(5) | Ribosomal protein S3  1(4) |
|  | [YP_001933211.1](http://www.ncbi.nlm.nih.gov/protein/189025439) | **B2S2F3** | 30S ribosomal protein S5 | 30S ribosomal protein S5  1(5) | ribosomal protein S5  1(4) | 30S ribosomal protein S5  1(5) | 30S ribosomal protein S5 [rpsE]  1(5) |
|  | [YP_001933212.1](http://www.ncbi.nlm.nih.gov/protein/189025440) | **B2S2F4** | 50S ribosomal protein L30 | 50S ribosomal protein L30  1(5) | ribosomal protein L30  1(4) | 50S ribosomal protein L30  1(5) | 50S ribosomal protein L30 [rpmD]  1(5) |
| 100. | YP_001933213.1 | **B2S2F5** | 50S ribosomal protein L15 | 50S ribosomal protein L15  1(5) | ribosomal protein L15  1(4) | 50S ribosomal protein L15  1(5) | 50S ribosomal protein L15 [rplO]  1(5) |
