# Supplementary material for: Identification of Functional Candidates amongst Hypothetical Proteins of Treponema pallidum ssp. pallidum
Source: PLoS One. 2015 Apr 20;10(4):e0124177. doi: 10.1371/journal.pone.0124177 (PMC4403809; doi:10.1371/journal.pone.0124177)
Supplement: S7 Table — (DOC) [file pone.0124177.s007.doc]

| **Table S7: List of functionally annotated domain of 100 proteins with known function from *T. pallidum* *ssp. pallidum* by CATH, SUPERFAMILY, PANTHER, CDART, Pfam, SYSTERS, and ProtoNet for ROC analysis.** | | | | | | | | | | |
| --- | --- | --- | --- | --- | --- | --- | --- | --- | --- | --- |
| **S. No** | **Uniprot ID** | **Protein Name** | **CATH** | **SUPER**  **FAMILY** | **Panther** | | **CDART** | **Pfam**  **(family/Domain)** | **SYSTERS**  **(Cluster** | **ProtoNet**  **Cluster**  **(cluster name)** |
|  | B2S1V1 | chromosomal replication initiation protein | Chromosomal replication initiator protein DnaA -like domain  1(5) | Chromosomal replication initiation factor DnaA C-terminal domain IV  1(4) | CHROMOSOMAL REPLICATION INITIATOR PROTEIN DNAA  1(5) | | DnaA_N superfamily  1(4) | DnaA N-terminal domain  1(4) | Cluster 146901  (Chromosomal replication initiator protein dnaA)  1(5) | Cluster 4155404  Cluster Name: Chromosomal replication control, initiator DnaA  1(5) |
|  | **B2S1V2** | DNA-directed DNA polymerase beta subunit | DNA polymerase III subunit beta -like domain  1(5) | DNA polymerase III, beta subunit  1(5) | DNA POLYMERASE III SUBUNIT BETA  1(5) | | Beta_clamp superfamily  (beta subunit of DNA polymerase III holoenzyme)  1(5) | DNA_pol3_beta family  (DNA polymerase III beta subunit)  1(5) | Cluster 143457  (DNA polymerase III, beta chain)  1(5) | Cluster 4188337  Cluster Name: DNA Polymerase III; Chain A, domain 2  1(5) |
|  | **B2S1V3** | recombination protein RecF | Mx1 protein -like domain  1(2) | P-loop containing nucleoside triphosphate hydrolase  1(2) | DNA REPLICATION AND REPAIR PROTEIN RECF  1(5) | | ABC_ATPase superfamily  1(2) | AAA_23  Family  1(2) | Cluster 148110  (DNA replication and repair protein recF)  1(5) | Cluster 4152794  Cluster Name: DNA-binding, RecF  1(5) |
|  | B2S1V5 | DNA topoisomerase (ATP-hydrolyzing) subunit A  (DNA gyrase) | DNA gyrase subunit A -like domain  1(5) | Type II DNA topoisomerase  1(5) | : DNA GYRASE SUBUNIT A, CHLOROPLASTIC/MITOCHONDRIAL-RELATED  1(5) | | TOP4c superfamily  (DNA Topoisomerase, subtype IIA; domain A)  1(5) | DNA_topoisoIV family  (DNA gyrase/topoisomerase IV, subunit A)  1(5) | Cluster 137822  (DNA topoisomerase IV ParC subunit)  1(5) | Cluster 4147839  Cluster Name: Topoisomerase II; domain 5  1(5) |
|  | **B2S1W3** | phenylalanyl-tRNA synthetase subunit beta | No result  0(5) | Class II aminoacyl-tRNA synthetase (aaRS)-like, catalytic domain  1(4) | PHENYLALANYL-TRNA SYNTHETASE BETA CHAIN AND LEUCINE-RICH REPEAT-CONTAINING PROTEIN 47  1(5) | | B3_4 superamily  (domain found in tRNA synthetase beta subunit)  1(5) | B5 family  (tRNA synthetase B5 domain)  1(5) | Cluster 139353  (Phenylalanyl-tRNA synthetase beta chain)  1(5) | Cluster 4094159Cluster Name: Phenylalanyl-tRNA synthetase, class IIc, beta subunit, archae/euk cytosolic  1(5) |
|  | **B2S1W4** | ATP-dependent protease LA | Archaeal Lon protease -like domain  1(4) | ATP-dependent protease Lon (La), catalytic domain  1(5) | LON PROTEASE HOMOLOG-RELATED  1(4) | | Lon_C superfamily  (Lon protease (S16) C-terminal proteolytic domain)  1(5) | Lon_C family  (Lon protease (S16) C-terminal proteolytic domain)  1(5) | Cluster 144105  (ATP-dependent protease La (EC 3.4.21.53))  1(5) | Cluster 4146748  Cluster Name: Peptidase S16, Lon protease, C-terminal region  1(5) |
|  | **B2S1W6** | transcription elongation factor | Bacteriophage N4 adsorption protein A -like domain  1(2) | TPR like  1(2) | TRANSCRIPTION ELONGATION FACTOR GREA  1(5) | | GreA_GreB_N superfamilly  (Transcription elongation factor, N-terminal)  1(5) | TPR_8 family  1(2) | Cluster 143924  (TPR)  1(2) | Cluster 3861180  Cluster Name: GreA transcript cleavage protein, N-terminal domain  1(5) |
|  | **B2S1W7** | transcription elongation factor GreA, partial | Transcription elongation factor greA -like domain  1(5) | GreA transcript cleavage protein, N-terminal domain  1(5) | TRANSCRIPTION ELONGATION FACTOR GREB  1(4) | | GreA_GreB_N superfamilly  (Transcription elongation factor, N-terminal)  1(5) | GreA_GreB_N familly  (Transcription elongation factor, N-terminal)  1(5) | Cluster 155129  (Transcription elongation factor greA)  1(5) | Cluster 4315654Cluster Name: GreA transcript cleavage protein, N-terminal domain  1(5) |
|  | **B2S1X1** | sodium- and chloride- dependent transporter | No result  0 | SNF like  (Ion transport)  1(4) | SODIUM/CHLORIDE DEPENDENT TRANSPORTER  1(5) | | SLC5-6-like_sbd superfamily  (sodium- and chloride-dependent neurotransmitter transporters)  1(5) | SNF family  (Sodium:neurotransmitter symporter family)  1(4) | Cluster 140814  (Sodium- and chloride-dependent transporter)  1(5) | Cluster 4133831  Cluster Name: Sodium:neurotransmitter symporter  1(5) |
|  | **B2S1X4** | flagellar motor switch protein | Flagellar motor switch protein -like domain  1(5) | FliG superfamily  1(4) | FLAGELLAR MOTOR SWITCH PROTEIN FLIG  1(5) | | FliG_C famiy  (FliG C-terminal domain)  1(5) | FliG_C famiy  (FliG C-terminal domain)  1(5) | Cluster 145955  (Flagellar motor switch protein fliG)  1(5) | Cluster 4257921  Cluster Name: Flagellar motor switch protein FliG  1(5) |
|  | **B2S1X5** | hemolysin | No result  0 | CBS-domain pair  1(2) | HEMOLYSIN-RELATED 1(4) | | CBS_pair superfamily  1(2) | CBS family  1(2) | Cluster 143846 (Hemolysin)  1(5) | Cluster 4144744  Cluster Name: Transporter-associated region  1(2) |
|  | **B2S1X6** | hemolysin | No result  0 | CBS-domain pair  1(2) | HEMOLYSIN-RELATED 1(4) | | CBS_pair superfamily  1(2) | CBS family  1(2) | Cluster 143846 (Hemolysin)  1(5) | Cluster 4036317  Cluster Name: Transporter-associated region  1(5) |
|  | **B2S1X7** | UDP-N-acetylglucosamine 1-carboxyvinyltransferase | 3-phosphoshikimate 1-carboxyvinyltransferase -like domain  1(4) | Enolpyruvate transferase, EPT  1(3) | UDP-N-ACETYLGLUCOSAMINE 1-CARBOXYVINYLTRANSFERASE  1(5) | | EPT_RTPC like superfamily  1(5) | EPSP_synthase family  (EPSP synthase (3-phosphoshikimate 1-carboxyvinyltransferase))  1(4) | Cluster 144179  (UDP-N-acetylglucosamine 1-carboxyvinyltransferase (EC 2.5.1.7))  1(5) | Cluster 4024083  Cluster Name: UDP-N-acetylglucosamine 1-carboxyvinyltransferase  1(5) |
|  | **B2S1X8** | chaperonin GroEL | 60 kDa chaperonin -like domain  1(5) | GroEL-like chaperone, apical domain  1(5) | CHAPERONIN  1(5) | | chaperonin –like superfamily  1(5) | Cpn60_TCP1  (TCP-1/cpn60 chaperonin family)  1(5) | Cluster 142088  (60 kDa chaperonin (Protein Cpn60) (groEL protein)  1(5) | Cluster 4437231  Cluster Name: GroEL equatorial domain-like  1(5) |
|  | **B2S1Y0** | 16S ribosomal RNA methyltransferase RsmE | Ribosomal RNA small subunit methyltransferase E -like domain  1(5) | SpoU-like RNA 2'-O ribose methyltransferase  1(4) | RIBOSOMAL RNA SMALL SUBUNIT METHYLTRANSFERASE E  1(5) | | Methyltrans_RNA superfamily  (RNA methyltransferase)  1(5) | Methyltrans_RNA family  (RNA methyltransferase)  1(5) | Cluster 153855  0 | Cluster 4253449  Cluster Name: Ribosomal RNA small subunit methyltransferase E  1(5) |
|  | **B2S1Y2** | ABC transporter, periplasmic binding protein | Light-independent protochlorophyllide reductase -like domain  1(3) | "Helical backbone" metal receptor  1(5) | ABC-TRANSPORTER METAL-BINDING PROTEIN  1(4) | | TroA_like superfamily  1(4) | TroA  Family  (Periplasmic solute binding protein)  1(5) | Cluster 140437  (ABC-type transporter, periplasmic component)  1(5) | Cluster 4153106  Cluster Name: Adhesin B  1(5) |
|  | **B2S1Y3** | ABC transporter, ATP-binding protein | metal transport system ATP-binding -like domain  1(5) | ABC transporter ATPase domain-like  1(5) | ATP-binding cassette (ABC) transporter  1(5) | | ABC_ATPase superfamily  (ATP-binding cassette transporter nucleotide-binding domain)  1(5) | ABC_tran  Family  (ABC transporter)  1(5) | Cluster 137568  (metal transport system ATP-binding protein)  1(5) | Cluster 4221499  Cluster Name: ABC transporter-like  1(5) |
|  | **B2S1Y4** | ABC transporter, permease protein | No result  0 | ABC transporter involved in vitamin B12 uptake, BtuC  1(5) | ABC-TRANSPORTER METAL-BINDING PROTEIN  1(5) | | TM_ABC_iron-siderophores_like Superfamily  1(5) | ABC-3  Family  (ABC 3 transport family)  1(5) | Cluster 149307  (ABC-type transporter, permease components)  1(5) | Cluster 4165676  Cluster Name: ABC-3  1(5) |
|  | **B2S1Y5** | D-specific D-2-hydroxyacid dehydrogenase | Erythronate-4-phosphate dehydrogenase -like domain  1(4) | Formate/glycerate dehydrogenases, NAD-domain  1(4) | 2-HYDROXYACID DEHYDROGENASE-RELATED  1(5) | | FDH_GDH_like  formate/glycerate dehydrogenases, D-specific 2-hydroxy acid dehydrogenases and related dehydrogenases  1(4) | 2-Hacid_dh family  (D-isomer specific 2-hydroxyacid dehydrogenase, catalytic domain)  1(5) | Cluster 139457  (2-hydroxyacid dehydrogenase)  1(5) | Cluster 4188286  Cluster Name: D-isomer specific 2-hydroxyacid dehydrogenase, NAD-binding  1(5) |
|  | **B2S1Y6** | regulatory protein PfoS/R | No result  0 | No result  0 | No result  0 | | PfoR superfamily  (membrane protein, putative toxin regulator)  1(5) | PTS_EIIC_2 family  (Phosphotransferase system, EIIC)  1(3) | Cluster 149635  (Regulatory protein PFOS/R)  1(5) | Cluster 4109239  Cluster Name: Firmicutes  1(5) |
|  | **B2S1Y8** | methyl-accepting chemotaxis protein | Methyl-accepting chemotaxis protein -like domain  1(5) | Methyl-accepting chemotaxis protein (MCP) signaling domain  1(5) | No result  0 | | MCP_signal superfamily  1(5) | MCPsignal  family  (Methyl-accepting chemotaxis protein (MCP) signalling domain)  1(5) | Cluster 103465  (Methyl-accepting chemotaxis protein)  1(5) | Cluster 4187767  Cluster Name: Chemotaxis methyl-accepting receptor  1(5) |
|  | **B2S1Z1** | soluble lytic transglycosylase | Transglycosylase -like domain  1(5) | transglycosylase domain-like  1(5) | No result  0 | | lysozyme_like superamily  (Lytic Transglycosylases (SLT))  1(5) | SLT family  (Transglycosylase SLT domain)  1(5) | Cluster 140455  (Soluble lytic transglycosylase)  1(5) | Cluster 4167841  Cluster Name: Bacterial muramidases  (soluble lytic transglycosylase)  1(5) |
|  | **B2S1Z2** | tRNA uridine 5-carboxymethylaminomethyl modification enzyme GidA | tRNA uridine 5-carboxymethylaminomethyl -like domain  1(5) | GidA-like  1(5) | GLUCOSE-INHIBITED DIVISION FAMILY A PROTEIN  1(5) | | GIDA_assoc_3 superfamily  (GidA associated domain 3)  1(5) | GIDA family  (Glucose inhibited division protein A)  1(5) | Cluster 149351  (Glucose inhibited division protein A)  1(5) | Cluster 3800379  (tRNA uridine 5-carboxymethylaminomethyl modification enzyme mnmG)  1(5) |
|  | **B2S1Z3** | adenosine deaminase | Adenosine deaminase -like domain  1(5) | Adenosine/AMP deaminase  1(5) | ADENOSINE DEAMINASE  1(5) | | A_deaminase_N superfamily  (Adenosine/AMP deaminase N-terminal)  1(5) | A_deaminase family  (Adenosine/AMP deaminase)  1(5) | Cluster 145244  (Adenosine deaminase (EC 3.5.4.4) (Adenosine aminohydrolase))  1(5) | Cluster 4095439  Cluster Name: Adenosine deaminase  1(5) |
|  | **B2S1Z9** | peptide chain release factor 1 | Peptide chain release factor 1 -like domain  1(5) | Release factor  1(4) | PEPTIDE CHAIN RELEASE FACTOR 1, MITOCHONDRIAL  1(5) | | PCRF superfamily  (peptide chain release factors)  1(5) | PCRF family  1(5) | Cluster 141939  (Peptide chain release factor 1 (RF-1))  1(5) | Cluster 4106370  (Peptide chain release factor 1)  1(5) |
|  | **B2S200** | protoporphyrinogen oxidase | Release factor glutamine methyltransferase -like domain  1(5) | S-adenosyl-L-methionine-dependent methyltransferase  1(4) | HEMK METHYLTRANSFERASE FAMILY MEMBER 1  1(4) | | AdoMet_MTases  Superamilly  (S-adenosyl-L-methionine-dependent methyltransferase)  1(4) | Methyltransf_26 family  (Methyltransferase domain)  1(4) | Cluster 145161  (protophorphyrinogen oxidase activity)  1(5) | Cluster 4151922  (Protoporphyrinogen oxidase)  1(5) |
|  | **B2S201** | ribonucleotide-diphosphate reductase subunit beta | Ribonucleoside-diphosphate reductase small subunit -like domain  1(5) | Ribonucleotide reductase-like  1(5) | RIBONUCLEOSIDE-DIPHOSPHATE REDUCTASE SUBUNIT M2  1(5) | | Ferritin_like  Superamily  1(5) | Ribonuc_red_sm family  (Ribonucleotide reductase, small chain)  1(5) | Cluster 149119  (Ribonucleoside-diphosphate reductase small chain (EC 1.17.4.1) (Ribonucleotide reductase))  1(5) | Cluster 3614208  Cluster Name: Ribonucleotide reductase  1(5) |
|  | **B2S204** | pyruvate carboxylase subunit B | Biotin synthase -like domain  1(3) | Conserved carboxylase domain  1(4) | CARBOXYLASE:PYRUVATE/ACETYL-COA/PROPIONYL-COA CARBOXYLASE  1(5) | | PYC_OADA family  (Conserved carboxylase domain)  1(5) | PYC_OADA family  (Conserved carboxylase domain)  1(5) | Cluster 140470  (Carboxylase)  1(3) | Cluster 4067452  (Oxaloacetate decarboxylase alpha subunit)  1(5) |
|  | **B2S205** | oxaloacetate decarboxylase, subunit beta | No result  0 | No result  0 | No result  0 | | OAD_beta family  (Na+-transporting oxaloacetate decarboxylase beta subunit)  1(5) | OAD_beta family  (Na+-transporting oxaloacetate decarboxylase beta subunit)  1(5) | Cluster 130697  (Oxaloacetate decarboxylase beta chain (EC 4.1.1.3))  1(5) | Cluster 3987271  Cluster Name: Na+-transporting methylmalonyl-CoA/oxaloacetate decarboxylase, beta subunit  1(5) |
|  | **B2S206** | replicative DNA helicase DnaB | Replicative DNA helicase -like domain  1(5) | N-terminal domain of DnaB helicase  1(5) | REPLICATIVE DNA HELICASE DNAB  1(5) | | DnaB superfamily  (DnaB-like helicase N terminal domain)  1(5) | DnaB family  (DnaB-like helicase N terminal domain)  1(5) | Cluster 138751  (Replicative DNA helicase)  1(5) | Cluster 4108785  Cluster Name: DNA helicase, DnaB type  1(5) |
|  | **B2S208** | 50S ribosomal protein L9 | 50S ribosomal protein L9 -like domain  1(5) | Ribosomal protein L9 C-domain  1(5) | 50S RIBOSOMAL PROTEIN L9  1(5) | | Ribosmal protein L9  1(5) | Ribosomal_L9 family  1(5) | Cluster 154046  (50S ribosomal protein L9)  1(5) | Cluster 4139397  Cluster Name: Ribosomal protein L9, C-terminal  1(5) |
|  | **B2S209** | 30S ribosomal protein S18 | 30S ribosomal protein S18 -like domain  1(5) | Ribosomal protein S18  1(5) | 30S RIBOSOMAL PROTEIN S18  1(5) | | Ribosomal_S18  superfamily  (Ribosomal protein S18)  1(5) | Ribosomal_S18  Family  (Ribosomal protein S18)  1(5) | Cluster 149976  (30S ribosomal protein S18)  1(5) | Cluster 4291602  Cluster Name: Ribosomal protein S18  1(5) |
|  | **B2S210** | single-strand DNA binding protein Ssb | Single-stranded DNA-binding protein -like domain  1(5) | Single strand DNA-binding domain, SSB  1(5) | SINGLE-STRANDED DNA-BINDING PROTEIN  1(5) | | RPA_2b-aaRSs_OBF_like Superfamily  (ssDNA binding activity)  1(4) | SSB family  (Single-strand binding protein family)  1(5) | Cluster 147266  (Single-strand DNA binding protein)  1(5) | Cluster 4269657  Cluster Name: Single-strand DNA-binding  1(5) |
|  | **B2S211** | 30S ribosomal protein S6 | 30S ribosomal protein S6 -like domain  1(5) | Ribosomal protein S6  1(5) | MITOCHONDRIAL 28S RIBOSOMAL PROTEIN S6  1(4) | | Ribosomal_S6 superfamily  (Ribosomal protein S6)  1(5) | Ribosomal_S6 family  (Ribosomal protein S6)  1(5) | Cluster 150821  (30S ribosomal protein S6)  1(5) | Cluster 4334787Clcluster Name: Ribosomal protein S6  1(5) |
|  | **B2S219** | ATP-dependent Clp protease, subunit B | No result  0 | Double Clp-N motif  1(4) | HEAT SHOCK PROTEIN 104  1(5) | | Clp_N family  (Clp amino terminal domain)  1(4) | Clp_N family  (Clp amino terminal domain)  1(4) | Cluster 140711  (ATP-dependent protease (Heat shock protein))  1(5) | Cluster 4148192  cluster Name: ATPase associated with various cellular activities, AAA-2  1(5) |
|  | **B2S222** | sugar ABC transporter, periplasmic binding protein | Maltose/maltodextrin-binding protein -like domain  1(4) | Periplasmic binding protein-like II  1(5) | SN-GLYCEROL-3-PHOSPHATE-BINDING PERIPLASMIC PROTEIN UGPB  1(4) | | SBP_bac_1 superfamily  (Bacterial extracellular solute-binding protein)  1(4) | SBP_bac_1 family  (Bacterial extracellular solute-binding protein)  1(4) | Cluster 138970  (ABC transporter, substrate binding protein)  1(5) | Cluster 4238094  (Sugar ABC transporter, periplasmic binding protein)  1(5) |
|  | **B2S223** | sugar ABC transporter, permease protein | Sulfate transport system permease protein -like domain  1(4) | MetI-like  (Transport)  1(4) | ABC TRANSPORTER PERMEASE PROTEIN  1(5) | | TM_PBP2 superfamily  (Periplasmic Binding Protein (PBP)-dependent ATP-Binding Cassette (ABC) transporters)  1(5) | BPD_transp_1 family  (Binding-protein-dependent transport system inner membrane component)  1(4) | Cluster 139846  (ABC transporter, membrane spanning protein)  1(5) | Cluster 4176631  (Sugar ABC transporter, permease protein)  1(5) |
|  | **B2S224** | sugar ABC transporter, permease protein | Sulfate transport system permease protein -like domain  1(4) | MetI-like  (Transport)  1(4) | INNER MEMBRANE ABC TRANSPORTER PERMEASE PROTEIN YCJP  1(4) | | TM_PBP2 superfamily  (Periplasmic Binding Protein (PBP)-dependent ATP-Binding Cassette (ABC) transporters)  1(5) | BPD_transp_1 family  (Binding-protein-dependent transport system inner membrane component)  1(4) | Cluster 139451  (sugar uptake ABC transporter permease protein)  1(5) | Cluster 4159005  (Sugar ABC transporter, permease protein)  1(5) |
|  | **B2S225** | capsular polysaccharide biosynthesis protein | No result  0 | NAD(P)-binding Rossmann-fold domains  1(4) | NAD DEPENDENT EPIMERASE/DEHYDRATASE  1(4) | | CoA binding superfamily  1(4) | Polysacc_synt_2  Family  (Polysaccharide biosynthesis protein)  1(5) | Cluster 141334 (Capsular polysaccharide biosynthesis protein)  1(5) | Cluster 4168064  Cluster Name: Polysaccharide biosynthesis protein CapD-like  1(5) |
|  | **B2S226** | spore coat polysaccharide biosynthesis protein,  DegT/DnrJ/EryC1/StrS family pyridoxal dependent aminotransferase | No result  0 | PLP-dependent transferases  1(5) | FLAGELLIN-RELATED  1(3) | | AAT_I superamily  (Aspartate aminotransferase (AAT) superfamily (fold type I))  1(5) | DegT_DnrJ_EryC1 family  (DegT/DnrJ/EryC1/StrS aminotransferase family)  1(5) | Cluster 141558  (DegT/DnrJ/EryC1/StrS family protein)  1(5) | Cluster 4167827  Cluster Name: DegT/DnrJ/EryC1/StrS aminotransferase  1(5) |
|  | **B2S228** | quinoline 2-oxidoreductase | Carbon monoxide dehydrogenase small chain  1(4) | CO dehydrogenase ISP C-domain like  1(4) | XANTHINE DEHYDROGENASE FAD-BINDING SUBUNIT  1(5) | | Fer2_2 superfamily  ([2Fe-2S] binding domain)  1(5) | Fer2_2 family  ([2Fe-2S] binding domain)  1(5) | Cluster 143899  (Xanthine dehydrogenase C-terminal subunit)  1(5) | Cluster 4154823  Cluster Name: CO dehydrogenase ISP C-domain like  1(5) |
|  | **B2S230** | formate hydrogenlyase transcriptional activator FhlA | No result  0 | GAF domain like  1(5) | ANAEROBIC NITRIC OXIDE REDUCTASE TRANSCRIPTION REGULATOR NORR  1(4) | | GAF domain  1(5) | Sigma54_activat family  (Sigma-54 interaction domain)  1(4) | Cluster 138630  (Formate hydrogenlyase transcriptional activator)  1(5) | Cluster 4204544  (Formate hydrogenlyase transcriptional activator FhlA)  1(5) |
|  | **B2S233** | PTS system, nitrogen regulatory IIA component | Mannitol-specific phosphotransferase enzyme IIA -like domain  1(5) | IIA domain of mannitol-specific and ntr phosphotransferase EII  1(5) | FRUCTOSE-LIKE PTS SYSTEM EIIBC COMPONENT-RELATED  1(4) | | PTS_IIA_Fru  Superfamily  (PTS_IIA, PTS system, fructose/mannitol specific IIA subunit)  1(5) | PTS_EIIA_2 family  (Phosphoenolpyruvate-dependent sugar phosphotransferase system, EIIA 2)  1(5) | Cluster 138202  (PTS system, mannitol-specific enzyme II)  1(5) | Cluster 4156078  Cluster Name: Mannitol-specific EII; Chain A  1(5) |
|  | **B2S237** | cyclic nucleotide binding protein | cAMP-dependent protein kinase regulatory subunit -like domain  1(5) | cAMP-binding domain-like  1(5) | CAMP-DEPENDENT PROTEIN KINASE REGULATORY CHAIN  1(5) | | CAP_ED superfamily  (cAMP receptor protein)  1(5) | cNMP_binding family  (Cyclic nucleotide-binding domain)  1(5) | Cluster 96513  (Cyclic nucleotide dependent protein kinase)  1(5) | Cluster 4139469  Cluster Name: CAMP-dependent protein kinase complex  1(5) |
|  | **B2S238** | UDP-N-acetylenolpyruvoylglucosamine reductase | UDP-N-acetylenolpyruvoylglucosamine reductase -like domain  1(5) | Uridine diphospho-N-Acetylenolpyruvylglucosamine reductase, MurB, C-terminal domain  1(5) | UDP-N-ACETYLENOLPYRUVOYLGLUCOSAMINE REDUCTASE  1(5) | | MurB_C superfamily  (UDP-N-acetylenolpyruvoylglucosamine reductase, C-terminal domain)  1(5) | MurB_C family  (UDP-N-acetylenolpyruvoylglucosamine reductase, C-terminal domain)  1(5) | Cluster 149719  (UDP-N-acetylenolpyruvoylglucosamine reductase (EC 1.1.1.158))  1(5) | Cluster 4137725  Cluster Name: UDP-N-acetylenolpyruvoylglucosamine reductase, C-terminal  1(5) |
|  | **B2S239** | cysteinyl-tRNA synthetase | Cysteinyl-tRNA synthetase -like domain  1(5) | Class I aminoacyl-tRNA synthetases (RS), catalytic domain  1(4) | CYSTEINYL-TRNA SYNTHETASE  1(5) | | Nt_trans superfamily  (class I amino-acyl tRNA synthetases)  1(4) | tRNA-synt_1e family  (tRNA synthetases class I (C) catalytic domain)  1(4) | Cluster 143743  (Cysteinyl-tRNA synthetase (EC 6.1.1.16))  1(5) | Cluster 4107691  Cluster Name: Cysteinyl-tRNA synthetase, class Ia  1(5) |
|  | **B2S240** | RNA polymerase sigma-24 factor | RNA polymerase sigma factor -like domain  1(5) | Sigma3 and sigma4 domains of RNA polymerase sigma factors  1(4) | SIGMA 19 FACTOR  1(4) | | Sigma70_r2  Family  1(4) | Sigma70_r2  Family  1(4) | Cluster 145893  (RNA polymerase sigma factor)  1(5) | Cluster 4153797  Cluster Name: RNA polymerase sigma factor 70, region 4 type 2  1(4) |
|  | **B2S242** | phosphate acetyltransferase | Phosphate acetyltransferase -like domain  1(5) | Phosphotransacetylase  1(5) | ETHANOLAMINE UTILIZATION PROTEIN EUTD-RELATED  (acetyltransferase activity)  1(4) | | PTA_PTB  superfamily  (Phosphate acetyl/butaryl transferase)  1(5) | PTA_PTB  Family  (Phosphate acetyl/butaryl transferase)  1(5) | Cluster 146989  (Phosphate acetyl/butyryltransferase family protein)  1(5) | Cluster 4077505  Cluster Name: Phosphate acetyltransferase  1(5) |
|  | **B2S244** | dnaK suppressor | No result  0 | DnaK suppressor protein DksA, alpha-hairpin domain  1(5) | No result  0 | | zf-dskA_traR family  (Prokaryotic dksA/traR C4-type zinc finger)  1(4) | zf-dskA_traR family  (Prokaryotic dksA/traR C4-type zinc finger)  1(4) | Cluster 151030  (DnaK deletion suppressor protein)  1(5) | Cluster 4149892  Cluster Name: Zinc finger, DksA/TraR C4-type  1(5) |
|  | **B2S245** | translation initiation factor IF-1 | Translation initiation factor IF-1 -like domain  1(5) | Nucleic acid-binding proteins  1(3) | No result  0 | | S1 like superfamily  (S1_like: Ribosomal protein S1-like RNA-binding domain)  1(4) | eIF-1a family  (Translation initiation factor 1A / IF-1)  1(5) | Cluster 140098  (Translation initiation factor IF-1)  1(5) | Cluster 4210741  Cluster Name: Translation initiation factor IF-1  1(5) |
|  | **B2S246** | heat-shock protein | DNAJ protein -like domain  1(5) | Chaperone J-domain  1(4) | DNAJ LIKE PROTEIN  1(5) | | DnaJ superfamily  1(5) | DnaJ family  1(5) | Cluster 100990 (Heat-shock protein)  1(5) | Cluster 4230148  Cluster Name: Molecular chaperone, heat shock protein, Hsp40, DnaJ  1(5) |
|  | **B2S247** | uridylate kinase | Uridylate kinase -like domain  1(5) | Carbamate kinase-like  1(4) | GLUTAMATE SEMIALDEHYDE DEHYDROGENASE  1(3) | | AAK superfamily  (Amino Acid Kinases)  1(5) | AA_kinase  (Amino acid kinase family)  1(5) | Cluster 146359  (uridylate kinase (EC 2.7.4.-))  1(5) | Cluster 3940125  Cluster Name: Uridylate kinase, archaeal/spirochete, putative  1(5) |
|  | **B2S248** | thioredoxin | SCO1/SenC family protein -like domain  1(3) | Thioredoxin-like  1(5) | THIOREDOXIN PEROXIDASE  1(5) | | Thioredoxin_like superfamily  1(5) | Redoxin family  1(4) | Cluster 140798  (Thioredoxin)  1(5) | Cluster 4186565  Cluster Name: Thioredoxin-like  1(5) |
|  | **B2S249** | cytochrome c biogenesis protein | No result  0 | No result  0 | CYTOCHROME C-TYPE BIOGENESIS CCDA-LIKE CHLOROPLASTIC PROTEIN  1(5) | | DsbC supertfamily  1(3) | DsbD family  (Cytochrome C biogenesis protein transmembrane region)  1(5) | Cluster 140799  (Cytochrome C-type biogenesis protein)  1(5) | Cluster 4073180  Cluster Name: Cytochrome c assembly protein, transmembrane region  1(5) |
|  | **B2S250** | Rep helicase, single-stranded DNA-dependent ATPase | ATP-dependent DNA helicase pcrA -like domain  1(4) | Tandem AAA-ATPase domain  1(4) | DNA HELICASE II  1(4) | | UvrD_C_2 superfamily  1(4) | UvrD-helicase family  (UvrD/REP helicase N-terminal domain)  1(5) | Cluster 142844  (ATP-dependent DNA helicase REP)  1(5) | Cluster 4189843  Cluster Name: DNA helicase, UvrD-like, C-terminal  1(5) |
|  | **B2S251** | ATP-dependent DNA helicase | ATP-dependent DNA helicase -like domain  1(5) | Tandem AAA-ATPase domain  1(4) | DNA HELICASE RECQ FAMILY MEMBER  1(5) | | DEXDc superfamilyu  (DEAD-like helicases superfamily)  1(5) | DEAD family  (DEAD/DEAH box helicase)  1(5) | Cluster 136987  (ATP-dependent DNA helicase)  1(5) | Cluster 4154465  Cluster Name: DNA helicase, ATP-dependent, RecQ type  1(5) |
|  | **B2S252** | 5'-nucleotidase | 5'-nucleotidase -like domain  1(5) | 5'-nucleotidase (syn. UDP-sugar hydrolase), N-terminal domain  1(5) | 5'-NUCLEOTIDASE-RELATED  1(5) | | 5_nucleotid_C superfamily  1(5) | 5_nucleotid_C family  1(5) | Cluster 138753  (5'-nucleotidase)  1(5) | Cluster 4172495  Cluster Name: 5'-nucleotidase (syn. UDP-sugar hydrolase), C-terminal domain  1(5) |
|  | **B2S253** | DNA polymerase I | No result  0 | DNA polymerase I  1(5) | DNA POLYMERASE NU  1(5) | | DNA_pol_A  superfamily  1(5) | DNA_pol_A  Family  1(5) | Cluster 140209  (DNA polymerase I)  1(5) | Cluster 4146670  Cluster Name: DNA-directed DNA polymerase, family A  1(5) |
|  | **B2S254** | carnitine transporter | No result  0 | No result  0 | HIGH-AFFINITY CHOLINE TRANSPORT PROTEIN-RELATED  1(3) | | BCCT superfamily  (transporter)  1(3) | BCCT family  (transporter)  1(3) | Cluster 132699  (carnitine transporter CniT)  1(5) | Cluster 4019199  Cluster Name: BCCT transporter  1(5) |
|  | **B2S255** | protein LicC | No result  0 | Cytidylytransferase  1(5) | SUGAR-1-PHOSPHATE GUANYL TRANSFERASE  1(4) | | APH_ChoK superfamily  (Aminoglycoside 3'-phosphotransferase (APH) and Choline Kinase (ChoK))  1(5) | Choline_kinase family  (Choline/ethanolamine kinase)  1(5) | Cluster- 141583  (Protein licC)  1(5) | Cluster 4204437  Cluster Name: Nucleotidyltransferase activity  (Protein LicC)  1(5) |
|  | **B2S256** | diphosphate--fructose-6-phosphate 1-phosphotransferase | 6-phosphofructokinase -like domain  1(5) | Phosphofructokinase  1(5) | PHOSPHOFRUCTOKINASE  1(5) | | PFK superfamily  (Phosphofructokinase)  1(5) | PFK family  (Phosphofructokinase)  1(5) | Cluster 148678  (Phosphofructokinase)  1(5) | Cluster 3975908  Cluster Name: Pyrophosphate-dependent phosphofructokinase TP0108  1(5) |
|  | **B2S257** | rRNA methylase | 23S rRNA (guanosine-2'-O-)-methyltransferase RlmB -like domain  1(4) | SpoU-like RNA 2'-O ribose methyltransferase  1(5) | RNA METHYLTRANSFERASE  1(5) | | SpoU_methylase superfamily  (SpoU rRNA Methylase family)  1(5) | SpoU_methylase  (SpoU rRNA Methylase family)  1(5) | Cluster 143099  (RRNA methylase)  1(5) | Cluster 4163001  Cluster Name: RNA methyltransferase TrmH, group 3  1(5) |
|  | **B2S259** | RNA polymerase sigma-54 factor | No result  0 | No result  0 | RNA POLYMERASE SIGMA-54 FACTOR  1(5) | | Sigma54_CBD  Supperfamily  1(4) | Sigma54_AID family  (Sigma-54 factor, Activator interacting domain)  1(4) | Cluster 152588 (RNA polymerase sigma-54 factor)  1(5) | Cluster 3989521  Cluster Name: RNA polymerase sigma factor 54, DNA-binding  1(5) |
|  | **B2S260** | aminopeptidase C | Cysteine proteinase -like domain  1(5) | Cysteine proteinases  1(5) | BLEOMYCIN HYDROLASE  1(5) | | Peptidase_C1 superfamily  1(4) | Peptidase_C1_2 family  1(4) | Cluster 150408  (Aminopeptidase)  1(5) | Cluster 4462404  Cluster Name: Peptidase C1B, bleomycin hydrolase  1(5) |
|  | **B2S261** | Lambda CII stability-governing protein, HflK protein | No result  0 | Band 7/SPFH domain  1(4) | | MODULATOR OF FTSH PROTEASE HFLK  1(5) | SPFH like superfamily  (HflK/C)  1(5) | Band_7 family  (SPFH domain / Band 7 family)  1(4) | Cluster 143375  (HFLK protein)  1(5) | Cluster 3999424  Cluster Name: HflK  1(5) |
|  | **B2S262** | Lambda CII stability-governing protein | No result  0 | Band 7/SPFH domain  1(4) | MODULATOR OF FTSH PROTEASE HFLC  1(5) | | SPFH like superfamily  (HflK/C)  1(5) | Band_7 family  (SPFH domain / Band 7 family)  1(4) | Cluster 143375  (HFLC protein)  1(5) | Cluster 4131544  Cluster Name: HflC  1(5) |
|  | **B2S263** | phosphomethypyrimidine kinase | Hydroxyethylthiazole kinase -like domain  1(4) | Ribokinase-like  1(4) | PHOSPHOMETHYLPYRIMIDINE KINASE  1(5) | | Ribokinase_pfkb_like superfamily  1(4) | Phos_pyr_kin family  (Phosphomethylpyrimidine kinase)  1(5) | Cluster 144215  (Phosphomethylpyrimidine kinase)  1(5) | Cluster 4146344  Cluster Name: Phosphomethylpyrimidine kinase  1(5) |
|  | **B2S264** | excinuclease ABC subunit B | UvrABC system protein B -like domain  1(5) | C-terminal UvrC-binding domain of UvrB  1(4) | UVRABC SYSTEM PROTEIN B  1(5) | | UvrB superfamily  (Ultra-violet resistance protein B)  1(5) | UvrB family  (Ultra-violet resistance protein B)  1(5) | Cluster 138856  (UvrABC system protein B)  1(5) | Cluster 4337081  Cluster Name: C-terminal UvrC-binding domain of UvrB  1(5) |
|  | **B2S267** | amino acid ABC transporter, permease protein | D-methionine transport system permease protein -like domain  1(5) | MetI-like  1(5) | D-METHIONINE TRANSPORT SYSTEM PERMEASE PROTEIN METI  1(5) | | TM_PBP2 superfamily  (Transmembrane subunit (TM) found in Periplasmic Binding Protein (PBP)-dependent ATP-Binding Cassette (ABC) transporters)  1(5) | BPD_transp_1 family  (Binding-protein-dependent transport system inner membrane component)  1(4) | Cluster 141382  (ABC transporter permease protein)  1(5) | Cluster 4122255  (Amino acid ABC transporter, permease protein)  1(5) |
|  | **B2S268** | amino acid ABC transporter, ATP-binding protein | Methionine import ATP-binding protein MetN -like domain  1(5) | ABC transporter ATPase domain-like  1(5) | METHIONINE IMPORT ATP-BINDING PROTEIN METN  1(5) | | ABC_ATPase superfamily  (ATP-binding cassette transporter nucleotide-binding domain)  1(5) | ABC_tran family  (ABC transporter)  1(5) | Cluster 137276  (ABC transporter, nucleotide binding/ATPase protein)  1(5) | Cluster 4221499  Cluster Name: ABC transporter-like  1(5) |
|  | **B2S270** | phosphoenolpyruvate carboxykinase | No result  0 | PEP carboxykinase-like  1(5) | PHOSPHOENOLPYRUVATE CARBOXYKINASE  1(5) | | PEPCK_Hprk superfamily  (Phosphoenolpyruvate carboxykinase)  1(5) | PEPCK family  (Phosphoenolpyruvate carboxykinase)  1(5) | Cluster 131470  (Phosphoenolpyruvate carboxykinase (EC 4.1.1.32))  1(5) | Cluster 4634117  Cluster Name: Phosphoenolpyruvate carboxykinase, GTP-utilising  1(5) |
|  | **B2S272** | GTP-dependent nucleic acid-binding protein EngD | GTP-binding protein YchF -like domain  1(5) | G proteins  1(3) | RIBOSOME-BINDING ATPASE YCHF  1(4) | | Ras_like_GTPase  Superfamily  (Rat sarcoma (Ras)-like superfamily of small guanosine triphosphatases (GTPases)  1(3) | YchF-GTPase_C family  1(5) | Cluster 137757  (GTP-binding protein ychF)  1(5) | Cluster 4372094  Cluster Name: GTP1/OBG  1(5) |
|  | **B2S273** | exodeoxyribonuclease | Inpp5b protein -like domain  1(2) | DNase I-like  1(3) | AP ENDONUCLEASE  1(3) | | EEP superfamily  (Exonuclease-Endonuclease-Phosphatase)  1(3) | Exo_endo_phos  Family  (Endonuclease/Exonuclease/phosphatase family)  1(3) | Cluster 141220  (Exodeoxyribonuclease III)  1(5) | Cluster 4377068  Cluster Name: Exodeoxyribonuclease III xth  1(5) |
|  | **B2S288** | K+ transport protein | No result  0 | No result  0 | No result  0 | | TrkH family  (Cation transport protein)  1(4) | TrkH family  (Cation transport protein)  1(4) | Cluster 151550  (K+ transport protein)  1(5) | Cluster 3946306  Cluster Name: Cation transporter  1(5) |
|  | **B2S289** | methylated-DNA-protein-cysteine S-methyltransferase | O-6-alkylguanine-DNA/cysteine-protein -like domain  1(4) | Methylated DNA-protein cysteine methyltransferase domain  1(5) | METHYLATED-DNA--PROTEIN-CYSTEINE METHYLTRANSFERASE  1(5) | | ATase superfamily  1(4) | DNA_binding_1 family  (6-O-methylguanine DNA methyltransferase, DNA binding domain)  1(5) | Cluster 141370  (Methylated-DNA--protein-cysteine methyltransferase)  1(5) | Cluster 4122286  Cluster Name: Methylated-DNA-[protein]-cysteine S-methyltransferase activity  1(5) |
|  | **B2S2A7** | prolyl-tRNA synthetase | No result  0 | Class II aaRS and biotin synthetases  1(5) | PROLINE--TRNA LIGASE, MITOCHONDRIAL-RELATED  1(5) | | Class_II_aaRS_core superfamily  1(5) | tRNA-synt_2b family  (tRNA synthetase class II core domain (G, H, P, S and T))  1(5) | Cluster 127052  (Prolyl-tRNA synthetase )  1(5) | Cluster 4005408  Cluster Name: Prolyl-tRNA synthetase, class IIa, bacterial  1(5) |
|  | **B2S2A9** | Holliday junction DNA helicase RuvB | No result  0 | Helicase DNA-binding domain  1(4) | CHROMOSOME TRANSMISSION FIDELITY PROTEIN 18  1(3) | | RuvB_C superfamily  (Holliday junction DNA helicase ruvB C-terminus)  1(5) | RuvB_C family  (Holliday junction DNA helicase ruvB C-terminus)  1(5) | Cluster 128040  (Holliday junction DNA helicase ruvB)  1(5) | Cluster 4264166  Cluster Name: DNA helicase, Holliday junction RuvB type  1(5) |
|  | **B2S2B0** | ABC transporter, periplasmic binding protein | Light-independent protochlorophyllide reductase -like domain  1(3) | “Helical backbone" metal receptor  (TroA family)  1(5) | ABC-TRANSPORTER METAL-BINDING PROTEIN  1(5) | | TroA family  (Periplasmic solute binding protein family)  1(5) | TroA family  (Periplasmic solute binding protein family)  1(5) | Cluster 140437  (ABC transporter, substrate binding protein)  1(5) | Cluster 4153106  Cluster Name: Adhesin B  (Periplasmic zinc-binding protein troA)  1(5) |
|  | **B2S2B1** | ABC transporter, ATP-binding protein | Zinc import ATP-binding protein ZnuC -like domain  1(4) | ABC transporter ATPase domain-like  1(5) | ABC TRANSPORTER  1(4) | | ABC_ATPase superfamily  (ATP-binding cassette transporter nucleotide-binding domain)  1(5) | ABC_tran family  (ABC transporter)  1(5) | Cluster 137566  (ABC transporter, nucleotide binding/ATPase protein)  1(5) | Cluster 4221499  Cluster Name: ABC transporter-like  1(5) |
|  | **B2S2B2** | ABC transporter, permease protein | Vitamin B12 import system permease protein BtuC -like domain  1(5) | ABC transporter involved in vitamin B12 uptake, BtuC  1(5) | ABC-TRANSPORTER METAL-BINDING PROTEIN  1(5) | | TM_ABC_iron-siderophores_like Superfamily  1(5) | ABC-3 family  (ABC 3 transport family)  1(5) | Cluster 149307  (ABC transporter)  1(5) | Cluster 4136791  Cluster Name: ABC-3  1(5) |
|  | **B2S2B3** | ABC transporter, permease protein | No result  0 | ABC transporter involved in vitamin B12 uptake, BtuC  1(5) | ABC-TRANSPORTER METAL-BINDING PROTEIN  1(5) | | TM_ABC_iron-siderophores_like Superfamily  1(5) | ABC-3 family  (ABC 3 transport family)  1(5) | Cluster 149307  (ABC transporter)  1(5) | Cluster 4136791  Cluster Name: ABC-3  1(5) |
|  | **B2S2B4** | cation-activated repressor protein | Transcriptional regulator slyA -like domain  1(3) | Iron-dependent repressor protein, dimerization domain  1(5) | No result  0 | | Fe_dep_repress  superfaamily  (Iron dependent repressor, N-terminal DNA binding domain)  1(4) | Fe_dep_repress  Family  (Iron dependent repressor, N-terminal DNA binding domain)  1(4) | Cluster 149306  (Iron-dependent repressor)  1(5) | Cluster 4146703  (Cation-activated repressor protein)  1(5) |
|  | **B2S2B5** | phosphoglyceromutase | Phosphoglycerate mutase -like domain  1(5) | Phosphoglycerate mutase-like  1(5) | PHOSPHOGLYCERATE MUTASE  1(5) | | HP superfamily  (Histidine phosphatase domain)  1(3) | His_Phos_1  (Histidine phosphatase superfamily (branch 1))  1(3) | Cluster 140958  (phosphoglycerate mutase)  1(5) | Cluster 4119883  Cluster Name: Phosphoglycerate mutase 1  1(5) |
|  | **B2S2B7** | protein Pfs | 5'-methylthioadenosine/S-adenosylhomocysteine -like domain  1(3) | Purine and uridine phosphorylases | : PROTEIN H14E04.2, ISOFORM C  1(2) | | PNP_UDP_1  (Phosphorylase superfamily)  1(5) | PNP_UDP_1  (Phosphorylase superfamily)  1(5) | Cluster 146158  (nucleoside phoshorylase)  1(5) | Cluster 4187901  Cluster Name: MTA/SAH nucleosidase  1(3) |
|  | **B2S2B8** | lipoprotein, 15 kDa | No result  0 | No result  0 | No result  0 | | FMN_bind family  (FMN-binding domain)  1(5) | FMN_bind family  (FMN-binding domain)  1(5) | Cluster 134377  (15kD lipoprotein)  1(5) | Cluster 3924259  Cluster Name: Major membrane immunogen, tpp15, predicted  1(5) |
|  | **B2S2D1** | SsrA-binding protein | SsrA-binding protein -like domain  1(5) | Small protein B (SmpB)  1(5) | : TMRNA-BINDING COMPONENT OF TRANS-TRANSLATION TAGGING COMPLEX  1(4) | | SmpB superfamily  1(5) | SmpB family  1(5) | Cluster 146778  (SsrA-binding protein)  1(5) | Cluster 4160483  Cluster Name: SsrA-binding protein  1(5) |
|  | **B2S2D2** | signal peptidase I | Signal peptidase I -like domain  1(5) | Type 1 signal peptidase  1(5) | PROTEASE FAMILY S26 MITOCHONDRIAL INNER MEMBRANE PROTEASE-RELATED  1(3) | | Peptidase_S24_S26superfamily  1(4) | Peptidase_S24 family  1(4) | Cluster 82554  (Signal peptidase I (SIP))  1(5) | Cluster 4365129  Cluster Name: Signal peptidase I  1(5) |
|  | **B2S2D3** | oxygen-independent coproporphyrinogen III oxidase | (Dimethylallyl)adenosine tRNA -like domain  1(3) | Oxygen-independent coproporphyrinogen III oxidase HemN  1(5) | RADICAL S-ADENOSYL METHIONINE DOMAIN-CONTAINING PROTEIN 1, MITOCHONDRIAL  1(4) | | HemN_C superfamily  (HemN C-terminal domain)  1(5) | HemN_C family  (HemN C-terminal domain)  1(5) | Cluster 145177  (Oxygen-independent coproporphyrinogen III oxidase)  1(5) | Cluster 4155457  Cluster Name: Putative oxygen-independent coproporphyrinogen III oxidase  1(5) |
|  | **B2S2D4** | elongation factor Tu | Elongation factor Tu -like domain  1(5) | Elongation factors  1(5) | ELONGATION FACTOR TU, MITOCHONDRIAL  1(5) | | Translation_factor_III superfamily  (Elongation factor Tu (EF-Tu) domain II-like proteins)  1(4) | GTP_EFTU family  (Elongation factor Tu GTP binding domain)  1(5) | Cluster 137863  (Elongation factor TU)  1(5) | Cluster 3750444  Cluster Name: Translation elongation factor EFTu/EF1A, bacterial and organelle  1(5) |
|  | **B2S2D5** | 30S ribosomal protein S10 | 30S ribosomal protein S10 -like domain  1(5) | Ribosomal protein S10  1(5) | 30S RIBOSOMAL PROTEIN S10 FAMILY MEMBER  1(5) | | Ribosomal_S10 superfamily  1(5) | Ribosomal_S10 family  1(5) | Cluster 144541  (30S ribosomal protein S10)  1(5) | Cluster 4061736  Cluster Name: Ribosomal protein S10, bacterial  1(5) |
|  | **B2S2D6** | 50S ribosomal protein L3 | Ribosomal protein L3 -like domain  1(5) | Ribosomal protein L3  1(5) | 50S RIBOSOMAL PROTEIN L3  1(5) | | Ribosomal_L3 superfamily  1(5) | Ribosomal_L3 family  1(5) | Cluster 152892  (50S ribosomal protein L3P)  1(5) | Cluster 4437676  Cluster Name: Ribosomal protein L3  1(5) |
|  | **B2S2D7** | 50S ribosomal protein L4 | Ribosomal protein L4 -like domain  1(5) | Ribosomal protein L4  1(5) | 50S RIBOSOMAL PROTEIN L4  1(5) | | Ribosomal_L4  superfamily  (Ribosomal protein L4/L1 family)  1(5) | Ribosomal_L4  (Ribosomal protein L4/L1 family)  1(5) | Cluster 151306  (50S ribosomal protein L4)  1(5) | Cluster 4193105  Cluster Name: Ribosomal protein L4/L1e, bacterial-type  1(5) |
|  | **B2S2D8** | 50S ribosomal protein L23 | 50S ribosomal protein L23 -like domain  1(5) | Ribosomal proteins S24e, L23 and L15e  1(5) | 60S RIBOSOMAL PROTEIN L23A  1(4) | | Ribosomal_L23 superfamily  1(5) | Ribosomal_L23 family  1(5) | Cluster 143276  (50S ribosomal protein L23)  1(5) | Cluster 4366602  Cluster Name: Ribosomal protein L25/L23  1(5) |
|  | **B2S2D9** | 50S ribosomal protein L2 | 50S ribosomal protein L2 -like domain  1(5) | C-terminal domain of ribosomal protein L2  1(4) | 54S RIBOSOMAL PROTEIN RML2, MITOCHONDRIAL  1(4) | | Ribosomal_L2 superfamily  (Ribosomal Proteins L2, RNA binding domain)  1(5) | Ribosomal_L2 family  (Ribosomal Proteins L2, RNA binding domain)  1(5) | Cluster 142863  (50S ribosomal protein L2P)  1(5) | Cluster 4149764  Cluster Name: Ribosomal protein L2, bacterial-type  1(5) |
|  | **B2S2E0** | 30S ribosomal protein S19 | 30S ribosomal protein S19 -like domain  1(5) | Ribosomal protein S19  1(5) | 37S RIBOSOMAL PROTEIN S19, MITOCHONDRIAL  1(4) | | Ribosomal_S19 superfamily  1(5) | Ribosomal_S19 family  1(5) | Cluster 140739  (30S ribosomal protein S19)  1(5) | Cluster 4068229  Cluster Name: 30s Ribosomal Protein S19; Chain A  1(5) |
|  | **B2S2E1** | 50S ribosomal protein L22 | 50S ribosomal protein L22 -like domain  1(5) | Ribosomal protein L22  1(5) | CHLOROPLAST 50S RIBOSOMAL PROTEIN L22-RELATED  1(5) | | Ribosomal_L22 superfamily  1(5) | Ribosomal_L22 family  1(5) | Cluster 111671  (50S ribosomal protein L22)  1(5) | Cluster 4156639  Cluster Name: Ribosomal protein L22, bacterial-typ1(5) |
|  | **B2S2E2** | 30S ribosomal protein S3 | 30S ribosomal protein S3 -like domain  1(5) | Ribosomal protein S3 C-terminal domain  1(5) | 30S RIBOSOMAL PROTEIN S3, CHLOROPLASTIC-RELATED  1(5) | | Ribosomal_S3_C superfamily  (Ribosomal protein S3, C-terminal domain)  1(5) | Ribosomal_S3_C family  (Ribosomal protein S3, C-terminal domain)  1(5) | Cluster 138836  (30S ribosomal protein S3)  1(5) | Cluster 4040040  Cluster Name: Ribosomal protein S3, bacterial  1(5) |
|  | **B2S2F3** | 30S ribosomal protein S5 | No result  0 | Ribosomal protein S5 domain 2-like  1(4) | RIBOSOMAL S SUBUNIT  1(3) | | Ribosomal_S5_C superfamily  (Ribosomal protein S5, C-terminal domain)  1(5) | Ribosomal_S5_C family  (Ribosomal protein S5, C-terminal domain)  1(5) | Cluster 150402  (30S ribosomal protein S5P)  1(5) | Cluster 4239968  Cluster Name: Ribosomal protein S5, C-terminal  1(5) |
|  | **B2S2F4** | 50S ribosomal protein L30 | 50S ribosomal protein L30 -like domain  1(5) | Ribosomal protein L30p/L7e  1(5) | MITOCHONDRIAL RIBOSOMAL PROTEIN L30  1(5) | | Ribosomal_L30_like superfamily  1(5) | Ribosomal_L30  (Ribosomal protein L30p/L7e)  1(5) | Cluster 149387(50S ribosomal protein L30) 1(5) | Cluster 4328974Cluster Name: Ribosomal protein L30, bacterial-type  1(5) |
|  | **B2S2F5** | 50S ribosomal protein L15 | 50S ribosomal protein L15 -like domain  1(5) | Ribosomal proteins L15p and L18e  1(5) | 50S RIBOSOMAL PROTEIN L15  1(5) | | Ribosomal_L18e superfamily  (Ribosomal protein L18e/L15)  1(5) | Ribosomal_L18e family  (Ribosomal protein L18e/L15)  1(5) | Cluster 151381  (50S ribosomal protein L15)  1(5) | Cluster 4378716  Cluster Name: Ribosomal protein L15, bacterial-type  1(5) |
