# Supplementary material for: Identification of Functional Candidates amongst Hypothetical Proteins of Treponema pallidum ssp. pallidum
Source: PLoS One. 2015 Apr 20;10(4):e0124177. doi: 10.1371/journal.pone.0124177 (PMC4403809; doi:10.1371/journal.pone.0124177)
Supplement: S8 Table — (DOC) [file pone.0124177.s008.doc]

| **S.No.**  **Table S8:** List of accuracy, sensitivity, specificity and ROC area of various bioinformatics tools used for predicting function of HPs from *H. influenzae* obtained after ROC analysis. | **Software name** | **Accuracy of prediction** | **Sensitivity** | **Specificity** | **ROC Area** |
| --- | --- | --- | --- | --- | --- |
|  | BLAST | 100% | 100% | N/A | N/A |
|  | HMMER | 97% | 97% | N/A | N/A |
|  | SMART | 100% | 100% | N/A | N/A |
|  | INTERPROSCAN | 97% | 97% | N/A | N/A |
|  | SUPERFAMILY | 88% | 94.6% | 0% | 0.301 |
|  | CATH | 71% | 95.9% | 0% | 0.351 |
|  | PANTHER | 90% | 98.9% | 0% | 0.33 |
|  | Pfam | 96% | 96% | N/A | N/A |
|  | SYSTERS | 98% | 99% | 0% | 0.49 |
|  | CDART | 97% | 97% | N/A | N/A |
|  | ProtoNet | 99% | 99% | N/A | N/A |
|  | Average | 93.91% | 98% |  |  |
