# Supplementary material for: Identification of Functional Candidates amongst Hypothetical Proteins of Treponema pallidum ssp. pallidum
Source: PLoS One. 2015 Apr 20;10(4):e0124177. doi: 10.1371/journal.pone.0124177 (PMC4403809; doi:10.1371/journal.pone.0124177)
Supplement: S9 Table — (DOCX) [file pone.0124177.s009.docx]

| **Table S9: Functionally annotated HPs from *T. pallidum ssp. pallidum* with low level of confidence** | | | | |
| --- | --- | --- | --- | --- |
| **S. No.** | **Protein Name** | **GeneID** | **Uniprot ID** | **Function** |
|  | HP TPASS_0004 | [6333119](http://www.ncbi.nlm.nih.gov/gene/6333119) | **B2S1V4** | RNA binding protein |
|  | HP TPASS_0008 | [6333121](http://www.ncbi.nlm.nih.gov/gene/6333121) | **B2S1V7** | Lipoprotein |
|  | HP TPASS_0010 | [6332864](http://www.ncbi.nlm.nih.gov/gene/6332864) | **B2S1V8** | beta and beta-prime subunits of DNA dependent RNA-polymerase |
|  | HP TPASS_0012 | [6333144](http://www.ncbi.nlm.nih.gov/gene/6333144) | **B2S1W0** | TonB dependent receptor |
|  | HP TPASS_0013 | [6333129](http://www.ncbi.nlm.nih.gov/gene/6333129) | **B2S1W1** | Lipoprotein |
|  | HP TPASS_0014 | [6333761](http://www.ncbi.nlm.nih.gov/gene/6333761) | **B2S1W2** | Transcriptional regulator |
|  | HP TPASS_0021 | [6332872](http://www.ncbi.nlm.nih.gov/gene/6332872) | **B2S1W9** | Helicase C-terminal |
|  | HP TPASS_0031 | [6333177](http://www.ncbi.nlm.nih.gov/gene/6333177) | **B2S1X9** | Thiolase-like |
|  | HP TPASS_0033 | [6333152](http://www.ncbi.nlm.nih.gov/gene/6333152) | **B2S1Y1** | Membrane protein |
|  | HP TPASS_0039 | [6332888](http://www.ncbi.nlm.nih.gov/gene/6332888) | **B2S1Y7** | ATP synthase (F1-ATPase), gamma subunit |
|  | HP TPASS_0041 | [6333169](http://www.ncbi.nlm.nih.gov/gene/6333169) | **B2S1Y9** | Thymidylate synthase-complementing protein Thy1 |
|  | HP TPASS_0047 | [6333759](http://www.ncbi.nlm.nih.gov/gene/6333759) | **B2S1Z5** | Nicotinate mononucleotide:5,6-dimethylbenzimidazole phosphoribosyltransferase (CobT) |
|  | HP TPASS_0059 | [6333167](http://www.ncbi.nlm.nih.gov/gene/6333167) | **B2S207** | oxidation resistance protein |
|  | HP TPASS_0069 | [6333154](http://www.ncbi.nlm.nih.gov/gene/6333154) | **B2S217** | N-acetylmuramoyl-L-alanine amidase |
|  | HP TPASS_0070 | [6333822](http://www.ncbi.nlm.nih.gov/gene/6333822) | **B2S218** | phosphoenolpyruvate-protein phosphotransferase |
|  | HP TPASS_0087 | [6333150](http://www.ncbi.nlm.nih.gov/gene/6333150) | **B2S235** | Tryptophan synthase beta subunit-like PLP-dependent enzymes |
|  | HP TPASS_0088 | [6333814](http://www.ncbi.nlm.nih.gov/gene/6333814) | **B2S236** | Histidine kinase |
|  | HP TPASS_0093 | [6333807](http://www.ncbi.nlm.nih.gov/gene/6333807) | **B2S241** | Transcriptional regulator |
|  | HP TPASS_0110 | [6333793](http://www.ncbi.nlm.nih.gov/gene/6333793) | **B2S258** | excinuclease ABC, A subunit-related protein |
|  | HP TPASS_0118 | [6333788](http://www.ncbi.nlm.nih.gov/gene/6333788) | **B2S266** | PLC-like phosphodiesterases |
|  | HP TPASS_0127 | [6333181](http://www.ncbi.nlm.nih.gov/gene/6333181) | **B2S275** | Hepatitis B viral capsid |
|  | HP TPASS_0128 | [6332902](http://www.ncbi.nlm.nih.gov/gene/6332902) | **B2S276** | Nucleoplasmin-like/VP (viral coat and capsid proteins) |
|  | HP TPASS_0129 | [6333781](http://www.ncbi.nlm.nih.gov/gene/6333781) | **B2S277** | glutamate 5-kinase |
|  | HP TPASS_0130 | [6332866](http://www.ncbi.nlm.nih.gov/gene/6332866) | **B2S278** | Repeat protein K |
|  | HP TPASS_0132 | [6333782](http://www.ncbi.nlm.nih.gov/gene/6333782) | **B2S280** | lantibiotic dehydratase domain-containing protein |
|  | HP TPASS_0133 | [6333784](http://www.ncbi.nlm.nih.gov/gene/6333784) | **B2S281** | Outer membrane protein |
|  | HP TPASS_0134 | [6333136](http://www.ncbi.nlm.nih.gov/gene/6333136) | **B2S282** | Outer membrane protein |
|  | HP TPASS_0135 | [6333778](http://www.ncbi.nlm.nih.gov/gene/6333778) | **B2S283** | Sec7 domain |
|  | HP TPASS_0136 | [6333777](http://www.ncbi.nlm.nih.gov/gene/6333777) | **B2S284** | Outer membrane protein |
|  | HP TPASS_0137 | [6333780](http://www.ncbi.nlm.nih.gov/gene/6333780) | **B2S285** | No significant match found |
|  | HP TPASS_0138 | [6333779](http://www.ncbi.nlm.nih.gov/gene/6333779) | **B2S286** | PAS/PAC sensor protein |
|  | HP TPASS_0148 | [6333201](http://www.ncbi.nlm.nih.gov/gene/6333201) | **B2S295** | Trypsin-like serine proteases |
|  | HP TPASS_0149 | [6333200](http://www.ncbi.nlm.nih.gov/gene/6333200) | **B2S296** | ITPase-like |
|  | HP TPASS_0150 | [6332898](http://www.ncbi.nlm.nih.gov/gene/6332898) | **B2S297** | PTS fructose transporter subunit IIA |
|  | HP TPASS_0159 | [6333192](http://www.ncbi.nlm.nih.gov/gene/6333192) | **B2S2A6** | glutamate--cysteine ligase |
|  | HP TPASS_0161 | [6332897](http://www.ncbi.nlm.nih.gov/gene/6332897) | **B2S2A8** | peptidoglycan glycosyltransferase |
|  | HP TPASS_0169 | [6333795](http://www.ncbi.nlm.nih.gov/gene/6333795) | **B2S2B6** | protein Pfs |
|  | HP TPASS_0172 | [6333604](http://www.ncbi.nlm.nih.gov/gene/6333604) | **B2S2B9** | diguanylate cyclase |
|  | HP TPASS_0173 | [6332822](http://www.ncbi.nlm.nih.gov/gene/6332822) | **B2S2C0** | diguanylate cyclase |
|  | HP TPASS_0174 | [6333039](http://www.ncbi.nlm.nih.gov/gene/6333039) | **B2S2C1** | (Trans)glycosidases |
|  | HP TPASS_0175 | [6333600](http://www.ncbi.nlm.nih.gov/gene/6333600) | **B2S2C2** | Bcr-Abl oncoprotein oligomerization domain |
|  | HP TPASS_0176 | [6333038](http://www.ncbi.nlm.nih.gov/gene/6333038) | **B2S2C3** | ABC transporter ATP-binding protein |
|  | HP TPASS_0177 | [6332821](http://www.ncbi.nlm.nih.gov/gene/6332821) | **B2S2C4** | Clavaminate synthase-like |
|  | HP TPASS_0178 | [6333037](http://www.ncbi.nlm.nih.gov/gene/6333037) | **B2S2C5** | Aconitate hydratase |
|  | HP TPASS_0179 | [6332820](http://www.ncbi.nlm.nih.gov/gene/6332820) | **B2S2C6** | Fe-only hydrogenase |
|  | HP TPASS_0180 | [6333601](http://www.ncbi.nlm.nih.gov/gene/6333601) | **B2S2C7** | dioxygenases related to 2-nitropropane dioxygenase |
|  | HP TPASS_0183 | [6333034](http://www.ncbi.nlm.nih.gov/gene/6333034) | **B2S2D0** | testican-3 isoform 4 |
|  | HP TPASS_0214 | [6333566](http://www.ncbi.nlm.nih.gov/gene/6333566) | **B2S2G2** | Tryptophan synthase beta subunit-like PLP-dependent enzymes |
|  | HP TPASS_0222 | [6333561](http://www.ncbi.nlm.nih.gov/gene/6333561) | **B2S2G9** | methyltransferase domain protein |
|  | HP TPASS_0224 | [6333020](http://www.ncbi.nlm.nih.gov/gene/6333020) | **B2S2H1** | Copper-binding of amyloid precursor, CuBD |
|  | HP TPASS_0232 | [6333552](http://www.ncbi.nlm.nih.gov/gene/6333552) | **B2S2H9** | No significant match found |
|  | HP TPASS_0248 | [6333533](http://www.ncbi.nlm.nih.gov/gene/6333533) | **B2S2J5** | sporulation and spore germination |
|  | HP TPASS_0250a | [6333532](http://www.ncbi.nlm.nih.gov/gene/6333532) | **B2S2J7** | Multiheme cytochrome |
|  | HP TPASS_0258 | [6333004](http://www.ncbi.nlm.nih.gov/gene/6333004) | **B2S2K6** | signal peptide protein |
|  | HP TPASS_0266 | [6333521](http://www.ncbi.nlm.nih.gov/gene/6333521) | **B2S2L4** | Thiamin pyrophosphokinase, catalytic domain |
|  | HP TPASS_0273 | [6333514](http://www.ncbi.nlm.nih.gov/gene/6333514) | **B2S2M1** | membrane protein |
|  | HP TPASS_0278 | [6333511](http://www.ncbi.nlm.nih.gov/gene/6333511) | **B2S2M6** | Protein kinase-like |
|  | HP TPASS_0280 | [6333507](http://www.ncbi.nlm.nih.gov/gene/6333507) | **B2S2M8** | amidohydrolase |
|  | HP TPASS_0281 | [6333509](http://www.ncbi.nlm.nih.gov/gene/6333509) | **B2S2M9** | Class II aaRS and biotin synthetases |
|  | HP TPASS_0284 | [6332797](http://www.ncbi.nlm.nih.gov/gene/6332797) | **B2S2N2** | radical SAM additional 4Fe4S-binding SPASM domain-containing protein |
|  | HP TPASS_0286 | [6332993](http://www.ncbi.nlm.nih.gov/gene/6332993) | **B2S2N4** | glycosyl transferase family |
|  | HP TPASS_0287 | [6333504](http://www.ncbi.nlm.nih.gov/gene/6333504) | **B2S2N5** | SAC3/GANP family protein |
|  | HP TPASS_0293 | [6332990](http://www.ncbi.nlm.nih.gov/gene/6332990) | **B2S2P1** | coat F domain-containing protein |
|  | HP TPASS_0299 | [6332988](http://www.ncbi.nlm.nih.gov/gene/6332988) | **B2S2P7** | sugar ABC superfamily ATP binding cassette transporter |
|  | HP TPASS_0311 | [6332984](http://www.ncbi.nlm.nih.gov/gene/6332984) | **B2S2Q9** | Fe-only hydrogenase |
|  | HP TPASS_0312 | [6333485](http://www.ncbi.nlm.nih.gov/gene/6333485) | **B2S2R0** | Membrane protein |
|  | HP TPASS_0314 | [6333484](http://www.ncbi.nlm.nih.gov/gene/6333484) | **B2S2R2** | Subtilisin-like |
|  | HP TPASS_0315 | [6333480](http://www.ncbi.nlm.nih.gov/gene/6333480) | **B2S2R3** | type I L-asparaginase |
|  | HP TPASS_0318 | [6333478](http://www.ncbi.nlm.nih.gov/gene/6333478) | **B2S2R5** | alpha/beta-Hydrolases |
|  | HP TPASS_0320 | [6333479](http://www.ncbi.nlm.nih.gov/gene/6333479) | **B2S2R7** | membrane lipoprotein |
|  | HP TPASS_0324 | [6332976](http://www.ncbi.nlm.nih.gov/gene/6332976) | **B2S2S1** | outer membrane protein |
|  | HP TPASS_0325 | [6333473](http://www.ncbi.nlm.nih.gov/gene/6333473) | **B2S2S2** | Purine and uridine phosphorylases |
|  | HP TPASS_0332 | [6333464](http://www.ncbi.nlm.nih.gov/gene/6333464) | **B2S2S9** | BRCT domain |
|  | HP TPASS_0338 | [6333456](http://www.ncbi.nlm.nih.gov/gene/6333456) | **B2S2T5** | GHMP Kinase, C-terminal domain |
|  | HP TPASS_0346 | [6333451](http://www.ncbi.nlm.nih.gov/gene/6333451) | **B2S2U3** | Lipoprtein |
|  | HP TPASS_0347 | [6333445](http://www.ncbi.nlm.nih.gov/gene/6333445) | **B2S2U4** | DNA-binding domain |
|  | HP TPASS_0355 | [6333442](http://www.ncbi.nlm.nih.gov/gene/6333442) | **B2S2V2** | CDP-alcohol phosphatidyltransferase |
|  | HP TPASS_0359 | [6332938](http://www.ncbi.nlm.nih.gov/gene/6332938) | **B2S2V6** | 1-deoxy-D-xylulose 5-phosphate reductoisomerase |
|  | HP TPASS_0360 | [6333443](http://www.ncbi.nlm.nih.gov/gene/6333443) | **B2S2V7** | molecular chaperone DnaK |
|  | HP TPASS_0368 | [6333432](http://www.ncbi.nlm.nih.gov/gene/6333432) | **B2S2W5** | ECF subfamily RNA polymerase sigma-24 factor |
|  | HP TPASS_0370 | [6332958](http://www.ncbi.nlm.nih.gov/gene/6332958) | **B2S2W7** | Rhabdovirus spike glycoprotein |
|  | HP TPASS_0375 | [6332945](http://www.ncbi.nlm.nih.gov/gene/6332945) | **B2S2X2** | Homeodomain-like |
|  | HP TPASS_0376 | [6332948](http://www.ncbi.nlm.nih.gov/gene/6332948) | **B2S2X3** | Lipoprotein |
|  | HP TPASS_0377 | [6333403](http://www.ncbi.nlm.nih.gov/gene/6333403) | **B2S2X4** | flagellar basal body-associated protein FliL |
|  | HP TPASS_0382 | [6333393](http://www.ncbi.nlm.nih.gov/gene/6333393) | **B2S2X9** | cell division protein |
|  | HP TPASS_0408 | [6333557](http://www.ncbi.nlm.nih.gov/gene/6333557) | **B2S305** | chromosome segregation ATPase domain protein |
|  | HP TPASS_0409 | [6333542](http://www.ncbi.nlm.nih.gov/gene/6333542) | **B2S306** | Cytochrome P450 |
|  | HP TPASS_0415 | [6333643](http://www.ncbi.nlm.nih.gov/gene/6333643) | **B2S312** | pyrroline-5-carboxylate synthetase-like protein |
|  | HP TPASS_0420 | [6333654](http://www.ncbi.nlm.nih.gov/gene/6333654) | **B2S317** | acid phosphatase |
|  | HP TPASS_0422 | [6333057](http://www.ncbi.nlm.nih.gov/gene/6333057) | **B2S319** | tyrosine phosphatase |
|  | HP TPASS_0425 | [6333623](http://www.ncbi.nlm.nih.gov/gene/6333623) | **B2S322** | GTP cyclohydrolase |
|  | HP TPASS_0432 | [6333022](http://www.ncbi.nlm.nih.gov/gene/6333022) | **B2S329** | serine/threonine protein kinase Sgk2 |
|  | HP TPASS_0437 | [6333558](http://www.ncbi.nlm.nih.gov/gene/6333558) | **B2S333** | multidrug resistance outer membrane protein |
|  | HP TPASS_0443 | [6333088](http://www.ncbi.nlm.nih.gov/gene/6333088) | **B2S339** | transcriptional regulator |
|  | HP TPASS_0451 | [6333692](http://www.ncbi.nlm.nih.gov/gene/6333692) | **B2S347** | uracil phosphoribosyltransferase |
|  | HP TPASS_0453 | [6333093](http://www.ncbi.nlm.nih.gov/gene/6333093) | **B2S349** | sugar ABC transporter substrate-binding protein |
|  | HP TPASS_0454 | [6332846](http://www.ncbi.nlm.nih.gov/gene/6332846) | **B2S350** | type IV pilus assembly PilZ |
|  | HP TPASS_0455 | [6333085](http://www.ncbi.nlm.nih.gov/gene/6333085) | **B2S351** | Fibronectin type III |
|  | HP TPASS_0456 | [6333691](http://www.ncbi.nlm.nih.gov/gene/6333691) | **B2S352** | Clavaminate synthase-like |
|  | HP TPASS_0457 | [6333690](http://www.ncbi.nlm.nih.gov/gene/6333690) | **B2S353** | DNA polymerase III subunit delta |
|  | HP TPASS_0462 | [6332845](http://www.ncbi.nlm.nih.gov/gene/6332845) | **B2S358** | Subtilisin-like |
|  | HP TPASS_0463 | [6333687](http://www.ncbi.nlm.nih.gov/gene/6333687) | **B2S359** | Heme-dependent peroxidases |
|  | HP TPASS_0465 | [6333082](http://www.ncbi.nlm.nih.gov/gene/6333082) | **B2S361** | peptidase M23 |
|  | HP TPASS_0466 | [6332844](http://www.ncbi.nlm.nih.gov/gene/6332844) | **B2S362** | "Helical backbone" metal receptor |
|  | HP TPASS_0467 | [6333685](http://www.ncbi.nlm.nih.gov/gene/6333685) | **B2S363** | Clavaminate synthase-like |
|  | HP TPASS_0473 | [6333080](http://www.ncbi.nlm.nih.gov/gene/6333080) | **B2S368** | ribose/galactose ABC transporter permease |
|  | HP TPASS_0479 | [6333680](http://www.ncbi.nlm.nih.gov/gene/6333680) | **B2S374** | ABC transporter ATP-binding protein |
|  | HP TPASS_0480 | [6333078](http://www.ncbi.nlm.nih.gov/gene/6333078) | **B2S375** | Pyruvoyl dependent aspartate decarboxylase |
|  | HP TPASS_0481 | [6333106](http://www.ncbi.nlm.nih.gov/gene/6333106) | **B2S376** | chromosome segregation ATPase |
|  | HP TPASS_0482 | [6333677](http://www.ncbi.nlm.nih.gov/gene/6333677) | **B2S377** | 4-helical cytokine |
|  | HP TPASS_0490 | [6333758](http://www.ncbi.nlm.nih.gov/gene/6333758) | **B2S385** | antirepressor protein |
|  | HP TPASS_0491 | [6333714](http://www.ncbi.nlm.nih.gov/gene/6333714) | **B2S386** | aminodeoxychorismate lyase |
|  | HP TPASS_0503 | [6332849](http://www.ncbi.nlm.nih.gov/gene/6332849) | **B2S397** | Uridine kinases |
|  | HP TPASS_0504 | [6333708](http://www.ncbi.nlm.nih.gov/gene/6333708) | **B2S398** | No significant match found |
|  | HP TPASS_0535 | [6333736](http://www.ncbi.nlm.nih.gov/gene/6333736) | **B2S3C7** | E3 ubiquitin-protein ligase |
|  | HP TPASS_0539 | [6333738](http://www.ncbi.nlm.nih.gov/gene/6333738) | **B2S3D1** | RTA-like protein |
|  | HP TPASS_0552 | [6333728](http://www.ncbi.nlm.nih.gov/gene/6333728) | **B2S3E4** | foldase PrsA |
|  | HP TPASS_0553 | [6332854](http://www.ncbi.nlm.nih.gov/gene/6332854) | **B2S3E5** | Membrane protein |
|  | HP TPASS_0557 | [6333748](http://www.ncbi.nlm.nih.gov/gene/6333748) | **B2S3E9** | ABC transporter substrate-binding protein |
|  | HP TPASS_0564 | [6332855](http://www.ncbi.nlm.nih.gov/gene/6332855) | **B2S3F6** | E2 regulatory, transactivation domain |
|  | HP TPASS_0573 | [6333210](http://www.ncbi.nlm.nih.gov/gene/6333210) | **B2S3G5** | retinal-specific ATP-binding cassette transporter-like |
|  | HP TPASS_0577 | [6333327](http://www.ncbi.nlm.nih.gov/gene/6333327) | **B2S3G8** | Peptidase S8 and S53 subtilisin kexin sedolisin |
|  | HP TPASS_0579 | [6333325](http://www.ncbi.nlm.nih.gov/gene/6333325) | **B2S3H0** | Argininosuccinate synthetase |
|  | HP TPASS_0583 | [6333238](http://www.ncbi.nlm.nih.gov/gene/6333238) | **B2S3H4** | ubiquitin C-terminal hydrolase 3 |
|  | HP TPASS_0584 | [6332905](http://www.ncbi.nlm.nih.gov/gene/6332905) | **B2S3H5** | glutamate-1-semialdehyde aminotransferase |
|  | HP TPASS_0587 | [6333112](http://www.ncbi.nlm.nih.gov/gene/6333112) | **B2S3H8** | DNA-directed DNA polymerase III delta subunit |
|  | HP TPASS_0590 | [6333322](http://www.ncbi.nlm.nih.gov/gene/6333322) | **B2S3I1** | ribosomal protein |
|  | HP TPASS_0593 | [6333318](http://www.ncbi.nlm.nih.gov/gene/6333318) | **B2S3I4** | 3-oxoacyl-[acyl-carrier protein] reductase |
|  | HP TPASS_0594 | [6333353](http://www.ncbi.nlm.nih.gov/gene/6333353) | **B2S3I5** | signal peptide protein |
|  | HP TPASS_0598 | [6333594](http://www.ncbi.nlm.nih.gov/gene/6333594) | **B2S3I8** | WD domain, G-beta repeat protein |
|  | HP TPASS_0607 | [6333304](http://www.ncbi.nlm.nih.gov/gene/6333304) | **B2S3J7** | Trypsin-like serine protease |
|  | HP TPASS_0617 | [6333229](http://www.ncbi.nlm.nih.gov/gene/6333229) | **B2S3K7** | pyrogenic exotoxin B |
|  | HP TPASS_0618 | [6332796](http://www.ncbi.nlm.nih.gov/gene/6332796) | **B2S3K8** | proto-oncogene c-Rel |
|  | HP TPASS_0619 | [6333792](http://www.ncbi.nlm.nih.gov/gene/6333792) | **B2S3K9** | Fe,Mn superoxide dismutase |
|  | HP TPASS_0629 | [6332914](http://www.ncbi.nlm.nih.gov/gene/6332914) | **B2S3L9** | 2-dehydro-3-deoxygluconokinase |
|  | HP TPASS_0638 | [6333307](http://www.ncbi.nlm.nih.gov/gene/6333307) | **B2S3M7** | Na+-driven multidrug efflux pump |
|  | HP TPASS_0645 | [6333236](http://www.ncbi.nlm.nih.gov/gene/6333236) | **B2S3N4** | Thiamin diphosphate-binding fold |
|  | HP TPASS_0646 | [6332969](http://www.ncbi.nlm.nih.gov/gene/6332969) | **B2S3N5** | NHL repeat containing protein |
|  | HP TPASS_0656 | [6333247](http://www.ncbi.nlm.nih.gov/gene/6333247) | **B2S3P5** | Gamma-aminobutyrate:alpha-ketoglutarate aminotransferase |
|  | HP TPASS_0661 | [6333816](http://www.ncbi.nlm.nih.gov/gene/6333816) | **B2S3Q0** | golgi trafficking protein GRIP |
|  | HP TPASS_0665 | [6333258](http://www.ncbi.nlm.nih.gov/gene/6333258) | **B2S3Q4** | peptidase T |
|  | HP TPASS_0666 | [6333321](http://www.ncbi.nlm.nih.gov/gene/6333321) | **B2S3Q5** | ABC multidrug transporter |
|  | HP TPASS_0668 | [6333232](http://www.ncbi.nlm.nih.gov/gene/6333232) | **B2S3Q7** | YGGT family protein |
|  | HP TPASS_0676 | [6333667](http://www.ncbi.nlm.nih.gov/gene/6333667) | **B2S3R5** | carbon monoxide dehydrogenase |
|  | HP TPASS_0677 | [6333334](http://www.ncbi.nlm.nih.gov/gene/6333334) | **B2S3R6** | RIO1-domain-containing protein |
|  | HP TPASS_0678 | [6332908](http://www.ncbi.nlm.nih.gov/gene/6332908) | **B2S3R7** | glutamate-1-semialdehyde aminotransferase |
|  | HP TPASS_0679 | [6333756](http://www.ncbi.nlm.nih.gov/gene/6333756) | **B2S3R8** | C-C motif chemokine 20 isoform 1 precursor |
|  | HP TPASS_0690 | [6333598](http://www.ncbi.nlm.nih.gov/gene/6333598) | **B2S3S9** | Subtilisin-like |
|  | HP TPASS_0693 | [6332915](http://www.ncbi.nlm.nih.gov/gene/6332915) | **B2S3T2** | O-succinylbenzoate-CoA ligase |
|  | HP TPASS_0697 | [6333705](http://www.ncbi.nlm.nih.gov/gene/6333705) | **B2S3T6** | 2-polyprenylphenol hydroxylase |
|  | HP TPASS_0698 | [6333741](http://www.ncbi.nlm.nih.gov/gene/6333741) | **B2S3T7** | Lipoprotein |
|  | HP TPASS_0700 | [6333744](http://www.ncbi.nlm.nih.gov/gene/6333744) | **B2S3T9** | UBA/THIF-type NAD/FAD binding protein |
|  | HP TPASS_0703 | [6333224](http://www.ncbi.nlm.nih.gov/gene/6333224) | **B2S3U2** | M23B subfamily peptidase |
|  | HP TPASS_0707 | [6332904](http://www.ncbi.nlm.nih.gov/gene/6332904) | **B2S3U6** | "Helical backbone" metal receptor |
|  | HP TPASS_0708 | [6333664](http://www.ncbi.nlm.nih.gov/gene/6333664) | **B2S3U7** | rhomboid family protein |
|  | HP TPASS_0711 | [6333213](http://www.ncbi.nlm.nih.gov/gene/6333213) | **B2S3V0** | ABC transporter-like protein |
|  | HP TPASS_0723 | [6333254](http://www.ncbi.nlm.nih.gov/gene/6333254) | **B2S3W2** | ribosomal RNA large subunit methyltransferase |
|  | HP TPASS_0744 | [6333462](http://www.ncbi.nlm.nih.gov/gene/6333462) | **B2S3Y3** | Ribosomal protein |
|  | HP TPASS_0747 | [6333825](http://www.ncbi.nlm.nih.gov/gene/6333825) | **B2S3Y6** | RNA modification GTPase TrmE |
|  | HP TPASS_0749 | [6333826](http://www.ncbi.nlm.nih.gov/gene/6333826) | **B2S3Y8** | RNA polymerase beta subunit |
|  | HP TPASS_0753 | [6333840](http://www.ncbi.nlm.nih.gov/gene/6333840) | **B2S3Z2** | major facilitator transporter |
|  | HP TPASS_0759 | [6333293](http://www.ncbi.nlm.nih.gov/gene/6333293) | **B2S3Z8** | penicillin-binding protein |
|  | HP TPASS_0761 | [6333568](http://www.ncbi.nlm.nih.gov/gene/6333568) | **B2S400** | penicillin-binding protein |
|  | HP TPASS_0762 | [6333245](http://www.ncbi.nlm.nih.gov/gene/6333245) | **B2S401** | PLP-dependent transferase |
|  | HP TPASS_0763 | [6333700](http://www.ncbi.nlm.nih.gov/gene/6333700) | **B2S402** | TPR repeat-containing response regulator |
|  | HP TPASS_0766 | [6333291](http://www.ncbi.nlm.nih.gov/gene/6333291) | **B2S405** | (Trans)glycosidases |
|  | HP TPASS_0772 | [6333851](http://www.ncbi.nlm.nih.gov/gene/6333851) | **B2S411** | LysR family transcriptional regulator |
|  | HP TPASS_0781 | [6332814](http://www.ncbi.nlm.nih.gov/gene/6332814) | **B2S420** | peptidase M23 |
|  | HP TPASS_0783 | [6332818](http://www.ncbi.nlm.nih.gov/gene/6332818) | **B2S422** | Pyrrolo-quinoline quinone |
|  | HP TPASS_0787 | [6332883](http://www.ncbi.nlm.nih.gov/gene/6332883) | **B2S426** | cation diffusion facilitator family transporter |
|  | HP TPASS_0788 | [6332892](http://www.ncbi.nlm.nih.gov/gene/6332892) | **B2S427** | SPFH domain-containing protein |
|  | HP TPASS_0789 | [6333842](http://www.ncbi.nlm.nih.gov/gene/6333842) | **B2S428** | sigma E regulatory protein MucB/RseB |
|  | HP TPASS_0791 | [6333844](http://www.ncbi.nlm.nih.gov/gene/6333844) | **B2S430** | tRNA (Guanine37-N(1)-) methyltransferase |
|  | HP TPASS_0793 | [6333843](http://www.ncbi.nlm.nih.gov/gene/6333843) | **B2S432** | ARM repeat containing protein |
|  | HP TPASS_0795 | [6333329](http://www.ncbi.nlm.nih.gov/gene/6333329) | **B2S434** | S-adenosylmethionine synthetase |
|  | HP TPASS_0799 | [6332936](http://www.ncbi.nlm.nih.gov/gene/6332936) | **B2S438** | lytic transglycosylase subunit |
|  | HP TPASS_0802 | [6333382](http://www.ncbi.nlm.nih.gov/gene/6333382) | **B2S441** | glycerol kinase protein |
|  | HP TPASS_0811 | [6333372](http://www.ncbi.nlm.nih.gov/gene/6333372) | **B2S450** | No significant match found |
|  | HP TPASS_0813 | [6333384](http://www.ncbi.nlm.nih.gov/gene/6333384) | **B2S451** | small-conductance mechanosensitive channel |
|  | HP TPASS_0816 | [6333370](http://www.ncbi.nlm.nih.gov/gene/6333370) | **B2S454** | plasma membrane sodium ion/proton antiporter Sod2 |
|  | HP TPASS_0818 | [6332939](http://www.ncbi.nlm.nih.gov/gene/6332939) | **B2S456** | PTS fructose transporter subunit IIA, partial |
|  | HP TPASS_0825 | [6332944](http://www.ncbi.nlm.nih.gov/gene/6332944) | **B2S463** | tRNA pseudouridine synthase A |
|  | HP TPASS_0827 | [6333402](http://www.ncbi.nlm.nih.gov/gene/6333402) | **B2S465** | YbbR-like protein |
|  | HP TPASS_0829 | [6332946](http://www.ncbi.nlm.nih.gov/gene/6332946) | **B2S467** | RNA 3'-terminal phosphate cyclase |
|  | HP TPASS_0833 | [6333408](http://www.ncbi.nlm.nih.gov/gene/6333408) | **B2S471** | LPXTG-motif cell wall anchor domain-containing protein |
|  | HP TPASS_0836 | [6333410](http://www.ncbi.nlm.nih.gov/gene/6333410) | **B2S474** | pilus modification protein PilQ |
|  | HP TPASS_0839 | [6333413](http://www.ncbi.nlm.nih.gov/gene/6333413) | **B2S477** | transposase |
|  | HP TPASS_0845 | [6333420](http://www.ncbi.nlm.nih.gov/gene/6333420) | **B2S483** | YaiI/YqxD family protein |
|  | HP TPASS_0847 | [6333421](http://www.ncbi.nlm.nih.gov/gene/6333421) | **B2S485** | Maf transcription factor |
|  | HP TPASS_0855 | [6333430](http://www.ncbi.nlm.nih.gov/gene/6333430) | **B2S493** | Protein prenylyltransferase |
|  | HP TPASS_0856 | [6333429](http://www.ncbi.nlm.nih.gov/gene/6333429) | **B2S494** | FYVE/PHD zinc finger |
|  | HP TPASS_0857 | [6333431](http://www.ncbi.nlm.nih.gov/gene/6333431) | **B2S495** | (E)-beta-farnesene synthase-like |
|  | HP TPASS_0858 | [6332959](http://www.ncbi.nlm.nih.gov/gene/6332959) | **B2S496** | Carbamoyl phosphate synthetase, small subunit |
|  | HP TPASS_0859 | [6332960](http://www.ncbi.nlm.nih.gov/gene/6332960) | **B2S497** | Outer membrane protein |
|  | HP TPASS_0865 | [6333416](http://www.ncbi.nlm.nih.gov/gene/6333416) | **B2S4A3** | TPR-like |
|  | HP TPASS_0867 | [6333369](http://www.ncbi.nlm.nih.gov/gene/6333369) | **B2S4A4** | PHD-finger domain-containing protein |
|  | HP TPASS_0869 | [6332926](http://www.ncbi.nlm.nih.gov/gene/6332926) | **B2S4A6** | cAMP-binding protein |
|  | HP TPASS_0871 | [6333367](http://www.ncbi.nlm.nih.gov/gene/6333367) | **B2S4A8** | Porin |
|  | HP TPASS_0873 | [6333065](http://www.ncbi.nlm.nih.gov/gene/6333065) | **B2S4B0** | Ubiquitin carboxyl-terminal hydrolase |
|  | HP TPASS_0874 | [6333659](http://www.ncbi.nlm.nih.gov/gene/6333659) | **B2S4B1** | ABC transporter |
|  | HP TPASS_0878 | [6333206](http://www.ncbi.nlm.nih.gov/gene/6333206) | **B2S4B5** | lysosomal alpha-mannosidase-like |
|  | HP TPASS_0895 | [6333645](http://www.ncbi.nlm.nih.gov/gene/6333645) | **B2S4D2** | CrcB family protein |
|  | HP TPASS_0896 | [6333642](http://www.ncbi.nlm.nih.gov/gene/6333642) | **B2S4D3** | ATP synthase CF1 alpha subunit |
|  | HP TPASS_0904 | [6333639](http://www.ncbi.nlm.nih.gov/gene/6333639) | **B2S4E1** | sialic acid synthase |
|  | HP TPASS_0910 | [6333632](http://www.ncbi.nlm.nih.gov/gene/6333632) | **B2S4E7** | glycosyltransferase-like protein |
|  | HP TPASS_0914 | [6333631](http://www.ncbi.nlm.nih.gov/gene/6333631) | **B2S4F1** | Fe-S oxidoreductase |
|  | HP TPASS_0916 | [6333629](http://www.ncbi.nlm.nih.gov/gene/6333629) | **B2S4F3** | iron-only hydrogenase maturation protein |
|  | HP TPASS_0918 | [6333630](http://www.ncbi.nlm.nih.gov/gene/6333630) | **B2S4F5** | phosphatidate cytidylyltransferase |
|  | HP TPASS_0922 | [6333625](http://www.ncbi.nlm.nih.gov/gene/6333625) | **B2S4F9** | TGS-like protein |
|  | HP TPASS_0927 | [6333046](http://www.ncbi.nlm.nih.gov/gene/6333046) | **B2S4G4** | AraC family transcriptional regulator |
|  | HP TPASS_0928 | [6333045](http://www.ncbi.nlm.nih.gov/gene/6333045) | **B2S4G5** | PpiC-type peptidyl-prolyl cis-trans isomerase |
|  | HP TPASS_0929 | [6332826](http://www.ncbi.nlm.nih.gov/gene/6332826) | **B2S4G6** | PpiC-type peptidyl-prolyl cis-trans isomerase |
|  | HP TPASS_0930 | [6333616](http://www.ncbi.nlm.nih.gov/gene/6333616) | **B2S4G7** | GroES-like protein |
|  | HP TPASS_0932 | [6333619](http://www.ncbi.nlm.nih.gov/gene/6333619) | **B2S4G9** | Crp/Fnr family transcription regulator |
|  | HP TPASS_0938 | [6333610](http://www.ncbi.nlm.nih.gov/gene/6333610) | **B2S4H5** | NAD dependent epimerase/dehydratase family protein |
|  | HP TPASS_0940 | [6332824](http://www.ncbi.nlm.nih.gov/gene/6332824) | **B2S4H7** | pyruvate oxidoreductase |
|  | HP TPASS_0941 | [6333607](http://www.ncbi.nlm.nih.gov/gene/6333607) | **B2S4H8** | NADPH-protochlorophyllide oxidoreductase |
|  | HP TPASS_0950 | [6333833](http://www.ncbi.nlm.nih.gov/gene/6333833) | **B2S4I7** | glutamate dehydrogenase |
|  | HP TPASS_0955 | [6333111](http://www.ncbi.nlm.nih.gov/gene/6333111) | **B2S4J2** | Cysteine proteinase |
|  | HP TPASS_0956 | [6333094](http://www.ncbi.nlm.nih.gov/gene/6333094) | **B2S4J3** | "Helical backbone" metal receptor |
|  | HP TPASS_0966 | [6333296](http://www.ncbi.nlm.nih.gov/gene/6333296) | **B2S4K3** | Outer membrane protein |
|  | HP TPASS_0967 | [6333243](http://www.ncbi.nlm.nih.gov/gene/6333243) | **B2S4K4** | Immunoglobulin-like beta-sandwich |
|  | HP TPASS_0968 | [6333257](http://www.ncbi.nlm.nih.gov/gene/6333257) | **B2S4K5** | chemotaxis protein |
|  | HP TPASS_0969 | [6333242](http://www.ncbi.nlm.nih.gov/gene/6333242) | **B2S4K6** | Outer membrane efflux protein |
|  | HP TPASS_0970 | [6333556](http://www.ncbi.nlm.nih.gov/gene/6333556) | **B2S4K7** | Ribonuclease H-like |
|  | HP TPASS_0974 | [6333283](http://www.ncbi.nlm.nih.gov/gene/6333283) | **B2S4L1** | Anti-sigma-28 factor, FlgM |
|  | HP TPASS_0976 | [6333286](http://www.ncbi.nlm.nih.gov/gene/6333286) | **B2S4L3** | Prolyl oligopeptidase family |
|  | HP TPASS_0983 | [6332999](http://www.ncbi.nlm.nih.gov/gene/6332999) | **B2S4M0** | iron ABC transporter ATP-binding protein |
|  | HP TPASS_0987 | [6333031](http://www.ncbi.nlm.nih.gov/gene/6333031) | **B2S4M4** | integral membrane protein |
|  | HP TPASS_0992 | [6333237](http://www.ncbi.nlm.nih.gov/gene/6333237) | **B2S4M9** | L-Ala-D/L-Glu epimerase |
|  | HP TPASS_0996 | [6333301](http://www.ncbi.nlm.nih.gov/gene/6333301) | **B2S4N3** | cyclic nucleotide-binding domain protein |
|  | HP TPASS_1000 | [6333253](http://www.ncbi.nlm.nih.gov/gene/6333253) | **B2S4N7** | Lumazine synthase |
|  | HP TPASS_1001 | [6333297](http://www.ncbi.nlm.nih.gov/gene/6333297) | **B2S4N8** | trypsin-like serine protease |
|  | HP TPASS_1002 | [6333862](http://www.ncbi.nlm.nih.gov/gene/6333862) | **B2S4N9** | Protein of unknown function DUF2259, secreted |
|  | HP TPASS_1003 | [6333255](http://www.ncbi.nlm.nih.gov/gene/6333255) | **B2S4P0** | Polyketide Synthase III |
|  | HP TPASS_1014 | [6333873](http://www.ncbi.nlm.nih.gov/gene/6333873) | **B2S4Q1** | tetratricopeptide repeat protein |
|  | HP TPASS_1030 | [6333231](http://www.ncbi.nlm.nih.gov/gene/6333231) | **B2S4R7** | eukaryotic translation initiation factor 4 gamma 1-like |
